# Owl Monkey *Alu* Insertion Polymorphisms and *Aotus* Phylogenetics

**DOI:** 10.3390/genes13112069

**Published:** 2022-11-08

**Authors:** Jessica M. Storer, Jerilyn A. Walker, Lydia C. Rewerts, Morgan A. Brown, Thomas O. Beckstrom, Scott W. Herke, Christian Roos, Mark A. Batzer

**Affiliations:** 1 Department of Biological Sciences, Louisiana State University, 202 Life Sciences Building, Baton Rouge, LA 70803, USA; 2 Institute for Systems Biology, Seattle, WA 98109, USA; 3 Department of Oral and Maxillofacial Surgery, University of Washington, 1959 NE Pacific Street, Health Sciences Building B-241, Seattle, WA 98195, USA; 4 Gene Bank of Primates and Primate Genetics Laboratory, German Primate Center, Leibniz Institute for Primate Research, 37077 Göttingen, Germany

**Keywords:** Aotidae, owl monkey, *Alu*, *Aotus*, phylogeny, Platyrrhini

## Abstract

Owl monkeys (genus *Aotus*), or “night monkeys” are platyrrhine primates in the Aotidae family. Early taxonomy only recognized one species, *Aotus trivirgatus*, until 1983, when Hershkovitz proposed nine unique species designations, classified into red-necked and gray-necked species groups based predominately on pelage coloration. Recent studies questioned this conventional separation of the genus and proposed designations based on the geographical location of wild populations. *Alu* retrotransposons are a class of mobile element insertion (MEI) widely used to study primate phylogenetics. A scaffold-level genome assembly for one *Aotus* species, *Aotus nancymaae* [Anan_2.0], facilitated large-scale ascertainment of nearly 2000 young lineage-specific *Alu* insertions. This study provides candidate oligonucleotides for locus-specific PCR assays for over 1350 of these elements. For 314 *Alu* elements across four taxa with multiple specimens, PCR analyses identified 159 insertion polymorphisms, including 21 grouping *A. nancymaae* and *Aotus azarae* (red-necked species) as sister taxa, with *Aotus vociferans* and *A. trivirgatus* (gray-necked) being more basal. DNA sequencing identified five novel *Alu* elements from three different taxa. The *Alu* datasets reported in this study will assist in species identification and provide a valuable resource for *Aotus* phylogenetics, population genetics and conservation strategies when applied to wild populations.

## 1. Introduction

Owl monkeys (*Aotus* spp.) are small, neotropical platyrrhine primates (monkeys found in the Americas) within the Aotidae family; they are often referred to as night monkeys and are widespread across South America and Panama [1,2]. The nocturnal habits of owl monkeys have made them difficult to study in the field compared to other platyrrhine primates, and the morphological similarities of *Aotus* species have led to frequent misidentifications [2,3]. Early taxonomic studies of owl monkeys only recognized one species, designated as *A. trivirgatus*, until 1983, when Hershkovitz designated nine species based predominately on pelage coloration and karyotyping and proposed the separation of “red-necked” and “gray-necked” species groups [4]. Hershkovitz described the “gray-necked” group as having the “side of neck grayish agouti to brownish agouti” in pelage and included species “*Aotus lemurinus* (with subspecies *A. 1. lemurinus* (I. Geoffroy) and *A. 1. griseimembra* (Elliot)), *A. vociferans* (Spix), *A. trivirgatus* (Humboldt) and *A. brumbacki*, a species previously misidentified with *A. azarae*” and named in honor of Dr. Brumback [4]. By contrast, Hershkovitz described the “red-necked” group as having the “side of neck partly to entirely orange or yellowish like that of chest” and included species “*Aotus miconax* (Thomas), *A. nigriceps* (Dollman), *A. infulatus* (Olfers), *A. azarae* (with subspecies *A. a. azarae* (Humboldt) and *A. a. boliviensis* (Elliot)) and *A. nancymai*, the species formerly believed to represent *A. trivirgatus* of the first group” and named in honor of Dr. Ma [4]. The biogeographical data provided in further support of these designations described that gray-necked species are located north of the Solimões-Amazonas River, and the red-necked species are located south of the river with the exception of *A. nancymaae* (spelled as *A. nancymai* in [4]) (red-necked) and *A. vociferans* (gray-necked). These two species are located on opposite sides of the Solimões-Amazonas than their respective subgroups and are in sympatry in some locations [4]. Hershkovitz further proposed that *A. nancymaae* first emerged south of the Solimões-Amazonas and originated all other red-necked species before migrating north of the river and that the red-necked group is derived from the gray-necked group [4]. The color “gray” is now more commonly spelled “grey” in the literature and, therefore, is used throughout the remainder of this manuscript. 

Subsequent studies challenged Hershkovitz’s interpretation to varying degrees. Two studies using mitochondrial cytochrome c oxidase subunit II gene (mtCOII) included either six specimens across five taxa [5] or 12 specimens across seven taxa [6]; both studies contradicted Hershkovitz by placing *A. nancymaae* with the northern grey-necked group rather than with the red-necked *Aotus azarae boliviensis*. Menezes et al. (2010) [2] analyzed five mitochondrial genes and one Y-linked gene (SRY) for 18 specimens and proposed a phylogenetic reconstruction in which *A. nancymaae* emerged north of the Solimões-Amazonas River and originated the grey-necked species. Then, in 2011, Ruiz-Garcia et al. [3] reported a more extensive study based on seven taxa (that did not include *A. trivirgatus*) using mtCOII in 69 wild *Aotus* specimens, all with a precise geographic origin. These mtDNA results supported the northern grey-necked group, comprised of *A. vociferans*, *A. brumbacki* and *A. griseimembra,* as being more genetically homogeneous than the polyphyletic red-necked group (comprised in their study of *A. nancymaae*, *A. nigriceps*, *A. azarae azarae* and *A. azarae boliviensis*). They further concluded that *A. nancymaae* is the red-necked species most closely related to the grey-necked group and that the ancestors of the grey-necked *A. vociferans* likely represent the original species that led to the formation of the current *Aotus* genus [3]. 

Nuclear genetic markers have also been applied to *Aotus* phylogeny, first as a component of a broader study of primate phylogenetics [7] and later within platyrrhine primates only [8]; both studies placed *A. nancymaae* and *A. azarae* as sister taxa within the red-necked group. However, due to the broader focus of those studies, the number of *Aotus* specimens and representative species was low, and the samples were generally from captive sources such as a zoo or biomedical research colony. Recently, Martins-Junior et al. (2022) [1] analyzed 45 samples from eight taxa using 20 molecular markers (10 nuclear markers previously used by Perelman et al. (2011) [7] and Jameson Kiesling et al. (2015) [8]; 10 mitochondrial markers). Species-level identification of the 45 wild individuals sampled was based on “external pelage traits and the geographical location where the specimens were collected” [1]. Therefore, some individuals were believed to be part of a recent dispersal of wild *Aotus* populations that occurred within the last 1.6 million years (MY), leading to the authors’ rejection of red and grey-necked groups and their proposed taxonomic reclassification into a Northern group of eight taxa (*A. miconax*, *A. nancymaae*, *A. trivirgatus*, *A. vociferans*, *A. l. lemurinus*, *A. griseimembra*, *A. zonalis* and *A. brumbacki*) and a Southern clade of four taxa (*A. nigriceps*, *A. boliviensis*, *A. infulatus* and *A. azarae*).

Species identification based solely on external pelage traits and geographical location can be subject to interpretation, and the morphological similarity of *Aotus* species has led to some misidentifications [2,3]. Accurate species identification is required for biomedical research studies in which owl monkey species vary in their susceptibility to the disease and treatments [9]. For instance, *A. nancymaae* is a frequently used model organism for the study of malaria treatment and vaccine development [10]. Unfortunately, the biomedical utility of owl monkeys has led to a reported resurgence in their illegal traffic and trade into Columbia from other countries [11], displacing them from their native habitats. These issues highlight the need for more accurate molecular methods to differentiate *Aotus* taxa reliably.

Primate-specific *Alu* retrotransposons are a type of SINE (short interspersed element), are approximately 300 bp long and consist of two diverged dimers separated by an A-rich region [12] and ending in a 3′ A-rich tail [13]. Non-autonomous *Alu* elements mobilize via a “copy and paste” mechanism through an RNA intermediate, utilizing the enzymatic machinery of autonomous LINE (L1) elements [14], a process termed “target-primed reverse transcription” (TPRT) [15]. The TPRT integration process involves staggard nicks in the host DNA that produce 5′ and 3′ flanking target site duplications (TSDs) upon insertion. The TPRT mechanism is considered unidirectional such that the absence of insertion is, by default, the ancestral state; conversely, shared insertions with matching TSDs are accepted as being inherited from a common ancestor. These unique attributes have made *Alu* elements well-established diagnostic molecular markers for the study of primate population genetic and phylogenetic relationships [16,17,18,19,20,21,22,23,24,25,26,27,28,29,30,31].

Random nucleotide mutations in *Alu* elements that are subsequently mobilized by TPRT allow for the accumulation of diagnostic substitutions representative of discrete *Alu* repeat subfamilies [32,33,34,35], such that each primate lineage contains a unique group of *Alu* subfamilies [36,37,38,39,40]. The oldest known *Alu* subfamily, *Alu*J, is found in all primates, whereas *Alu*S was more dominant following the separation of Strepsirrhini and Tarsiiformes from Platyrrhini and Catarrhini [39,40] and the younger *Alu*Y subfamily is found only in catarrhines [36]. Therefore, only *Alu*J and *Alu*S subfamilies and their modern derivatives are present in platyrrhines.

The first platyrrhine-specific *Alu* subfamily discovered was given the new name *Alu*T because it was created by a fusion event between *Alu*Sc and *Alu*Sp elements [21]. This platyrrhine-specific *Alu*Ta-lineage includes Ta7, Ta10 and Ta15, with *Alu*Ta15 thought to be limited to the Cebidae family [21]. Other platyrrhine *Alu* subfamilies have been characterized in marmoset [41], squirrel monkey [42], capuchin [43,44] and owl monkeys [43,45]. Some studies utilized *Alu* elements in platyrrhine phylogeny [16,28,29,46,47]; however, an analysis of recently integrated, young *Alu* insertions in owl monkeys has not been conducted. A scaffold-level genome assembly is currently available for one species, *A. nancymaae* [Anan_2.0]. The purpose of this study was to computationally ascertain a dataset of the youngest *Alu* insertions specific to the owl monkey lineage from the [Anan_2.0] genome and to perform locus-specific PCR on a DNA panel of 23 captive *Aotus* specimens to identify *Alu* insertion polymorphisms. The *Alu* datasets reported in this study provide a valuable resource to assist in species identification and to facilitate future studies of *Aotus* phylogeny, population genetics and conservation strategies.

## 2. Materials and Methods

### 2.1. Lineage-Specific Alu Elements

Ascertainment of lineage-specific *Alu* insertions from the *A. nancymaae* genome [Anan_2.0] was performed as described previously [43,45]. Briefly, the scaffold level genome assembly (GCA_000952055.2 Anan_2.0) was obtained from the National Center for Biotechnology Information and analyzed for full-length *Alu* elements with RepeatMasker [48] (RepeatMasker-Open-4.0). Full-length elements were defined as being 267 bp or longer and starting no more than 4 bp from the 5′ start of the *Alu* consensus sequence (RepeatMasker-Open-4.0). Successive implementations of BLAT [49] were performed with the following genomes in the following order: marmoset (*Callithrix jacchus*; calJac3), squirrel monkey (*Saimiri boliviensis*; SaiBol1) and capuchin (*Cebus imitator*; Cebus imitator_1.0). After each BLAT was completed, the output was searched for shared or unique elements by looking for specific gap sizes between the input *Alu* sequences and the target genome. The unique elements were computationally extracted (described at link https://github.com/t-beck; accessed on 24 October 2022). A combination of cross_match (http://www.phrap.org/phredphrapconsed.html; accessed on 6 November 2022) and COSEG (www.repeatmasker.org/COSEGDownload.html; accessed on 6 November 2022) analyses were completed to determine if any of the lineage-specific *Alu* elements extracted from the *Aotus* genome represented new subfamilies [45]. The putative lineage-specific *Alu* elements were aligned with the recently discovered *Saimiri*-specific *Alu* subfamilies, and exact matches were eliminated.

A local installation of RepeatMasker was used to determine the percent divergence of each lineage-specific *Alu* element compared to the consensus sequence. Young elements, defined as being less than 2% diverged, were retained. FASTA files of these young, lineage-specific elements were extracted by a custom Python script (described at link https://github.com/t-beck; accessed on 24 October 2022) and then sorted by subfamily. Representative elements from each *Alu* subfamily were proportionally selected for experimental validation based on the number of subfamily members. If an *Alu* subfamily contained less than five elements, then all elements were selected for experimental validation. BLAT was used to obtain orthologous sequences, including 600 bp of flanking sequences from the marmoset, squirrel monkey and capuchin genomes. BioEdit was used to create multiple sequence alignments for each locus [50].

### 2.2. Oligonucleotide Primer Design

For polymerase chain reactions (PCRs), some of the oligonucleotide primers were designed manually using Primer3 [51] with the following specifications: Tm range = 57–62 °C, Max TmDifference = 2, Max Poly X = 3, Min GC Content = 40 [52]. Other primers were designed by using an in-house primer design pipeline consisting of a series of custom Python scripts in conjunction with Primer3 (available on link https://github.com/t-beck; accessed on 24 October 2022). Following each method, all primers were analyzed for primer specificity and predicted PCR amplicon length using NCBI Primer Blast [53]. The oligonucleotide primers were obtained from Sigma Aldrich (Woodlands, TX, USA).

### 2.3. DNA Samples

DNA samples and their origins are described in Supplementary File S1. Both tissue and DNA samples were obtained from a variety of universities, museums and biomedical research centers. DNA was prepared from tissue samples by proteinase K digestion followed by phenol:chloroform extraction and ethanol precipitation. Extracted DNA was stored in 10 mM Tris/0.1 mM EDTA (TLE) and quantified by an Eppendorf Biophotometer.

DNA samples from 23 owl monkey individuals (12 red-necked; 11 grey-necked) were used for experimental validation by PCR. Biomaterials were obtained for *A. trivirgatus* (*N* = 4), *A. nancymaae* (*N* = 9), *A. vociferans* (*N* = 6), *A. azarae* (*N* = 3) and *Aotus lemurinus griseimembra* (*N* = 1) as well as for outgroups (Supplemental File S1).

### 2.4. PCR Amplification

PCR amplifications were performed in 25 μL reactions that contained 25–50 ng of DNA, 200 nM of each primer, 1.5 mM MgCl_2_, 10× PCR buffer, 0.2 mM deoxyribonucleotide triphosphates and 1 unit of *Taq* DNA polymerase. PCR cycling parameters were: 95 °C for 1 min, 32 cycles of denaturation at 94 °C for 30 s, 30 s at the primer’s respective annealing temperature (typically 57 °C) and extension at 72 °C for 30 s, followed by an extension step at 72 °C for 2 min. Gel electrophoresis was performed on a 2% agarose gel containing 0.2 μg/mL ethidium bromide for 60 min at 175 V. A BioRad ChemiDoc XRS imaging system was used to visualize the DNA fragments by UV fluorescence (Hercules, CA, USA). Gel images were exported for publication as *.tiff files and uploaded to PowerPoint for annotation. PCR results were used to identify *Alu* insertion polymorphisms based on DNA fragment sizes for *Alu* presence/absence following gel electrophoresis. Genotypic data for each allele were recorded in an Excel spreadsheet as follows: (1,1) represented individuals homozygous present for the *Alu* insertion; (0,0) represented individuals homozygous absent for the *Alu* element; and (1,0) represented individuals heterozygous for the *Alu* element.

### 2.5. Sanger Chain Termination DNA Sequencing

Due to the many conflicting reports regarding the phylogenetic relationships among *Aotus* species, all potentially parsimony informative PCR results were subjected to traditional chain-termination DNA sequencing [54]. DNA sequencing was performed as described previously [55]. Briefly, four PCR fragments per locus were gel purified using a Wizard SV gel purification kit (Promega Corporation, Madison, WI, USA, catalog A9282) and 4 µL of the elution were used for chain termination cycle sequencing using BigDye Terminator v3.1. Four separate reactions were conducted for each locus using the original PCR primers (forward vs. reverse) and two internal-*Alu* primers (IntF1: 5′GGTGGCTCACGCCTGTAATC 3′ [55] vs. SIntR1: 5′TCTCGGCTCACCGCAACCTCC 3′ [16]). Following capillary electrophoresis, sequence quality was evaluated using the ABI software Sequence Scanner v2.0. Sequence alignments were constructed in BioEdit [50]. *Alu* elements in the reverse orientation (minus strand) were reverse complemented to be viewed in BioEdit in the forward orientation, and then a consensus sequence for each locus was aligned from the multiple forward and reverse sequences obtained for each locus.

### 2.6. Phylogenetic Analyses

A nexus file was generated from the post-sequencing adjusted PCR data. Unlike the binomial genotypes described previously, single values were used for nexus data: “1” for insertion present, “0” for insertion absent and unknown genotypes were scored as “?”. Because the absence of an *Alu* insertion is the ancestral state of each locus, Dollo’s law of irreversibility was applicable, and all loci were set to Dollo.up. A heuristic search was performed using PAUP* 4.0a169 [56] for ten thousand bootstrap replicates. The output was visualized using FigTree v1.4.4. (http://tree.bio.ed.ac.uk/software/figtree/; accessed on 6 November 2022) and exported as a .jpg file to PowerPoint for annotation.

## 3. Results

### 3.1. Young Lineage-Specific Alu Elements and PCR

**Figure 1:**
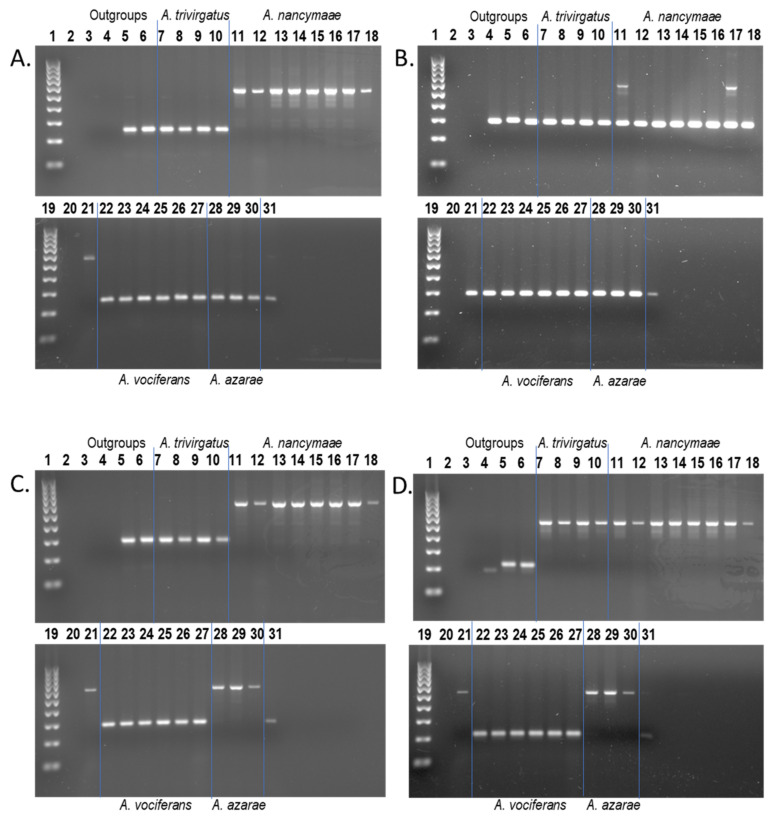
*Aotus Alu* insertion polymorphisms. Lanes: 1, 19—100 bp DNA ladder; 2, 20—blank; 3—TLE (negative control); 4—Human (HeLa); 5—*C. jacchus* (Common marmoset); 6—*Saimiri s. sciureus* (Common squirrel monkey); 7–10—*A. trivirgatus*; 11–18, 21—*A. nancymaae*; 22–27—*A. vociferans*; 28–30—*A. azarae*; 31—*A. lemurinus griseimembra.* (**A**) Locus *Aotus*_1555, *Alu* is homozygous present in all *A. nancymaae* individuals (~567 bp DNA fragment) and absent in all other *Aotus* samples (~245 bp DNA fragment); (**B**) Locus *Aotus*_1706, *Alu* is present in only two *A. nancymaae* individuals (~607 bp DNA fragment lanes 11 and 17), while absent in all other *Aotus* samples (~300 bp DNA fragment) (**C**) Locus *Aotus*_1276, *Alu* is homozygous present in all *A. nancymaae* and *A. azarae* individuals (~600 bp DNA fragment) while absent in all other *Aotus* individuals (~300 bp DNA fragment). (**D**) Locus *Aotus*_1518, *Alu* is homozygous present in all *A. trivirgatus, A. nancymaae* and *A. azarae* individuals (~527 bp DNA fragment) while absent in all *A. vociferans* individuals and the *A. l. griseimembra* sample (~205 bp DNA fragment). Blue lines superimposed on gel images visually separate species groups.

From a total of 658,009 full-length *Alu* insertions [43], we found 12,089 lineage-specific to the owl monkey [Anan_2.0] genome. We identified 1,992 of these elements as young (≤2% sequence divergence from their consensus). Local RepeatMasker [48] (RepeatMasker-Open-4.0) output for these 1,992 loci, along with their genome coordinates and *Alu* subfamily designation, are shown in Supplementary File S1. Wet bench locus-specific PCR analyses were performed on a subset of 332 young insertions, with 305 successful results. An additional nine phylogenetically informative *Aotus*-specific *Alu* insertion polymorphisms taken from Storer et al. (2020) [43] with >2% sequence divergence were re-analyzed for inclusion in this study for a total of 314 elements with successful PCR results. Potential oligonucleotide primers for PCR for an additional 1040 *Alu* elements (52% of the 1992 elements) designed using an in-house primer pipeline were also provided (https://github.com/t-beck; accessed on 24 October 2022) (Supplementary File S1). Each locus name described in the text below is available in Supplementary File S1, along with a matching “query sequence” column containing the genomic coordinates. Data for the 314 young insertions with successful PCR results are available in Supplementary File S1 (worksheets “PCR this study” and “genotypes”) and include representatives from 37 different *Alu* subfamilies [45], summarized in Table S1. PCR results for these 314 *Alu* elements segregated into three basic genotype categories: (1) homozygous present in all *Aotus* samples (*N* = 155) indicating high allele frequency or possible fixation within the genus; (2) homozygous absent in all DNA samples (*N* = 10), indicating very low allele frequency or perhaps unique to the [Anan_2.0] genome; or (3) polymorphic pattern for insertion presence/absence (*N* = 149). These results correspond to a ~50% polymorphism rate within the candidate dataset. These 149 insertion polymorphisms were further divided into four general categories. (1) The target *Alu* insertion was restricted to the reference genome [Anan_2.0] species *A*. *nancymaae* and was homozygous present (h.p.) in all *A. nancymaae* samples (PCR code: A.n. (h.p.)) *N* = 31. An example is shown in Figure 1A. These are potentially useful molecular markers for *A. nancymaae* species identification. (2) The target *Alu* insertion was restricted to *A. nancymaae* and was polymorphic for insertion presence/absence among the nine *A. nancymaae* samples on our DNA panel (PCR code: A.n. (poly)) *N* = 83 (Figure 1B). These *Alu* insertion polymorphisms could be applied to the study of population genetics among *A. nancymaae* wild populations. (3) The target *Alu* insertion was present in both *A. nancymaae* and *A. azarae* samples while being absent in *A. trivirgatus* and *A. vociferans* (PCR code: A.n. and A.a.) *N* = 21 (Figure 1C). These could be employed to study *Aotus* phylogeny. (4) The target *Alu* was homozygous present in *A. nancymaae*, *A. azarae* and *A. trivirgatus* while being absent from *A. vociferans*, *N* = 3 (Figure 1D), suggesting that *A. vociferans* is most basal among the species represented in this study.

**Figure 2:**
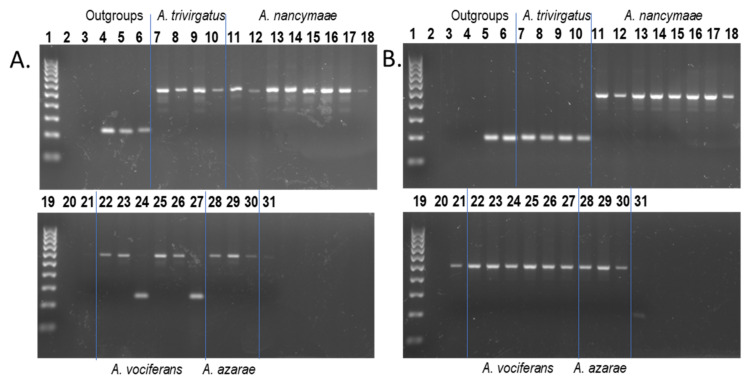
*Aotus Alu* insertion polymorphisms illustrate conflicting topologies. Lanes: 1, 19—100 bp DNA ladder; 2, 20—blank; 3-TLE (negative control); 4—Human (HeLa); 5—*C. jacchus* (Common marmoset); 6—*S. s. sciureus* (Common squirrel monkey); 7–10—*A. trivirgatus*; 11–18, 21—*A. nancymaae;* 22–27—*A. vociferans*; 28–30—*A. azarae*; *31*—*A. lemurinus griseimembra*. (**A**) Locus *Aotus*_1288v2, *Alu* is homozygous present in all *Aotus* samples (~576 bp DNA fragment) with the exception of two *A. vociferans* individuals (~231 bp DNA fragment), lanes 24 and 27. (**B**) Locus *Aotus*_1602, *Alu* is homozygous present in all *A. nancymaae, A. vociferans* and *A. azarae* individuals (~491 bp DNA fragment) while absent in all *A. trivirgatus* individuals and the *A. l. griseimembra* sample (~188 bp DNA fragment). Blue lines superimposed on gel images visually separate species groups.

Further support of this phylogenetic relationship was found in a single locus that was homozygous present in all *Aotus* samples, with the exception of two *A. vociferans* individuals who lacked the insertion (Figure 2A). By contrast, two other *Alu* insertion polymorphisms (*Aotus*_127 and *Aotus*_1602) supported an alternative topology in which the target *Alu* was present in *A. nancymaae*, *A. azarae* and *A. vociferans* while being absent from *A. trivirgatus* (Figure 2B), suggesting that *A. trivirgatus* is more basal than *A. vociferans*, among the taxa available in this study. All potential phylogenetically informative results were subjected to DNA sequencing of PCR amplicons to either confirm the initial genotypes or refute them and provide an alternative explanation.

### 3.2. DNA Sequencing

All potential parsimony informative insertion polymorphisms, as well as any ambiguous PCR fragments or evidence of incomplete lineage sorting (ILS), were subjected to DNA sequencing. These data are shown as Supplemental Figures in Supplementary File S2 and described below. DNA sequencing resulted in the discovery of five novel *Aotus Alu* insertion polymorphisms, two each from *A. azarae* and *A. vociferans* and one from *A. trivirgatus* samples (GenBank Accession Nos. OP549031 to OP549035). Future investigations with expanded species sampling and sample sizes are required to determine the utility of these novel elements. Sequencing also identified traces of non-repeat DNA with the potential for species identification.

#### 3.2.1. Red-Necked Species Group (*A. nancymaae* and *A. azarae*) and Novel *Alu* Discovery

PCR-based genotypes initially identified 23 *Alu* insertion polymorphisms shared by *A. nancymaae* and *A. azarae* to the exclusion of *A. trivirgatus* and *A. vociferans*. DNA sequencing confirmed 19 of these (Supplementary File S2; Figures S1–S19), while in two other cases, the amplicons were too weak to be successfully sequenced (Locus Owl_2LS_040 and Storer_*Aotus*_1124). DNA sequencing of the remaining two candidates revealed the existence of “near parallel insertions”, a different *Alu* insertion from the intended target, but still located between the oligonucleotide primers producing a PCR amplicon of the predicted “*Alu*-present” size. The PCR-based gel image for Locus *Aotus*_404 is shown in Figure S20a and illustrates how the amplicon sizes are similar for *A. nancymaae* and *A. azarae*, while the sequencing alignment (Figure S20b) reveals that *A. azarae* has a different *Alu* about 90 bp 5′ of the target insertion and in the opposite orientation. The second novel *A. azarae Alu* insertion was discovered in Locus *Aotus*_1196. In this case, *A. azarae* sample 85818 was the only one of three *A. azarae* individuals displaying the *Alu* present genotype, and DNA sequencing revealed that this individual has a different *Alu* about 260 bp upstream of the target insertion (Figure S21). These two novel *A. azarae*-derived *Alu* insertions are not parsimony-informative in the current dataset but could be applied to *Aotus* phylogeny, particularly in neighboring populations of *A. boliviensis* and *A. infulatus*. An alternative oligonucleotide primer was designed in each case (Figures S20b and S21) for investigators to potentially genotype these novel insertions separately.

In addition, PCR-based genotypes for Locus *Aotus*_364 indicated that the target *Alu* insertion was shared by *A. nancymaae* and *A. azarae* but also appeared to be homozygous present in *A. vociferans* sample 86218 (Figure S22a). DNA sequencing confirmed that *A. azarae* shares the target insertion with *A. nancymaae* (the 20th confirmed case) but also revealed that *A. vociferans* 86218 has a different *Alu* in the opposite orientation about 115 bp upstream of the target (Figure S22b). This novel *A. vociferans*-derived *Alu* insertion polymorphism is perhaps useful for *Aotus* phylogeny. Therefore, an alternative reverse PCR primer was designed, *Aotus*_364_vociferans-R: GAGTGTTTACTTTGTGCCAAGC, which could be used in conjunction with the existing forward primer to potentially genotype this locus separately (Figure S22b). Depending on the allele frequency distribution of this locus, it could also be useful for species identification.

A second novel *A. vociferans Alu* insertion, was discovered in Locus Owl_2LS_120. PCR-based genotypes indicated that the target *Alu* element was restricted to the reference species *A. nancymaae* except for *A. vociferans* sample 85962, which appeared heterozygous for the insertion. Sequencing alignments revealed that *A. vociferans* 85962 lacks the target insertion and instead has a different *Alu*, a near parallel insertion, about 200 bp upstream (Figure S23) in a genomic region dense with repetitive elements. An alternative reverse primer, Owl_2LS_120-A.voc.-R: CAAGTGAAGTGGAGGTTAGTGT, was designed to use in conjunction with the existing forward primer to potentially genotype this novel *A. vociferans Alu* element separately. These oligonucleotide primers were confirmed to have only one exact match each, in spite of the repeat dense genomic landscape.

In addition to the novel *A. azarae* and *A. vociferans Alu* elements discovered, a fifth novel *Alu* insertion, this one derived from *A. trivirgatus*, was revealed in Locus *Aotus*_68. PCR-based genotypes indicated that the target *Alu* insertion was polymorphic among *A. nancymaae* individuals and homozygous present in other *Aotus* samples, while *A. trivirgatus* individuals displayed an additional larger amplicon fragment suggesting that they might harbor an additional *Alu* insertion that is not present in other *Aotus* species (Figure S24a). Sequence alignments confirmed that *A. trivirgatus* has a second *Alu* insertion located about 113 bp 5′ from the target insertion. An alternative forward PCR primer (*Aotus*_68_A.triv-F: ACTTGCTTTGTCATGGCTTCATC) was designed that could be used in conjunction with the existing reverse primer to potentially genotype this newly discovered *A. trivirgatus* derived *Alu* element separately from the [Anan_2.0] ascertained target insertion (Figure S24b).

#### 3.2.2. Grey-Necked Species Group (*A. vociferans* and *A. trivirgatus*)

*Alu* insertion polymorphisms that grouped the two red-necked species, *A. nancymaae* and *A. azarae*, were generally absent from both *A. trivirgatus* and *A. vociferans* individuals. However, as shown in Figure 1D and Figure 2A, there were other *Alu* insertions that grouped *A. trivirgatus*, a traditional grey-necked or Northern species, together with *A. nancymaae* and *A. azarae* to the general exclusion of *A. vociferans*. DNA sequencing confirmed this genotype pattern for Locus Owl_2LS_078 (Figure S25) and Locus *Aotus*_1518 (Figure S26). In addition, PCR-based genotypes for Locus *Aotus*_1546 indicated that the target *Alu* insertion was polymorphic among *A. nancymaae* individuals, homozygous present in *A. trivirgatus*, *A. azarae* and the *A. lemurinus griseimembra* sample, while completely absent from all six *A. vociferans* individuals (Figure S27a). DNA sequencing confirmed these genotypes (Figure S27b), placing *A. vociferans* as the most basal among the *Aotus* species in this study; further, based on the heterozygous genotypes among some *A. nancymaae* individuals at this locus, these results suggest that *A. nancymaae* is more closely related to the grey-necked species than is *A. azarae*. Alternatively, this could be evidence of ILS or a consequence of captivity, although pedigree records indicate that the two heterozygous *A. nancymaae* individuals (86334 and 85974) are unrelated males.

For Locus *Aotus*_1288V2 (Figure 2A), the target *Alu* element is homozygous present in all *Aotus* subjects except for two *A. vociferans* individuals, 86100 and 86230, which displayed precise pre-integration sites with sequence traces spanning the target region, confirming the genotypes (Figure S28). This locus also places *A. vociferans* as the most basal among the *Aotus* species in this study. However, it should be noted that *A. vociferans* individuals 86100 and 86230 are known to be full siblings (sister and brother, respectively; pedigree records provided by the source institution).

Locus Owl_2LS_042 was originally suspected of having a second *Alu* element, in addition to the target insertion, that was shared by *A. trivirgatus* and *A. vociferans* to the exclusion of *A. nancymaae* and *A. azarae* (Figure S29a); if confirmed, that would represent a novel *Alu* element that grouped *A. trivirgatus* and *A. vociferans* together. However, DNA sequencing revealed that this shared larger PCR amplicon is not an *Alu* element, but rather a ~224 bp insertion of extra non-repeat sequence about 80 bp 5′ of the reverse primer and about 100 bp downstream of the target reference *Alu* insertion, which was confirmed to be shared by both *A. trivirgatus* and *A. vociferans* (Figure S29b). The target *Alu* is likely homozygous present within *Aotus*. This extra shared sequence in both *A. trivirgatus* and *A. vociferans* was most likely inherited from a common ancestor, as it seems unlikely that such a relatively large insertion occurred independently in each lineage. Thus, it represents an informative serendipitous finding even though not a homoplasy-free structural variant (GenBank Accession No. OP549036).

Although the above results placed *A. vociferans* as the most basal *Aotus* species, we also recovered three other *Alu* insertions (Figure 2B) that grouped *A. vociferans* together with *A. nancymaae* and *A. azarae* to the general exclusion of *A. trivirgatus*, suggesting that *A. trivirgatus* is the most basal *Aotus* species. Gel images for Locus *Aotus*_127 (Figure S30a) and Locus *Aotus*_1602 (Figure 2B) illustrate this genotype distribution. DNA sequencing results confirmed these insertion presence/absence patterns for both *Aotus*_127 and *Aotus*_1602 (Figures S30b and S31, respectively). PCR-based genotypes for a third *Alu* element, Locus Storer_*Aotus*_622 indicated that the target *Alu* insertion was homozygous present in *A. nancymaae* and *A. azarae*, polymorphic among *A. vociferans* samples while being absent in all four *A. trivirgatus* individuals. DNA sequencing confirmed that both *A. vociferans* MVZ-155159 and *A. azarae* 85818 share the target *Alu* insertion with *A. nancymaae*, while both *A. trivirgatus* crl1556 and *A. vociferans* 86230 have a clean pre-integration site spanning the target region, thus confirming the genotypes (Figure S32).

The remaining seven *Alu* insertion polymorphisms did not meet any of the general classifications described above and instead displayed erratic insertion patterns indicative of ILS or artifactual origin. PCR-based genotypes for Locus Owl_2LS_018 indicated that the target *Alu* insertion was polymorphic among *A. nancymaae* and *A. vociferans* individuals, homozygous present in *A. trivirgatus* while being absent in *A. azarae*. DNA sequencing confirmed this strange genotype distribution (Figure S33), indicative of extensive ILS. PCR-based genotypes for Locus *Aotus*_1582 indicated that the target *Alu* insertion was homozygous absent from all *Aotus* individuals except for *A. vociferans* MVZ-155159 and 86218 (Figure S34a), perhaps suggestive of a de novo insertion unique to *A. vociferans.* However, DNA sequencing confirmed that both *A. vociferans* individuals share the target *Alu* insertion (Figure S34b), unveiling an interesting case of ILS, especially given that no *A. nancymaae* samples in our DNA panel possess the reference target insertion. Another suspected case of extensive ILS occurred for Locus Owl_2LS_046 in which PCR-based genotypes indicated that the target *Alu* insertion was polymorphic among *A. nancymaae*, absent from *A. azarae* and *A. vociferans* while being homozygous present in all four *A. trivirgatus* individuals (Figure S35a), giving the appearance that *A. trivirgatus* is most related to *A. nancymaae.* However, DNA sequencing revealed that the *A. trivirgatus* amplicon does not contain an *Alu* element but rather is comprised of 306 bp of non-*Alu* sequence, some of which is an L1, and therefore the target *Alu* insertion is restricted to *A. nancymaae* (Figure S35b).

Locus *Aotus*_1153 displayed a PCR pattern in which only the *A. vociferans* samples lacked what was suspected to be a second *Alu* element (a larger amplicon) in addition to the intended target insertion (Figure S36a). However, DNA sequencing revealed that this larger amplicon observed in some *A. trivirgatus*, *A. nancymaae* and *A. azarae* samples was not an *Alu* element but rather an uninformative artifact sequence (Figure S36b). Similarly, the target *Alu* insertion in Locus *Aotus*_1422 appeared to be restricted to *A. nancymaae* except for a possible truncated form observed only among *A. vociferans*; however, DNA sequencing revealed *A. vociferans* contained only a non-repetitive sequence in this region (Figure S37). Lastly, two *Alu* insertion polymorphisms seemed homozygous present in all *Aotus* samples except for a single heterozygous individual, indicative of ILS. DNA sequencing confirmed the heterozygous genotype in both cases, Locus Owl_2LS_110 and Locus *Aotus*_1765 (Figures S38 and S39, respectively). Post-sequencing adjusted genotypes and comments are available in Supplementary File S1. Sequence alignments constructed in BioEdit [50] are available as *.GB files in Supplementary File S3.

### 3.3. Maximum Parsimony

**Figure 3:**
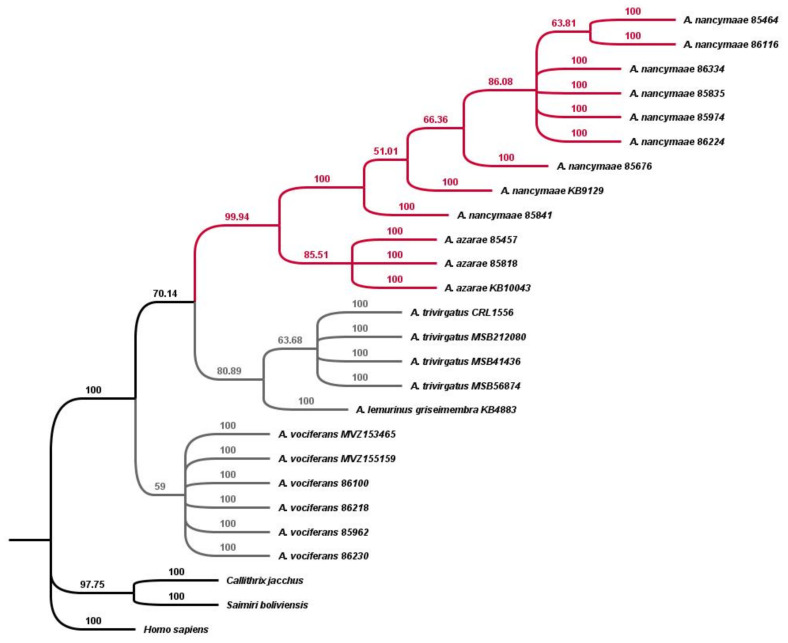
The most parsimonious tree generated from analysis of 314 *Alu* insertions from the *A. nancymaae* genome [Anan_2.0]. The amplification patterns and post-sequencing adjustments of the *Alu* insertions were used to construct a Dollo parsimony tree of phylogenetic relationships with human (*Homo sapiens*), marmoset (*C. jacchus*) and squirrel monkey (*S. boliviensis*) as outgroups using the PAUP* (version 4.0a169) program [56]. Numbers above branches are bootstrap values. Red-necked species *A. nancymaae* and *A. azarae* group together (red nodes), while *A. vociferans* is most basal among grey-necked species (grey nodes) but with low bootstrap support. At the top of the tree, *A. nancymaae* 85464 and 86116 form a separate branch within the *A. nancymaae* clade, and they are known to be mother/daughter, respectively. Consistency index (CI): 0.608; homoplasy index (HI): 0.392; retention index (RI): 0.923. The relatively high homoplasy index results from ILS among the grey-necked group.

Post-sequencing adjusted genotype data were used to construct a data matrix of 26 taxa (23 *Aotus* samples and 3 outgroups) and 314 characters to perform a maximum parsimony heuristic search. Of the 314 *Alu* insertion loci, 165 were constant, and another 114 were restricted to *A. nancymaae,* leaving 35 potentially parsimony informative among *Aotus* species (Figure 3).

The debate over the phylogenetic relationships among *Aotus* species has generally centered around the placement of *A. nancymaae*, either as a red-necked species within the southern group or as a member of a northern geographical group more closely related to the traditional grey-neck species. The topology of this tree strongly supports *A. nancymaae* and *A. azarae* as sister taxa with nearly 100% bootstrap support, as well as the monophyly of both *A. trivirgatus* and *A. vociferans* as separate taxa. *A. vociferans* is basal on the tree with only 59% bootstrap support, while three *Alu* elements support an alternative topology, placing *A. trivirgatus* as the most basal. The tree generally supports the division of traditional red and grey-necked species across these five taxa.

## 4. Discussion

The *Alu* data presented in this study are in general agreement with the research of Hershkovitz [4] and studies using other nuclear genetic markers [7,8] by providing support for the separation of *Aotus* taxa along traditional red and grey-necked groups. Among the studies that utilized mtDNA markers, this *Alu* study is more in agreement with Ruiz-Garcia et al. (2011) [3], who concluded that *A. nancymaae* is the red-necked species most closely related to the grey-necked group and that the ancestors of the grey-necked *A. vociferans* likely represent the original species that led to the formation of the current *Aotus* genus. The *Alu* evidence in this study firmly places *A. nancymaae* together with *A. azarae*, in contradiction to other studies [1,2] that placed *A. nancymaae* within the northern species group alongside *A. vociferans*.

However, a recognized limitation of the current *Alu* study is that all the specimens were primarily from captive breeding colonies, a zoo or from museum archives and therefore are unlikely to harbor widespread geographic origins that would accurately simulate the modern dynamics of wild populations [1]. Further, this study only included five *Aotus* taxa, and only four of them contain multiple specimens per species, some of which were obtained from at least two different independent sources, while others are known relatives based on pedigree records. Moreover, this study did not include representative samples from several recognized species (including *A. infulatus* or *A. nigriceps,* which are geographic neighbors to *A. azarae*), nor did it include *A. miconax*, *A. zonalis* or *A. brumbacki* from northern species neighboring *A. vociferans* [1]. Therefore, the resolution of *Aotus* phylogeny was not a reasonable expectation of this study. However, the observation that the *Alu* genotypes in this study are often segregated by species group, more so than by sample source, indicates that this *Alu* dataset could be broadly applied to wild specimens to track population genetics and infer phylogenetic relationships.

The discrepancy in the placement of *A. nancymaae* observed between nuclear and mtDNA-based phylogenies implies that the maternally derived mitochondrial genome is ancestrally distant from that of the nuclear genome, as has been reported, for instance, in baboons [57]. This can be caused by male migration and introgression into more stable female populations. An alternative explanation provided by Martins-Junior et al. (2022) [1] suggests that successive periods of rapid divergence among owl monkeys allowed the faster-evolving mitochondrial genome to keep pace, compared to the slower rate of nuclear markers. Another explanation discussed is possible ancestral hybridization. Although there is evidence of inter-species *Aotus* hybridization in captivity [58], there is no evidence of hybrids among the samples in the current study and very little evidence of successful inter-species hybridization in wild *Aotus* populations in sympatric regions [1]. The *Alu* data in this study, however, confirmed the existence of extensive ILS among the *Aotus* taxa investigated, warranting further investigation. Implementation of identical-by-descent retrotransposons could help resolve some of these complex issues.

The number of *Aotus* lineage-specific *Alu* insertions (~12,000) is considerably higher than the ~5000 reported for humans [59] and somewhat higher than the ~9000 reported for capuchin [43,44], a fellow member of the related Cebidae lineage. A high rate of *Alu* retrotransposition increases the likelihood of detecting “near parallel insertions”, which are independent retrotransposons within the span of the same PCR amplicon. This situation can complicate PCR interpretation unless resolved by sequencing, as they were in this study. However, a high retrotransposition rate also provides an opportunity for serendipitous findings of novel *Alu* elements from taxa currently lacking whole genome sequence assemblies. The five novel *Alu* elements reported in this study were not ascertained from the *A. nancymaae* reference genome [Anan_2.0] and are, therefore, not subject to the same level of directional ascertainment bias. Until more genomes for other *Aotus* species become available, the five novel *Alu* elements reported here provide alternate evolutionary perspectives to [Anan_2.0].

*Alu* insertion polymorphisms were identified from 37 different platyrrhine *Alu* subfamilies, all of which were *Alu*Ta15 derived [45]. The highest polymorphism rate occurred among subfamilies discovered in the owl monkey genome [Anan_2.0], meaning that those *Alu* subfamilies do not have detectable members in the platyrrhine genomes characterized prior to *Aotus*, those being marmoset, [calJac3], squirrel monkey, [SaiBol1] and capuchin, [Cebus imitator_1.0]. This strategic approach facilitates the discovery of the youngest *Alu* subfamilies with the ongoing proliferation of progeny elements.

## 5. Conclusions

This study provides a comprehensive dataset of *Aotus* lineage-specific *Alu* elements and demonstrates their utility as phylogenetically diagnostic insertion polymorphisms. It shows that multiple *Alu* subfamilies have evidence of recent mobilization within owl monkey genomes. *Alu* elements restricted to *A. nancymaae*, as well as the discovery of novel *Alu* elements from three additional owl monkey taxa, will assist in species identification. Application of this dataset of *Alu* insertion polymorphisms to a large sample size of wild populations could potentially resolve some of the complex issues influencing the current taxonomy. The *Alu* insertions reported in this study will provide researchers with an additional resource for the study of *Aotus* phylogenetic and population genetic relationships, bringing more insight into the elusive “night monkeys” to further facilitate conservation strategies.

## Data Availability

The algorithms used in this study are available on GitHub (https://github.com/t-beck; accessed on 24 October 2022). The Supplementary Data Files are available on the online version of this paper and through the Batzer Lab website under publications, https://biosci-batzerlab.biology.lsu.edu/; accessed on 6 November 2022. Novel DNA sequences have been deposited in GenBank under Accession Nos. OP549031 to OP549036).

## Supplementary Materials

The following supporting information can be downloaded at https://www.mdpi.com/article/10.3390/genes13112069/s1: Supplementary File S1 is an Excel file containing the RepeatMasker output for the *Alu* elements ascertained from [Anan_2.0] and separate worksheets for Table S1, PCR this study, DNA samples and Genotypes. Supplementary File S2 is a PDF containing the supplemental figures, Figures S1 to S39, including DNA sequencing alignments and the associated gel image if required for illustration. Supplementary File S3 is a compressed zipped folder containing the DNA sequencing alignments as *.GB file format.
