# Supplementary material for: Owl Monkey Alu Insertion Polymorphisms and Aotus Phylogenetics
Source: Genes (Basel). 2022 Nov 8;13(11):2069. doi: 10.3390/genes13112069 (PMC9691001; doi:10.3390/genes13112069)
Supplement: Supplementary file 1 [file genes-13-02069-s001.zip › Supplementary File S2.pdf]

Aotus Alu Supplementary Figures
Table of Contents

Figure S1: *A. nancymaae* and *A. azarae* shared: Locus Owl_2LS_049 sequence alignment
Figure S2: *A. nancymaae* and *A. azarae* shared: Locus Owl_2LS_056 sequence alignment
Figure S3: *A. nancymaae* and *A. azarae* shared: Locus Owl_2LS_091 sequence alignment
Figure S4a: *A. nancymaae* and *A. azarae* shared; one *A. trivirgatus*: Locus Owl_2LS_095 gel image
Figure S4b: *A. nancymaae* and *A. azarae* shared; one *A. trivirgatus*: Locus Owl_2LS_095 sequence alignment
Figure S5: *A. nancymaae* and *A. azarae* shared: Locus Owl_2LS_111 sequence alignment
Figure S6: *A. nancymaae* and *A. azarae* shared: Locus *Aotus*_359 sequence alignment
Figure S7: *A. nancymaae* and *A. azarae* shared: Locus *Aotus*_420 sequence alignment
Figure S8: *A. nancymaae* and *A. azarae* shared: Locus *Aotus*_506 sequence alignment
Figure S9: *A. nancymaae* and *A. azarae* shared: Locus *Aotus*_592 sequence alignment
Figure S10: *A. nancymaae* and *A. azarae* shared: Locus *Aotus*_1202 sequence alignment
Figure S11: *A. nancymaae* and *A. azarae* shared: Locus *Aotus*_1203 sequence alignment
Figure S12: *A. nancymaae* and *A. azarae* shared: Locus *Aotus*_1276 sequence alignment
Figure S13: *A. nancymaae* and *A. azarae* shared: Locus *Aotus*_1507 sequence alignment
Figure S14: *A. nancymaae* and *A. azarae* shared: Locus *Aotus*_1528 sequence alignment
Figure S15: *A. nancymaae* and *A. azarae* shared: Locus *Aotus*_1534 sequence alignment
Figure S16: *A. nancymaae* and *A. azarae* shared: Locus *Aotus*_1559V2 sequence alignment
Figure S17: *A. nancymaae* and *A. azarae* shared: Storer, et al. 2020_*Aotus*_827 sequence alignment
Figure S18: *A. nancymaae* and *A. azarae* shared: Storer, et al. 2020_*Aotus*_1120 sequence alignment
Figure S19: *A. nancymaae* and *A. azarae* shared: Storer, et al. 2020_*Aotus*_6430 sequence alignment
Figure S20a: *A. azarae* novel *Alu*: near parallel insertion: Locus *Aotus*_404 gel image
Figure S20b: *A. azarae* novel *Alu*: near parallel insertion: Locus *Aotus*_404 sequence alignment
Figure S21: *A. azarae* novel *Alu*: near parallel insertion: Locus *Aotus*_1196 sequence alignment
Figure S22a: *A. nancymaae* and *A. azarae* shared; *A. vociferans* novel *Alu*: near parallel insertion: Locus *Aotus*_364 gel image
Figure S22b: *A. nancymaae* and *A. azarae* shared; *A. vociferans* novel *Alu*: near parallel insertion: Locus *Aotus*_364 alignment
Figure S23: A. vociferans novel *Alu*: near parallel insertion: Locus Owl_2LS_120 sequence alignment
Figure S24a: *A. trivirgatus* novel *Alu*: Locus *Aotus*_68 gel image
Figure S24b: *A. trivirgatus* novel *Alu*: Locus *Aotus*_68 sequence alignment
Figure S25: Supports *A. vociferans* as basal: Locus Owl_2LS_078 sequence alignment
Figure S26: Supports *A. vociferans* as basal: Locus *Aotus*_1518 sequence alignment

Figure S27a: Supports *A. vociferans* as basal: Locus *Aotus*_1546 gel image
Figure S27b: Supports *A. vociferans* as basal: Locus *Aotus*_1546 sequence alignment
Figure S28: Supports *A. vociferans* as basal: Locus *Aotus*_1288V2 sequence alignment
Figure S29a: *A. vociferans* and *A. trivirgatus*; share extra sequence; Locus Owl_2LS_042 gel image
Figure S29b: *A. vociferans* and *A. trivirgatus*; share extra sequence; Locus Owl_2LS_042 sequence alignment
Figure S30a Supports *A. trivirgatus* as basal: Locus *Aotus*_127 gel image
Figure S30b Supports *A. trivirgatus* as basal: Locus *Aotus*_127 sequence alignment
Figure S31 Supports *A. trivirgatus* as basal: Locus *Aotus*_1602 sequence alignment
Figure S32 Supports *A. trivirgatus* as basal: Storer, et al. 2020_*Aotus*_622 sequence alignment
Figure S33 Evidence of incomplete lineage sorting (ILS) Locus Owl_2LS_018 sequence alignment
Figure S34a Evidence of incomplete lineage sorting (ILS) Locus *Aotus*_1582 gel image
Figure S34b Evidence of incomplete lineage sorting (ILS) Locus *Aotus*_1582 sequence alignment
Figure S35a Suspected incomplete lineage sorting (ILS); non-repeat sequence; Locus Owl_2LS_046 gel image
Figure S35b Suspected incomplete lineage sorting (ILS); non-repeat sequence; Locus Owl_2LS_046 sequence alignment
Figure S36a Suspected 2nd *Aotus Alu* absent from *A. vociferans*; non-repeat sequence Locus *Aotus*_1153 gel image
Figure S36b Suspected 2nd *Aotus Alu* absent from *A. vociferans*; non-repeat sequence Locus *Aotus*_1153 sequence alignment
Figure S37 Suspected *A. vociferans Alu*; non-repeat sequence; Locus *Aotus*_1422 sequence alignment
Figure S38 Confirmed heterozygosity: Locus Owl_2LS_110 sequence alignment
Figure S39 Confirmed heterozygosity: Locus *Aotus*_1765 sequence alignment

Abbreviations used in alignment figures:

F: Sequence trace using the forward PCR primer
R: Sequence trace using the reverse PCR primer
IntF: Sequence trace using the Internal-*Alu* forward primer 5’ GGTGGCTCACGCCTGTAATC 3’ (Konkel et al. 2015)
SIntR: Sequence trace using the Internal-*Alu* reverse primer 5’ TCTCGGCTCACCGCAACCTCC 3’ (Baker et al. 2018)
-rc: Sequence trace reverse compliment to align properly
-E: Empty site PCR amplicon (*Alu* absent fragment) was sequenced

```
                                    510       520       530       540       550       560       570       580       590       600
                                ....|....|....|....|....|....|....|....|....|....|....|....|....|....|....|....|....|....|....|....|
Owl_2LS_049 [Anan_2.0]          CTTTCCCGTttagatcaggaacaaggcaagaatgtctGTTCTctccacttctattcaacattgtactagaggTTCTAGCCATGGaggttagaaaaaagaa
LR-49-85818-F                   ~~~~~~~~~~~~~~~~~~~~~~~~~~~~~~~~~~~~~~~~~~~~~~~CTTCTATTCAACATTGTACTAGAGGTTCTAGCCATGGAGGTTAGAAAAAAGAA
LR-49-85818-SIntR-rc            CTTTCCCGTTTAGATCAGGAACAAGGCAAGAATGTCTGTTCTCTCCACTTCTATTCAACATTGTACTAGAGGTTCTAGCCATGGAGGTTAGAAAAAAGAA
LR-49-85818-IntF                ~~~~~~~~~~~~~~~~~~~~~~~~~~~~~~~~~~~~~~~~~~~~~~~~~~~~~~~~~~~~~~~~~~~~~~~~~~~~~~~~~~~~~~~~~~~~~~~~~~~~
LR-49-85818-R-rc                ~~~~~~~~~~~~~~~~~~~~~~~~~~~~~~~~~~~~~~~~~~~~~~~~~~~~~~~~~~~~~~~~~~~~~~~~~~~~~~~~~~~~~~~~~~~~~~~~~~~~

                                    610       620       630       640       650       660       670       680       690       700
                                ....|....|....|....|....|....|....|....|....|....|....|....|....|....|....|....|....|....|....|....|
Owl_2LS_049 [Anan_2.0]          aggccgggcgcggtggctcaagcctgtaatcccagcactttgggaggccgaggcgggtggatcacgaggtcaagagatcgagaccatcctggtcaaaatg
LR-49-85818-F                   AGGCCGGGCGCGGTGGCTCAAGCCTATAATCCCAGCACTTTGGGAGGCCGAGGCGGGTGGATCACGAGGTCAAGAGATCGAGACCATCCTGGTCAAAATG
LR-49-85818-SIntR-rc            AGGCCGGGCGCGGTGGCTCAAGCCTATAATCCCAGCACTTTGGGAGGCCGAGGCGGGTGGATCACGAGGTCAAGAGATCGAGACCATCCTGGTCAAAATG
LR-49-85818-IntF                ~~~~~~~~~~~~~~~~~~~~~~~~~~~~~~~~~~~~~~~~~~~~~~~~~~~~~~~~~~~~~~~~~~~~~~~~~~~~~~~~~GACCATCCTGGTCAAA~TG
LR-49-85818-R-rc                ~~~~~~~~~~~~~~~~~~~~~~~~~~~~~~~~~~~~~~~~~~~~~~~~~~~~~~~~~~~~~~~~~~~~~~~~~~~~~~~~~~~~~~~~~~~~~~~~~~~~

                                    710       720       730       740       750       760       770       780       790       800
                                ....|....|....|....|....|....|....|....|....|....|....|....|....|....|....|....|....|....|....|....|
Owl_2LS_049 [Anan_2.0]          gtgaaaccccgtctctactaatagaaaaaattagctgggcatggtggcgcgtgcctgtaatcccagctactcaggatgctgaggcaggagaattgcctga
LR-49-85818-F                   GTGAAACCCCGTCTCTACTAATAGAAAAAATTAGCTGGGCATGGTGGCGCGTGCCTGTAATCCCAGCTACTCAGGAGGCTGAGGCAGGAGAATTGCCTGA
LR-49-85818-SIntR-rc            GTGAAACCCCGTCTCTACTAATAGAAAAAATTAGCTGGGCATGGTGGCGCGTGCCTGTAATCCCAGCTACTCAGGAGG
LR-49-85818-IntF                GTGAAACCCCGTCTCTACTAATAGAAAAAATTAGCTGGGCATGGTGGCGCGTGCCTGTAATCCCAGCTACTCAGGAGGCTGAGGCAGGAGAATTGCCTGA
LR-49-85818-R-rc                ~~~~~~~~~~~~~~~~~~~~~~~~~~~~~~~~~~~~~~~~~~~~~~~~~~~~~~~~~~~~~~~~~~~~~~~~~~~~~~~~~~~~~~~~~~~~~~~~~~~~

                                    810       820       830       840       850       860       870       880       890       900
                                ....|....|....|....|....|....|....|....|....|....|....|....|....|....|....|....|....|....|....|....|
Owl_2LS_049 [Anan_2.0]          acccaggaggcggaggttgcggtgagccgagatcgcgccattgcactccagcctgggtaacaagagcgaaactccgtctcaaaaaaaaaaaaaaaaaaaa
LR-49-85818-F                   ACCCAGGAGGCGGAGGTTGCGGTGAGCCGAGATCGCGCCATTGCACTCCAGCCTGGGTAACAAGAGCGAAACTCCGTCTCAAAAAAAAAAAAAAAAAAAA
LR-49-85818-SIntR-rc
LR-49-85818-IntF                ACCCAGGAGGCGGAGGTTGCGGTGAGCCGAGATCGCGCCATTGCACTCCAGCCTGGGTAACAAGAGCGAAACTCCGTCTCAAAAAAAAAAAAAAAAAAAA
LR-49-85818-R-rc                ~~~~~~~~~~~~~~~~~~~~~~~~~~~~~~~~~~~~~~~~~~~~~~~~~~~~~~~~~~~~~~~~~~~~~~~~~~~~~~~~~~~~~~~~~~~AAAAAAAAA

                                    910       920       930       940       950       960       970       980       990      1000
                                ....|....|....|....|....|....|....|....|....|....|....|....|....|....|....|....|....|....|....|....|
Owl_2LS_049 [Anan_2.0]          gaaagaaagaaagaaaagaagaaaggaatgaagggagtgagggagggaggaaggaaggagaagaatgaacaaacaaacaagacaagacatccagattgga
LR-49-85818-F                   AAAAAAAAAAAA
LR-49-85818-SIntR-rc
LR-49-85818-IntF                AAAAAAAAAAAAA
LR-49-85818-R-rc                AAAAGAAAGAAAGAAAAGAAGAAAGGAATGAAGGGAGTGAGGGAGGGAGGAAGGAAGGAGAAGAATGAACAAACAAACAAGACAAGACATCCAGA
```

Figure S1. Sequence alignment for *Alu* locus Owl_2LS_049_018487317.1:279352-280858 (LR-49). PCR-based genotypes indicate the target *Alu* insertion is homozygous present among *A. nancymaae* and *A. azarae* individuals, while being absent from other *Aotus* species. The *Aotus* reference genome [Anan_2.0] is shown at the top followed by *A. azarae* 85818 who shares the target *Alu* insertion starting at position 602 (grey highlight), with matching flanking sequence and TSDs in yellow highlight.

```
                                810       820       830       840       850       860       870       880       890       900
                        ....|....|....|....|....|....|....|....|....|....|....|....|....|....|....|....|....|....|....|....|
Owl_2LS_056 [Anan_2.0]  TaccaggagttaaaaaaaaaaatgaaacaaaacaaaaataactactATCAGGATTGTTATCCTAaaggcaatttttaatttttatattttacttaaaccc
LR-56-85818-F           ~~~~~~~~~~~~~~~~~~~~~~~~~~~~~~~~~~~~~~~~~~~~~~~~~~~~~~~~~~ATCCTAAAGGCAATTTTTAATTTTTATATTTTACTTAAACCC
LR-56-85818-SIntR-rc    TACCAGGAGTTAAAAAAAAAAATGAAACAAAACAAAAATAACTACTATCAGGATTGTTATCCTAAAGGCAATTTTTAATTTTTATATTTTACTTAAACCC
LR-56-85818-IntF        ~~~~~~~~~~~~~~~~~~~~~~~~~~~~~~~~~~~~~~~~~~~~~~~~~~~~~~~~~~~~~~~~~~~~~~~~~~~~~~~~~~~~~~~~~~~~~~~~~~~~
LR-56-85818-R-rc        ~~~~~~~~~~~~~~~~~~~~~~~~~~~~~~~~~~~~~~~~~~~~~~~~~~~~~~~~~~~~~~~~~~~~~~~~~~~~~~~~~~~~~~~~~~~~~~~~~~~~

                                910       920       930       940       950       960       970       980       990      1000
                        ....|....|....|....|....|....|....|....|....|....|....|....|....|....|....|....|....|....|....|....|
Owl_2LS_056 [Anan_2.0]  ttaaatattttttttaaagagtgactctggccgggcgcggtggctcaagcctgtaatcccagcactttgggaggccgaggcgggtggatcacgagtcaag
LR-56-85818-F           TTAAATATTTTTTTTAAAGACTGACTCTGGCCGGGCGCGGTGGCTCAAGCCTGTAATCCCAGCACTTTGGGAGGCCGAGGCGGGTGGATCACGAGTCAAG
LR-56-85818-SIntR-rc    TTAAATATTTTTTTTAAAGACTGACTCTGGCCGGGCGCGGTGGCTCAAGCCTGTAATCCCAGCACTTTGGGAGGCCGAGGCGGGTGGATCACGAGTCAAG
LR-56-85818-IntF        ~~~~~~~~~~~~~~~~~~~~~~~~~~~~~~~~~~~~~~~~~~~~~~~~~~~~~~~~~~~~~~~~~~~~~~~~~~~~~~~~~~~~~~~~~~~~GAGTCAAG
LR-56-85818-R-rc        ~~~~~~~~~~~~~~~~~~~~~~~~~~~~~~~~~~~~~~~~~~~~~~~~~~~~~~~~~~~~~~~~~~~~~~~~~~~~~~~~~~~~~~~~~~~~~~~~~~~~

                               1010      1020      1030      1040      1050      1060      1070      1080      1090      1100
                        ....|....|....|....|....|....|....|....|....|....|....|....|....|....|....|....|....|....|....|....|
Owl_2LS_056 [Anan_2.0]  agatcgagaccatcctggtcaacatggtgaaaccccgtctctactaaaaatacaaaaaattagctgggcacagtggtgcgtgcctgtaatcccagctact
LR-56-85818-F           AGATCGAGACCATCCTGGTCAACATGGTGAAACCCCGTCTCTACTAAAAATACAAAAAATTANCTGGGCACAGTGGTGCGTGCCTGTAATCCCAGCTACT
LR-56-85818-SIntR-rc    AGATCGAGACCATCCTGGTCAACATGGTGAAACCCCGTCTCTACTAAAAATACAAAAAATTAGCTGGGCACAGTGGTGCGTGCCTGTAATCCCAGCTACT
LR-56-85818-IntF        AGATCGAGACCATCCTGGTCAACATGGTGAAACCCCGTCTCTACTAAAAATACAAAAAATTAGCTGGGCACAGTGGTGCGTGCCTGTAATCCCAGCTACT
LR-56-85818-R-rc        ~~~~~~~~~~~~~~~~~~~~~~~~~~~~~~~~~~~~~~~~~~~~~~~~~~~~~~~~~~~~~~~~~~~~~~~~~~~~~~~~~~~~~~~~~~~~~~~~~~~~

                               1110      1120      1130      1140      1150      1160      1170      1180      1190      1200
                        ....|....|....|....|....|....|....|....|....|....|....|....|....|....|....|....|....|....|....|....|
Owl_2LS_056 [Anan_2.0]  caggaggctgaggcaggagaattgcctgaacccaggaggcggaggttgcggtgagccgagatcgcgccattgcactccagcctgggtaatgagcgaaact
LR-56-85818-F           CAGGAGGCTGAGGCAGGAAATTTGCCTGAACCCAGGAGGCGGAGGTTGCGGTGAGCCGAGATCGCGCCATTGCACTCCACCCTGGGTAATGAGCGAAACT
LR-56-85818-SIntR-rc    CAGGAGG
LR-56-85818-IntF        CAGGAGGCTGAGGCAGGAGATTTGCCTGAACCCAGGAGGCGGAGGTTGCGGTGAGCCGAGATCGCGCCATTGCACTCCAGCCTGGGTAATGAGCGAAACT
LR-56-85818-R-rc        ~~~~~~~~~~~~~~~~~~~~~~~~~~~~~~~~~~~~~~~~~~~~~~~~~~~~~~~~~~~~~~~~~~~~~~~~~~~~~~~~~~~~~~~~~~~~~~~~~~~~

                               1210      1220      1230      1240      1250      1260      1270      1280      1290      1300
                        ....|....|....|....|....|....|....|....|....|....|....|....|....|....|....|....|....|....|....|....|
Owl_2LS_056 [Anan_2.0]  ccgtctcaaaaaaaaaaaaaaaaaaaaaagagtgactcTTAGATTCTACTTAGGGCACACTTTCACCTTATAGTAAAAAGTACTGGATCACACATACCTT
LR-56-85818-F           CCGTCTCAAAAAAAAAAAAAAAAAAAAA
LR-56-85818-SIntR-rc
LR-56-85818-IntF        CCGTCTCAAAAAAAAAAAAAAAAAAAAA
LR-56-85818-R-rc        ~~~~~~~~~~~AAAAAAAAAAAAAAAAAAGAGTGACTCTTAGATTCTACTTAGGGCACACTTTCACCTTATAGTAAAAAGTAC
```

Figure S2. Sequence alignment for *Alu* locus Owl_2LS_056_018502759.1:6075589-6077089 (LR-56). PCR-based genotypes indicate the target *Alu* insertion is polymorphic among *A. nancymaae* individuals, homozygous present in all three *A. azarae* individuals, while being absent from other *Aotus* species. The *Aotus* reference genome [Anan_2.0] is shown at the top followed by *A. azarae* 85818 who shares the target *Alu* insertion starting at position 929 (grey highlight), with matching flanking TSDs in yellow highlight.

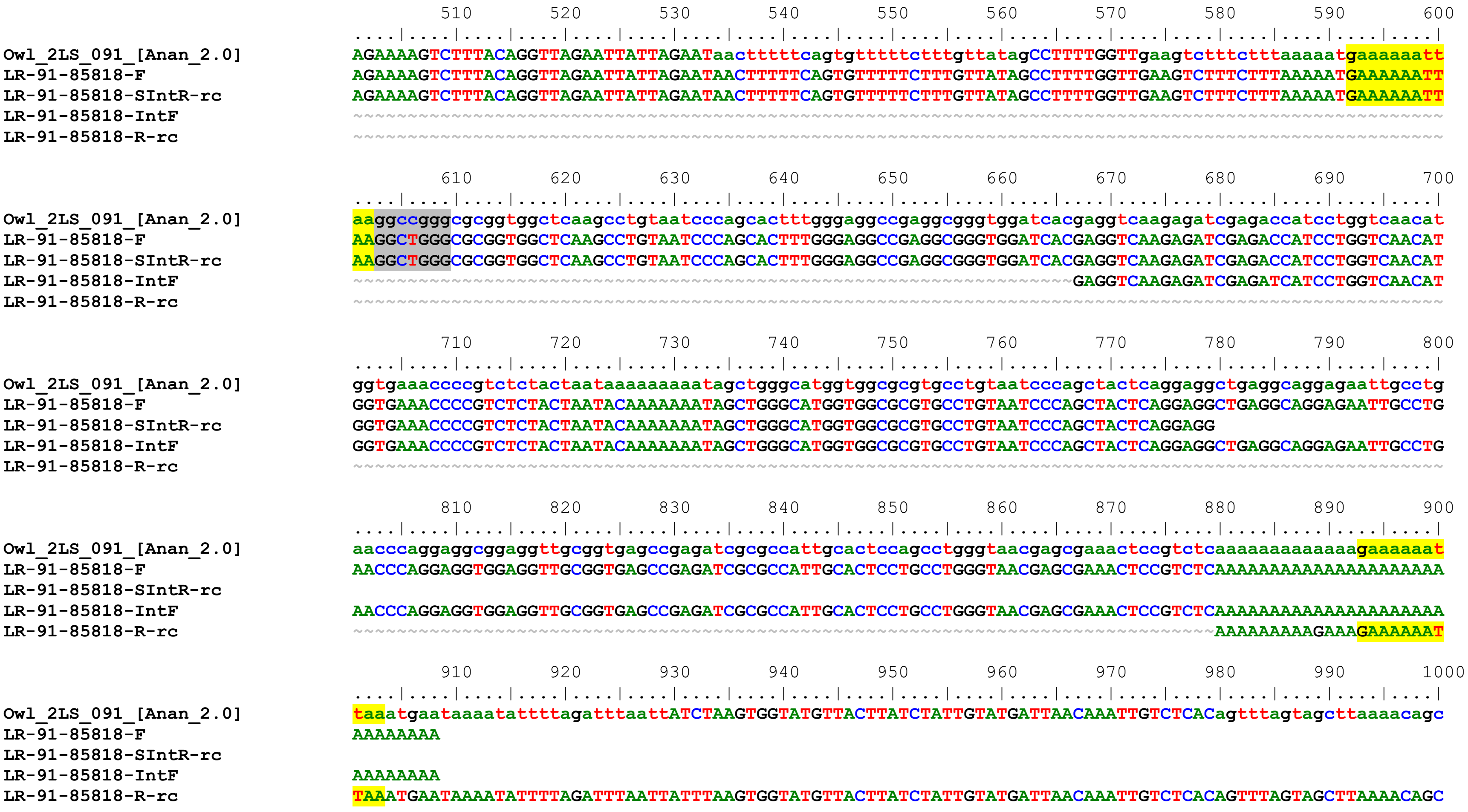

Figure S3. Sequence alignment for *Alu* locus Owl_2LS_091_018503592.1:8635400-8636896 (LR-91). PCR-based genotypes indicate the target *Alu* insertion is homozygous present among *A. nancymaae* and *A. azarae* individuals, while being absent from other *Aotus* species. The *Aotus* reference genome [Anan_2.0] is shown at the top followed by *A. azarae* 85818 who shares the target *Alu* insertion starting at position 603 (grey highlight), with matching flanking sequence and TSDs in yellow highlight.

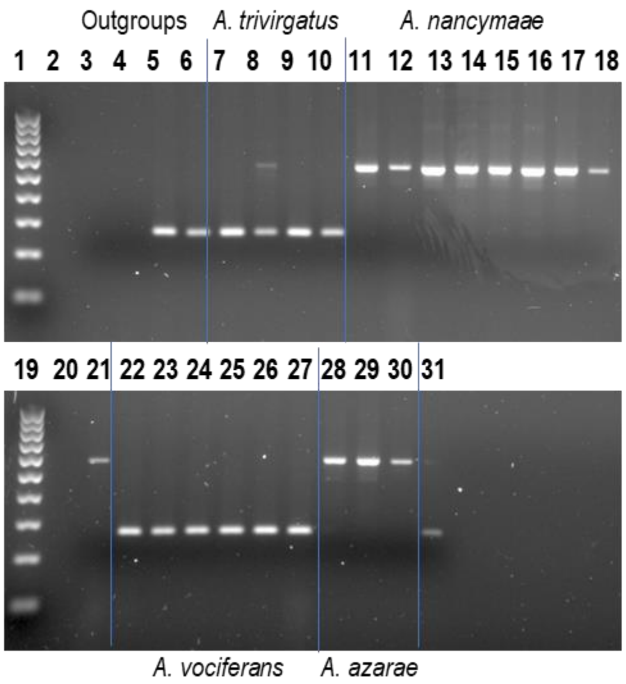

Figure S4a: *Alu* locus Owl_2LS_095_018496424.1:3086922-3088414 (LR-95): Lanes: 1, 19- 100 bp DNA ladder, 2, 20-blank, 3-TLE (negative control), 4- Human (HeLa), 5- *Callithrix jacchus* (Common marmoset), 6- *Saimiri s. sciureus* (Common squirrel monkey), 7-10 *A. trivirgatus*, 11-18, 21 *A. nancymaae*, 22-27 *A. vociferans*, 28-30 *A. azarae*, 31-*A. lemurinus griseimembra*. Vertical blue lines superimposed on the gel image visually separate the species groups. The predicted amplicon sizes are 563/266 bp. PCR-based genotypes indicate that the target *Alu* insertion is homozygous present (563 bp fragment) in *A. nancymaae* (lanes 11-18, & 21) and *A. azarae* (lanes 28-30), while homozygous absent (266 bp fragment) in *A. vociferans* (lanes 22-27), yes appears to be heterozygous for the insertion in *A. trivirgatus* sample MSB-212080 (lane 8). DNA sequencing was performed to confirm these genotypes (See Figure S4b).

```
                                    510       520       530       540       550       560       570       580       590       600
                              ....|....|....|....|....|....|....|....|....|....|....|....|....|....|....|....|....|....|....|....|
Owl_2LS_095 [Anan_2.0]        AAGATTCATTTCATATAAAGGTATgttgatataaaatttttaaagaaaacactttgaaaatg-aaaaagcataaaTAGCTCGGATGGTTTAAAAAATGcg
LR-95-212080-SIntR-rc         AAAATTCATTTCATATAAAGGTATGTTGATATAAAATTTTTAAAGAAAACACTTTGAAAATGAAAAAAGCATAAATAGCTCGGATGGTTTAAAAAATGCG
LR=95-212080-F                ~~~~~~~ATTTCATATAAAGGTATGTTGATATAAAATTTTTAAAGAAAACACTTTGAAANTGANNAAAGCATAAATAGCTCGGATGGTTTAAAAAATGCN
LR-95-212080-IntF             ~~~~~~~~~~~~~~~~~~~~~~~~~~~~~~~~~~~~~~~~~~~~~~~~~~~~~~~~~~~~~~~~~~~~~~~~~~~~~~~~~~~~~~~~~~~~~~~~~~~~
LR-95-212080-R-rc             ~~~~~~~~~~~~~~~~~~~~~~~~~~~~~~~~~~~~~~~~~~~~~~~~~~~~~~~~~~~~~~~~~~~~~~~~~~~~~~~~~~~~~~~~~~~~~~~~~~~~
LR-95-85818-SIntR-rc          AAGATTCATTTCATATAAAGGTATGTTGATATAAAATTTTTAAAGAAAACACTTTGAAAATGAAAAA~GCATAAATAGCTCGGATGGTTTAAAAAATGCG
LR-95-85818-F                 ~~~~~~~~~~~~~TATAAAGGTATGTTGATATAAAATTTTTAAAGAAAACACTTTGAAAATAGAAAAAGCATAAATAGCTCGGATGGTTTAAAAAATGCN
LR-95-85818-IntF              ~~~~~~~~~~~~~~~~~~~~~~~~~~~~~~~~~~~~~~~~~~~~~~~~~~~~~~~~~~~~~~~~~~~~~~~~~~~~~~~~~~~~~~~~~~~~~~~~~~~~
LR-95-85818-R-rc              ~~~~~~~~~~~~~~~~~~~~~~~~~~~~~~~~~~~~~~~~~~~~~~~~~~~~~~~~~~~~~~~~~~~~~~~~~~~~~~~~~~~~~~~~~~~~~~~~~~~~
LR-95-86230-E-F               ~~~~~~~~~~~~~TATAAAGGTATGTTGATATAAAATTTTTAAAGAAAACACTTTGAAAANNAANNNAGCATAAATAGCTCGGATGGTTTAAAAAATGCG
LR-95-86230-E-R-rc            AAAATTCATTTCATATAAAGGTATGTTGATATAAAATTTTTAAAGAAAACACTTTGAAAATGAAAAAAGCATAAATAGCTCGGATGGTTTAAAAAATGCG

                                    610       620       630       640       650       660       670       680       690       700
                              ....|....|....|....|....|....|....|....|....|....|....|....|....|....|....|....|....|....|....|....|
Owl_2LS_095 [Anan_2.0]        ttcggccgggcgcggtggctcaagcctgtaatcccagcactttgggaggccgaggcgggtggatcacgaggtcaagagatcaagaccatcctggtcaaca
LR-95-212080-SIntR-rc         TTCGGCCGGGCGCGGTGGCTCAAGCCTGTAATCCCAGCACTTTGGGAGGCCGAGGCGGGTGGATCACGAGGTCAATAGATCAAGACCATCCTGGTCAACA
LR=95-212080-F                NNCTTNCGGGCGCGGTGGCTCAAGCCTGTAATCCCAGCACTTTGGGAGGCCGAGGCGGGTGGATCACGAGGTCAATAGATCAAGACCATCCTGGTCAACA
LR-95-212080-IntF             ~~~~~~~~~~~~~~~~~~~~~~~~~~~~~~~~~~~~~~~~~~~~~~~~~~~~~~~~~~~~~~~~~~~GAGGTCAATAGATCAAGACCATCCTGGTCAACA
LR-95-212080-R-rc             ~~~~~~~~~~~~~~~~~~~~~~~~~~~~~~~~~~~~~~~~~~~~~~~~~~~~~~~~~~~~~~~~~~~~~~~~~~~~~~~~~~~~~~~~~~~~~~~~~~~~
LR-95-85818-SIntR-rc          TTCGGCCGGGCGCGGTGGCTCAAGCCTGTAATCCCAGCACTTTGGGAGGCCGAGGCGGGTGGATCACGAGGTCAAGAGATGGAGACCATCCTGGTCAACA
LR-95-85818-F                 TTCGGCCGGGCGCGGTGGCTCAAGCCTGTAATCCCAGCACTTTGGGAGGCCGAGGCGGGTGGATCACGAGGTCAAGAGATGGAGACCATCCTGGTCAACA
LR-95-85818-IntF              ~~~~~~~~~~~~~~~~~~~~~~~~~~~~~~~~~~~~~~~~~~~~~~~~~~~~~~~~~~~~~~~~~~~GAGGTCAAGAGATGGAGACCATCCTGGTCAACA
LR-95-85818-R-rc              ~~~~~~~~~~~~~~~~~~~~~~~~~~~~~~~~~~~~~~~~~~~~~~~~~~~~~~~~~~~~~~~~~~~~~~~~~~~~~~~~~~~~~~~~~~~~~~~~~~~~
LR-95-86230-E-F               TTC~~~~~~~~~~~~~~~~~~~~~~~~~~~~~~~~~~~~~~~~~~~~~~~~~~~~~~~~~~~~~~~~~~~~~~~~~~~~~~~~~~~~~~~~~~~~~~~~~
LR-95-86230-E-R-rc            TTC~~~~~~~~~~~~~~~~~~~~~~~~~~~~~~~~~~~~~~~~~~~~~~~~~~~~~~~~~~~~~~~~~~~~~~~~~~~~~~~~~~~~~~~~~~~~~~~~~

                                    710       720       730       740       750       760       770       780       790       800
                              ....|....|....|....|....|....|....|....|....|....|....|....|....|....|....|....|....|....|....|....|
Owl_2LS_095 [Anan_2.0]        tggtgaaaccccgtctctactaatacaaaaaaatagctgggcatggtggcgcgtgcctgtaatcccagctgctcaggaggctgaggcaggagaattgact
LR-95-212080-SIntR-rc         TGATGAAACCCCGTCTCTACTAATACAAAAAACTAGCTGGGCATGGTGGCGCGTGCCTGTAATCCCAGCTACTCAGGAGGC
LR=95-212080-F                TGATGAAACCCCGTCTCTACTAAGACAAAAAACTA
LR-95-212080-IntF             TGATGAAACCCCGTCTCTACTAATACAAAAAACTAGCTGGGCATGGTGGCGCGTGCCTGTAATCCCAGCTACTCAGGAGGCTGAGGCAGGAGAATTGCCT
LR-95-212080-R-rc             ~~~~~~~~~~~~~~~~~~~~~~~~~~~~~~~~~~~~~~~~~~~~~~~~~~~~~~~~~~~~~~~~~~~~~~~~~~~~~~~~~~~~~~~~~~~~~~~~~~~~
LR-95-85818-SIntR-rc          TGGTGAAACCCCGTCTCTACTAATACAAAAAACTAGCTGGGCATGGTGGCGCGTGCCTGTAATCCCAGCTACTCAGGAGG
LR-95-85818-F                 TGGTGAAACCCCGTCTCTACTAATACAAAAAACTAGCTGGGCATGGTGGCGCGTGCCTGTAATCCCAGCTACTCAGGAGGCTGAGGCAGGAGAATTGACT
LR-95-85818-IntF              TGGTGAAACCCCGTCTCTACTAATACAAAAAACTAGCTGGGCATGGTGGCGCGTGCCTGTAATCCCAGCTACTCAGGAGGCTGAGGCAGGAGAATTGACT
LR-95-85818-R-rc              ~~~~~~~~~~~~~~~~~~~~~~~~~~~~~~~~~~~~~~~~~~~~~~~~~~~~~~~~~~~~~~~~~~~~~~~~~~~~~~~~~~~~~~~~~~~~~~~~~~~~
LR-95-86230-E-F               ~~~~~~~~~~~~~~~~~~~~~~~~~~~~~~~~~~~~~~~~~~~~~~~~~~~~~~~~~~~~~~~~~~~~~~~~~~~~~~~~~~~~~~~~~~~~~~~~~~~~
LR-95-86230-E-R-rc            ~~~~~~~~~~~~~~~~~~~~~~~~~~~~~~~~~~~~~~~~~~~~~~~~~~~~~~~~~~~~~~~~~~~~~~~~~~~~~~~~~~~~~~~~~~~~~~~~~~~~
```

```
                                     810       820       830       840       850       860       870       880       890       900
                             ....|....|....|....|....|....|....|....|....|....|....|....|....|....|....|....|....|....|....|....|
Owl_2LS_095 [Anan_2.0]       gaacccaggaggcggaggttgcggtgagccgagatagcgccattgcactccagcctgggtaacgagcgaaactccgtctcaaaaaaaaaaaaaaaaagcgt
LR-95-212080-SIntR-rc
LR=95-212080-F
LR-95-212080-IntF            GAACCCAGGAGGCGAAGGTTGCGGTGAGCCGAGATCGCGCCATTGCACTCCAGCCTGGGTAATGAGCGAAACTCCGTCTCAAAAAAA
LR-95-212080-R-rc            ~~~~~~~~~~~~~~~~~~~~~~~~~~~~~~~~~~~~~~~~~~~~~~~~~~~~~~~~~~~~~~~~~~~~~~~~~~~~~~~~~~~~AAAAAAAAAAAAGCGT
LR-95-85818-SIntR-rc
LR-95-85818-F                GAACCCAGGAGGCGGAGGTTGCGGTGAGCCGAGATCGCGCCATTGCACTCCAGCC
LR-95-85818-IntF             GAACCCAGGAGGCGGAGGTTGCGGTGAGCCGAGATCGCGCCATTGCACTCCAGCCTGGGTAACGAGCGAAACTCCGTCTCAAAAAAAAAAAAAAAAAAAAA
LR-95-85818-R-rc             ~~~~~~~~~~~~~~~~~~~~~~~~~~~~~~~~~~~~~~~~~~~~~~~~~~~~~~~~~~~~~~~~~~~~AAAAAAAAAAAAAAAAAAAAAAAAAAATGCGT
LR-95-86230-E-F              ~~~~~~~~~~~~~~~~~~~~~~~~~~~~~~~~~~~~~~~~~~~~~~~~~~~~~~~~~~~~~~~~~~~~~~~~~~~~~~~~~~~~~~~~~~~~~~~~~~~~~
LR-95-86230-E-R-rc           ~~~~~~~~~~~~~~~~~~~~~~~~~~~~~~~~~~~~~~~~~~~~~~~~~~~~~~~~~~~~~~~~~~~~~~~~~~~~~~~~~~~~~~~~~~~~~~~~~~~~

                                     910       920       930       940       950       960       970       980       990      1000
                             ....|....|....|....|....|....|....|....|....|....|....|....|....|....|....|....|....|....|....|....|
Owl_2LS_095 [Anan_2.0]       tcatctcatatgagaaaaaaaattcatttatcctGATGTATACTGGACcctagtttttcttttcagattccACTGAGTAGCAGTCATTTCTTTTGATAGA
LR-95-212080-SIntR-rc
LR=95-212080-F
LR-95-212080-IntF
LR-95-212080-R-rc            TCATCTCATCTGAGNAAANNNNNN~~TTTATCCTGATGTATACTGGACCCTAGTNNTCTTNTCAGACTCCAC
LR-95-85818-SIntR-rc
LR-95-85818-F
LR-95-85818-IntF
LR-95-85818-R-rc             TCATCTCATATGAGAAAAAAAATTCATTTATCCTGATGTATACTGGACCCTAGTTTTTCTTTTCNAGATTCCACTGA
LR-95-86230-E-F              ~~ATCTCATCTGAGAAAAAAAATTCATTTATCCTGATGTATACTGGACCCTAGTTTTTCTTTTCAGACTCCACTGAGTAGCAGTCATTTCTTTTGATAGG
LR-95-86230-E-R-rc           ~~ATCTCATCTGAGAAAAAAAATTNATTTATCCTGATGTATACTGGACCCTAG
```

Figure S4b. Sequence alignment for *Alu* locus Owl_2LS_095_018496424.1:3086922-3088414 (LR-95). PCR-based genotypes (Figure S4a) indicate the target *Alu* insertion is homozygous present (1/1) in all *A. nancymaae* and *A. azarae* individuals and homozygous absent (0/0) in all *A. vociferans* samples, yet a single *A. trivirgatus* individual (MSB-212080) appears to be heterozygous (1/0) for the insertion. DNA sequencing was performed to confirm this pattern. The reference genome [Anan_2.0] is shown at the top followed by *A. trivirgatus* MSB-212080 (filled site amplicon) and *A. azarae* 85818, both of whom share the target *Alu* insertion starting at position 604 (grey highlight) and flanked by TSDs in yellow highlight. Sequencing of the empty site amplicon for *A. vociferans* 86230 displays a precise pre-integration site with forward and reverse sequence traces spanning the target region, confirming the genotypes. This pattern supports grey-neck and red-neck grouping and supports *A. vociferans* as basal to *A. trivirgatus* among the *Aotus* species on our panel.

```
                                     510       520       530       540       550       560       570       580       590       600
                            ....|....|....|....|....|....|....|....|....|....|....|....|....|....|....|....|....|....|....|....|
Owl_2LS_111_[Anan_2.0]      ATTTGGAAGTAAGGAAGGTCTCGCTCTAAGAGTTTACCTATGAGACTAACTCACAGCAGAACAGAGATATGCAAAATCGCCGTTAAGAATCTGAttaatc
LR-111-85818-F              ATTTGGAAGTAAGGAAGGTCTCGCTCTAAGAGTTTACCTATGAGACTAACTCACAGCAGAACAGAGATATGCAAAATCGCCGTTAAGAATCTGATGAATC
LR-111-85818-IntF           ~~~~~~~~~~~~~~~~~~~~~~~~~~~~~~~~~~~~~~~~~~~~~~~~~~~~~~~~~~~~~~~~~~~~~~~~~~~~~~~~~~~~~~~~~~~~~~~~~~~~
LR-111-85818-SIntR-rc       ATTTGGAAGTAAGGAAGGTCTCGCTCTAAGAGTTTACCTATGAGACTAACTCACAGCAGAACAGAGATATGCAAAATCGCCGTTAAGAATCTGATGAATC

                                     610       620       630       640       650       660       670       680       690       700
                            ....|....|....|....|....|....|....|....|....|....|....|....|....|....|....|....|....|....|....|....|
Owl_2LS_111_[Anan_2.0]      ggccgggcgcggtggctcaagcctgtaatcccagcactttgggaggccgaggcgggtggatcacgaggtcaagagatcgagaccatcctggtcaacatgg
LR-111-85818-F              GGCCGGGCGCGGTGGCTCAAGCCTGTAATCCCAGCACTTTGGGAGGCCGAGGCAGGTGGATCACGAGGTCAAGAGATCGAGACCATCCTGGTCAACATGG
LR-111-85818-IntF           ~~~~~~~~~~~~~~~~~~~~~~~~~~~~~~~~~~~~~~~~~~~~~~~~~~~~~~~~~~~~~~~~GAGGTCAAGAGATCGAGACCATCCTGGTCAACATGG
LR-111-85818-SIntR-rc       GGCCGGGCGCGGTGGCTCAAGCCTGTAATCCCAGCACTTTGGGAGGCCGAGGCAGGTGGATCACGAGGTCAAGAGATCGAGACCATCCTGGTCAACATGG

                                     710       720       730       740       750       760       770       780       790       800
                            ....|....|....|....|....|....|....|....|....|....|....|....|....|....|....|....|....|....|....|....|
Owl_2LS_111_[Anan_2.0]      tgaaaccccgtctctactaaaaa~~tacaaaaaattagctgggcatggtggcgcgtgcctgtaatcccagctactcaggaggctgaggcaggagaattgc
LR-111-85818-F              TGAAACTCCGTCTCTACTAAAAAAATACNAAAAA
LR-111-85818-IntF           TGAAACTCCGTCTCTACTAAAAAA~TACAAAAAATTAGCTGGGCATGGTGGTGCGTGCCTGTAATCCCAGCTACTCAGGAGGCTGAGGCAGGAGAATTGC
LR-111-85818-SIntR-rc       TGAAACTCCGTCTCTACTAAAAAA~TACAAAAAATTAGCTGGGCATGGTGGTGCGTGCCTGTAATCCCAGCTACTCAGGAGG

                                     810       820       830       840       850       860       870       880       890       900
                            ....|....|....|....|....|....|....|....|....|....|....|....|....|....|....|....|....|....|....|....|
Owl_2LS_111_[Anan_2.0]      ctgaacccaggaggcagaggttgcggtgagctgagatcgcgccattgcactccagcctgggtaacgagagcgaaactccgtctcaaaaaaaaaaaaaaag
LR-111-85818-F
LR-111-85818-IntF           CTGAACCCAGGAGGCGGAGGTTGTAGTGAGCCGAGATCGCGCCATTGCACTCCAGCCTGGGTAACGAGAGCGAAACTCCGTCTCAAAAAAAAAAAAAAAA
LR-111-85818-SIntR-rc

                                     910       920       930       940       950       960       970       980       990      1000
                            ....|....|....|....|....|....|....|....|....|....|....|....|....|....|....|....|....|....|....|....|
Owl_2LS_111_[Anan_2.0]      aatctgattaACTCCCTGGACTTTGCAACCAGACTGCATATAATAAGAtacttaaaggaaataaaacatctgTTGTCAATGGGATGTGGTAGAGGAGGTA
LR-111-85818-F
LR-111-85818-IntF           AAAAAA
LR-111-85818-SIntR-rc
```

Figure S5. Sequence alignment for *Alu* locus Owl_2LS_111_018494522.1:1204876-1206375 (LR-111). PCR-based genotypes indicate the target *Alu* insertion is homozygous present among *A. nancymaae* and *A. azarae* individuals, while being absent from other *Aotus* species. The *Aotus* reference genome [Anan_2.0] is shown at the top followed by *A. azarae* 85818 who shares the target *Alu* insertion starting at position 601 (grey highlight), with matching 5' flanking sequence. Sequencing using the reverse primer failed to produce sequence traces so 3' flanking sequence is unavailable. The target insertion TSDs are shown in yellow highlight.

```
                                        510       520       530       540       550       560       570       580       590       600
                               ....|....|....|....|....|....|....|....|....|....|....|....|....|....|....|....|....|....|....|....|
Aotus_359-rc [Anan_2.0]        AACTGTAGAATTACAAGCCTATGTTTTACATGTTCATGTAGCCTTTCAGAATAGTCTGGTTCAAGTTATTCAGAAATGTTTTCCTTAGAACTGAAAttaa
JW-359-85818-SIntR-rc          AACTGTAGAATTACAAGCCTATGTTTTACATGTTCATGTAGCCTTTCAGAATAGTCTGGTTCAAGTTATTCAGAAATGTTTTCCTTAGAACTGAAATTAA
JW-359-85818-R                 AACTGTAGAATTACAAGCCTATGTTTTACATGTTCATGTAGCCTTTCAGAATAGTCTGGTTCAAGTTATTCAGAAATGTTTTCCTTAGAACTGAAATTAA
JW-359-85818-IntF              ~~~~~~~~~~~~~~~~~~~~~~~~~~~~~~~~~~~~~~~~~~~~~~~~~~~~~~~~~~~~~~~~~~~~~~~~~~~~~~~~~~~~~~~~~~~~~~~~~~~~
JW-359-85818-F-rc              ~~~~~~~~~~~~~~~~~~~~~~~~~~~~~~~~~~~~~~~~~~~~~~~~~~~~~~~~~~~~~~~~~~~~~~~~~~~~~~~~~~~~~~~~~~~~~~~~~~~~

                                        610       620       630       640       650       660       670       680       690       700
                               ....|....|....|....|....|....|....|....|....|....|....|....|....|....|....|....|....|....|....|....|
Aotus_359-rc [Anan_2.0]        gaggccgggtgcggtggctcaagcctgtaatcccagcactttgggaggccgaggcgggtggatcacgaggtcaatagatcgagaccatcctggtcaacat
JW-359-85818-SIntR-rc          GAGGCCGGGCGCGGTGGCTCAAGCCTGTAATCCCAGCACTTTGGGAGGCCGAGGCGGGTGGATCACGAGGGCAAGAGATTGAGACCATCCTGGTCAACAT
JW-359-85818-R                 GAGGCCGGGCGCGGTGGCTCAAGCCTGTAATCCCAGCACTTTGGGAGGCCGAGGCGGGTGGATCACGAGGGCAAGAGATTGAGACCATCCTGGTCAACAT
JW-359-85818-IntF              ~~~~~~~~~~~~~~~~~~~~~~~~~~~~~~~~~~~~~~~~~~~~~~~~~~~~~~~~~~~~~~~~~~GAGGGCAAGAGATTGAGACCATCCTGGTCAACAT
JW-359-85818-F-rc              ~~~~~~~~~~~~~~~~~~~~~~~~~~~~~~~~~~~~~~~~~~~~~~~~~~~~~~~~~~~~~~~~~~~~~~~~~~~~~~~~~~~~~~~~~~~~~~~~~~~~

                                        710       720       730       740       750       760       770       780       790       800
                               ....|....|....|....|....|....|....|....|....|....|....|....|....|....|....|....|....|....|....|....|
Aotus_359-rc [Anan_2.0]        ggtgaaaccccgtctctactaatacaaaaaattagctgggcacggtggcgcgtgcctgtaatcccagctactcaggaggctgaggcaggagaattgcctg
JW-359-85818-SIntR-rc          GGTGAAACCCCGTCTCTACTAATACAAAAAATTAGCTGGGCACGGTGGCGCGTGCCTGTAATCCCAGCTACTCAGGAGGCT
JW-359-85818-R                 GGTGAAACCCCGTCTCTACTAATACAAAAAATTAGCTGGGCACGGTGGCGCGTGCCTGTAATCCCAGCTACTCAGGAGGCTGAGGCAGGAGAATTGCCTG
JW-359-85818-IntF              GGTGAAACCCCGTCTCTACTAATACAAAAAATTAGCTGGGCACGGTGGCGCGTGCCTGTAATCCCAGCTACTCAGGAGGCTGAGGCAGGAGAATTGCCTG
JW-359-85818-F-rc              ~~~~~~~~~~~~~~~~~~~~~~~~~~~~~~~~~~~~~~~~~~~~~~~~~~~~~~~~~~~~~~~~~~~~~~~~~~~~~~~~~~~~~~~~~~~~~~~~~~~~

                                        810       820       830       840       850       860       870       880       890       900
                               ....|....|....|....|....|....|....|....|....|....|....|....|....|....|....|....|....|....|....|....|
Aotus_359-rc [Anan_2.0]        aacccaggaggcggaggttgcggtgagccgagatcgcgccactgcactccagcctgggtatcaagagcgaaactccgtctcaaaaaaaaaaaaaaaaaaa
JW-359-85818-SIntR-rc
JW-359-85818-R                 AACCCAGGAGGCGGAGGTTGCGGTGAGCCGAGATCGCGCCATTGCACTCCAGCCTGGGTATCAAGAG~~AAACTCCCCCTCTCAAAAAAAAAAAAAAAAA
JW-359-85818-IntF              AACCCAGGAGGCGGAGGTTGCGGTGAGCCGAGATCGCGCCATTGCACTCCAGCCTGGGTATC
JW-359-85818-F-rc              ~~~~~~~~~~~~~~~~~~~~~~~~~~~~~~~~~~~~~~~~~~~~~~~~~~~~~~~~~~~~~~~~~~~~~~~~~~~~~~~~~~~~AAAAAAAAAAAAAAAA

                                        910       920       930       940       950       960       970       980       990      1000
                               ....|....|....|....|....|....|....|....|....|....|....|....|....|....|....|....|....|....|....|....|
Aotus_359-rc [Anan_2.0]        aaaaaaaaaagaactgaaattaagtAGTTGAAACgacatatgaaatattttgactttcattttgttttagagagaGACTACTGAAACTAAAGTTTTCATT
JW-359-85818-SIntR-rc
JW-359-85818-R                 AAAAAAAAAAA
JW-359-85818-IntF
JW-359-85818-F-rc              AAAAAAAAAAGAACTGAAATTAAGTAGTTGAAACGACATATGAAATATTTTGACTTTCATTTTGTTTTAGAGAGAGACTACTGAAACTAAAG
```

Figure S6. Sequence alignment for *Alu* locus *Aotus*_359_018505794.1:22106446-22107953 (JW-359). PCR-based genotypes indicate the target *Alu* insertion is homozygous present (1/1) in all *A. nancymaae* and *A. azarae* while absent in other *Aotus* species. The reference genome [Anan_2.0] is shown at the top followed by A. azarae 85818 who shares the target insertion starting at position 603 (grey highlight) with flanking TSDs in yellow highlight. Sequencing confirms the genotypes linking *A. nancymaae* and *A. azarae*.

```
                              510       520       530       540       550       560       570       580       590       600
                      ....|....|....|....|....|....|....|....|....|....|....|....|....|....|....|....|....|....|....|....|
Aotus_420_[Anan_2.0]  AGGAATGCCATGAGAAAATGGCACTGCATTTACCAAGTCATCCCTAGATTCTTGTGCAAAGAAAAAGATTGAGACTATGTATTAACAATTTCATAAACAA
JW-420-85818-F        ~~~~~~~~~~~~~~~~~~~~~~~~~~~~~~~~~~~~~~~~~~~~~~~~~~~~~~~~~~~~~~~~~~~~~TGAGACTATGTATTAACAATTTCATAAACAA
JW-420-85818-SIntR-rc ~~~~~~~~~~~~~~~~~~TGGCACTGCATTTACCAAGTCATCCCTAGATTCTTGTGCAAAGAAAAAGATTGAGACTATGTATTAACAATTTCATAAACAA
JW-420-85818-IntF     ~~~~~~~~~~~~~~~~~~~~~~~~~~~~~~~~~~~~~~~~~~~~~~~~~~~~~~~~~~~~~~~~~~~~~~~~~~~~~~~~~~~~~~~~~~~~~~~~~~~~
JW-420-85818-R-rc     ~~~~~~~~~~~~~~~~~~~~~~~~~~~~~~~~~~~~~~~~~~~~~~~~~~~~~~~~~~~~~~~~~~~~~~~~~~~~~~~~~~~~~~~~~~~~~~~~~~~~

                              610       620       630       640       650       660       670       680       690       700
                      ....|....|....|....|....|....|....|....|....|....|....|....|....|....|....|....|....|....|....|....|
Aotus_420_[Anan_2.0]  ataatcaggccgggcgcggtggctcatgcctgtaatcccagcactttgggaggccgaggcgggtggatcacgaggtcaagagatcgagaccattctggtc
JW-420-85818-F        ATAATCAGGCCGGGCGCGGTGGCTCACGCCTGTAATCCCAGCACTTTGGGAGGCCGAGGCGGGTGGATCACGAGGTCAAGAGATCGAGACCATTCTGGTC
JW-420-85818-SIntR-rc ATAATCAGGCCGGGCGCGGTGGCTCACGCCTGTAATCCCAGCACTTTGGGAGGCCGAGGCGGGTGGATCACGAGGTCAAGAGATCGAGACCATTCTGGTC
JW-420-85818-IntF     ~~~~~~~~~~~~~~~~~~~~~~~~~~~~~~~~~~~~~~~~~~~~~~~~~~~~~~~~~~~~~~~~~~~~~~~GAGGTCAAGAGATCGAGACCATTCTGGTC
JW-420-85818-R-rc     ~~~~~~~~~~~~~~~~~~~~~~~~~~~~~~~~~~~~~~~~~~~~~~~~~~~~~~~~~~~~~~~~~~~~~~~~~~~~~~~~~~~~~~~~~~~~~~~~~~~~

                              710       720       730       740       750       760       770       780       790       800
                      ....|....|....|....|....|....|....|....|....|....|....|....|....|....|....|....|....|....|....|....|
Aotus_420_[Anan_2.0]  aacatggtgaaaccccgtctctactaatacaaaaaattagctgggcatggtggcgcgtgcctgtaatcccagctactcaggaggctgaggcaggagaatt
JW-420-85818-F        AACATGGTGAAACCCCGTCTCTACTAATACAAAAAATTAGCTGGGCATGGTGGCGCGTGCCTGTAATCCCAGCTACTCAGGAGGCTGAGGCAGGAGAATT
JW-420-85818-SIntR-rc AACATGGTGAAACCCCGTCTCTACTAATACAAAAAATTAGCTGGGCATGGTGGCGCGTGCCTGTAATCCCAGCTACTCAGGAGG
JW-420-85818-IntF     AACATGGTGAAACCCCGTCTCTACTAATACAAAAAATTAGCTGGGCATGGTGGCGCGTGCCTGTAATCCCAGCTACTCAGGAGGCTGAGGCAGGAGAATT
JW-420-85818-R-rc     ~~~~~~~~~~~~~~~~~~~~~~~~~~~~~~~~~~~~~~~~~~~~~~~~~~~~~~~~~~~~~~~~~~~~~~~~~~~~~~~~~~~~~~~~~~~~~~~~~~~~

                              810       820       830       840       850       860       870       880       890       900
                      ....|....|....|....|....|....|....|....|....|....|....|....|....|....|....|....|....|....|....|....|
Aotus_420_[Anan_2.0]  gcctgaacccaggaggcggaggttgcggtgagccgagatcgcgccattgcactccagcctgggtaacaagagcgaaactccgtctcaaaaaaaaaaaaaa
JW-420-85818-F        GCCTGAACCCAGGAGGCGGAGGTTGCGGTGAGCCGAGATCGCGCCATTGCACTCCAGCCTGGGTAACAAGAGC
JW-420-85818-SIntR-rc
JW-420-85818-IntF     GCCTGAACCCAGGAGGCGGAGGTTGCGGTGAGCCGAGATCGCGCCATTGCACTCCAGCCTGGGTAACAAGAGCGAAACTCCGTCTCAAAAAAAAA
JW-420-85818-R-rc     ~~~~~~~~~~~~~~~~~~~~~~~~~~~~~~~~~~~~~~~~~~~~~~~~~~~~~~~~~~~~~~~~~~~~~~~~~~~~~~~~~~~~~~~~~~~~~~~~~~~~

                              910       920       930       940       950       960       970       980       990      1000
                      ....|....|....|....|....|....|....|....|....|....|....|....|....|....|....|....|....|....|....|....|
Aotus_420_[Anan_2.0]  aaacaaataatcaacaATTTCACTCCATTATTTTGATGTTGGGTAAAGCCTGAAATGTGGTGTCAAGTTTTTCCATTTGCATTACATCAAATTCTTGTCT
JW-420-85818-F
JW-420-85818-SIntR-rc
JW-420-85818-IntF
JW-420-85818-R-rc     ~~~~~~~~~~~~~~~~~~~~~~~~~~~~~~~~~~~~~~~~~~~~~~~~~~~~~~~~~~~~~~~~~~~~~~~~~~~~~~~~~~~~~~~~~~~~~~~~~~~~

                             1010      1020      1030      1040      1050      1060      1070      1080      1090      1100
                      ....|....|....|....|....|....|....|....|....|....|....|....|....|....|....|....|....|....|....|....|
Aotus_420_[Anan_2.0]  ACTATGGATATTCTGATATGACAAAGACTAAATTCCTATTGAAACATTTGCCATTTACTACACTCACATAACTTCTCTTAGGTATAGATTTTTTGATGTT
JW-420-85818-F
JW-420-85818-SIntR-rc
JW-420-85818-IntF
JW-420-85818-R-rc     ~~~~~~~~~~~~~~~~~~~~~~~~~~~~~~~~~~~~~~~~~~~~~~~~~~~~~~~~~~~~~~~~~~~~~~~~~~~~~~~~~~~~~~~~~~~~~~~~~~~~
```

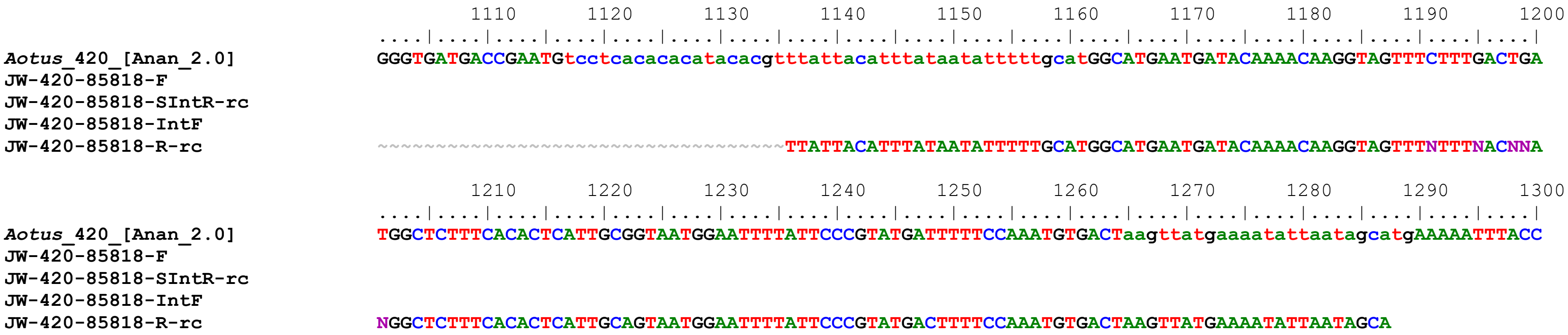

Figure S7. Sequence alignment for *Alu* locus *Aotus*_420_ 018497736.1:502088-503587 (JW-420). PCR-based genotypes indicate the target *Alu* insertion is homozygous present (1/1) in all *A. nancymaae* and *A. azarae* while absent in other *Aotus* species. The reference genome [Anan_2.0] is shown at the top followed by A. azarae 85818 who shares the target insertion starting at position 608 (grey highlight) with flanking TSDs in yellow highlight. The reverse primer sequence trace initiates at position 1287 but does not extend back to the A-tail of the target insertion, however the forward and SIntR traces confirm the genotypes linking *A. nancymaae* and *A. azarae*.

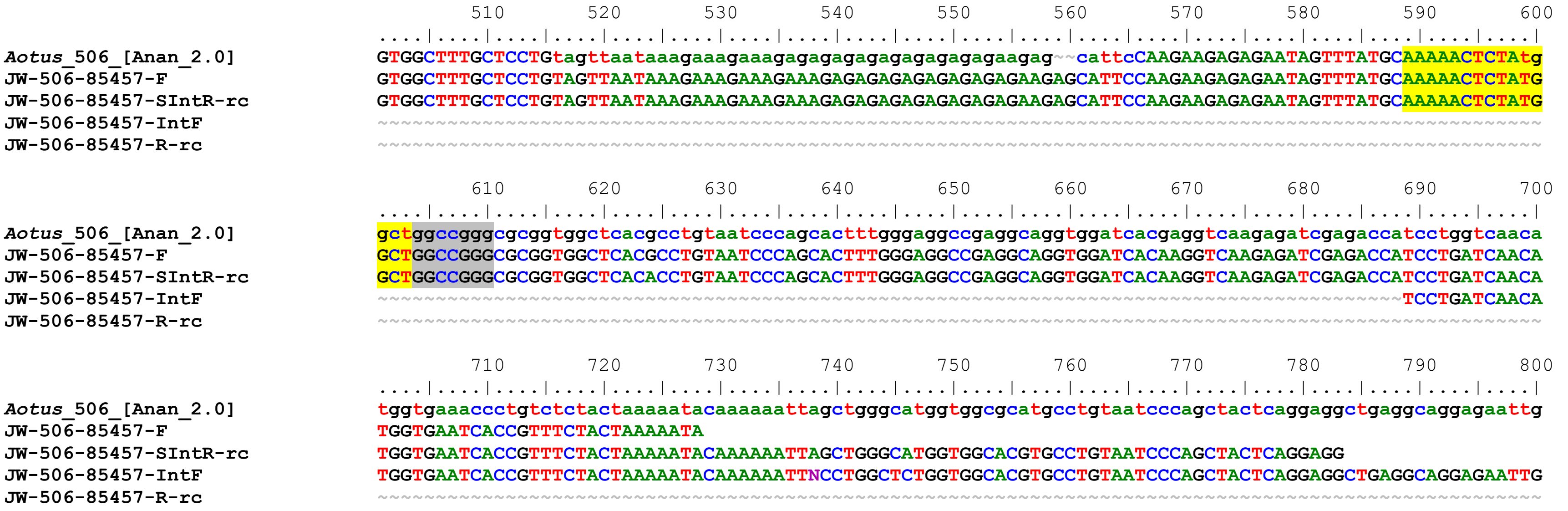

```
                              810       820       830       840       850       860       870       880       890       900
                     ....|....|....|....|....|....|....|....|....|....|....|....|....|....|....|....|....|....|....|....|
Aotus_506_[Anan_2.0] cctgaacccaggaggcggaggttgcggtgagccgagatcgcgccattgcactccagcctgggtaacaagagcgaaactccgtctcaaaaaaaaaaaaaaa
JW-506-85457-F
JW-506-85457-SIntR-rc
JW-506-85457-IntF    CCTGAACCCAGGAGGCGGAGGTTGCGGTGAGCCGAGATCGCGCCATTGCACTCCAGCCTGGGTAACAAGAGCGAAACTCCGTCTCCAAAAAAA
JW-506-85457-R-rc    ~~~~~~~~~~~~~~~~~~~~~~~~~~~~~~~~~~~~~~~~~~~~~~~~~~~~~~~~~~~~~~~~~~~~~~~~~~~~~~~~~~AAAAAAAAAAAAAAAAAA

                              910       920       930       940       950       960       970       980       990      1000
                     ....|....|....|....|....|....|....|....|....|....|....|....|....|....|....|....|....|....|....|....|
Aotus_506_[Anan_2.0] aaattctatggctTAGGCAAGAAGCAATCCATGCGGGGCTTTATAGGCCATATCAAAGAATTCTGTCCTAATCCTAAAACCATAGAAAGCCACTTGGGGT
JW-506-85457-F
JW-506-85457-SIntR-rc
JW-506-85457-IntF
JW-506-85457-R-rc    AAACTCTATGGCTTAGGCAAGAAGCAATCCATGCGGGG
```

Figure S8. Sequence alignment for *Alu* locus *Aotus*_506_018511366.1:24422032-24423531 (JW-506). PCR-based genotypes indicate the target *Alu* insertion is present in *A. nancymaae* and *A. azarae* while absent in other *Aotus* species. The reference genome [Anan_2.0] is shown at the top followed by *A. azarae* 85457 who shares the target insertion starting at position 604 (grey highlight) with flanking TSDs in yellow highlight. Sequencing confirms the genotypes linking *A. nancymaae* and *A. azarae*.

```
                              510       520       530       540       550       560       570       580       590       600
                     ....|....|....|....|....|....|....|....|....|....|....|....|....|....|....|....|....|....|....|....|
Aotus_592_[Anan_2.0] AGGCAACCTAGTTCATCACTTAGACTGGAAATCATGTCTTCTAATTCTCAGTGATTTGCTCTTTCTACTGAATCAAAATTattcaattcaaaaaaaaaat
JW-592-85457-R       NNNNAACCTAGTTCATCACTTAGACTGGAAATCATGTCTTNNNNTTCTCAGTGATTTGCTCTTTCTACTGAATCAAAATTATTCAATTCAAA
JW-592-85457-SIntR-rc ~~~~~~~~~~~~~~~~~~~~~~~~~~~~~~~~~~~~~~~~~~~~~~~~~~~~~~~~~~~~~~~~~~~~~~~~~~~~~~~~~~~~AATTCAAAAAAAAAAT
JW-592-85457-IntF    ~~~~~~~~~~~~~~~~~~~~~~~~~~~~~~~~~~~~~~~~~~~~~~~~~~~~~~~~~~~~~~~~~~~~~~~~~~~~~~~~~~~~~~~~~~~~~~~~~~~~

                              610       620       630       640       650       660       670       680       690       700
                     ....|....|....|....|....|....|....|....|....|....|....|....|....|....|....|....|....|....|....|
Aotus_592_[Anan_2.0] actgccgggcgcggtggctcacgcctgtaatcccagcactttgggaggccgaggcgggtggatcatgaggtcaagagatcgagaccatcctggtcaacat
JW-592-85457-R
JW-592-85457-SIntR-rc ACTGCCGGGCGCGGTGGCTCACGCCTGTAATCCCAGCACTTTGGGAGGCCGAGGCGGGTGGATCATGAGGTCAAGAGATCGAGACCATCATGGTCAACAT
JW-592-85457-IntF    ~~~~~~~~~~~~~~~~~~~~~~~~~~~~~~~~~~~~~~~~~~~~~~~~~~~~~~~~~~~~~~~~~~~~~~~~~~~~~~~~~~GACCATCCTGGTCAACAT

                              710       720       730       740       750       760       770       780       790       800
                     ....|....|....|....|....|....|....|....|....|....|....|....|....|....|....|....|....|....|....|
Aotus_592_[Anan_2.0] ggtgaaaccccgtctctactaaaaatacaaaaaattagctgggca~tggtggcgcgtgcctgtaatcccagctactcaggaggctgaggcaggagaattg
JW-592-85457-R
JW-592-85457-SIntR-rc GGTGAAACCCCGTCTCTACTAAAAATACAAAAAATTAGCTGGGCA~TGGTGGCGCGTGCCTGTAATCCCAGCTACTCAGGAGG
JW-592-85457-IntF    GGTGAAACCCCGTCTCTACTAAAAATACAAACNAGAACAAAAGTCCTGGTGACACGTGCCTGTAATCCCAGCTACTCAGGAGGCTGAGGCAGGAGAATTG

                              810       820       830       840       850       860       870       880       890       900
                     ....|....|....|....|....|....|....|....|....|....|....|....|....|....|....|....|....|....|....|
Aotus_592_[Anan_2.0] cctgaacccaggaggcagaggttgcggtgagccgagatcgcgccattgcactccagcctgggtaacaggagcaaaactccgtctcaaaaaaaaaaaaaaa
JW-592-85457-R
JW-592-85457-SIntR-rc
JW-592-85457-IntF    CCTGAACCCAGGAGGCAGAGGTTGCGGTGAGCCGAGATCGCACCATTGCACTCCAGCCTGGGTAACAGGAGCAAAA
```

```
                                        910       920       930       940       950       960       970       980       990      1000
                                ....|....|....|....|....|....|....|....|....|....|....|....|....|....|....|....|....|....|....|....|
Aotus_592_[Anan_2.0]            aaaaaaatacttaggaagaaGCCTGCCACTGTGACAAAGAGAGTGCCATGCTACAGCTCAGCGGTTCCTCATGAGCAGCTTGTCAAAAAGGACAAGGCCC
JW-592-85457-R
JW-592-85457-SIntR-rc
JW-592-85457-IntF
```

Figure S9. Sequence alignment for *Alu* locus *Aotus*_592_ 018503629.1:2778893-2780395 (JW-592). PCR-based genotypes indicate the target *Alu* insertion is homozygous present (1/1) in all *A. nancymaae* and *A. azarae* while absent in other *Aotus* species. The reference genome [Anan_2.0] is shown at the top followed by A. azarae 85457 who shares the target insertion starting at position 604 (grey highlight) with flanking TSDs in yellow highlight. The internal-*Alu* forward and forward-rc primer sequence traces are relatively poor, however the SIntR-rc sequence trace confirms the Alu start position and 5' TSD, confirming the genotypes linking *A. nancymaae* and *A. azarae*.

```
                                        510       520       530       540       550       560       570       580       590       600
                                ....|....|....|....|....|....|....|....|....|....|....|....|....|....|....|....|....|....|....|....|
Aotus_1202-rc [Anan_2.0]        TATGTTACTTTATATCATCTTTAGTTTTATAGTCAGTCTCTGAAGTAGGTATTACCGTCCTCACTTTATGTTTGAAAACCTGAAGTTAAAAGAtgttcag
JW-1202-85818-SIntR-rc          ~~~~~~~~~~~~~~~~~~~~~~~~~~~~~~~~~~~~~~~~~~~~~~~~~~~~~~~~~~~~~~~~~~~~~~~~TGAAAACCTGAAGTTAAAAGATGTTCAG
JW-1202-85818-R

                                        610       620       630       640       650       660       670       680       690       700
                                ....|....|....|....|....|....|....|....|....|....|....|....|....|....|....|....|....|....|....|....|
Aotus_1202-rc [Anan_2.0]        ggccgggcgcggtggctcaagcctgtaatcccagcactttgggaggccgaggcgggtggatcacgagttcaagagatcgagaccatcctggtcaacatgg
JW-1202-85818-SIntR-rc          GGCCGGGCGCGGTGGCTCAAGCCTGTAATCCCAGCACTTTGGGAGGCCGAGGCGGGTGGATCACGAGTTCAAGAGATCGAGACCATCCTGGTCAACATGG
JW-1202-85818-R

                                        710       720       730       740       750       760       770       780       790       800
                                ....|....|....|....|....|....|....|....|....|....|....|....|....|....|....|....|....|....|....|....|
Aotus_1202-rc [Anan_2.0]        tgaaaccccgtctctactaatacaaaaaattagctgggcatggtggcgcgtgcctgtaatcccagctactcaggaggctgaggcaggagaattgcctgaa
JW-1202-85818-SIntR-rc          TGAAACCCCGTCTCTACTAATACAAAAAATTAGCTGGGCATGGTGGCGCGTGCCTGTAATCCCAGCTACTCAGGAGGCTGAGGC
JW-1202-85818-R

                                        810       820       830       840       850       860       870       880       890       900
                                ....|....|....|....|....|....|....|....|....|....|....|....|....|....|....|....|....|....|....|....|
Aotus_1202-rc [Anan_2.0]        cccaggaggcggaggttgcggtgagccgagatcgcgccattgcactccagcctgggtaacaagaacaaaactctgtctcaaaaaaaaaaaaaaaaaaaaa
JW-1202-85818-SIntR-rc
JW-1202-85818-R

                                        910       920       930       940       950       960       970       980       990      1000
                                ....|....|....|....|....|....|....|....|....|....|....|....|....|....|....|....|....|....|....|....|
Aotus_1202-rc [Anan_2.0]        aagatgttcaggTAACTAGGCCAAAATCACACGTCTGGTCTAAGTAG--TCAGATTTGAACACAGGTTGACCTGATTCCAAAATCTATACTGTATTCATT
JW-1202-85818-SIntR-rc
JW-1202-85818-R
```

Figure S10. Sequence alignment for *Alu* locus *Aotus*_1202_018500372.1:1331491-1332992 (JW-1202). PCR-based genotypes indicate the target *Alu* insertion is polymorphic among *A. nancymaae* individuals, also homozygous present in all three *A. azarae* individuals, while being absent from other *Aotus* species. DNA sequencing was limited but the SIntR sequence trace from *A. azarae* 85818 shows it shares the target *Alu* insertion in the [Anan_2.0] reference genome. The target insertion starts at position 601 (grey highlight) and is flanked by TSDs AAAAGATGTTCAG in yellow highlight.

```
                                510       520       530       540       550       560       570       580       590       600
                           ....|....|....|....|....|....|....|....|....|....|....|....|....|....|....|....|....|....|....|....|
Aotus_1203-rc [Anan_2.0]   AGTTTCTGTTCCATGAAGTGAGGATGAAAATAATACCTATCCTGCATATACCTCACAGTTATGCtctgaagaacaaataaaatctgtaaataaaagtatt
JW-1203-85818-R            AGTTTCTGTTCCATGAAGTGAGGATGAAAATAATACCTATCCTGCATATACCTCACAGTTATGCTCTGAAGAACAAATAAAATCTGTAAATAAAAGTATT
JW-1203-85818-IntF         ~~~~~~~~~~~~~~~~~~~~~~~~~~~~~~~~~~~~~~~~~~~~~~~~~~~~~~~~~~~~~~~~~~~~~~~~~~~~~~~~~~~~~~~~~~~~~~~~~~~~
JW-1203-85818-SIntR-rc     AGTTTCTGTTCCATGAAGTGAGGATGAAAATAATACCTATCCTGCATATACCTCACAGTTATGCTCTGAAGAACAAATAAAATCTGTAAATAAAAGTATT
JW-1203-85818-F-rc         ~~~~~~~~~~~~~~~~~~~~~~~~~~~~~~~~~~~~~~~~~~~~~~~~~~~~~~~~~~~~~~~~~~~~~~~~~~~~~~~~~~~~~~~~~~~~~~~~~~~~

                                610       620       630       640       650       660       670       680       690       700
                           ....|....|....|....|....|....|....|....|....|....|....|....|....|....|....|....|....|....|....|
Aotus_1203-rc [Anan_2.0]   tgcggccgggcacggtggctcaagcctgtaatcccagcactttgggaggccgaggcgggtggatcacgaggtcaagagatcgagaccatcctggtcaaca
JW-1203-85818-R            TGCGGCCGGGCGCGGTGTCTCGAGCCTGTAATCCCAGCACTTTGGGAGGCCGAGGCGGGTGGATCACGAGGTCAAGAGATCGAGACCATCCTGGTCAACA
JW-1203-85818-IntF         ~~~~~~~~~~~~~~~~~~~~~~~~~~~~~~~~~~~~~~~~~~~~~~~~~~~~~~~~~~~~~~~~~~~GAGGTCAAGAGATCGAGACCATCCTGGTCAACA
JW-1203-85818-SIntR-rc     TGCGGCCGGGCGCGGTGTCTCGAGCCTGTAATCCCAGCACTTTGGGAGGCCGAGGCGGGTGGATCACGAGGTCAAGAGATCGAGACCATCCTGGTCAACA
JW-1203-85818-F-rc         ~~~~~~~~~~~~~~~~~~~~~~~~~~~~~~~~~~~~~~~~~~~~~~~~~~~~~~~~~~~~~~~~~~~~~~~~~~~~~~~~~~~~~~~~~~~~~~~~~~~~

                                710       720       730       740       750       760       770       780       790       800
                           ....|....|....|....|....|....|....|....|....|....|....|....|....|....|....|....|....|....|....|
Aotus_1203-rc [Anan_2.0]   tggtgaaaccccgtctctactaatacaaaaaattagctgggcatggtggcgcatgcctataatcccagctgctcaggaggctgaggcaggagaattgcct
JW-1203-85818-R            TGGTGAAACCCCGTCTCTACTAATACAAAAAATTAGCTGGGCATGGTGGCGCATGCCTATAATCCCAGCTACTCAGGAGGCTGAGGCAGGAGAATTGCCT
JW-1203-85818-IntF         TGGTGAAACCCCGTCTCTACTAATACAAAAAATTAGCTGGGCATGGTGGCGCATGCCTATAATCCCAGCTACTCAGGAGGCTGAGGCAGGAGAATTGCCT
JW-1203-85818-SIntR-rc     TGGTGAAACCCCGTCTCTACTAATACAAAAAATTAGCTGGGCATGGTGGCGCATGCCTATAATCCCTAGCTACTCAGGAGG
JW-1203-85818-F-rc         ~~~~~~~~~~~~~~~~~~~~~~~~~~~~~~~~~~~~~~~~~~~~~~~~~~~~~~~~~~~~~~~~~~~~~~~~~~~~~~~~~~~~~~~~~~~~~~~~~~~~

                                810       820       830       840       850       860       870       880       890       900
                           ....|....|....|....|....|....|....|....|....|....|....|....|....|....|....|....|....|....|....|
Aotus_1203-rc [Anan_2.0]   gaacccaggaggcggaggttgcggtgagccgagatcgcgccattgcactccagcctgggtaacaagagcgaaactccgtctcaaaaaaaaaaaaaa~~~~
JW-1203-85818-R            GAACCCAGGAGGCGGAGGTTGCGGTGAGCCGAGATCGCGCCATTGCACTCCAGCCTGGGTAACAAGAGCGAAACTCCGTCTCAAAAAAAAAAAAAAAAAA
JW-1203-85818-IntF         GAACCCAGGAGGCGGAGGTTGCGGTGAGCCGAGATCGCGCCATTGCACTCCAGCCTGGGTAACAAGAGCGAAACTCCGTCTCAAAAAAAAAAAAAAAAAA
JW-1203-85818-SIntR-rc
JW-1203-85818-F-rc         ~~~~~~~~~~~~~~~~~~~~~~~~~~~~~~~~~~~~~~~~~~~~~~~~~~~~~~~~~~~~~~~~~~~~~~~~~~~~~~~~~~AAAAAAAAAAAAAAAAAA

                                910       920       930       940       950       960       970       980       990      1000
                           ....|....|....|....|....|....|....|....|....|....|....|....|....|....|....|....|....|....|....|
Aotus_1203-rc [Anan_2.0]   ~~~gtatttgcatatttcaaaaccCCATACAAGTCTAGGGGATTAGTATGTAAGTATTTGTGTAATATTAATGCTTGCTTTCCTGACTTGAAatgatagc
JW-1203-85818-R            AAAGTTTTTGC
JW-1203-85818-IntF         AAA
JW-1203-85818-SIntR-rc
JW-1203-85818-F-rc         AAAGTATTTGCATATTTCAAAACCCCATACAAGTCTAGGGGATTAGTATGTAAGTATTTGTGTAATATTAATGCTTGCTTTCCTGACTTGAAATGATAG
```

Figure S11. Sequence alignment for *Alu* locus *Aotus*_1203_018500372.1:1581468-1582960 (JW-1203). PCR-based genotypes indicate the target *Alu* insertion is polymorphic among *A. nancymaae* individuals, also present in two of three *A. azarae* individuals, while being absent from other *Aotus* species. The *Aotus* reference genome [Anan_2.0] is shown at the top followed by *A. azarae* 85818 who shares the target *Alu* insertion that starts at position 604 (grey highlight) and flanked by TSDs in yellow highlight.

```
                                     510       520       530       540       550       560       570       580       590       600
                              ....|....|....|....|....|....|....|....|....|....|....|....|....|....|....|....|....|....|....|....|
Aotus_1276-rc [Anan_2.0]      AAGACCTAGACTTTTTTCTGGCAAACCTTTTCAGACCAAGTAGTGGAATGGTCAGTCTTCTTGGGTTTGGGGAACATGGCGCAATCAAGACTGGtttcaa
JW-1276-85818-SIntR-rc        AAGACCTAGACTTTTTTCTGGTAAACCTTTTCAGACCAAGTAGTGGAATGGTCAGTCTTCTTGGGTTTGGGGAACATGGAGCAATCAAGATTGGTTTCAA
JW-1276-85818-R               AAGACCTAGACTTTTTTCTGGTAAACCTTTTCAGACCAAGTAGTGGAATGGTCAGTCTTCTTGGGTTTGGGGAACATGGAGCAATCAAGATTGGTTTCAA
JW-1276-85818-IntF            ~~~~~~~~~~~~~~~~~~~~~~~~~~~~~~~~~~~~~~~~~~~~~~~~~~~~~~~~~~~~~~~~~~~~~~~~~~~~~~~~~~~~~~~~~~~~~~~~~~~~

                                     610       620       630       640       650       660       670       680       690       700
                              ....|....|....|....|....|....|....|....|....|....|....|....|....|....|....|....|....|....|....|....|
Aotus_1276-rc [Anan_2.0]      ggccgggcgcggtggctcaagcctgtaatcccagcactttgggaggccgaggcgggtggatcacgaggtcaagagatcgagaccatcctggtcatcatgg
JW-1276-85818-SIntR-rc        GGCTGGGCGCGGTGGCTCAAGCCTGTAATCCCAGCACTTTGGGAGGCCGAGGCGGGTGGATCACGAGGTCAAGAGATCGAGACCATCCTGGTCAACATGG
JW-1276-85818-R               GGCTGGGCGCGGTGGCTCAAGCCTGTAATCCCAGCACTTTGGGAGGCCGAGGCGGGTGGATCACGAGGTCAAGAGATCGAGACCATCCTGGTCAACATGG
JW-1276-85818-IntF            ~~~~~~~~~~~~~~~~~~~~~~~~~~~~~~~~~~~~~~~~~~~~~~~~~~~~~~~~~~~~~~~~GAGGTCAAGAGATCGAGACCATCCTGGTCAACATGG

                                     710       720       730       740       750       760       770       780       790       800
                              ....|....|....|....|....|....|....|....|....|....|....|....|....|....|....|....|....|....|....|....|
Aotus_1276-rc [Anan_2.0]      tgaaaccccgtctctactaatacaaaaaattagctgggcatggtggcgcgtgcctgtaatcccagctactcaggaggctgaggcaggagaattgcctgaa
JW-1276-85818-SIntR-rc        TGAAACCCCGTCTCTACTAATACAAAAAATTAGCTGGGCATGGTGGCGCGTGCCTGTAATCCCAGCTACTCAGGAGG
JW-1276-85818-R               TGAAACCCCGTCTCTACTAATACAAAAAATTAGCTGGGCATGGTGGCGCGTGCCTGTAATCCCAGCTACTCAGGAGGCTGAGGCAGGAGAATTGCCTGAA
JW-1276-85818-IntF            TGAAACCCCGTCTCTACTAATACAAAAAATTAGCTGGGCATGGTGGCGCGTGCCTGTAATCCCAGCTACTCAGGAGGCTGAGGCAGGAGAATTGCCTGAA

                                     810       820       830       840       850       860       870       880       890       900
                              ....|....|....|....|....|....|....|....|....|....|....|....|....|....|....|....|....|....|....|....|
Aotus_1276-rc [Anan_2.0]      cccaggaggcggaggttgcggtgagccaagatcgtgccattgcactccagcctgggtatcaagagcgaaactccgtctcaaaaacaacaacaacaaaaaa
JW-1276-85818-SIntR-rc
JW-1276-85818-R               CCCAGGAGGCGGAGGTTGCGGTGAGCTGAGATCGTGCCATTGCACTCCAGCCTGGGTATCAAGAGCGAAACTCCGTCTCAAAAAAAAAAAAAAAAAAAAA
JW-1276-85818-IntF            CCCAGGAGGCGGAGGTTGCGGTGAGCTGAGATCGTGCCATTGCACTCCAGCCTGGGTATCAAGAGCGAAACTCCGTCTCAAAAAAAAAAAAAAAAAAAAA

                                     910       920       930       940       950       960       970       980       990      1000
                              ....|....|....|....|....|....|....|....|....|....|....|....|....|....|....|....|....|....|....|....|
Aotus_1276-rc [Anan_2.0]      gactggTTTCAGCCTATCGAGTAGATGATGGTGCTGATTTATGGTTGAgggaatgttttttgtttgttttttgagatgg---------------------
JW-1276-85818-SIntR-rc
JW-1276-85818-R               AA
JW-1276-85818-IntF            AA
```

Figure S12. Sequence alignment for *Alu* locus *Aotus*_1276_018501266.1:1161971-1163470 (JW-1276). PCR-based genotypes indicate the target *Alu* insertion is homozygous present in all *A. nancymaae* and *A. azarae* individuals, while being absent from other *Aotus* species. The *Aotus* reference genome [Anan_2.0] is shown at the top followed by *A. azarae* 85818 who appears to share the

target insertion starting at position 601 (grey highlight) and flanked by TSDs in yellow highlight. However, no 3’ flanking sequence trace was successfully obtained, therefore it remains a remote possibility that this is a precise parallel insertion based solely on 5’ matching flanking sequence. We will consider this sequence as evidence of a shared insertion.

```
                                     510       520       530       540       550       560       570       580       590       600
                             ....|....|....|....|....|....|....|....|....|....|....|....|....|....|....|....|....|....|....|....|
Aotus_1507-rc [Anan_2.0]     atccTCCTGTTAGAGTAGGAGAACGATTAAATATACTATAGATATTGAAGAAATGGTACCATATTCAATTACAGCGCCAGCGTGTTAAGTTCCATttctc
JW-1507-85818-SIntR-rc       ATCCTCCTGTTAGAGTAGGAGAACGATTAAATATACTATAGATATTGAAGAAATCGTACCATATTCAATTACAGCGCCAGCGTGTTAAGTTCCATTTCTC
JW-1507-85818-R              ATCCTCCTGTTAGAGTAGGAGAACGATTAAATATACTATAGATATTGAAGAAATCGTACCATATTCAATTACAGCGCCAGCGTGTTAAGTTCCATTTCTC
JW-1507-85818-IntF           ~~~~~~~~~~~~~~~~~~~~~~~~~~~~~~~~~~~~~~~~~~~~~~~~~~~~~~~~~~~~~~~~~~~~~~~~~~~~~~~~~~~~~~~~~~~~~~~~~~~~

                                     610       620       630       640       650       660       670       680       690       700
                             ....|....|....|....|....|....|....|....|....|....|....|....|....|....|....|....|....|....|....|....|
Aotus_1507-rc [Anan_2.0]     ggccaggcgcggtggctcaagcctgtaatcccagcactttgggagaccgaggcgggtggatcacgaggtcgagagatcgagaccatcctggtcaacattg
JW-1507-85818-SIntR-rc       GGCCAGGCGCGGTGGCTCAAGCCTGTAATCCCAGCACTTTGGGAGACCGAGGCGGGTGGATCACGAGGTCGAGAGATCGAGACCATCCTGGTCAACATTG
JW-1507-85818-R              GGCCAGGCGCGGTGGCTCAAGCCTGTAATCCCAGCACTTTGGGAGACCGAGGCGGGTGGATCACGAGGTCGAGAGATCGAGACCATCCTGGTCAACATTG
JW-1507-85818-IntF           ~~~~~~~~~~~~~~~~~~~~~~~~~~~~~~~~~~~~~~~~~~~~~~~~~~~~~~~~~~~~~~~~GAGGTCGAGAGATCGAGACCATCCTGGTCAACATTG

                                     710       720       730       740       750       760       770       780       790       800
                             ....|....|....|....|....|....|....|....|....|....|....|....|....|....|....|....|....|....|....|....|
Aotus_1507-rc [Anan_2.0]     tgaaaccccgtctctactaatacaaaaaaattagctgggcacggtggcacgtgcctgtaatcccagctactcaggaggctgaggcaggagaattgcctga
JW-1507-85818-SIntR-rc       TGAAACCCCGTCTCTACTAATACAAAAAAATTAGCTGGGCACGGTGGCACGTGCCTGTAATCCCAACTACTCAGGAGG
JW-1507-85818-R              TGAAACCCCGTCTCTACTAATACAAAAAAATTAGCTGGGCACGGTGGCACGTGCCTGTAATCCCAACTACTCAGGAGGCTGAGGCAGGAGAATTGCCTGA
JW-1507-85818-IntF           TGAAACCCCGTCTCTACTAATACAAAAAAATTAGCTGGGCACGGTGGCACGTGCCTGTAATCCCAACTACTCAGGAGGCTGAGGCAGGAGAATTGCCTGA

                                     810       820       830       840       850       860       870       880       890       900
                             ....|....|....|....|....|....|....|....|....|....|....|....|....|....|....|....|....|....|....|....|
Aotus_1507-rc [Anan_2.0]     acccaggaggcggaggttgcggtgagccgagatcgcgccattgcactccagcctgggtaacaagagcgagactccgtctcaaaaaaaaaaaaaaaaaaaa
JW-1507-85818-SIntR-rc
JW-1507-85818-R              ACCCAGGAGGCGGAGGTTGCGGTGAGCCGAGATCGCGCCATTGCACTCCAGCCTGGGTAACAAGAGCGAGACTCCGTCTCAAAAAAAAAAAAAAAAAA
JW-1507-85818-IntF           ACCCAGGAGGCGGAGGTTGCGGTGAGCCGAGATCGCGCCATTGCACTCCAGCCTGGGTAACAAGAGCGAGACTCCGTCTCAAAAAAAAAAAAAAAAAA

                                     910       920       930       940       950       960       970       980       990      1000
                             ....|....|....|....|....|....|....|....|....|....|....|....|....|....|....|....|....|....|....|....|
Aotus_1507-rc [Anan_2.0]     aagttccatttCTCACTGTGTCTGAAGTTGTGCAGTTGGGTTTCTGCCATATTAGTGTGGGTGGGAAACTCCTTGATTAAATGTCCTCTTAGGTGAAATG
JW-1507-85818-SIntR-rc
JW-1507-85818-R
JW-1507-85818-IntF
```

Figure S13. Sequence alignment for *Alu* locus *Aotus*_1507_018505863.1:9051781-9053282 (JW-1507). PCR-based genotypes indicate the target *Alu* insertion is present in all *A. nancymaae* and *A. azarae* individuals, while being absent from other *Aotus* species. The *Aotus* reference genome [Anan_2.0] is shown at the top followed by *A. azarae* 85818 who appears to share the target insertion starting at position 601 (grey highlight) and flanked by TSDs in yellow highlight. However, no 3’ flanking sequence trace was successfully obtained, therefore it remains a remote possibility that this is a precise parallel insertion based solely on 5’ matching flanking sequence. We will consider this sequence as evidence of a shared insertion.

```
                                       510       520       530       540       550       560       570       580       590       600
                              ....|....|....|....|....|....|....|....|....|....|....|....|....|....|....|....|....|....|....|....|
Aotus_1528 [Anan_2.0]         AGGTACTCTTGGTCACAAGATGTTTGTGGTTAAGGAAACAAGTTAATGGTGCTAACTAATTAAGTAAGACTCAGAACTTATGGAAGTGTCCCaataaaga
JW-1528-85818-F               AGGTACTCTTGGTCACAAGATGTTTGTGGTTAAGGAAACAAGTTAATGGTGCTAACTAATTAAGTAAGACTCAGAACTTATGGAAGTGTCCCAATAAAGA
JW-1528-85818-SIntR-rc        AGGTACTCTTGGTCACAAGATGTTTGTGGTTAAGGAAACAAGTTAATGGTGCTAACTAATTAAGTAAGACTCAGAACTTATGGAAGTGTCCCAATAAAGA
JW-1528-85818-IntF            ~~~~~~~~~~~~~~~~~~~~~~~~~~~~~~~~~~~~~~~~~~~~~~~~~~~~~~~~~~~~~~~~~~~~~~~~~~~~~~~~~~~~~~~~~~~~~~~~~~~~
JW-1528-85818-R-rc            ~~~~~~~~~~~~~~~~~~~~~~~~~~~~~~~~~~~~~~~~~~~~~~~~~~~~~~~~~~~~~~~~~~~~~~~~~~~~~~~~~~~~~~~~~~~~~~~~~~~~

                                       610       620       630       640       650       660       670       680       690       700
                              ....|....|....|....|....|....|....|....|....|....|....|....|....|....|....|....|....|....|....|....|
Aotus_1528 [Anan_2.0]         aaaagcatccggccgggcgcggtggctcaagcctgtaatcccagcactttgggaggccgaggcgggtggatcacgaggtcaagagatcgagaccatcctg
JW-1528-85818-F               AAAAGCATCCGGCCGGGCGCAGTGGCTCACGCCTGCAATCCCAGCACTTTGGGAGGCCGAGGCGGGTGGATCACGAGGTCGAGAGATTGAGACCATCCTG
JW-1528-85818-SIntR-rc        AAAAGCATCCGGCCGGGCGCAGTGGCTCACGCCTGCAATCCCAGCACTTTGGGAGGCCGAGGCGGGTGGATCACGAGGTCGAGAGATTGAGACCATCCTG
JW-1528-85818-IntF            ~~~~~~~~~~~~~~~~~~~~~~~~~~~~~~~~~~~~~~~~~~~~~~~~~~~~~~~~~~~~~~~~~~~~~~~~~~GAGGTCGAGAGATTGAGACCATCCTG
JW-1528-85818-R-rc            ~~~~~~~~~~~~~~~~~~~~~~~~~~~~~~~~~~~~~~~~~~~~~~~~~~~~~~~~~~~~~~~~~~~~~~~~~~~~~~~~~~~~~~~~~~~~~~~~~~~~

                                       710       720       730       740       750       760       770       780       790       800
                              ....|....|....|....|....|....|....|....|....|....|....|....|....|....|....|....|....|....|....|....|
Aotus_1528 [Anan_2.0]         gtcaacattgtgaaaccccgtctctactgatacaaaaaaattagctgggcacggtggcacgtgcctgtaatcccagctactcaggaggctgaggcaggag
JW-1528-85818-F               GTCAACATTGTGAAACCCCGTCTCTACTGATACAAAAATATTAGCTGGGCACGGTGGCGCGTGCCTGTAATCCCAGCTACTCAGGAGGCTGAGGCAGGAG
JW-1528-85818-SIntR-rc        GTCAACATTGTGAAACCCCGTCTCTACTGATACAAAAATATTAGCTGGGCACGGTGGCGCGTGCC
JW-1528-85818-IntF            GTCAACATTGTGAAACCCCGTCTCTACTGATACAAAAATATTAGCTGGGCACGGTGGCGCGTGCCTGTAATCCCAGCTACTCAGGAGGCTGAGGCAGGAG
JW-1528-85818-R-rc            ~~~~~~~~~~~~~~~~~~~~~~~~~~~~~~~~~~~~~~~~~~~~~~~~~~~~~~~~~~~~~~~~~~~~~~~~~~~~~~~~~~~~~~~~~~~~~~~~~~~~

                                       810       820       830       840       850       860       870       880       890       900
                              ....|....|....|....|....|....|....|....|....|....|....|....|....|....|....|....|....|....|....|....|
Aotus_1528 [Anan_2.0]         aattgcctgaacccaggaggcggaggttgcggtgagctgagatcgcgccattgcactccagcctgggtaacaagaacgagactccgtctcaaaaaaaaaa
JW-1528-85818-F               AATTGCCTGAACCCAGGAGGTGGAGGTTGCGGTGAGCCGAGATCGCGCCATTGCTCTCCAGCCTGGGTAACAAGAGCGAGACTCCGTCTCAAAAAAAAAA
JW-1528-85818-SIntR-rc
JW-1528-85818-IntF            AATTGCCTGAACCCAGGAGGTGGAGGTTGCGGTGAGCCGAGATCGCGCCATTGCTCTCCAGCCTGGGTAACAAGAGCGAGACTCCGTCTCAAAAAAAAAA
JW-1528-85818-R-rc            ~~~~~~~~~~~~~~~~~~~~~~~~~~~~~~~~~~~~~~~~~~~~~~~~~~~~~~~~~~~~~~~~~~~~~~~~~~~~~~~~~~~~~~~~~~~~AAAAAAAA

                                       910       920       930       940       950       960       970       980       990      1000
                              ....|....|....|....|....|....|....|....|....|....|....|....|....|....|....|....|....|....|....|....|
Aotus_1528 [Anan_2.0]         aaaagaaaaagcatcctTAGTTTAAgagtttcactttaaaaataataatatactcaCAGATTCTTGCTGAAATCAATAGTTGCAGAAGAGAATAACAATG
JW-1528-85818-F               AAAAAAAA
JW-1528-85818-SIntR-rc
JW-1528-85818-IntF            AAAAAAAA
JW-1528-85818-R-rc            AAAAGAAAAAGCATCCTTAGTTTAAGAGTTTCACTTTAAAAATAATAATATACTCACAGATTCTT
```

Figure S14. Sequence alignment for *Alu* locus *Aotus*_1528_018502886.1:12506963-12508462 (JW-1528). PCR-based genotypes indicate the target *Alu* insertion is polymorphic among *A. nancymaae* individuals, homozygous present in all three *A. azarae* individuals, while being absent from other *Aotus* species. The *Aotus* reference genome [Anan_2.0] is shown at the top followed by *A. azarae* 85818 who shares the target *Alu* insertion starting at position 611 (grey highlight), with matching flanking TSDs in yellow highlight.

```
                                510       520       530       540       550       560       570       580       590       600
                        ....|....|....|....|....|....|....|....|....|....|....|....|....|....|....|....|....|....|....|....|
Aotus_1534 [Anan_2.0]   TGTTTCAGCTCTTCCTATTTTTAATGgccactaatcccagcactcagaagAATATGGCTCATACTCCATCCAcatattttcttaagaatttatggaaaag
JW-1534-85818-IntR-rc   TGTTTCAGCTCTTCCTATTTTTAATGGCCACTAATCCCAGCACTCAGAAGAATATGGCTCATACTCCATCCACATATTTTCTTAAGAATTTATGGAAAAG
JW-1534-85818-F         TGTTTCAGCTCTTCCTATTTTTAATGGCCACTAATCCCAGCACTCAGAAGAATATGGCTCATACTCCATCCACATATTTTCTTAAGAATTTATGGAAAAG
JW-1534-85818-IntF      ~~~~~~~~~~~~~~~~~~~~~~~~~~~~~~~~~~~~~~~~~~~~~~~~~~~~~~~~~~~~~~~~~~~~~~~~~~~~~~~~~~~~~~~~~~~~~~~~~~~~
JW-1534-85818-R-rc      ~~~~~~~~~~~~~~~~~~~~~~~~~~~~~~~~~~~~~~~~~~~~~~~~~~~~~~~~~~~~~~~~~~~~~~~~~~~~~~~~~~~~~~~~~~~~~~~~~~~~

                                610       620       630       640       650       660       670       680       690       700
                        ....|....|....|....|....|....|....|....|....|....|....|....|....|....|....|....|....|....|....|
Aotus_1534 [Anan_2.0]   cggccgggcgcggtggctcaagcctgtaatcccagcactttgggaggccgaggcgggtggatcacgaggtcaagagatcgagaccatcctggtcaacatg
JW-1534-85818-IntR-rc   CGGCCGGGCGCGGTGGCTCAAGCCTGTAATCCCAGCACTTTGGGAGGCCGAGGCGGGTGGATCACGAGGTCAAGAGATCGAGACCATCTTGGTCGACATG
JW-1534-85818-F         CGGCCGGGCGCGGTGGCTCAAGCCTGTAATCCCAGCACTTTGGGAGGCCGAGGCGGGTGGATCACGAGGTCAAGAGATCGAGACCATCTTGGTCGACATG
JW-1534-85818-IntF      ~~~~~~~~~~~~~~~~~~~~~~~~~~~~~~~~~~~~~~~~~~~~~~~~~~~~~~~~~~~~~~~~~GAGGTCAAGAGATCGAGACCATCTTGGTCGACATG
JW-1534-85818-R-rc      ~~~~~~~~~~~~~~~~~~~~~~~~~~~~~~~~~~~~~~~~~~~~~~~~~~~~~~~~~~~~~~~~~~~~~~~~~~~~~~~~~~~~~~~~~~~~~~~~~~~~

                                710       720       730       740       750       760       770       780       790       800
                        ....|....|....|....|....|....|....|....|....|....|....|....|....|....|....|....|....|....|....|
Aotus_1534 [Anan_2.0]   gtgaaaccccgtctctactaatacaaaaaattagctgggcatggtggcgcgtgcctgtaatcccagctactcaggaggctgaggcaggagaattgcctga
JW-1534-85818-IntR-rc   GTGAAACCCCGTCTCTACAAATACAAAAAATTAGCTGGGCATGGTAGCGCGTGCCTGTAGTCCCAGCTACTCAGGAGG
JW-1534-85818-F         GTGAAACCCCGTCTCTACTAATA
JW-1534-85818-IntF      GTGAAACCCCGTCTCTACAAATACAAAAAATTAGCTGGGCATGGTAGCGCGTGCCTGTAGTCCCAGCTACTCAGGAGGCTGAGGCAGGAGAATTGCCTGA
JW-1534-85818-R-rc      ~~~~~~~~~~~~~~~~~~~~~~~~~~~~~~~~~~~~~~~~~~~~~~~~~~~~~~~~~~~~~~~~~~~~~~~~~~~~~~~~~~~~~~~~~~~~~~~~~~~~

                                810       820       830       840       850       860       870       880       890       900
                        ....|....|....|....|....|....|....|....|....|....|....|....|....|....|....|....|....|....|....|
Aotus_1534 [Anan_2.0]   acccaggaggcggaggttgcggtgagccgagatcgcgccattgcactccagcctgggtaacgagcgaaactccgtctcaaaaaaaaaaaaaaaaaaaaaa
JW-1534-85818-IntR-rc
JW-1534-85818-F
JW-1534-85818-IntF      ACCCAGGAGGCGGAGGTTGCGGTGAGCCGAGATCGCGCCTTTGCACTCCAGCCTGGGTAACGAGCGAAACTCCGTCTCAAAAAAAAAAAAAAAAAAA
JW-1534-85818-R-rc      ~~~~~~~~~~~~~~~~~~~~~~~~~~~~~~~~~~~~~~~~~~~~~~~~~~~~~~~~~~~~~~~~~~~~~~~~~~~~~~~~~~~~~AAAAAAAAAAAAAAA

                                910       920       930       940       950       960       970       980       990      1000
                        ....|....|....|....|....|....|....|....|....|....|....|....|....|....|....|....|....|....|....|
Aotus_1534 [Anan_2.0]   aagaatttatggaAAAGCACTGTCTTTATCTGTATTTGAATAGTCCAAGGCTACTAAAGGACCCCAGTTGTAATCATTCATGCTCTATCCAAATCTAAAT
JW-1534-85818-IntR-rc
JW-1534-85818-F
JW-1534-85818-IntF
JW-1534-85818-R-rc      AAGAATTTANGGAAAAGCACTG
```

Figure S15. Sequence alignment for *Alu* locus *Aotus*_1534_018505554.1:7580189-7581692 (JW-1534). PCR-based genotypes indicate the target *Alu* insertion is polymorphic among *A. nancymaae* individuals, homozygous present in all three *A. azarae* individuals, while being absent from other *Aotus* species. The *Aotus* reference genome [Anan_2.0] is shown at the top followed by *A. azarae* 85818 who shares the target *Alu* insertion starting at position 602 (grey highlight), with matching flanking TSDs in yellow highlight.

```
                                        510       520       530       540       550       560       570       580       590       600
                                ....|....|....|....|....|....|....|....|....|....|....|....|....|....|....|....|....|....|....|....|
Aotus_1559 [Anan_2.0]           CTATACCCAGCCCCTACAggtacacattcacacacatatgcacacattttaGGTTGAGCTACAGGAGATCACTGTTTTTGCAAGTAAAAAGTAatttcag
JW-1559V2-KB10043-SIntR-rc      ~~~~~~~~~~CCCCTACAGGTACACATTCACACACATATGCACACATTTTAGGTTGAGCTACAGGAGATCACTGTTTTTGCAAGTAAAAAGTAATTTCAG
JW-1559V2-KB10043-F             ~~~~~~~~~~~~~~~~~~~~~~~~~~~~~~~~~~~~~~~~~~~~~~~~~~~~~~~~~~~~~~~~~~~~TCACTGTTTTTGCAAGTAAAAAGTAATTTCAG
JW-1559V2-KB10043-IntF          ~~~~~~~~~~~~~~~~~~~~~~~~~~~~~~~~~~~~~~~~~~~~~~~~~~~~~~~~~~~~~~~~~~~~~~~~~~~~~~~~~~~~~~~~~~~~~~~~~~~
JW-1559V2-KB10043-R-rc          ~~~~~~~~~~~~~~~~~~~~~~~~~~~~~~~~~~~~~~~~~~~~~~~~~~~~~~~~~~~~~~~~~~~~~~~~~~~~~~~~~~~~~~~~~~~~~~~~~~~
JW-1559V2-85457-F               ~~~~~~~~~~~~~~~~~~~~~~~~~~~~~~~~~~~~~~~~~~~~~~~~~~~~~~~~~~~~~~~GGAGATCACTGTTTTTGCAAGTAAAAAGTAATTTCAG
JW-1559V2-85457-IntF            ~~~~~~~~~~~~~~~~~~~~~~~~~~~~~~~~~~~~~~~~~~~~~~~~~~~~~~~~~~~~~~~~~~~~~~~~~~~~~~~~~~~~~~~~~~~~~~~~~~~
JW-1559V2-85457-SIntR-rc        ~~~~~~~~~~~~~~~~~~~~~~~~~~~~~~~~~~~~~~~~~~~~~~~~~~~~~~~~~~~~~~~~~~~~~~~~~~~~~~~~~~~~~~~~~~~~~~~~~~~
JW-1559V2-85457-R-rc            ~~~~~~~~~~~~~~~~~~~~~~~~~~~~~~~~~~~~~~~~~~~~~~~~~~~~~~~~~~~~~~~~~~~~~~~~~~~~~~~~~~~~~~~~~~~~~~~~~~~
JW-1559V2-85818-F               ~~~~~~~~~~~~~~~~~~~~~~~~~~~~~~~~~~~~~~~~~~~~~~~~~~~~~~~GAGCTACAGGAGANCACTGTTTTTGCAAGTAAAAAGTAATTTCAG
JW-1559V2-85818-IntF            ~~~~~~~~~~~~~~~~~~~~~~~~~~~~~~~~~~~~~~~~~~~~~~~~~~~~~~~~~~~~~~~~~~~~~~~~~~~~~~~~~~~~~~~~~~~~~~~~~~~
JW-1559V2-85818-SIntR-rc        ~~~~~~~~~~~~~~~~~~~~~~~~~~~~~~~~~~~~~~~~~~~~~~~~~~~~~~~~~~~~~~~~~~~~~~~~~~~~~~~~~~~~~~~~~~~~~~~~~~~
JW-1559V2-85818-R-rc            ~~~~~~~~~~~~~~~~~~~~~~~~~~~~~~~~~~~~~~~~~~~~~~~~~~~~~~~~~~~~~~~~~~~~~~~~~~~~~~~~~~~~~~~~~~~~~~~~~~~

                                        610       620       630       640       650       660       670       680       690       700
                                ....|....|....|....|....|....|....|....|....|....|....|....|....|....|....|....|....|....|....|....|
Aotus_1559 [Anan_2.0]           ggccgggcgcggtggctcaagcctgtaatcccagcactttgggaggccgaggcgggtggatcacgaggtcaagagatcgagaccatcctggtcaacatgg
JW-1559V2-KB10043-SIntR-rc      GGCCGGGCGCGGTGGCTCAAGCCTGTAATCCCAGCACTTTGGGAGGCCGAGGCGGGTGGATCACGAGGTCAAGAGATCGAGACCATCCTGGTCAACATGG
JW-1559V2-KB10043-F             GGCCGGGCGCGGTGGCTCAAGCCTGTAATCCCAGCACTTTGGGAGGCCGAGGCGGGTGGATCACGAGGTCAAGAGATCGAGACCATCCTGGTCAACATGG
JW-1559V2-KB10043-IntF          ~~~~~~~~~~~~~~~~~~~~~~~~~~~~~~~~~~~~~~~~~~~~~~~~~~~~~~~~~~~~~~~~GAGGTCAAGAGATCGAGACCATCCTGGTCAACATGG
JW-1559V2-KB10043-R-rc          ~~~~~~~~~~~~~~~~~~~~~~~~~~~~~~~~~~~~~~~~~~~~~~~~~~~~~~~~~~~~~~~~~~~~~~~~~~~~~~~~~~~~~~~~~~~~~~~~~~~
JW-1559V2-85457-F               GGCCGGGCGCGGTGNCTCAAGCCTGTAATCCCAGCACTTTGGGAGGCCGAGGCGGGTGGATCACGAGGTCAAGAGATCGAGACCATCCTGGTCAACATGG
JW-1559V2-85457-IntF            ~~~~~~~~~~~~~~~~~~~~~~~~~~~~~~~~~~~~~~~~~~~~~~~~~~~~~~~~~~~~~~~~GAGGTCAAGAGATCGAGACCATCCTGGTCAACATGG
JW-1559V2-85457-SIntR-rc        ~~~~~~~~~~~~~~~~~~~~~~~~~~~~~~~~~~~~~~~~~~~~~~~~~~~~~~~~~~~~~~~~~~~~~~~~~~~~~~~~~~~~~~~~~~~~~~~~~~~
JW-1559V2-85457-R-rc            ~~~~~~~~~~~~~~~~~~~~~~~~~~~~~~~~~~~~~~~~~~~~~~~~~~~~~~~~~~~~~~~~~~~~~~~~~~~~~~~~~~~~~~~~~~~~~~~~~~~
JW-1559V2-85818-F               GGCCGGGCGCGGTGGCTCAAGCCTGTAATCCCAGCACTTTGGGAGGCCGAGGCGGGTGGATCACGAGGTCAAGAGATCGAGACCATCCTGGTCAACATGG
JW-1559V2-85818-IntF            ~~~~~~~~~~~~~~~~~~~~~~~~~~~~~~~~~~~~~~~~~~~~~~~~~~~~~~~~~~~~~~~~GAGGTCAAGAGATCGAGACCATCCTGGTCAACATGG
JW-1559V2-85818-SIntR-rc        ~~~~~~~~~~~~~~~~~~~~~~~~~~~~~~~~~~~~~~~~~~~~~~~~~~~~~~~~~~~~~~~~~~~~~~~~~~~~~~~~~~~~~~~~~~~~~~~~~~~
JW-1559V2-85818-R-rc            ~~~~~~~~~~~~~~~~~~~~~~~~~~~~~~~~~~~~~~~~~~~~~~~~~~~~~~~~~~~~~~~~~~~~~~~~~~~~~~~~~~~~~~~~~~~~~~~~~~~

                                        710       720       730       740       750       760       770       780       790       800
                                ....|....|....|....|....|....|....|....|....|....|....|....|....|....|....|....|....|....|....|....|
Aotus_1559 [Anan_2.0]           tgaaaccccgtctctactaatacaaaaaaaaaacactagctgggcatggtggcgcgtgcctgtaatcccagctactcaggaggctgaggcaggggaattg
JW-1559V2-KB10043-SIntR-rc      TGAAACCCCGTCTCTACTAATACAAAAAAAAA~CACTAGCTGGGCATGGTGGCGCGTGCCTGTANTCCCAGCTACTCAGGAGG
JW-1559V2-KB10043-F             TGAAACCCCGTCTCTACTAATACAAAAAAAAA~CACTAGCTGGGCATGGTGGCGCGTGCCTGTAATCCCAGCTACTCAGGAGGCTGAGGCAGGAGAATTG
JW-1559V2-KB10043-IntF          TGAAACCCCGTCTCTACTAATACAAAAAAAAA~CACTAGCTGGGCATGGTGGCGCGTGCCTGTAATCCCAGCTACTCAGGAGGCTGAGGCAGGAGAATTG
JW-1559V2-KB10043-R-rc          ~~~~~~~~~~~~~~~~~~~~~~~~~~~~~~~~~~~~~~~~~~~~~~~~~~~~~~~~~~~~~~~~~~~~~~~~~~~~~~~~~~~~~~~~~~~~~~~~~~~
JW-1559V2-85457-F               TGAAACCCCGTCTCTACTAATACAAAAAAAAAAAAAAN~CTGGGCGGGGTGGCGCGTGCCTGTAATCCCACCTACTCACGAGGCTGANGCAGGGGAATTA
JW-1559V2-85457-IntF            TGAAACCCCGTCTCTACTAATACAAAAAAAAA~CACAANCTGGGCGTGGTGGCGCGTGCCTGTAATCCCACCTACTCACGAGGCTGAGGCAGGGGAATTG
JW-1559V2-85457-SIntR-rc        ~~~~~~~~~~~~~~~~~TAAAAAAAAAAAAAA~CACTAGCTGGGCATGGTGGCGCGTGCCTGTAATCCCAGCTACTCAGGAGGC
JW-1559V2-85457-R-rc            ~~~~~~~~~~~~~~~~~~~~~~~~~~~~~~~~~~~~~~~~~~~~~~~~~~~~~~~~~~~~~~~~~~~~~~~~~~~~~~~~~~~~~~~~~~~~~~~~~~~
JW-1559V2-85818-F               TGAAACCCCGTCTCTACTAATACAAAAAAAAAA~ACTAGCTGGGGGGGGGGGCGCGTGCCTGTAATCCCACCTACTCAGGAGGCTGAGGCAGGAGAATTG
JW-1559V2-85818-IntF            TGAAACCCCGTCTCTACTAATACAAAAAAAAA
JW-1559V2-85818-SIntR-rc        ~~~~~~~~~~~~~~~~~~~AAAAAAAAAAAAAACACTAGCTGGGCATGGTGGCGCGTGCCTGTANTCCCAGCTACTCAGGAGG~
JW-1559V2-85818-R-rc            ~~~~~~~~~~~~~~~~~~~~~~~~~~~~~~~~~~~~~~~~~~~~~~~~~~~~~~~~~~~~~~~~~~~~~~~~~~~~~~~~~~~~~~~~~~~~~~~~~~~
```

```
                                         810       820       830       840       850       860       870       880       890       900
                                ....|....|....|....|....|....|....|....|....|....|....|....|....|....|....|....|....|....|....|....|
Aotus_1559 [Anan_2.0]          cctgaacccaggaggcggaggttgcggtgagccgagatcgcgccattgcactccagcatgggtaacgagcgaaactccgtctcaaaaaaaaaaaaaaaaa
JW-1559V2-KB10043-SIntR-rc
JW-1559V2-KB10043-F             CCTGAACCCAGGAGGCGGAGGTTGCGGTGAGCCGAGATCGCGCCATTGCACTCCAGCCTGGGTAACGAGCGAAACTCCGTCTCAAAAAAAAAAAAAAAAA
JW-1559V2-KB10043-IntF          CCTGAACCCAGGAGGCGGAGGTTGCGGTGAGCCGAGATCGCGCCATTGCACTCCAGCCTGGGTAACGAGCGAAACTCCGTCTCAAAAAAAAAAAAAAAAA
JW-1559V2-KB10043-R-rc          ~~~~~~~~~~~~~~~~~~~~~~~~~~~~~~~~~~~~~~~~~~~~~~~~~~~~~~~~~~~~~~~~~~~~~~~~~~~~~~~~~~~~~~~~AAAAAAAAAAAA
JW-1559V2-85457-F               CCTGAACCCACGAGGCGGAGGTTGCTGTGAGCCAACATCGCTCCATTGCTCTCCACCCTGGGTAACAAGCGAAACACCNTCTCACAAAAAAAAAAAAAAA
JW-1559V2-85457-IntF            CCNGAACCCACGANGCGGAGGTTGCGGTGAGCCNANATCGCGCCATTGCACTCCACCCTGGGTAACAAGCGAAACTCCNTCTCACAAAAAAAAAAAAAAA
JW-1559V2-85457-SIntR-rc
JW-1559V2-85457-R-rc            ~~~~~~~~~~~~~~~~~~~~~~~~~~~~~~~~~~~~~~~~~~~~~~~~~~~~~~~~~~~~~~~~~~~~~~~~~~~~~~~~~~~~~~~~~~~~~AAAAAAA
JW-1559V2-85818-F               CCTGAACCCACGAGGCAGAGGTTGTGGTGAGCCAACATCGCCCCCTTGCTCTCCACCCTGGGTAACAAGCGAAACTCCNTCTCAAAAAAAAAAAAAAAAA
JW-1559V2-85818-IntF
JW-1559V2-85818-SIntR-rc
JW-1559V2-85818-R-rc            ~~~~~~~~~~~~~~~~~~~~~~~~~~~~~~~~~~~~~~~~~~~~~~~~~~~~~~~~~~~~~~~~~~~~~~~~~~~~~~~~~~~~~~~~~~AAAAAAAAAA

                                         910       920       930       940       950       960       970       980       990      1000
                                ....|....|....|....|....|....|....|....|....|....|....|....|....|....|....|....|....|....|....|
Aotus_1559 [Anan_2.0]          aaaaaaaaagtaatttcagatGGCTCAATGGAACACATTATACTGTTTGGGAGGTGTCATCAGGGtaattaagttttatttatttatttttagaaagtg
JW-1559V2-KB10043-SIntR-rc
JW-1559V2-KB10043-F             AAAAAAA
JW-1559V2-KB10043-IntF          AAAAAAA
JW-1559V2-KB10043-R-rc          AAAAAAAAAGTAATTTCAGATGGCTCAATGGAACACATTATACTGTTTGGGAGGTGTCNTCAGGGT
JW-1559V2-85457-F               AAAAAAA
JW-1559V2-85457-IntF            AAAAAAAA
JW-1559V2-85457-SIntR-rc
JW-1559V2-85457-R-rc            AAAAAAAAAGTAATTTCAGATGGCTCAATGGAANNNATTATACTGTTTGGGAGGTG
JW-1559V2-85818-F               AAA
JW-1559V2-85818-IntF
JW-1559V2-85818-SIntR-rc
JW-1559V2-85818-R-rc            AAAAAAAAAGTAATTTCAGATGGCTCAATGGAACACATTATACTGTTTGGGAGGTGTCANCAGGGTAATTAA
```

Figure S16. Sequence alignment for *Alu* locus *Aotus*_1559_V2 018503287.1:3404582-3406090 (JW-1559V2). PCR-based genotypes indicate the target *Alu* insertion is homozygous present (1/1) in all *A. nancymaae* and *A. azarae* individuals and homozygous absent (0/0) in other *Aotus* species. DNA sequencing was performed to confirm this pattern. The reference genome [Anan_2.0] is shown at the top followed by *A. azarae* samples KB10043, 85457 and 85818. The target *Alu* insertion starts at position 601 (grey highlight) and is flanked by TSDs in yellow highlight. All three *A. azarae* individuals share the target insertion.

```
                                   910       920       930       940       950       960       970       980       990      1000
                           ....|....|....|....|....|....|....|....|....|....|....|....|....|....|....|....|....|....|....|....|
Storer_2020_Aotus-827      TCCCccaaaaaaccctcaaaataggccgggcgcggtggctcacgcctgtaatccaagcactttgggaggccgaggcgggtggatcacgaggtcaagagat
JS-827-85818-SIntR-rc      TCCCCCAAAAAACCCTCAAAATAGGCCGGGCGCGGTGGCTCACGCCTGTAATCCAAGCACTTTGGGAGGCCGAGGTGGGTGGATCACGAGGTCAAGAGAT
JS-827-85818-F             ~~~~~~~~~~~~~~~~~~~~~~~~~~~~~~~GCGGTGGCTCACGCCTGTAATCCAAGCACTTTGGGAGGCCGAGGTGGGTGGATCACGAGGTCAAGAGAT
JS-827-85818-IntF          ~~~~~~~~~~~~~~~~~~~~~~~~~~~~~~~~~~~~~~~~~~~~~~~~~~~~~~~~~~~~~~~~~~~~~~~~~~~~~~~~~~~~~~~GAGGTCAAGAGAT
JS-827-85818-R-rc          ~~~~~~~~~~~~~~~~~~~~~~~~~~~~~~~~~~~~~~~~~~~~~~~~~~~~~~~~~~~~~~~~~~~~~~~~~~~~~~~~~~~~~~~~~~~~~~~~~~~~

                                  1010      1020      1030      1040      1050      1060      1070      1080      1090      1100
                           ....|....|....|....|....|....|....|....|....|....|....|....|....|....|....|....|....|....|....|....|
Storer_2020_Aotus-827      cgagaccatcctggtcaacaaggtgaaaccccgtctctactaaaaatacaaaaaattagctgggcatggtggcgcgtgcctgtaatcccagctactcagg
JS-827-85818-SIntR-rc      CGAGACCATCCTGGTCAACAAGGTGAAACCCCGTCTCTACTAAAAATACAAAAAATTAGCTGGGCATGGTGGCGCGTGCCTGTAATCCCAGCTACTCAGG
JS-827-85818-F             CGAGACCATCCTGGTCAACATGGTGAAACCCCGTCTCTACTAAAAATACAAAAAATTAGCTGGGCATGGTGGCGCGTGCCTGTAATCCCAGCTACTCAGG
JS-827-85818-IntF          CGAGACCATCCTGGTCAACATGGTGAAACCCCGTCTCTACTAAAAATACAAAAAATTAGCTGGGCATGGTGGCGCGTGCCTGTAATCCCAGCTACTCAGG
JS-827-85818-R-rc          ~~~~~~~~~~~~~~~~~~~~~~~~~~~~~~~~~~~~~~~~~~~~~~~~~~~~~~~~~~~~~~~~~~~~~~~~~~~~~~~~~~~~~~~~~~~~~~~~~~~~

                                  1110      1120      1130      1140      1150      1160      1170      1180      1190      1200
                           ....|....|....|....|....|....|....|....|....|....|....|....|....|....|....|....|....|....|....|....|
Storer_2020_Aotus-827      aggctgaggcaggagaattgcctgaacccaggaggtggaggttgtggtgagctgagatcacaccattgcactccagcctgggtaacaagagtgaaactcc
JS-827-85818-SIntR-rc      AGGCTGAGGCAG
JS-827-85818-F             AGGCTGAGGCAGGAGAATTGCCTGAACCCAGGAGGTGGAGGTTGTGGTGAGCTGAGATCGCGCCATTGCACTCCAGCCTGGGTAACAAGAGTGAAACTCC
JS-827-85818-IntF          AGGCTGAGGCAGGAGAATTGCCTGAACCCAGGAGGTGGAGGTTGTGGTGAGCTGAGATCGCGCCATTGCACTCCAGCCTGGGTAACAAGAGTGAAACTCC
JS-827-85818-R-rc          ~~~~~~~~~~~~~~~~~~~~~~~~~~~~~~~~~~~~~~~~~~~~~~~~~~~~~~~~~~~~~~~~~~~~~~~~~~~~~~~~~~~~~~~~~~~~~~~~~~~~

                                  1210      1220      1230      1240      1250      1260      1270      1280      1290      1300
                           ....|....|....|....|....|....|....|....|....|....|....|....|....|....|....|....|....|....|....|....|
Storer_2020_Aotus-827      gtctcaaaaaaaaaaaaaaaaaccctcaaagtaGTAACATCTTTTCCAGtcaataaatgaaaatgtcaGAGCTAATATTGACTGTTGTCTCTCTTCATAT
JS-827-85818-SIntR-rc
JS-827-85818-F             GTCTCAAAAAAAAAAAAAAA
JS-827-85818-IntF          GTCTCAAAAAAAAAAAAAAA
JS-827-85818-R-rc          ~~~~~AAAAAAAAAAAAAAAAACCCTCAAAATAGTAACATCTTTTCCAGTCAATAAATGAAAATGTCAGAGCTAATATTGACTGTTGTCTCTCTTCATAT
```

Figure S17. Sequence alignment for *Alu* locus: Storer, et al. 2020: *Aotus*_827_018493494_6605199 (JS-827). PCR-based genotypes indicate that the target *Alu* insertion is homozygous present in *A. nancymaae* and *A. azarae* individuals while heterozygous (1/0) in *A. lemurinus griseimembra* KB4883 (filled site amplicons too weak to excise gel fragments). The reference genome [Anan_2.0] is shown at the top followed by *A. azarae* 85818 who shares the target insertion starting at position 924 (grey highlight) and flanked by TSDs shown in yellow highlight.

```
                                   510       520       530       540       550       560       570       580       590       600
                           ....|....|....|....|....|....|....|....|....|....|....|....|....|....|....|....|....|....|....|....|
Storer_2020_Aotus-1120     GTCTTCAAGACAATCAGTTGGCAACAAACTAAAACACCCCAGAGAAACTCTGTTACCATATTCTTTGAGACTTCTGCTTTAGAACCATGGATTGTAGTAA
JS-1120-85818-F            ~~~~~~~~~~~~~~~~GTTGGCAACAAACTAAAACACCCCAGAGAAACTCTGTTACCATATTCTTTGAGACTTCTGCTTTAGAACCATGGATTGTAGTAA
JS-1120-85818-IntF         ~~~~~~~~~~~~~~~~~~~~~~~~~~~~~~~~~~~~~~~~~~~~~~~~~~~~~~~~~~~~~~~~~~~~~~~~~~~~~~~~~~~~~~~~~~~~~~~~~~~~
JS-1120-85818-R-rc         ~~~~~~~~~~~~~~~~~~~~~~~~~~~~~~~~~~~~~~~~~~~~~~~~~~~~~~~~~~~~~~~~~~~~~~~~~~~~~~~~~~~~~~~~~~~~~~~~~~~~
JS-1120-85818-IntR-rc      GTCTTCAAGACAATCAGTTGGCAACAAACTAAAACACCCCAGAGAAACTCTGTTACCATATTCTTTGAGACTTCTGCTTTAGAACCATGGATTGTAGTAA

                                   610       620       630       640       650       660       670       680       690       700
                           ....|....|....|....|....|....|....|....|....|....|....|....|....|....|....|....|....|....|....|
Storer_2020_Aotus-1120     TAATaaatttcaggccgggcgcggtggctcacgcctataatcccagcactttgggaagccgaggcgggtggatcacgaggtcaaga~~tcgagaccatcc
JS-1120-85818-F            TAATAAATTTCAGGCCGGGCGCAGTGGCTCACGCCTATAATCCCAGCACTTTGGGAAGCCGAGGCGGGTGGATCACGAGGTCAAGAGATCGAGACCATCC
JS-1120-85818-IntF         ~~~~~~~~~~~~~~~~~~~~~~~~~~~~~~~~~~~~~~~~~~~~~~~~~~~~~~~~~~~~~~~~~~~~~~~~~~~~GAGGTCAAGAGATCGAGACCATCC
JS-1120-85818-R-rc         ~~~~~~~~~~~~~~~~~~~~~~~~~~~~~~~~~~~~~~~~~~~~~~~~~~~~~~~~~~~~~~~~~~~~~~~~~~~~~~~~~~~~~~~~~~~~~~~~~~~~
JS-1120-85818-IntR-rc      TAATAAATTTCAGGCCGGGCGCAGTGGCTCACGCCTATAATCCCAGCACTTTGGGAAGCCGAGGCGGGTGGATCACGAGGTCAAGAGATCGAGACCATCC

                                   710       720       730       740       750       760       770       780       790       800
                           ....|....|....|....|....|....|....|....|....|....|....|....|....|....|....|....|....|....|....|
Storer_2020_Aotus-1120     tggtcaacatggtgaaaccccgtctctactaaaaatacaaaaaattagctgggcatggtggcacgtgcctgtagtcccatctactcaggaggctgaggca
JS-1120-85818-F            TGGTCAACATGGTGAAACCCCGTCTCTACTAAAAATACAAAAAATTAGCTGGGCATGGTGGCGCGTGCCTGTAGTCCCATCTACTCAGGAGGCTGAGGCA
JS-1120-85818-IntF         TGGTCAACATGGTGAAACCCCGTCTCTACTAAAAATACAAAAAATTAGCTGGGCATGGTGGCGCGTGCCTGTAGTCCCATCTACTCAGGAGGCTGAGGCA
JS-1120-85818-R-rc         ~~~~~~~~~~~~~~~~~~~~~~~~~~~~~~~~~~~~~~~~~~~~~~~~~~~~~~~~~~~~~~~~~~~~~~~~~~~~~~~~~~~~~~~~~~~~~~~~~~~~
JS-1120-85818-IntR-rc      TGGTCAACATGGTGAAACCCCGTCTCTACTAAAAATACAAAAAATTAGCTGGGCATGGTGGCGCGTGCCTGTAGTCCCATCTACTCAGGAGG

                                   810       820       830       840       850       860       870       880       890       900
                           ....|....|....|....|....|....|....|....|....|....|....|....|....|....|....|....|....|....|....|
Storer_2020_Aotus-1120     ggagaattgcctgaacccaggaggcggaggttgcggtgagccgagatcgcgccattgcactccagcctgggtagcaagagcgaaactccgtctcaaaaaa
JS-1120-85818-F            GGAGAATTGCCTGAACCCAGGAGGCGGAGGTTGCAGTGAGCCAAGATCGCGCCATTGCACTCCAGCCTGGG
JS-1120-85818-IntF         GGAGAATTGCCTGAACCCAGGAGGCGGAGGTTGCAGTGAGCCGAGATCGCGCCATTGCACTCCAGCCTGGGTAGCAAGAGCGAAACTCCGTCTCAAAAAA
JS-1120-85818-R-rc         ~~~~~~~~~~~~~~~~~~~~~~~~~~~~~~~~~~~~~~~~~~~~~~~~~~~~~~~~~~~~~~~~~~~~~~~~~~~~~~~~~~~~~~~~~~~~~~AAAAAA
JS-1120-85818-IntR-rc

                                   910       920       930       940       950       960       970       980       990       1000
                           ....|....|....|....|....|....|....|....|....|....|....|....|....|....|....|....|....|....|....|
Storer_2020_Aotus-1120     aaaaatttcaatccAATATTGAGAATTAACTGATCACCACTTTCTAAGGCCTAAGCAGCCAGCGCCAGCTTCTGCTGGGAATATCCCCTGCCCAATGTGA
JS-1120-85818-F
JS-1120-85818-IntF         AAAAATTTCAATCCAATATTGAGAAT~AACTGATCACCACTTTCTAAGGCCTAAGCAGCCAGCGCCAGCTTCCGCTGGGAATATCCCCTGCCCAATGTGA
JS-1120-85818-R-rc         AAAAATTTCAATCCAATATTGAGAAT~AACTGATCACCACTTTCTAAGGCCTAAGCAGCCAGCGCCAGCTTCCGCTGGGAATATCCCCTGCCCAATGTGA
JS-1120-85818-IntR-rc
```

Figure S18. Sequence alignment for *Alu* locus: Storer, et al. 2020: *Aotus*_1120_018497016.1:3680404-3681894 (JS-1120). PCR-based genotypes indicate that all *A. nancymaae* and *A. azarae* individuals are homozygous present (1/1) for the target *Alu* insertion, while being absent from other *Aotus* species. The *Aotus* reference genome [Anan_2.0] is shown at the top followed by *A. azarae* 85818 who shares the target *Alu* insertion starting at position 613 (grey highlight), with matching flanking TSDs in yellow highlight.

```
                                     510       520       530       540       550       560       570       580       590       600
                                ....|....|....|....|....|....|....|....|....|....|....|....|....|....|....|....|....|....|....|....|
Storer_2020_Aotus-6430          CAGGGGGGTAGATATATTCTACCTTCTTCAGTGGGTAGCACTGCAAAGTTGCATATCAAAGGACGTGGATgcataattataatacaaaaagTACAttgac
JS-6430-85818-F                 CAGGGGGGTAGATATATTCTACCTTCTTCAGTGGGTAGCACTGCAAAGTTGCATATCAAAGGACGTGGATGCATAATTATAATACAAAAAGTACATTGAC
JS-6430-85818-IntF              ~~~~~~~~~~~~~~~~~~~~~~~~~~~~~~~~~~~~~~~~~~~~~~~~~~~~~~~~~~~~~~~~~~~~~~~~~~~~~~~~~~~~~~~~~~~~~~~~~~~~
JS-6430-85818-R-rc              ~~~~~~~~~~~~~~~~~~~~~~~~~~~~~~~~~~~~~~~~~~~~~~~~~~~~~~~~~~~~~~~~~~~~~~~~~~~~~~~~~~~~~~~~~~~~~~~~~~~~
JS-6430-85818-IntR-rc           CAGGGGGGTAGATATATTCTACCTTCTTCAGTGGGTAGCACTGCAAAGTTGCATATCAAAGGACGTGGATGCATAATTATAATACAAAAAGTACATTGAC

                                     610       620       630       640       650       660       670       680       690       700
                                ....|....|....|....|....|....|....|....|....|....|....|....|....|....|....|....|....|....|....|....|
Storer_2020_Aotus-6430          ggccgggcgcggtggctcaagcctgtaatcccagcactttgggaggccaaggcgggtggatcacgaggtcaagagatcgagaccatcctggtcaacatgg
JS-6430-85818-F                 GGCCGGGCGCGGTGGCTCAAGCCTGTAATCCCAGCACTTTGGGAGGCCGAGGCGGGTGGATCACGAGGTCAAGAGATCGAGACCATCCTGGTCAACATGG
JS-6430-85818-IntF              ~~~~~~~~~~~~~~~~~~~~~~~~~~~~~~~~~~~~~~~~~~~~~~~~~~~~~~~~~~~~~~~~~~~~~~~~~~~~~CGAGACCATCCTGGTCAACATGG
JS-6430-85818-R-rc              ~~~~~~~~~~~~~~~~~~~~~~~~~~~~~~~~~~~~~~~~~~~~~~~~~~~~~~~~~~~~~~~~~~~~~~~~~~~~~~~~~~~~~~~~~~~~~~~~~~~~
JS-6430-85818-IntR-rc           GGCCGGGCGCGGTGGCTCAAGCCTGTAATCCCAGCACTTTGGGAGGCCGAGGCGGGTGGATCACGAGGTCAAGAGATCGAGACCATCCTGGTCAACATGG

                                     710       720       730       740       750       760       770       780       790       800
                                ....|....|....|....|....|....|....|....|....|....|....|....|....|....|....|....|....|....|....|....|
Storer_2020_Aotus-6430          tgaa~ccccgtctctactaatacaaaaa~ttagctgggcacaggtggcgcgtgcctgtaatcccagctactcaggaggctgaggcaggagaattgcctga
JS-6430-85818-F                 TGAA~CCCCGTCTCTACTAATACAAAAAAT
JS-6430-85818-IntF              TGAAACCCCGTCTCTACTAATACAAAAAATTAGCTGGGCA~TGGTGGCGCGTGCCTGTAATCCCAGCTACTCAGGAGGCTGAAGCAGGAGAATTGCCTGA
JS-6430-85818-R-rc              ~~~~~~~~~~~~~~~~~~~~~~~~~~~~~~~~~~~~~~~~~~~~~~~~~~~~~~~~~~~~~~~~~~~~~~~~~~~~~~~~~~~~~~~~~~~~~~~~~~~~
JS-6430-85818-IntR-rc           TGAAACCCCGTCTCTACTAATACAAAAAATTAGCTGGGCAC~GGTGGCGCGTGCCTGTAATCCCAGCTACTCAGGAGG

                                     810       820       830       840       850       860       870       880       890       900
                                ....|....|....|....|....|....|....|....|....|....|....|....|....|....|....|....|....|....|....|....|
Storer_2020_Aotus-6430          acccaggatgcggaggttgcagtgagccgagatcgcgccattgcactccagcctgggtatcaagagcgaaactccgtctcaaaaaaaaaaaaaaaaagta
JS-6430-85818-F
JS-6430-85818-IntF              ACCCAGGAGGCGGAGGTTGCAGTGAGCCGAGATCGCGCCATTGCACTCCAGCCTGGGTATCAAGAGCGAAAC
JS-6430-85818-R-rc              ~~~~~~~~~~~~~~~~~~~~~~~~~~~~~~~~~~~~~~~~~~~~~~~~~~~~~~~~~~~~~~~~~~~~~~~~~~~~~AAAAAAAAAAAAAAAAAAAAGTA
JS-6430-85818-IntR-rc

                                     910       920       930       940       950       960       970       980       990      1000
                                ....|....|....|....|....|....|....|....|....|....|....|....|....|....|....|....|....|....|....|....|
Storer_2020_Aotus-6430          cattgacGATTAAATGCAGTGATTTAATCTACCTTAGCAGTATAATCTACTGAGAGGAGAGCAACATTTCCTATCTATGTTTTCACACAAAATAGGCAAA
JS-6430-85818-F
JS-6430-85818-IntF
JS-6430-85818-R-rc              CATTGACGATTAAATGCAGGGATTTAATCTACCTTAGCAGTATAATCTACTGAGAGGAGAGCAACATTTCCTATCTATGTTTTCACACAAAATAGGCAAA
JS-6430-85818-IntR-rc
```

Figure S19. Sequence alignment for *Alu* locus: Storer, et al. 2020: *Aotus*_6430_018507789.1:443811-445305 (JS-6430). PCR-based genotypes indicate the target *Alu* insertion is polymorphic among *A. nancymaae* individuals, homozygous present in all three *A. azarae* individuals, while being absent from other *Aotus* species. The *Aotus* reference genome [Anan_2.0] is shown at the top followed by *A. azarae* 85818 who shares the target *Alu* insertion starting at position 601 (grey highlight), with matching flanking TSDs in yellow highlight.

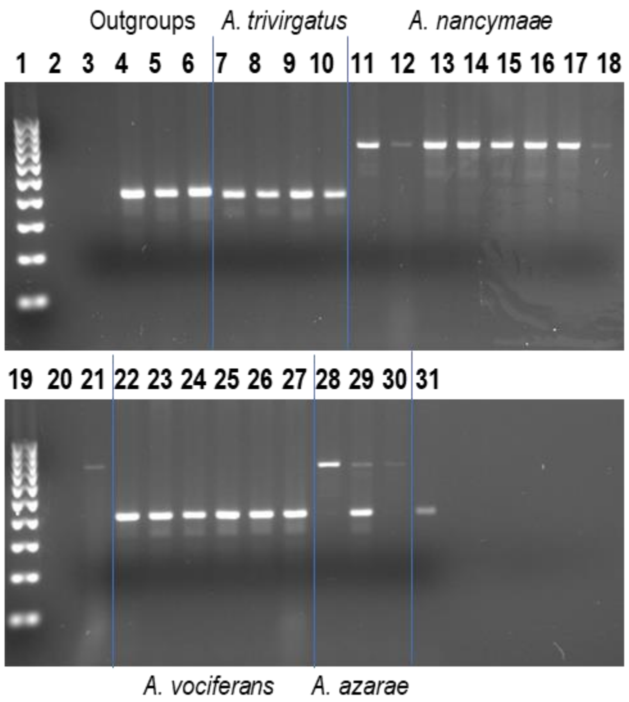

Figure S20a: *Alu* locus *Aotus*_404_018513849.1:2616941-2618444 (JW-404): Lanes: 1, 19- 100 bp DNA ladder, 2, 20-blank, 3- TLE (negative control), 4- Human (HeLa), 5- *Callithrix jacchus* (Common marmoset), 6- *Saimiri s. sciureus* (Common squirrel monkey), 7-10 *A. trivirgatus*, 11-18, 21 *A. nancymaae*, 22-27 *A. vociferans*, 28-30 *A. azarae*, 31-*A. lemurinus griseimembra.* Vertical blue lines superimposed on the gel image visually separate the species groups. The predicted amplicon sizes are 715/411 bp. These PCR genotypes indicate that the target *Alu* insertion is homozygous present in *A. nancymaae* (715 bp fragment) and shared by *A. azarae* individuals, while homozygous absent in grey-necked species. DNA sequencing was performed to determine if *A. azarae* samples share the target insertion (See Figure S20b).

```
                                  310       320       330       340       350       360       370       380       390       400
                         ....|....|....|....|....|....|....|....|....|....|....|....|....|....|....|....|....|....|....|....|
Aotus_404 [Anan_2.0]     ggagtgagtctcaaagaaaaaaaaaaaaaacagaaggaagaaccTGAAAATGCTCCCTGCCATAACCATCccagtttctctcttgtttctcCAAGATTAC
JW-404-85818-R           ~~~~~~~~~~~~~~~~~~~~~~~~~~~~~~~~~~~~~~~~~~~~~~~~~~~~~~~~~~~~~~~~~~~~~~~~~~~~~~~~~~~~~~~~~~~~~~~~~~~~
JW-404-85818-IntF-rc     ~~~~~~~~~~~~~~~~~~~~~~~~~~~~~~~~~~~~~~~~~~~~~~~~~~~~~~~~~~~~~~~~~~~~~~~~~~~~~~~~~~~~~~~~~~~~~~~~~~~
JW-404-85818-F-rc        ~~~~~~~~~~~~~~~~~~~~~~~~~~~~~~~~~~~~~~~~~~~~~~~~~~~~~~~~~~~~~~~~~~~~~~~~~~~~~~~~~~~~~~~~~~~~~~~~~~~
JW-404-85818-SIntR       ~~~~~~~~~~~~~~~~~~~~~~~~~~~~~~~~~~~~~~~~~~~~~~~~~~~~~~~~~~~~~~~~~~~~~~~~~~~~~~~~~~~~~~~~~~~~~~~~~~~
JW-404-85457-R           ~~~~~~~~~~~~~~~~~~~~~~~~~~~~~~~~~~~~~~~~~~~~~~~~~~~~~~~~~~~~~~~~~~~~~~~~~~~~~~~~~~~~~~~~~~~~~~~~~~~
JW-404-85457-IntF-rc     ~~~~~~~~~~~~~~~~~~~~~~~~~~~~~~~~~~~~~~~~~~~~~~~~~~~~~~~~~~~~~~~~~~~~~~~~~~~~~~~~~~~~~~~~~~~~~~~~~~~
JW-404-85457-F-rc        ~~~~~~~~~~~~~~~~~~~~~~~~~~~~~~~~~~~~~~~~~~~~~~~~~~~~~~~~~~~~~~~~~~~~~~~~~~~~~~~~~~~~~~~~~~~~~~~~~~~
JW-404-85457-SIntR       ~~~~~~~~~~~~~~~~~~~~~~~~~~~~~~~~~~~~~~~~~~~~~~~~~~~~~~~~~~~~~~~~~~~~~~~~~~~~~~~~~~~~~~~~~~~~~~~~~~~
JW-404-KB10043-R         ~~~~~~~~~~~~~~~~~~~~~~~~~~~~~~~~~~~~~~~~~~~~~~~~~~~~~~~~~~~~~~~~~~~~~~~~~~~~~~~~~~~~~~~~~~~~~~~~~~~
JW-404-KB10043-IntF-rc   ~~~~~~~~~~~~~~~~~~~~~~~~~~~~~~~~~~~~~~~~~~~~~~~~~~~~~~~~~~~~~~~~~~~~~~~~~~~~~~~~~~~~~~~~~~~~~~~~~~~
JW-404-KB10043-F-rc      ~~~~~~~~~~~~~~~~~~~~~~~~~~~~~~~~~~~~~~~~~~~~~~~~~~~~~~~~~~~~~~~~~~~~~~~~~~~~~~~~~~~~~~~~~~~~~~~~~~~
JW-404-KB10043-SintR     ~~~~~~~~~~~~~~~~~~~~~~~~~~~~~~~~~~~~~~~~~~~~~~~~~~~~~~~~~~~~~~~~~~~~~~~~~~~~~~~~~~~~~~~~~~~~~~~~~~~

                                  410       420       430       440       450       460       470       480       490       500
                         ....|....|....|....|....|....|....|....|....|....|....|....|....|....|....|....|....|....|....|....|
Aotus_404 [Anan_2.0]     AGCCACAGTGGTCATGAATCAACTATTACCAACAATCAAGTGGGAAAATGCCATAATTTCGGTCTTTTAGAGACAGGAAACAGGAGCCCAGTGAGGCAAT
JW-404-85818-R           ~~~~~~~~~~~~~~~~~~~~~~~~~~~~~~~~~~~~~~~~~~~~~~~~TGCCATAATTTCGGTCTTTTAGAGACAGGAAACAGGAGCCCAGTGAGGCAAT
JW-404-85818-IntF-rc     ~~~~~~~~~~~~~~~~~~~~~~~~~~~~~~~~~~~~~~~~~~~~~~~~~~~~~~~~~~~~~~~~~~~~~~~~~~~~~~~~~~~~~~~~~~~~~~~~~~~
JW-404-85818-F-rc        ~~~~~~~~~~~~~~~~~~~~~~~~~~~~~~~~~~~~~~~~~~~~~~~~~~~~~~~~~~~~~~~~~~~~~~~~~~~~~~~~~~~~~~~~~~~~~~~~~~~
JW-404-85818-SIntR       ~~~~~~~~~~~~~~~~~~~~~~~~~~~~~~~~~~~~~~~~~~~~~~~~~~~~~~~~~~~~~~~~~~~~~~~~~~~~~~~~~~~~~~~~~~~~~~~~~~~
JW-404-85457-R           ~~~~~~~~~~~~~~~~~~~~~~~~~~~~~~~~~~~~~~~GTGGG~AAATGCCATAATTTCGGTCTTTTAGAGACAGGAAACAGGAGCCCAGTGAGGCAAT
JW-404-85457-IntF-rc     ~~~~~~~~~~~~~~~~~~~~~~~~~~~~~~~~~~~~~~~~~~~~~~~~~~~~~~~~~~~~~~~~~~~~~~~~~~~~~~~~~~~~~~~~~~~~~~~~~~~
JW-404-85457-F-rc        ~~~~~~~~~~~~~~~~~~~~~~~~~~~~~~~~~~~~~~~~~~~~~~~~~~~~~~~~~~~~~~~~~~~~~~~~~~~~~~~~~~~~~~~~~~~~~~~~~~~
JW-404-85457-SIntR       ~~~~~~~~~~~~~~~~~~~~~~~~~~~~~~~~~~~~~~~~~~~~~~~~~~~~~~~~~~~~~~~~~~~~~~~~~~~~~~~~~~~~~~~~~~~~~~~~~~~
JW-404-KB10043-R         ~~~~~~~~~~~~~~~~~~~~~~~~~~~~~~~~~~~~~~~~~~~~~~~~TGCCATAATTTCGGTCTTTTAGAGACAGGAAACAGGAGCCCNNNNANGNAAT
JW-404-KB10043-IntF-rc   ~~~~~~~~~~~~~~~~~~~~~~~~~~~~~~~~~~~~~~~~~~~~~~~~~~~~~~~~~~~~~~~~~~~~~~~~~~~~~~~~~~~~~~~~~~~~~~~~~~~
JW-404-KB10043-F-rc      ~~~~~~~~~~~~~~~~~~~~~~~~~~~~~~~~~~~~~~~~~~~~~~~~~~~~~~~~~~~~~~~~~~~~~~~~~~~~~~~~~~~~~~~~~~~~~~~~~~~
JW-404-KB10043-SintR     ~~~~~~~~~~~~~~~~~~~~~~~~~~~~~~~~~~~~~~~~~~~~~~~~~~~~~~~~~~~~~~~~~~~~~~~~~~~~~~~~~~~~~~~~~~~~~~~~~~~

                                  510       520       530       540       550       560       570       580       590       600
                         ....|....|....|....|....|....|....|....|....|....|....|....|....|....|....|....|....|....|....|
Aotus_404 [Anan_2.0]     TAAAGAGGTAGT~~~~~~~~~~~~~~~~~~~~~~~~~~~~~~~~~~~~~~~~~~~~~~~~~~~~~~~~~~~~~~~~~~~~~~~~~~~~~~~~~~~~
JW-404-85818-R           TAAAGAGGTAGTGAGGTTTCACTTCTTTTTTTTTTTT~~~~~~~~~~~~~~~~~~~~~~~~~~~~~~~~~~~~~~~~~~~~~~~~~~~~~~~~~~~~~~~
JW-404-85818-IntF-rc     ~~~~~~~~~~~~~~~~TTTTTTTTTTTTTTTTTTTTTGAGACGGAGTTTCGCTCGTTACCCAGGCTGGAGTGCAATGGCGCGATCTCGGCTCACCGCAAC
JW-404-85818-F-rc        ~~~~~~~~~~~~~~~~TTTTTTTTTTTTTTTTTTTTTGAGACGGAGTTTCGCTCGTTACCCAGGCTGGAGTGCAATGGCGCGATCTCGGCTCACCGCAAC
JW-404-85818-SIntR       ~~~~~~~~~~~~~~~~~~~~~~~~~~~~~~~~~~~~~~~~~~~~~~~~~~~~~~~~~~~~~~~~~~~~~~~~~~~~~~~~~~~~~~~~~~~~~~~~~~~
JW-404-85457-R           TAAAGAGGTAGTGAGGTTTCACTTCTTTTTTTTTTTT~~~~~~~~~~~~~~~~~~~~~~~~~~~~~~~~~~~~~~~~~~~~~~~~~~~~~~~~~~~~~~~
JW-404-85457-IntF-rc     ~~~~~~~~~~~~~~~~~~TTTTTTTTTTTTTTTTTTTGAGACGGAGTTTCGCTCGTTACCCAGGCTGGAGTGCAATGGCGCGATCTCGGCTCACCGCAAC
JW-404-85457-F-rc        ~~~~~~~~~~~~~~~~~~~~~~~~~~~~~~~~~~~~~~~~~~~~~~~~~~~~~~~~~~~~~~~~~~~~~~~~~~~~~~~~~~~~~~~~~~~~~~~~~~~
JW-404-85457-SIntR       ~~~~~~~~~~~~~~~~~~~~~~~~~~~~~~~~~~~~~~~~~~~~~~~~~~~~~~~~~~~~~~~~~~~~~~~~~~~~~~~~~~~~~~~~~~~~~~~~~~~
JW-404-KB10043-R         TAAAGAGGTAGTGAGGTTTCACTTCTTTTTTTTTTTT
JW-404-KB10043-IntF-rc   ~~~~~~~~~~~~~~~~~~~~~~~~~~~~TTTTTTTTTGAGACGGAGTTTCGCTCGTTACCCAGGCTGGAGTGCAATGGCGCGATCTCGGCTCACCGCAAC
JW-404-KB10043-F-rc      ~~~~~~~~~~~~~~~~~~~~~~~~~~~~~~~~~~~~~~~~~~~~~~~~~~~~~~~~~~~~~~~~~~~~~~~~~~~~~~~~~~~~~~~~~~~~~~~~~~~
JW-404-KB10043-SintR     ~~~~~~~~~~~~~~~~~~~~~~~~~~~~~~~~~~~~~~~~~~~~~~~~~~~~~~~~~~~~~~~~~~~~~~~~~~~~~~~~~~~~~~~~~~~~~~~~~~~
```

```
                                                610       620       630       640       650       660       670       680       690       700
                                       ....|....|....|....|....|....|....|....|....|....|....|....|....|....|....|....|....|....|....|....|
Aotus_404 [Anan_2.0]                   ~~~~~~~~~~~~~~~~~~~~~~~~~~~~~~~~~~~~~~~~~~~~~~~~~~~~~~~~~~~~~~~~~~~~~~~~~~~~~~~~~~~~~~~~~~~~~~~~~~~~
JW-404-85818-R                         ~~~~~~~~~~~~~~~~~~~~~~~~~~~~~~~~~~~~~~~~~~~~~~~~~~~~~~~~~~~~~~~~~~~~~~~~~~~~~~~~~~~~~~~~~~~~~~~~~~~~
JW-404-85818-IntF-rc                   CTCCGCCTCTTGGGTTCAGGCAATTCTCCTGCCTCAGCCTCCTGAGTAGCTGGGATTACAGGCACGCGCCACCATGCCCAGCTAATTTTTTGTATTAGTA
JW-404-85818-F-rc                      CTCCGCCTCTTGGGTTCAGGCAATTCTCCTGCCTCAGCCTCCTGAGTAGCTGGGATTACAGGCACGCGCCACCATGCCCAGCTAATTTT~CGTATTAGTA
JW-404-85818-SIntR                     ~~~~~~~~~~~~~~~~~~~~~~~~~~~~~~~~~~~~~CCTCCTGAGTAGCTGGGATTACAGGCACGCGCCACCATGCCCAGCNAATTTTTTGTATTAGTA
JW-404-85457-R                         ~~~~~~~~~~~~~~~~~~~~~~~~~~~~~~~~~~~~~~~~~~~~~~~~~~~~~~~~~~~~~~~~~~~~~~~~~~~~~~~~~~~~~~~~~~~~~~~~~~~~
JW-404-85457-IntF-rc                   CTCCGCCTCTTGGGTTCAGGCAATTCTCCTGCCTCAGCCTCCTGAGTAGCTGGGATTACAGGCACGCGCCACCATGCCCAGCTAATTTTTTGTATTAGTA
JW-404-85457-F-rc                      ~~~~~~~~~~~~~~~~~~~~~~~~~~~~~~~~~~~~~~~~~~~~~~~~~~~~~~~~~~~AGGCACGCGCCACCATGCCCAGCTAATTTTTTGTATTAGTA
JW-404-85457-SIntR                     ~~~~~~~~~~~~~~~~~~~~~~~~~~~~~~~~~~~~~~~~CCTGAGTAGCTGGGATTACAGGCACGCGCCACCATGCCCAGCTAATTTTTTGTATTAGTA
JW-404-KB10043-R
JW-404-KB10043-IntF-rc                 CTCCGCCTCTTGGGTTCAGGCAATTCTCCTGCCTCAGCCTCCTGAGTAGCTGGGATTANAGGCNCGCGCCACCATGCCCAGCTAATTTTTTGTATTAGTA
JW-404-KB10043-F-rc                    ~~~~~~~~~~~~~~~~~~~~CAATTTTCCTGCCTCAGCCTCCTGAGNANAANGGATTACAGGCACGCGCCACCATGCCCAGCTAATTTTTTGTATTAGTA
JW-404-KB10043-SintR                   ~~~~~~~~~~~~~~~~~~~~~~~~~~~~~~~~~~~~~CCTCCTGAGTAGCTGGGATTACAGGCACGCGCCACCATGCCCAGCTAATTTTTTGTATTAGTA

                                                710       720       730       740       750       760       770       780       790       800
                                       ....|....|....|....|....|....|....|....|....|....|....|....|....|....|....|....|....|....|....|....|
Aotus_404 [Anan_2.0]                   ~~~~~~~~~~~~~~~~~~~~~~~~~~~~~~~~~~~~~~~~~~~~~~~~~~~~~~~~~~~~~~~~~~~~~~~~~~~~~~~~~~~~~~~~~~~~~~~~~~~~
JW-404-85818-R                         ~~~~~~~~~~~~~~~~~~~~~~~~~~~~~~~~~~~~~~~~~~~~~~~~~~~~~~~~~~~~~~~~~~~~~~~~~~~~~~~~~~~~~~~~~~~~~~~~~~~~
JW-404-85818-IntF-rc                   GAGANGGGGTTTCACCATGTCGACCAGGATGGTCTCGATCTCTTGACCTC~~~~~
JW-404-85818-F-rc                      GAGACGGGGTTTCACCATGTCGACCAGGATGGTCTCGATCTCTTGACCTCGTGATCCACCCGCCTCGGCCTCCCACAGGGATGGGATTACAGGCTTGAGC
JW-404-85818-SIntR                     GAGACGGGGTTTCACCATGTCGACCAGGATGGTCTCGATCTCTTGACCTCGTGATCCACCCGCCTCGGCCTCCCAAAGTGCTGGGATTACAGGCTTGAGC
JW-404-85457-R                         ~~~~~~~~~~~~~~~~~~~~~~~~~~~~~~~~~~~~~~~~~~~~~~~~~~~~~~~~~~~~~~~~~~~~~~~~~~~~~~~~~~~~~~~~~~~~~~~~~~~~
JW-404-85457-IntF-rc                   GAGACGGGGTTTCACCATGTCGACCAGGATGGTCTCGATCTCTTGACCTC
JW-404-85457-F-rc                      GAGACGGGGTTTCACCATGTCGACCAGGATGGTCTCGATCTCTTGACCTCGTGATCCACCCGCCTCGGCCTCCCAAAGTGCTGGGATTACAGGCTTGAGC
JW-404-85457-SIntR                     GAGACGGGGTTTCACCATGTCGACCAGGATGGTCTCGATCTCTTGACCTCGTGATCCACCCGCCTCGGCCTCCCAAAGTGCTGGGATTACAGGCTTGAGC
JW-404-KB10043-R
JW-404-KB10043-IntF-rc                 GANNNGGGGTTTCACCATGTCGACCAGGATGGTCTCGATCTCT~GACCTC~~~~
JW-404-KB10043-F-rc                    GAGACGGGGTTTCACCATGTCGACCAGGATGGTCTCGATCTCTTGACCTCGTGATCCACCCGCCTCGGCCTCCCAAAGTGCTGGGATTACAGGCTTGAGC
JW-404-KB10043-SintR                   GAGACGGGGTTTCACCATGTCGACCAGGATGGTCTCGATCTCTTGACCTCGTGATCCACCCGCCTCGGCCTCCCAAAGTGCTGGGATTACAGGCTTGAGC

                                                810       820       830       840       850       860       870       880       890       900
                                       ....|....|....|....|....|....|....|....|....|....|....|....|....|....|....|....|....|....|....|....|
Aotus_404 [Anan_2.0]                   ~~~~~~~~~~~~~~GAGGTTTCACTTCTAAACCTTCAAGAAACTCCACTCCCAATGAGGGTAGCTGCAGCACTTAGGGATATTCTAAAGAAACCCCacaa
JW-404-85818-R                         ~~~~~~~~~~~~~~~~~~~~~~~~~~~~~~~~~~~~~~~~~~~~
JW-404-85818-IntF-rc
JW-404-85818-F-rc                      CACCGCGCCCGGCCGAGGTTTCACTTCTAAACCTTCAAGAAACTCCACTCCCAATGAGGGTAGCTGCAGCACTTAGGGATATTCTAAAGAAACCCCACAA
JW-404-85818-SIntR                     CACCGCGCCCGGCCGAGGTTTCACTTCTAAACCTTCAAGAAACTCCACTCCCAATGAGGGTAGCTGCAGCACTTAGGGATATTCTAAAGAAACCCCACAA
JW-404-85457-R                         ~~~~~~~~~~~~~~~~~~~~~~~~~~~~~~~~~~~~~~~~~~~~
JW-404-85457-IntF-rc
JW-404-85457-F-rc                      CACCGCGCCCGGCCGAGGTTTCACTTCTAAACCTTCAAGAAACTCCACTCCCAATGAGGGTAGCTGCAGCACTTAGGGATATTCTAAAGAAACCCCACAA
JW-404-85457-SIntR                     CACCGCGCCCGGCCGAGGTTTCACTTCTAAACCTTCAAGAAACTCCACTCCCAATGAGGGTAGCTGCAGCACTTAGGGATATTCTAAAGAAACCCCACAA
JW-404-KB10043-R
JW-404-KB10043-IntF-rc
JW-404-KB10043-F-rc                    CACCGCGCCCGGCCGAGGTTTCACTTCTAAACCTTCAAGAAACTCCACTCCCAATGAGGGTAGCTGCAGCACTTAGGGATATTCTAAAGAAACCCCACAA
JW-404-KB10043-SintR                   CACCGCGCCCGGCCGAGGTTTCACTTCTAAACCTTCAAGAAACTCCACTCCCAATGAGGGTAGCTGCAGCACTTAGGGATATTCTAAAGAAACCCCACAA
```

```
                                    910       920       930       940       950       960       970       980       990      1000
                           ....|....|....|....|....|....|....|....|....|....|....|....|....|....|....|....|....|....|....|....|
Aotus_404 [Anan_2.0]       taggccgggcgcggtggctcaagcctgtaatcccagcactttgggaggccgaggcgggtggatcacgaggtcaagatatcgagaccatcctggtcaacat
JW-404-85818-R
JW-404-85818-IntF-rc
JW-404-85818-F-rc          TA~~~~~~~~~~~~~~~~~~~~~~~~~~~~~~~~~~~~~~~~~~~~~~~~~~~~~~~~~~~~~~~~~~~~~~~~~~~~~~~~~~~~~~~~~~~~~~~~~~
JW-404-85818-SIntR         TA~~~~~~~~~~~~~~~~~~~~~~~~~~~~~~~~~~~~~~~~~~~~~~~~~~~~~~~~~~~~~~~~~~~~~~~~~~~~~~~~~~~~~~~~~~~~~~~
JW-404-85457-R
JW-404-85457-IntF-rc
JW-404-85457-F-rc          TA~~~~~~~~~~~~~~~~~~~~~~~~~~~~~~~~~~~~~~~~~~~~~~~~~~~~~~~~~~~~~~~~~~~~~~~~~~~~~~~~~~~~~~~~~~~~~~~
JW-404-85457-SIntR         TA~~~~~~~~~~~~~~~~~~~~~~~~~~~~~~~~~~~~~~~~~~~~~~~~~~~~~~~~~~~~~~~~~~~~~~~~~~~~~~~~~~~~~~~~~~~~~~~
JW-404-KB10043-R
JW-404-KB10043-IntF-rc
JW-404-KB10043-F-rc        TA~~~~~~~~~~~~~~~~~~~~~~~~~~~~~~~~~~~~~~~~~~~~~~~~~~~~~~~~~~~~~~~~~~~~~~~~~~~~~~~~~~~~~~~~~~~~~~~
JW-404-KB10043-SintR       TA~~~~~~~~~~~~~~~~~~~~~~~~~~~~~~~~~~~~~~~~~~~~~~~~~~~~~~~~~~~~~~~~~~~~~~~~~~~~~~~~~~~~~~~~~~~~~~~

                                   1010      1020      1030      1040      1050      1060      1070      1080      1090      1100
                           ....|....|....|....|....|....|....|....|....|....|....|....|....|....|....|....|....|....|....|....|
Aotus_404 [Anan_2.0]       tgtgaaaccccgtctctactaatacaaaaaattagctgggcatggtggcgcgtgcctgtaatcccagctactcaggaagctgaggcaggagaatgctgaa
JW-404-85818-R
JW-404-85818-IntF-rc
JW-404-85818-F-rc          ~~~~~~~~~~~~~~~~~~~~~~~~~~~~~~~~~~~~~~~~~~~~~~~~~~~~~~~~~~~~~~~~~~~~~~~~~~~~~~~~~~~~~~~~~~~~~~~~
JW-404-85818-SIntR         ~~~~~~~~~~~~~~~~~~~~~~~~~~~~~~~~~~~~~~~~~~~~~~~~~~~~~~~~~~~~~~~~~~~~~~~~~~~~~~~~~~~~~~~~~~~~~~~~
JW-404-85457-R
JW-404-85457-IntF-rc
JW-404-85457-F-rc          ~~~~~~~~~~~~~~~~~~~~~~~~~~~~~~~~~~~~~~~~~~~~~~~~~~~~~~~~~~~~~~~~~~~~~~~~~~~~~~~~~~~~~~~~~~~~~~~~
JW-404-85457-SIntR         ~~~~~~~~~~~~~~~~~~~~~~~~~~~~~~~~~~~~~~~~~~~~~~~~~~~~~~~~~~~~~~~~~~~~~~~~~~~~~~~~~~~~~~~~~~~~~~~~
JW-404-KB10043-R
JW-404-KB10043-IntF-rc
JW-404-KB10043-F-rc        ~~~~~~~~~~~~~~~~~~~~~~~~~~~~~~~~~~~~~~~~~~~~~~~~~~~~~~~~~~~~~~~~~~~~~~~~~~~~~~~~~~~~~~~~~~~~~~~~
JW-404-KB10043-SintR       ~~~~~~~~~~~~~~~~~~~~~~~~~~~~~~~~~~~~~~~~~~~~~~~~~~~~~~~~~~~~~~~~~~~~~~~~~~~~~~~~~~~~~~~~~~~~~~~~

                                   1110      1120      1130      1140      1150      1160      1170      1180      1190      1200
                           ....|....|....|....|....|....|....|....|....|....|....|....|....|....|....|....|....|....|....|....|
Aotus_404 [Anan_2.0]       cccaggaggcggaggttgcagtgagccgagatcgcgcattgcactccagcctgggtaccgagagcgaaactccgtctcaaaaaaaaaaaaaaaaaaaaaa
JW-404-85818-R
JW-404-85818-IntF-rc
JW-404-85818-F-rc          ~~~~~~~~~~~~~~~~~~~~~~~~~~~~~~~~~~~~~~~~~~~~~~~~~~~~~~~~~~~~~~~~~~~~~~~~~~~~~~~~~~~~~~~~~~~~~~~~
JW-404-85818-SIntR         ~~~~~~~~~~~~~~~~~~~~~~~~~~~~~~~~~~~~~~~~~~~~~~~~~~~~~~~~~~~~~~~~~~~~~~~~~~~~~~~~~~~~~~~~~~~~~~~~
JW-404-85457-R
JW-404-85457-IntF-rc
JW-404-85457-F-rc          ~~~~~~~~~~~~~~~~~~~~~~~~~~~~~~~~~~~~~~~~~~~~~~~~~~~~~~~~~~~~~~~~~~~~~~~~~~~~~~~~~~~~~~~~~~~~~~~~
JW-404-85457-SIntR         ~~~~~~~~~~~~~~~~~~~~~~~~~~~~~~~~~~~~~~~~~~~~~~~~~~~~~~~~~~~~~~~~~~~~~~~~~~~~~~~~~~~~~~~~~~~~~~~~
JW-404-KB10043-R
JW-404-KB10043-IntF-rc
JW-404-KB10043-F-rc        ~~~~~~~~~~~~~~~~~~~~~~~~~~~~~~~~~~~~~~~~~~~~~~~~~~~~~~~~~~~~~~~~~~~~~~~~~~~~~~~~~~~~~~~~~~~~~~~~
JW-404-KB10043-SintR       ~~~~~~~~~~~~~~~~~~~~~~~~~~~~~~~~~~~~~~~~~~~~~~~~~~~~~~~~~~~~~~~~~~~~~~~~~~~~~~~~~~~~~~~~~~~~~~~~
```

```
                                   1210      1220      1230      1240      1250      1260      1270      1280      1290      1300
                             ....|....|....|....|....|....|....|....|....|....|....|....|....|....|....|....|....|....|....|....|
Aotus_404 [Anan_2.0]         aagaaaccccacaaTATTCTTTCatgcatttgtttgtttactcATTTTCAACTAAGATGTAAGCTGCTGGAAGGCCAACTGTGTCAGTCCTGCTCACTGC
JW-404-85818-R
JW-404-85818-IntF-rc
JW-404-85818-F-rc            ~~~~~~~~~~~~~~~~TTCTTTCATGCATTTGTTTGTTTACTCATTTTCAACTAAGATGTAAGCTGCTGGAAGGCCAACTGTGTCAGTCCTGCTCACTGC
JW-404-85818-SIntR           ~~~~~~~~~~~~~~~~TTCTTTCATGCATTTGTTTGTTTACTCATTTTCAACTAAGATGTAAGCTGCTGGAAGGCCAACTGTGTCAGTCCTGCTCACTGC
JW-404-85457-R
JW-404-85457-IntF-rc
JW-404-85457-F-rc            ~~~~~~~~~~~~~~~~TTCTTTCATGCATTTGTTTGTTTACTCATTTTCAACTAAGATGTAAGCTGCTGGAAGGCCAACTGTGTCAGTCCTGCTCACTGC
JW-404-85457-SIntR           ~~~~~~~~~~~~~~~~TTCTTTCATGCATTTGTTTGTTTACTCATTTTCAACTAAGATGTAAGCTGCTGGAAGGCCAACTGTGTCAGTCCTGCTCACTGC
JW-404-KB10043-R
JW-404-KB10043-IntF-rc
JW-404-KB10043-F-rc          ~~~~~~~~~~~~~~~~TTCTTTCATGCATTTGTTTGTTTACTCATTTTCAACTAAGATGTAAGCNGCTGGAAGGCCAACTGTGTCAGTCCTGCTCACTGC
JW-404-KB10043-SintR         ~~~~~~~~~~~~~~~~TTCTTTCATGCATTTGTTTGTTTACTCATTTTCAACTAAGATGTAAGCTGCTGGAAGGCCAACTGTGTCAGTCCTGCTCACTGC

                                   1310      1320      1330      1340      1350      1360      1370      1380      1390      1400
                             ....|....|....|....|....|....|....|....|....|....|....|....|....|....|....|....|....|....|....|....|
Aotus_404 [Anan_2.0]         CAGACCCCCATCACCCAGCATGAAAATGTTTCATCTTAGCCATGAGAATGTTTATTCCTGAATTATTTGTTGGCATAGAAGTTACATGGGCCAGAAGATT
JW-404-85818-R
JW-404-85818-IntF-rc
JW-404-85818-F-rc            CAGACCCCCATCACCCAGCATGAAAATGTTTCATCTTAGCCATGAGAA
JW-404-85818-SIntR           CAGACCCCCATCACCCAGCATGAAAATGTTTCATCTTAGCCATGAGAATGTTTATTCCTGAATTATTTGTTGGCATAGAAGTTACATGGGCCAGAAGATT
JW-404-85457-R
JW-404-85457-IntF-rc
JW-404-85457-F-rc            CAGACCCCCATCACCCAGCATGAAAATGTTTCATCTTAGCCATGAGAATGTTT~~~~~~~~~~~~~~~~~~~~~~~~~~~~~~~~~~~~~
JW-404-85457-SIntR           CAGACCCCCATCACCCAGCATGAAAATGTTTCATCTTAGCCATGAGAATGTTTATTCCTGAATTATTTGTTGGCATAGAAGTTACATGGGCCAGAAGATT
JW-404-KB10043-R
JW-404-KB10043-IntF-rc
JW-404-KB10043-F-rc          CTNNCCCCCATCACCCAGCATGAAAATGTTTCATCTTAGCCATGAGAA
JW-404-KB10043-SintR         CAGACCCCCATCACCCAGCATGAAAATGTTTCATCTTAGCCATGAGAATGTTTATTCCTGAATTATTTGTTGGCATAGAAGTTACATGGGCCAGAAGATT

                                   1410      1420      1430      1440      1450      1460      1470      1480      1490      1500
                             ....|....|....|....|....|....|....|....|....|....|....|....|....|....|....|....|....|....|....|....|
Aotus_404 [Anan_2.0]         TAGCACTTTCTAATTGTTAATGTAAATTGCACTAGAAAATACACACACTATATCTTATGGCCACACAAGTAAAGCTCTTATTTGAGTGTTCACTTGTCCC
JW-404-85818-R
JW-404-85818-IntF-rc
JW-404-85818-F-rc
JW-404-85818-SIntR           TA
JW-404-85457-R
JW-404-85457-IntF-rc
JW-404-85457-F-rc
JW-404-85457-SIntR           TAGCAA~
JW-404-KB10043-R
JW-404-KB10043-IntF-rc
JW-404-KB10043-F-rc
JW-404-KB10043-SintR         TAG
```

Figure S20b. Sequence alignment for *Alu* locus *Aotus*_404_018513849.1:2616941-2618444 (JW-404). PCR-based genotypes indicate the target *Alu* insertion is homozygous present (1/1) in *A. nancymaae* individuals and two of three *A. azarae* individuals while being

heterozygous present (1/0) in the third *A. azarae* sample and homozygous absent (0/0) in other *Aotus* species. DNA sequencing was performed to determine if the target insertion is shared by *A. azarae*. The reference genome [Anan_2.0] is shown at the top followed by *A. azarae* samples 85818, 85457 and KB10043. This alignment has been expanded to accommodate the sequence trace data. Oligonucleotide primers for PCR are highlighted in bright green at positions 389 and 1385. The target *Alu* starts at position 903 (grey highlight) and has TSDs (AAAGAAACCCCACAATA) in yellow highlight. All three *A. azarae* samples lack the target insertion as sequence traces span the pre-integration site. Instead, the *A. azarae* individuals all share a different *Alu*, a near parallel insertion, starting at position 815 (teal highlight) and running in the reverse orientation back to the T-tail (reverse compliment of an A-tail) at position 527. TSDs (GAGGTTTCACTTCT) are shown in aqua highlight before sequence traces once again align with the reference genome. This is the only confirmed case in which all three *A. azarae* samples on our panel appeared to share the target insertion but did not. Locus *Aotus*_1196 had only one of three *A. azarae* sample, 85818, who appeared to share the target insertion but had a near parallel insertion instead. An alternative forward PCR primer has been designed, *Aotus*_404_azarae-F: AGTGCTGCAGCTACCCTCAT (purple highlight) that could be used in conjunction with the existing reverse primer shown at position 389 (CTCCAAGATTACAGCCACAGTG) to potentially genotype this *A. azarae* derived *Alu* element separately. We did not attempt this PCR as the locus is not parsimony informative within our sample set.

```
                                  210       220       230       240       250       260       270       280       290       300
                         ....|....|....|....|....|....|....|....|....|....|....|....|....|....|....|....|....|....|....|....|
Aotus_1196_[Anan_2.0]    actaaaataataattcaaagagatatatacaaatggccaacagataaaattaaatactaaaaaattaccCAGAATAAGGCAGGAAATGGGAGTGgaggaa
JW-1196-85818-IntR-rc    ~~~~~~~~~~~~~~~~~~~~~~~~~~~~~~~~~~~~~~~~~~~~~~~~~~~~~~~~~~~~~~~~~~~~~~~~~ATAAGGCAGGAAATGGGAGTGGAGGAA
JW-1196-85818-R-rc       ~~~~~~~~~~~~~~~~~~~~~~~~~~~~~~~~~~~~~~~~~~~~~~~~~~~~~~~~~~~~~~~~~~~~~~~~~~~~~~~~~~~~~~~~~~~~~~~~~~~~

                                  310       320       330       340       350       360       370       380       390       400
                         ....|....|....|....|....|....|....|....|....|....|....|....|....|....|....|....|....|....|....|
Aotus_1196_[Anan_2.0]    caaaaaaagaataacaaataaaaaaa~ctaataagAA~~~~~~~~~~~~~~~~~~~~~~~~~~~~~~~~~~~~~~~~~~~~~~~~~~~~~~~~~~~~~~~
JW-1196-85818-IntR-rc    CAAAAAAAGAATAACAAATAAAAAAAACTAATAAGAAGGCCGGGCGCGGTGGCTCAAGCCTGTAATCCCAGCACTTTGGGAGGCCGAGGCGGGTGGATCA
JW-1196-85818-R-rc       ~~~~~~~~~~~~~~~~~~~~~~~~~~~~~~~~~~~~~~~~~~~~~~~~~~~~~~~~~~~~~~~~~~~~~~~~~~~~~~~~~~~~~~~~~~~~~~~~~~~~

                                  410       420       430       440       450       460       470       480       490       500
                         ....|....|....|....|....|....|....|....|....|....|....|....|....|....|....|....|....|....|....|
Aotus_1196_[Anan_2.0]    ~~~~~~~~~~~~~~~~~~~~~~~~~~~~~~~~~~~~~~~~~~~~~~~~~~~~~~~~~~~~~~~~~~~~~~~~~~~~~~~~~~~~~~~~~~~~~~~~
JW-1196-85818-IntR-rc    CGAGGTCAAGAGATCGAGACCATCCTGGTCAACATGGTGAAACCCCGTCTCTACTAATACAAAAAANNAGCTGGGCACGGTGGCGCGTGCCTGTAATCCC
JW-1196-85818-R-rc       ~~~~~~~~~~~~~~~~~~~~~~~~~~~~~~~~~~~~~~~~~~~~~~~~~~~~~~~~~~~~~~~~~~~~~~~~~~~~~~~~~~~~~~~~~~~~~~~~~~~~

                                  510       520       530       540       550       560       570       580       590       600
                         ....|....|....|....|....|....|....|....|....|....|....|....|....|....|....|....|....|....|....|
Aotus_1196_[Anan_2.0]    ~~~~~~~~~~~~~~~~~AATGATAGAACTAAGtccaattattttaataattacattaatgtAAATTGTACCTATTAAAGAATAGAGattatcaaattaaa
JW-1196-85818-IntR-rc    AGCTACTCAGGAGG~~~~~~~~~~~~~~~~~~~~~~~~~~~~~~~~~~~~~~~~~~~~~~~~~~~~~~~~~~~~~~~~~~~~~~~~~~~~~~~~~~~~~
JW-1196-85818-R-rc       ~~~~~~~~~~~~~~~~~~~~~~~~~~~~~~~~~~~~~~~~~~~~~~~~~~~~~~~~~~~~~~~~~~~~~~~~~~~~~~~~~~~~~~~~~~~~~~~~~~~~

                                  610       620       630       640       650       660       670       680       690       700
                         ....|....|....|....|....|....|....|....|....|....|....|....|....|....|....|....|....|....|....|
```

```
Aotus_1196_[Anan_2.0]          ttttaaaataagatccaactatatgttatctacaaaaaaattcactttaaatatgttgataaagttaaaagtaaaaagacGAAAAAAGATATAACATGC
JW-1196-85818-IntR-rc          ~~~~~~~~~~~~~~~~~~~~~~~~~~~~~~~~~~~~~~~~~~~~~~~~~~~~~~~~~~~~~~~~~~~~~~~~~~~~~~~~~~~~~~~~~~~~~~~~~~~~
JW-1196-85818-R-rc             ~~~~~~~~~~~~~~~~~~~~~~~~~~~~~~~~~~~~AAAAATTCACTTTAAATATGTTGATAAAGTTAAAAGTAAAAAGACGAAAAAAGATATAACATGC

                                        710       720       730       740       750       760       770       780       790       800
                               ....|....|....|....|....|....|....|....|....|....|....|....|....|....|....|....|....|....|....|....|
Aotus_1196_[Anan_2.0]          AAAtactaaccaaaagaaagctggagaagctatgctaatatcagacaaagcagactttggaacaagaaaaataactgccagccgggcgcggtggctcaag
JW-1196-85818-IntR-rc          ~~~~~~~~~~~~~~~~~~~~~~~~~~~~~~~~~~~~~~~~~~~~~~~~
JW-1196-85818-R-rc             AAATACTAACCAAAAGAAAGCTGGAGAAGCTNNAANNNTATCAGACAAAGCAGACTTTGGAACAAGAAAA~~~~~~~~~~~~~~~~~~~~~~~~~~~~~~

                                        810       820       830       840       850       860       870       880       890       900
                               ....|....|....|....|....|....|....|....|....|....|....|....|....|....|....|....|....|....|....|....|
Aotus_1196_[Anan_2.0]          cctgtaatcccagcactttgggaggccgagacgggtggatcacgaggtcaagagatcgagaccgttctggtcaacatggtgaaaccccgtctctactaat
JW-1196-85818-IntR-rc
JW-1196-85818-R-rc             ~~~~~~~~~~~~~~~~~~~~~~~~~~~~~~~~~~~~~~~~~~~~~~~~~~~~~~~~~~~~~~~~~~~~~~~~~~~~~~~~~~~~~~~~~~~~~~~~~~~~

                                        910       920       930       940       950       960       970       980       990      1000
                               ....|....|....|....|....|....|....|....|....|....|....|....|....|....|....|....|....|....|....|....|
Aotus_1196_[Anan_2.0]          acaaaaaattagctgggcatggtggcgcgtgcctgtaatcccagctactcaggaggctgaggcaggagaattgcctgaacccaggaggcggaggttgcgg
JW-1196-85818-IntR-rc
JW-1196-85818-R-rc             ~~~~~~~~~~~~~~~~~~~~~~~~~~~~~~~~~~~~~~~~~~~~~~~~~~~~~~~~~~~~~~~~~~~~~~~~~~~~~~~~~~~~~~~~~~~~~~~~~~~~

                                       1010      1020      1030      1040      1050      1060      1070      1080      1090      1100
                               ....|....|....|....|....|....|....|....|....|....|....|....|....|....|....|....|....|....|....|....|
Aotus_1196_[Anan_2.0]          tgagccgtgatcgcgccattgcactccagcctgggtaacgagcgaaacttcgcctcaaaaaaaaaaaaaaaaaaaaaaaaaagaaaaataactgcctAG
JW-1196-85818-IntR-rc
JW-1196-85818-R-rc             ~~~~~~~~~~~~~~~~~~~~~~~~~~~~~~~~~~~~~~~~~~~~~~~~~~~~~~~~~~~~~~~~~~~~~~~~~~~~~~~~~~~~~~~~NNNNNNNCCNAG

                                       1110      1120      1130      1140      1150      1160      1170      1180      1190      1200
                               ....|....|....|....|....|....|....|....|....|....|....|....|....|....|....|....|....|....|....|....|
Aotus_1196_[Anan_2.0]          GAAGAAGAACATTTTATAGTGGTAAAGGATCAATTCACCAAGATACACAGCATTTCCAACTGTGCATGTACTTTTAACAAGGTGCATGCTCCAAAATATA
JW-1196-85818-IntR-rc
JW-1196-85818-R-rc             AAAGAAGAACATTTTATAGTGGTAAAGGATCAATTCACCAAGATACA
```

Figure S21. Sequence alignment for *Alu* locus *Aotus*_1196_ 018495318.1:3327241-3328746 (JW-1196). PCR-based genotypes indicate the target *Alu* insertion is polymorphic among *A. nancymaae* individuals, also present in one of three *A. azarae* individuals, 85818, while being absent from other *Aotus* species. DNA sequencing was performed to determine if the target insertion is shared by *A. azarae* 85818. The reference genome [Anan_2.0] is shown at the top followed by *A. azarae* sample 85818. Oligonucleotide primers for PCR are highlighted in bright green at positions 274 and 1178. The target *Alu* starts at position 780 (grey highlight) and has TSDs (aagaaaaataactgcc) in yellow highlight. *A. azarae* sample 85818 lacks the target insertion as the R-rc sequence trace spans the pre-integration site. Instead, the *A. azarae* individual has a different *Alu*, a near parallel insertion, starting at position 338 (teal highlight) and running to position 514 shown in aqua highlight. Unfortunately, sequencing failed using the forward primer so only part of this novel *A. azarae Alu* element is detected. An alternative reverse PCR primer has been designed, *Aotus*_1196_azarae-R:

ccagctttcttttggttagtaTT (purple highlight) that could be used in conjunction with the existing forward primer (ATAAGGCAGGAAATGGGAGTG) shown at position 274 to potentially genotype this novel *A. azarae* derived *Alu* element separately, one that is polymorphic among *A. azarae* individuals. We did not attempt this PCR.

OLIGO	start	len	tm	gc%	any th	3' th	hairpin	seq
LEFT PRIMER	29	21	57.70	47.62	0.00	0.00	0.00	ATAAGGCAGGAAATGGGAGTG
RIGHT PRIMER	528	23	55.14	34.78	0.00	0.00	0.00	ccagctttcttttggttagtaTT

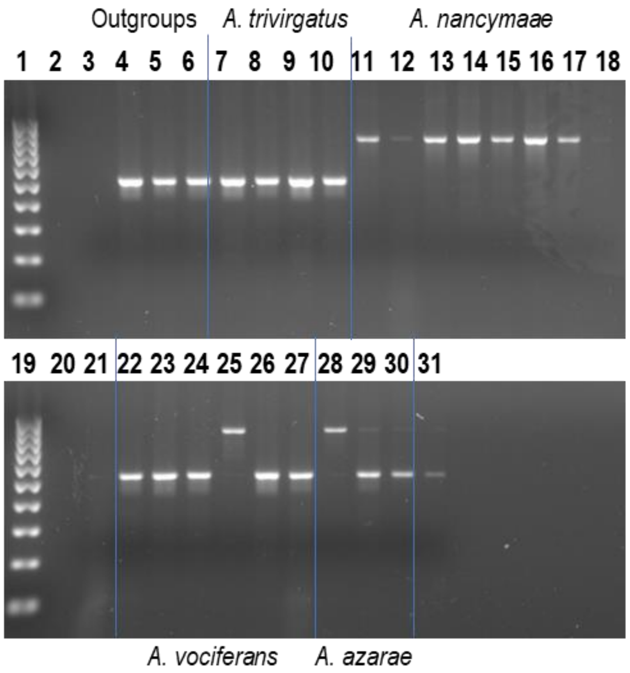

Figure S22a: *Alu* locus *Aotus*_364_ 018513626.1:9585827-9587338 (JW-364): Lanes: 1, 19- 100 bp DNA ladder, 2, 20-blank, 3-TLE (negative control), 4- Human (HeLa), 5- *Callithrix jacchus* (Common marmoset), 6- *Saimiri s. sciureus* (Common squirrel monkey), 7-10 *A. trivirgatus*, 11-18, 21 *A. nancymaae*, 22-27 *A. vociferans*, 28-30 *A. azarae*, 31-*A. lemurinus griseimembra*. Vertical blue lines superimposed on the gel image visually separate the species groups. The predicted amplicon sizes are 846/521 bp. These PCR genotypes indicate that the target *Alu* insertion is present in *A. nancymaae*, *A. azarae* as well as one *A. vociferans* individual

86218, while homozygous absent in *A. trivirgatus*. Perform DNA sequencing to confirm that *A. azarae* and *A. vociferans* share the target *Alu* insertion.

```
                                   110       120       130       140       150       160       170       180       190       200
                          ....|....|....|....|....|....|....|....|....|....|....|....|....|....|....|....|....|....|....|....|
Aotus_364_[Anan_2.0]      CTGTAGGTAGAGAGCCCGGATTTTCTTAAACAGCTTTGTATTAGTTCCTGAGAATGTGTTTGCTTGGTCCTGCCAGGCTCATCTGTCCAACACTGGAGCA
JW-364-85457-SIntR-rc     ~~~~~~~~~~~~~~~~~~~~~~~~~~~~~~~~~~~~~~~~~~~~~~~~~~~~~~~~~~~~~~~~~~~~~~~~~~~~~~~CATCTGTCCAACACTGGAGCA
JW-364-85457-F            ~~~~~~~~~~~~~~~~~~~~~~~~~~~~~~~~~~~~~~~~~~~~~~~~~~~~~~~~~~~~~~~~~~~~~~~~~~~~~~~~~~~~~~~~~~~~~~~~~~~~
JW-364-85457-IntF         ~~~~~~~~~~~~~~~~~~~~~~~~~~~~~~~~~~~~~~~~~~~~~~~~~~~~~~~~~~~~~~~~~~~~~~~~~~~~~~~~~~~~~~~~~~~~~~~~~~~~
JW-364-85457-R-rc         ~~~~~~~~~~~~~~~~~~~~~~~~~~~~~~~~~~~~~~~~~~~~~~~~~~~~~~~~~~~~~~~~~~~~~~~~~~~~~~~~~~~~~~~~~~~~~~~~~~~~
JW-364-86218-F            ~~~~~~~~~~~~~~~~~~~~~~~~~~~~~~~~~~~~~~~~~~~~~~~~~~~~~~~~~~~~~~~~~~~~~~~~~~~~~~~~~~~~~~~~~~~~~~~~~~~~
JW-364-86218-R-rc         ~~~~~~~~~~~~~~~~~~~~~~~~~~~~~~~~~~~~~~~~~~~~~~~~~~~~~~~~~~~~~~~~~~~~~~~~~~~~~~~~~~~~~~~~~~~~~~~~~~~~
JW-364-86218-SIntR        ~~~~~~~~~~~~~~~~~~~~~~~~~~~~~~~~~~~~~~~~~~~~~~~~~~~~~~~~~~~~~~~~~~~~~~~~~~~~~~~~~~~~~~~~~~~~~~~~~~~~
JW-364-86218-IntF-rc      ~~~~~~~~~~~~~~~~~~~~~~~~~~~~~~~~~~~~~~~~~~~~~~~~~~~~~~~~~~~~~~~~~~~~~~~~~~~~~~~~~~~~~~~~~~~~~~~~~~~~

                                   210       220       230       240       250       260       270       280       290       300
                          ....|....|....|....|....|....|....|....|....|....|....|....|....|....|....|....|....|....|....|....|
Aotus_364_[Anan_2.0]      ATCCCTATGTCCAGGGTGAGGTATTCTGATTGGCCAGATTGGATCTCATGATCAGATGTGTGACAAGCCAGGGACATTTCATGTTTGACAGTCCCCCAGG
JW-364-85457-SIntR-rc     ATCCCTATGTCCAGGGTGAGGTATTCTGATTGGCCAGATTGGATCTCATGATCAGATGTGTGACAAGCAAGGGACATTTCATGTTTGACAGTCCCCCAGG
JW-364-85457-F            ~~~~~~~~~~~~~~~~~~~~~~~~TCTGATTGGCCAGATTGGATCTCATGATCAGATGTGTGACAAGCAAGGGACATTTCATGTTTGACAGTCCCCCAGG
JW-364-85457-IntF         ~~~~~~~~~~~~~~~~~~~~~~~~~~~~~~~~~~~~~~~~~~~~~~~~~~~~~~~~~~~~~~~~~~~~~~~~~~~~~~~~~~~~~~~~~~~~~~~~~~~~
JW-364-85457-R-rc         ~~~~~~~~~~~~~~~~~~~~~~~~~~~~~~~~~~~~~~~~~~~~~~~~~~~~~~~~~~~~~~~~~~~~~~~~~~~~~~~~~~~~~~~~~~~~~~~~~~~~
JW-364-86218-F            ~~~~~~~~~~~~~~~~~~~~~~~~~~~GATTGGCCAGATTGGATCTCATGATCAGATGTGTGACAAGCAAGGGACATTTCATGTTTGACAGTCCCCCAGG
JW-364-86218-R-rc         ~~~~~~~~~~~~~~~~~~~~~~~~~~~~~~~~~~~~~~~~~~~~~~~~~~~~~~~~~~~~~~~~~~~~~~~~~~~~~~~~~~~~~~~~~~~~~~~~~~~~
JW-364-86218-SIntR        ~~~~~~~~~~~~~~~~~~~~~~~~~~~~~~~~~~~~~~~~~~~~~~~~~~~~~~~~~~~~~~~~~~~~~~~~~~~~~~~~~~~~~~~~~~~~~~~~~~~~
JW-364-86218-IntF-rc      ~~~~~~~~~~~~~~~~~~~~~~~~~~~~~~~~~~~~~~~~~~~~~~~~~~~~~~~~~~~~~~~~~~~~~~~~~~~~~~~~~~~~~~~~~~~~~~~~~~~~

                                   310       320       330       340       350       360       370       380       390       400
                          ....|....|....|....|....|....|....|....|....|....|....|....|....|....|....|....|....|....|....|....|
Aotus_364_[Anan_2.0]      AGCTAGAAGGAGAGGTTCCTAAAAGAGAATAGAAGAATGTATTGtctgaagaaaaaggaagggataTTAAAGGTCTGTTCACAGGTTTAAGAGGCAGAGA
JW-364-85457-SIntR-rc     AGCTAGAAGGAGAGGTTCCTAAAAGAGAATAGAAGAATGTATTGTCTGAAGAAAAAGGAAGGGATATTAAAGGTCTGTTCACAGGTTTAAGAGGCAGAGA
JW-364-85457-F            AGCTAGAAGGAGAGGTTCCTAAAAGAGAATAGAAGAATGTATTGTCTGAAGAAAAAGGAAGGGATATTAAAGGTCTGTTCACAGGTTTAAGAGGCAGAGA
JW-364-85457-IntF         ~~~~~~~~~~~~~~~~~~~~~~~~~~~~~~~~~~~~~~~~~~~~~~~~~~~~~~~~~~~~~~~~~~~~~~~~~~~~~~~~~~~~~~~~~~~~~~~~~~~~
JW-364-85457-R-rc         ~~~~~~~~~~~~~~~~~~~~~~~~~~~~~~~~~~~~~~~~~~~~~~~~~~~~~~~~~~~~~~~~~~~~~~~~~~~~~~~~~~~~~~~~~~~~~~~~~~~~
JW-364-86218-F            AGCTAGAAGGAGAGGTTCCTAAAAGAGAATAGAAGAATGTATTGTCTGAAGAAAAAGGAAGGGATATTAAAGGTCTGTTCACAGGTTTAGGAGGCAGAGA
JW-364-86218-R-rc         ~~~~~~~~~~~~~~~~~~~~~~~~~~~~~~~~~~~~~~~~~~~~~~~~~~~~~~~~~~~~~~~~~~~~~~~~~~~~~~~~~~~~~~~~~~~~~~~~~~~~
JW-364-86218-SIntR        ~~~~~~~~~~~~~~~~~~~~~~~~~~~~~~~~~~~~~~~~~~~~~~~~~~~~~~~~~~~~~~~~~~~~~~~~~~~~~~~~~~~~~~~~~~~~~~~~~~~~
JW-364-86218-IntF-rc      ~~~~~~~~~~~~~~~~~~~~~~~~~~~~~~~~~~~~~~~~~~~~~~~~~~~~~~~~~~~~~~~~~~~~~~~~~~~~~~~~~~~~~~~~~~~~~~~~~~~~

                                   410       420       430       440       450       460       470       480       490       500
                          ....|....|....|....|....|....|....|....|....|....|....|....|....|....|....|....|....|....|....|....|
Aotus_364_[Anan_2.0]      CGTTGGCTTGGGGCAGAAAGAATATTGATTTGGGAAATAGGGGATCACACTAGAAAGCAAGCTTCATGACgccaaagtttttatttattcactacTTCT~
JW-364-85457-SIntR-rc     CGTTGGCTTGGGGCAGAAAGAATATTGATTTGGGAAATAGGGGATCACACTAGAAAGCAAGCTTCATGACGCCAAAGTTTTTATTTATTCACTACTTCT~
JW-364-85457-F            CGTTGGCTTGGGGCAGAAAGAATATTGATTTGGGAAATAGGGGATCACACTAGAAAGCAAGCTTCATGACGCCAAAGTTTTTATTTATTCACTACTTCT~
JW-364-85457-IntF         ~~~~~~~~~~~~~~~~~~~~~~~~~~~~~~~~~~~~~~~~~~~~~~~~~~~~~~~~~~~~~~~~~~~~~~~~~~~~~~~~~~~~~~~~~~~~~~~~~~~~
JW-364-85457-R-rc         ~~~~~~~~~~~~~~~~~~~~~~~~~~~~~~~~~~~~~~~~~~~~~~~~~~~~~~~~~~~~~~~~~~~~~~~~~~~~~~~~~~~~~~~~~~~~~~~~~~~~
JW-364-86218-F            CATTGGCTTGGGGCAGAAAGAATATTGATTTGGGAAATAGGGGATCACACTAGAAAGCAAGCTTCATGACGCCGAAGTTTTTATTTATTCACTACTTCTT
JW-364-86218-R-rc         ~~~~~~~~~~~~~~~~~~~~~~~~~~~~~~~~~~~~~~~~~~~~~~~~~~~~~~~~~~~~~~~~~~~~~~~~~~~~~~~~~~~~~~~~~~~~~~~~~~TT
JW-364-86218-SIntR        ~~~~~~~~~~~~~~~~~~~~~~~~~~~~~~~~~~~~~~~~~~~~~~~~~~~~~~~~~~~~~~~~~~~~~~~~~~~~~~~~~~~~~~~~~~~~~~~~~~~~
JW-364-86218-IntF-rc      ~~~~~~~~~~~~~~~~~~~~~~~~~~~~~~~~~~~~~~~~~~~~~~~~~~~~~~~~~~~~~~~~~~~~~~~~~~~~~~~~~~~~~~~~~~~~~~~~~~T
```

```
                                      510       520       530       540       550       560       570       580       590       600
                             ....|....|....|....|....|....|....|....|....|....|....|....|....|....|....|....|....|....|....|....|
Aotus_364_[Anan_2.0]         ~~~~~~~~~~~~~~~~~~~~~~~~~~~~~~~~~~~~~~~~~~~~~~~~~~~~~~~~~~~~~~~~~~~~~~~~~~~~~~~~~~~~~~~~~~~~~~~~
JW-364-85457-SIntR-rc        ~~~~~~~~~~~~~~~~~~~~~~~~~~~~~~~~~~~~~~~~~~~~~~~~~~~~~~~~~~~~~~~~~~~~~~~~~~~~~~~~~~~~~~~~~~~~~~~~
JW-364-85457-F               ~~~~~~~~~~~~~~~~~~~~~~~~~~~~~~~~~~~~~~~~~~~~~~~~~~~~~~~~~~~~~~~~~~~~~~~~~~~~~~~~~~~~~~~~~~~~~~~~
JW-364-85457-IntF            ~~~~~~~~~~~~~~~~~~~~~~~~~~~~~~~~~~~~~~~~~~~~~~~~~~~~~~~~~~~~~~~~~~~~~~~~~~~~~~~~~~~~~~~~~~~~~~~~
JW-364-85457-R-rc            ~~~~~~~~~~~~~~~~~~~~~~~~~~~~~~~~~~~~~~~~~~~~~~~~~~~~~~~~~~~~~~~~~~~~~~~~~~~~~~~~~~~~~~~~~~~~~~~~
JW-364-86218-F               TTTTTTTTTTTTTTTTTTTTTTTTTTTT
JW-364-86218-R-rc            TTTTTTTTTTTTTTTTTTTTTTTTTTTTGGAGACGGAGTTTCGCTCTTGTTACCCAGGCTGGAGTGCAATGGCGCGATCTCGGCTCACTGCAACCTCCGC
JW-364-86218-SIntR           ~~~~~~~~~~~~~~~~~~~~~~~~~~~~~~~~~~~~~~~~~~~~~~~~~~~~~~~~~~~~~~~~~~~~~~~~~~~~~~~~~~~~~~~~~~~~~~~~
JW-364-86218-IntF-rc         TTTTTTTTTTTTTTTTTTTTTTTTTTTTGGAGACGGAGTTTCGCTCTTGTTACCCAGGCTGGAGTGCAATGGCGCGATCTCGGCTCACTGCAACCTCCGC

                                      610       620       630       640       650       660       670       680       690       700
                             ....|....|....|....|....|....|....|....|....|....|....|....|....|....|....|....|....|....|....|
Aotus_364_[Anan_2.0]         ~~~~~~~~~~~~~~~~~~~~~~~~~~~~~~~~~~~~~~~~~~~~~~~~~~~~~~~~~~~~~~~~~~~~~~~~~~~~~~~~~~~~~~~~~~~~~~~~
JW-364-85457-SIntR-rc        ~~~~~~~~~~~~~~~~~~~~~~~~~~~~~~~~~~~~~~~~~~~~~~~~~~~~~~~~~~~~~~~~~~~~~~~~~~~~~~~~~~~~~~~~~~~~~~~~
JW-364-85457-F               ~~~~~~~~~~~~~~~~~~~~~~~~~~~~~~~~~~~~~~~~~~~~~~~~~~~~~~~~~~~~~~~~~~~~~~~~~~~~~~~~~~~~~~~~~~~~~~~~
JW-364-85457-IntF            ~~~~~~~~~~~~~~~~~~~~~~~~~~~~~~~~~~~~~~~~~~~~~~~~~~~~~~~~~~~~~~~~~~~~~~~~~~~~~~~~~~~~~~~~~~~~~~~~
JW-364-85457-R-rc            ~~~~~~~~~~~~~~~~~~~~~~~~~~~~~~~~~~~~~~~~~~~~~~~~~~~~~~~~~~~~~~~~~~~~~~~~~~~~~~~~~~~~~~~~~~~~~~~~
JW-364-86218-F
JW-364-86218-R-rc            CTCCTGGGTTCAGGCAATTCTCCTGCCTCAGCCTCCCGAGTAGCTGGGATTACAGACACGCGCCACCATGCCCAGCTAATTTTTTTGTATTAGTAGAGAC
JW-364-86218-SIntR           ~~~~~~~~~~~~~~~~~~~~~CCTGCCTCAGCCTCCCGAGTAGCTGGGATTACAGACACGCGCCACCATGCCCAGCTAATTTTTTTGTATTAGTAGAGAC
JW-364-86218-IntF-rc         CTCCTGGGTTCAGGCAATTCTCCTGCCTCAGCCTCCCGAGTAGCTGGGATTACAGACACGCGCCACCATGCCCAGCTAATTTTTTTGTATTAGTAGAGAC

                                      710       720       730       740       750       760       770       780       790       800
                             ....|....|....|....|....|....|....|....|....|....|....|....|....|....|....|....|....|....|....|
Aotus_364_[Anan_2.0]         ~~~~~~~~~~~~~~~~~~~~~~~~~~~~~~~~~~~~~~~~~~~~~~~~~~~~~~~~~~~~~~~~~~~~~~~~~~~~~~~~~~~~~~~~~~~~~~~~
JW-364-85457-SIntR-rc        ~~~~~~~~~~~~~~~~~~~~~~~~~~~~~~~~~~~~~~~~~~~~~~~~~~~~~~~~~~~~~~~~~~~~~~~~~~~~~~~~~~~~~~~~~~~~~~~~
JW-364-85457-F               ~~~~~~~~~~~~~~~~~~~~~~~~~~~~~~~~~~~~~~~~~~~~~~~~~~~~~~~~~~~~~~~~~~~~~~~~~~~~~~~~~~~~~~~~~~~~~~~~
JW-364-85457-IntF            ~~~~~~~~~~~~~~~~~~~~~~~~~~~~~~~~~~~~~~~~~~~~~~~~~~~~~~~~~~~~~~~~~~~~~~~~~~~~~~~~~~~~~~~~~~~~~~~~
JW-364-85457-R-rc            ~~~~~~~~~~~~~~~~~~~~~~~~~~~~~~~~~~~~~~~~~~~~~~~~~~~~~~~~~~~~~~~~~~~~~~~~~~~~~~~~~~~~~~~~~~~~~~~~
JW-364-86218-F
JW-364-86218-R-rc            GGGGTTTCACCATGTCGACCAGGATGGTCTCGATCTCTTGACCTCGTGATCCACCCGCCTCGGCCTCCCAAAGTGCTGGGATTACAGGCTTGAGCCACCG
JW-364-86218-SIntR           GGGGTTTCACCATGTCGACCAGGATGGTCTCGATCTCTTGACCTCGTGATCCACCCGCCTCGGCCTCCCAAAGTGCTGGGATTACAGGCTTGAGCCACCG
JW-364-86218-IntF-rc         GGGGTTTCACCATGTCGACCAGGATGGTCTCGATCTCTTGACCTCGTGATC

                                      810       820       830       840       850       860       870       880       890       900
                             ....|....|....|....|....|....|....|....|....|....|....|....|....|....|....|....|....|....|....|
Aotus_364_[Anan_2.0]         ~~~~~~~~~~~~~~~~~~~~~~~~AAAATAAGGCTTGGCACAAAGTAAACACTCATTAATTGAATGAGTGAAATATTCAAATGGACCATGGAAATTCAGG
JW-364-85457-SIntR-rc        ~~~~~~~~~~~~~~~~~~~~~~~~AAAATAAGGCTTGGCACAAAGTAAACACTCATTAATTGAATGAGTGAAATATTCAAATGGACCATGGAAATTCAGG
JW-364-85457-F               ~~~~~~~~~~~~~~~~~~~~~~~~AAAATAAGGCTTGGCACAAAGTAAACACTCATTAATTGAATGAGTGAAATATTCAAATGGACCATGGAAATTCAGG
JW-364-85457-IntF            ~~~~~~~~~~~~~~~~~~~~~~~~~~~~~~~~~~~~~~~~~~~~~~~~~~~~~~~~~~~~~~~~~~~~~~~~~~~~~~~~~~~~~~~~~~~~~~~~
JW-364-85457-R-rc            ~~~~~~~~~~~~~~~~~~~~~~~~~~~~~~~~~~~~~~~~~~~~~~~~~~~~~~~~~~~~~~~~~~~~~~~~~~~~~~~~~~~~~~~~~~~~~~~~
JW-364-86218-F
JW-364-86218-R-rc            CGCCCGGCCTTATTCACTACTTCTAAAATAAGGCTTGGCACAAAGTAAACACTCATTAATTGAATGAGTGAAATATTCAAATGGACCATGGAAATTCAGG
JW-364-86218-SIntR           CGCCCGGCCTTATTCACTACTTCTAAAATAAGGCTTGGCACAAAGTAAACACTCATTAATTGAATGAGTGAAATATTCAAATGGACCATGGAAATTCAGG
JW-364-86218-IntF-rc
```

```
                                     910       920       930       940       950       960       970       980       990       1000
                                ....|....|....|....|....|....|....|....|....|....|....|....|....|....|....|....|....|....|....|....|
Aotus_364_[Anan_2.0]            ATTTACTTGAAAATATCAtgaataggccgggcgcggtggctcacgcctgtaatcccagcactttgggaggccgaggcgggtggatcacgaggtcaagaga
JW-364-85457-SIntR-rc           ATTTACTTGAAAATATCATGAATAGGCCGGGCGCGGTGGCTCACGCCTGTAATCCCAGCACTTTGGGAGGCCGAGGCGGGTGGATCACGAGGTCAAGAGA
JW-364-85457-F                  ATTTACTT
JW-364-85457-IntF               ~~~~~~~~~~~~~~~~~~~~~~~~~~~~~~~~~~~~~~~~~~~~~~~~~~~~~~~~~~~~~~~~~~~~~~~~~~~~~~~~~~~~~~~~GAGGTCA~GAGA
JW-364-85457-R-rc               ~~~~~~~~~~~~~~~~~~~~~~~~~~~~~~~~~~~~~~~~~~~~~~~~~~~~~~~~~~~~~~~~~~~~~~~~~~~~~~~~~~~~~~~~~~~~~~~~~~~~
JW-364-86218-F
JW-364-86218-R-rc               ATTTACTTGAAAATATCATGAATA~~~~~~~~~~~~~~~~~~~~~~~~~~~~~~~~~~~~~~~~~~~~~~~~~~~~~~~~~~~~~~~~~~~~~~~~~~~~
JW-364-86218-SIntR              ATTTACTTGAAAATATCATGAATA~~~~~~~~~~~~~~~~~~~~~~~~~~~~~~~~~~~~~~~~~~~~~~~~~~~~~~~~~~~~~~~~~~~~~~~~~~~~
JW-364-86218-IntF-rc

                                     1010      1020      1030      1040      1050      1060      1070      1080      1090      1100
                                ....|....|....|....|....|....|....|....|....|....|....|....|....|....|....|....|....|....|....|....|
Aotus_364_[Anan_2.0]            tcgagaccatcctggtcaacaaggtgaaaccccgtctctactaaaaatacaaaaaattagctgggcatggtggcgcgtgcctgtaatcccagctactcag
JW-364-85457-SIntR-rc           TCGAGACCATCCTGGTCAACAAGGTGAAACCTCGTCTCTACTAAAAATACAAAAAATTAGCTGGGCATGGTGGCGCGTGCCTGTAATCCCAGCTACTCAG
JW-364-85457-F
JW-364-85457-IntF               TCGAGACCATCCTGGTCAACAAGGTGAAACCTCGTCTCTACTAAAAATACAAAAAATAACCTGGGCATGGTGGCGCGTGCCTGTAATCCCAGCTACTCAG
JW-364-85457-R-rc               ~~~~~~~~~~~~~~~~~~~~~~~~~~~~~~~~~~~~~~~~~~~~~~~~~~~~~~~~~~~~~~~~~~~~~~~~~~~~~~~~~~~~~~~~~~~~~~~~~~~~
JW-364-86218-F
JW-364-86218-R-rc               ~~~~~~~~~~~~~~~~~~~~~~~~~~~~~~~~~~~~~~~~~~~~~~~~~~~~~~~~~~~~~~~~~~~~~~~~~~~~~~~~~~~~~~~~~~~~~~~~~~~~
JW-364-86218-SIntR              ~~~~~~~~~~~~~~~~~~~~~~~~~~~~~~~~~~~~~~~~~~~~~~~~~~~~~~~~~~~~~~~~~~~~~~~~~~~~~~~~~~~~~~~~~~~~~~~~~~~~
JW-364-86218-IntF-rc

                                     1110      1120      1130      1140      1150      1160      1170      1180      1190      1200
                                ....|....|....|....|....|....|....|....|....|....|....|....|....|....|....|....|....|....|....|....|
Aotus_364_ [Anan_2.0]           gaggctgaggcaggagaattgcctgaacccaggaggcggaggttgcggtgagccgagatcgcgccattgcactccagcctgggtaacaagagcgaaactc
JW-364-85457-SIntR-rc           GAGGCTGAGGC
JW-364-85457-F
JW-364-85457-IntF               GAGGCTGAGGCAGGAGAATTGCCTGAACCCAGGAGGCGGAGGTTGCGGTGAGCCGAGATCGCGCCATTGCACTCCAGCCTGGGTAACAAGAGCGAAACTC
JW-364-85457-R-rc               ~~~~~~~~~~~~~~~~~~~~~~~~~~~~~~~~~~~~~~~~~~~~~~~~~~~~~~~~~~~~~~~~~~~~~~~~~~~~~~~~~~~~~~~~~~~~~~~~~~~~
JW-364-86218-F
JW-364-86218-R-rc               ~~~~~~~~~~~~~~~~~~~~~~~~~~~~~~~~~~~~~~~~~~~~~~~~~~~~~~~~~~~~~~~~~~~~~~~~~~~~~~~~~~~~~~~~~~~~~~~~~~~~
JW-364-86218-SIntR              ~~~~~~~~~~~~~~~~~~~~~~~~~~~~~~~~~~~~~~~~~~~~~~~~~~~~~~~~~~~~~~~~~~~~~~~~~~~~~~~~~~~~~~~~~~~~~~~~~~~~
JW-364-86218-IntF-rc

                                     1210      1220      1230      1240      1250      1260      1270      1280      1290      1300
                                ....|....|....|....|....|....|....|....|....|....|....|....|....|....|....|....|....|....|....|....|
Aotus_364_[Anan_2.0]            cgtctcaaaaaaaaaaaaaaaagaaagaaaaaagaaaatatcatgaaTATCCTGGGGATGATATGGTAGATTATAAAATGCTCACAATTGTCCACCCTTT
JW-364-85457-SIntR-rc
JW-364-85457-F
JW-364-85457-IntF               CGTCTCAAAAAAAAA
JW-364-85457-R-rc               ~~~~~~~AAAAAAAAAAAAAAAAAAAAAAAAAAGAAAATATCATGAATATCCTGGGGATGATATGGTAGATTATAAAATGCTCACAA
JW-364-86218-F
JW-364-86218-R-rc               ~~~~~~~~~~~~~~~~~~~~~~~~~~~~~~~~~~~~~~~~~~~~~~~~~TCCTGGGGATGATATGGTAGATTATAAAATGCCCACAATTGTCCACC
JW-364-86218-SIntR              ~~~~~~~~~~~~~~~~~~~~~~~~~~~~~~~~~~~~~~~~~~~~~~~~~TCCTGGGGATGATATGGTAGATTATAAAATGCCCACAATTGTCCACCCTTT
JW-364-86218-IntF-rc
```

```
                                          1310      1320      1330      1340      1350      1360      1370      1380      1390      1400
                                  ....|....|....|....|....|....|....|....|....|....|....|....|....|....|....|....|....|....|....|....|
Aotus_364_[Anan_2.0]              CTATAAAGAGGTGGAGTCTATTTCCCCATCCTTGCATCTGAGTTGGGAACATGACCAACTTTGGGCAATGAGACATTAATAAACATCACTGGAGCAGAAG
JW-364-85457-SIntR-rc
JW-364-85457-F
JW-364-85457-IntF
JW-364-85457-R-rc
JW-364-86218-F
JW-364-86218-R-rc
JW-364-86218-SIntR                CTATAAAGAGGTGGAGTCTATTTCCCCATCCTTGCATCTGAGTTGANTCTACTAATACAAAAAAATTAGCTGGGCATGGTGGCGCGTGTCTGTAATCCCA
JW-364-86218-IntF-rc

                                          1410      1420      1430      1440      1450      1460      1470      1480      1490      1500
                                  ....|....|....|....|....|....|....|....|....|....|....|....|....|....|....|....|....|....|....|
Aotus_364_[Anan_2.0]              CTTGAGACACACTCATGAATGCAGGTCATGCAGGGCTTGTCCTCCTGCAGCTCTTGGATCTCCGCCACCGTTTGAATGAGCCTCAGCTAGcctgctggag
JW-364-85457-SIntR-rc
JW-364-85457-F
JW-364-85457-IntF
JW-364-85457-R-rc
JW-364-86218-F
JW-364-86218-R-rc
JW-364-86218-SIntR                GCTACTCGGGAGGCTGAGGCAGGAGAATTGCCTGAACCCAGGAGGCGGAGGTTGCGGTGAGCCGAGAA
JW-364-86218-IntF-rc
```

Figure S22b. Sequence alignment for *Alu* locus *Aotus*_364_ 018513626.1:9585827-9587338 (JW-364). PCR-based genotypes indicate the target *Alu* insertion is present in *A. nancymaae*, *A. azarae* as well as one *A. vociferans* individual 86218, while homozygous absent in *A. trivirgatus*. The reference genome [Anan_2.0] is shown at the top followed by *A. azarae* sample 85457 and *A. vociferans* sample 86218. Oligonucleotide primers for PCR are highlighted in bright green at positions 175 and 1325. The target *Alu* starts at position 925 (grey highlight) and has TSDs (GAAAATATCAtgaata) in yellow highlight and is shared by *A. azarae* sample 85457. However, the *A. vociferans* 86218 filled site amplicon lacks the target insertion as sequence traces span the pre-integration site. Instead, *A. vociferans* has a different *Alu*, a near parallel insertion, starting at position 810 (teal highlight) and running in the reverse orientation back to the T-tail (reverse compliment of an A-tail) at position 499. TSDs (TTATTCACTACTTCT) are shown in aqua highlight before sequence traces once again align with the reference genome. An alternative reverse PCR primer has been designed, *Aotus*_364_vociferans-R: GAGTGTTTACTTTGTGCCAAGC (purple highlight) that could be used in conjunction with the existing forward primer shown at position 175 (AGGCTCATCTGTCCAACACTG) to potentially genotype this novel *A. vociferans* derived *Alu* element separately. We did not attempt this PCR as the locus is not parsimony informative within our sample set. In addition, the *A. vociferans* 86218 sequence trace has the 2nd half of another *Alu* element (confirmed by RepeatMasker) starting at the mid-A-rich region at about position 1350 where the trace no longer matches the [Anan_2.0] reference genome, marked in <u>underline</u> text until the trace terminates.

```
                                  310       320       330       340       350       360       370       380       390       400
                          ....|....|....|....|....|....|....|....|....|....|....|....|....|....|....|....|....|....|....|....|
Owl_2LS_120_[Anan_2.0]    ctgaggcgggagactcaattgaatcagggaggcggaggctgcagtgagctgagatcgtgccagcctgggcgacagagtaagaccctgcctcaaaaacaaa
LR-120-85962-SIntR-rc     ~~~~~TCGGGAGACTCAATTGAATCAGGGAGGCGGAGGCTGCAGTGAGCTGAGATCGTTCCAGCCTGGGCGACAGAGTAAGACCCTGCCTCAAAAACAAA
LR-120-85962-F            ~~~~~~~~~~~~~~~~~~~~~~~~~~~~~~~~~~~~~~~~~~~~~~~~~~~~~~~~~~~~~~~CCTGGGCGACAGAGTAAGACCCTGCCTCAAAAACAAA
LR-120-85962-IntF         ~~~~~~~~~~~~~~~~~~~~~~~~~~~~~~~~~~~~~~~~~~~~~~~~~~~~~~~~~~~~~~~~~~~~~~~~~~~~~~~~~~~~~~~~~~~~~~~~~~~~
LR-120-85962-R-rc         ~~~~~~~~~~~~~~~~~~~~~~~~~~~~~~~~~~~~~~~~~~~~~~~~~~~~~~~~~~~~~~~~~~~~~~~~~~~~~~~~~~~~~~~~~~~~~~~~~~~~

                                  410       420       430       440       450       460       470       480       490       500
                          ....|....|....|....|....|....|....|....|....|....|....|....|....|....|....|....|....|....|....|....|
Owl_2LS_120_[Anan_2.0]    acaa~~~~~~~~~~~~~~~~~~~~~~~~~~~~~~~~~~~~~~~~~~~~~~~~~~~~~~~~~~~~~~~~~~~~~~~~~~~~~~~~~~~~~~~~~~~~
LR-120-85962-SIntR-rc     ACAAGGCCGGGCGCGGTGGCTCAAGCCTGTAATCCCAGCACTTTGGGAGGCCGAGGCGGGTGGATCACGAGGTCAAGAGATCGAGACCATCCTGGTCAAC
LR-120-85962-F            ACAAGGC
LR-120-85962-IntF         ~~~~~~~~~~~~~~~~~~~~~~~~~~~~~~~~~~~~~~~~~~~~~~~~~~~~~~~~~~~~~~~~~~~~GAGGTCAAGAGATCGAGACCATCCTGGTCAAC
LR-120-85962-R-rc         ~~~~~~~~~~~~~~~~~~~~~~~~~~~~~~~~~~~~~~~~~~~~~~~~~~~~~~~~~~~~~~~~~~~~~~~~~~~~~~~~~~~~~~~~~~~~~~~~~~~~

                                  510       520       530       540       550       560       570       580       590       600
                          ....|....|....|....|....|....|....|....|....|....|....|....|....|....|....|....|....|....|....|....|
Owl_2LS_120_[Anan_2.0]    ~~~~~~~~~~~~~~~~~~~~~~~~~~~~~~~~~~~~~~~~~~~~~~~~~~~~~~~~~~~~~~~~~~~~~~~~~~~~~~~~~~~~~~~~~~~~~~~~~~~~
LR-120-85962-SIntR-rc     ATTGTGAAACCCCGTCTCTACTAATACAAAAAAATTAGCTGGGCACGGTGGCGTGTGCCTGTAATCCCAGCTACTCAGGAGG
LR-120-85962-F
LR-120-85962-IntF         ATTGTGAAACCCCC~~~~~~~~~
LR-120-85962-R-rc         ~~~~~~~~~~~~~~~~~~~~~~~~~~~~~~~~~~~~~~~~~~~~~~~~~~~~~~~~~~~~~~~~~~~~~~~~~~~~~~~~~~~~~~~~~~~~~~~~~~~~

                                  610       620       630       640       650       660       670       680       690       700
                          ....|....|....|....|....|....|....|....|....|....|....|....|....|....|....|....|....|....|....|....|
Owl_2LS_120_[Anan_2.0]    ~~~~~~~~~~~~~~~~~~~~~~~~~~~aacaaaacagaacatacAATAAACACTAACCTCCACTTCACTTGTTTTCTCTTAATACCTTAATATATATGTT
LR-120-85962-SIntR-rc
LR-120-85962-F
LR-120-85962-IntF
LR-120-85962-R-rc         ~~~~~~~~~~~~~~~~~~~~~~~~~~~~~~~~~~~~~~~~~~~~~~~~~~~~~~~~~~~~~~~~~~~~~~~~~~~~~~~~~~~~~~~~~~~~~~~~~~~~

                                  710       720       730       740       750       760       770       780       790       800
                          ....|....|....|....|....|....|....|....|....|....|....|....|....|....|....|....|....|....|....|....|
Owl_2LS_120_[Anan_2.0]    GGAGGCCACTATATCAGTGTATAGAGATTTTCATCTACCTCATTCCTTTCTAGAGTTGCATAGTATTTTTTTATGTGGATGTATTATAGTTTCTTTCAGC
LR-120-85962-SIntR-rc
LR-120-85962-F
LR-120-85962-IntF
LR-120-85962-R-rc         ~~~~~~~~~~~~~~~~~~~~~~~~~~~~~~~~~~~~~~~~~~~~~~~~~~~~~~~~~~~~~~~~~~~~~~~~~~~~~~~~~~~~~~~~~~~~~~~~~~~~

                                  810       820       830       840       850       860       870       880       890       900
                          ....|....|....|....|....|....|....|....|....|....|....|....|....|....|....|....|....|....|....|....|
Owl_2LS_120_[Anan_2.0]    CAGTCTTATTTAAGAAcatttggggccgggcgcggtggctcacgcctgtaatcccagcacttagggaggccgaggcgggtggatcacgaggtcaagagat
LR-120-85962-SIntR-rc
LR-120-85962-F
LR-120-85962-IntF
LR-120-85962-R-rc         ~~~~~~~~~~~~~~~~~~~~~~~~~~~~~~~~~~~~~~~~~~~~~~~~~~~~~~~~~~~~~~~~~~~~~~~~~~~~~~~~~~~~~~~~~~~~~~~~~~~~
```

```
                               910       920       930       940       950       960       970       980       990      1000
                      ....|....|....|....|....|....|....|....|....|....|....|....|....|....|....|....|....|....|....|....|
Owl_2LS_120_[Anan_2.0] cgagaccatcctggtcaacatggtgaaaccccgtctctactaaaaatacaaaaaattagctgggcatggtggcgcgtgcctgtaatcccagctactcagg
LR-120-85962-SIntR-rc
LR-120-85962-F
LR-120-85962-IntF
LR-120-85962-R-rc      ~~~~~~~~~~~~~~~~~~~~~~~~~~~~~~~~~~~~~~~~~~~~~~~~~~~~~~~~~~~~~~~~~~~~~~~~~~~~~~~~~~~~~~~~~~~~~~~~~~~~

                              1010      1020      1030      1040      1050      1060      1070      1080      1090      1100
                      ....|....|....|....|....|....|....|....|....|....|....|....|....|....|....|....|....|....|....|
Owl_2LS_120_[Anan_2.0] aggctgaggcaggagaattgcctgaacccaggaggcggaggttgcgatgagccgagatcgcgccattgcactccagcctgggcaacaagagcgaaactcc
LR-120-85962-SIntR-rc
LR-120-85962-F
LR-120-85962-IntF
LR-120-85962-R-rc      ~~~~~~~~~~~~~~~~~~~~~~~~~~~~~~~~~~~~~~~~~~~~~~~~~~~~~~~~~~~~~~~~~~~~~~~~~~~~~~~~~~~~~~~~~~~~~~~~~~~~

                              1110      1120      1130      1140      1150      1160      1170      1180      1190      1200
                      ....|....|....|....|....|....|....|....|....|....|....|....|....|....|....|....|....|....|....|
Owl_2LS_120_[Anan_2.0] gtctcaaaaaaaaaaaaaaaagaacatttgggATGGGTGTAGCCTTTTGCTATAATGAACAGTATCActgtttccctttccctctcGGTGTACTCGGAGA
LR-120-85962-SIntR-rc
LR-120-85962-F
LR-120-85962-IntF
LR-120-85962-R-rc      ~~~~~~~~~~~~~~~~~~~~~~~~~~~~~~~~~~~~~~~~~~~~~~~~~~~~~~~~~~~~~~~~~~~~~~~~~~~~~~~~~~~~TCGGTGTACTCGGAGA

                              1210      1220      1230      1240      1250      1260      1270      1280      1290      1300
                      ....|....|....|....|....|....|....|....|....|....|....|....|....|....|....|....|....|....|....|
Owl_2LS_120_[Anan_2.0] AGAGTGGTACCCTGCCCTACTGCCACCTGAGAATCAGACGACTGCCCTGTTTCCCCAGACTGGCCCTAGGAgtatctttcattcattcaacgagCATTAG
LR-120-85962-SIntR-rc
LR-120-85962-F
LR-120-85962-IntF
LR-120-85962-R-rc      AGAGTGGTACCCTGCCCTACTGCCACCTGAGAATCAGACGACTGCCCTGTTTCCCCAGACTGGCCCTAGGAGTATCTTTCATTCATTCAACGAGCATTAG

                              1310      1320      1330      1340      1350      1360      1370      1380      1390      1400
                      ....|....|....|....|....|....|....|....|....|....|....|....|....|....|....|....|....|....|....|
Owl_2LS_120_[Anan_2.0] CGCCACAGGCGTGATTGGCGCTGTGCTGGGATCACAGTGGCGACAGAGACAAACCCAGTCCCTACTCTCATGGAACACACGGTCACAGACATCATAAAAC
LR-120-85962-SIntR-rc
LR-120-85962-F
LR-120-85962-IntF
LR-120-85962-R-rc      CGCCACCGGCGTGATTGGCGCTGTGCTGGGATCACAGTGGCGAC
```

Figure S23. Sequence alignment for *Alu* locus Owl_2LS_120_018512055.1:2444349-2445849 (LR-120). PCR based genotypes indicate the target *Alu* is restricted to *A. nancymaae*, with the exception of *A. vociferans* sample 85962 who appears heterozygous for the insertion (1/0). The reference genome [Anan_2.0] is shown at the top, the target *Alu* starts at position 824 (grey highlight) and is flanked by TSDs AAGAACATTTGG in yellow highlight. Sequencing reveals that *A. vociferans* 85962 has a different *Alu*, a near parallel insertion, starting at position 405 (teal highlight) and extending about 178 bp into the *Alu* sequence before terminating. Oligonucleotide primers for PCR are shown in bright green highlight. The SIntR-rc sequence that provides 5' flanking sequence. The

reverse primer sequence does not reach the A-tail. The target *Alu* insertion is likely restricted to *A. nancymaae*, however, this genomic region is very repeat dense so we recommend not using this locus for *Aotus* phylogeny due to the potential for confusing PCR amplicons. We have designed an alternative reverse primer, Owl_2LS_120-A.voc.-R:CAAGTGAAGTGGAGGTTAGTGT, in purple highlight to use in conjunction with the existing forward primer to potentially genotype this novel *A. vociferans Alu* element separately. These primers were confirmed to have only one match within this repeat dense genomic landscape.

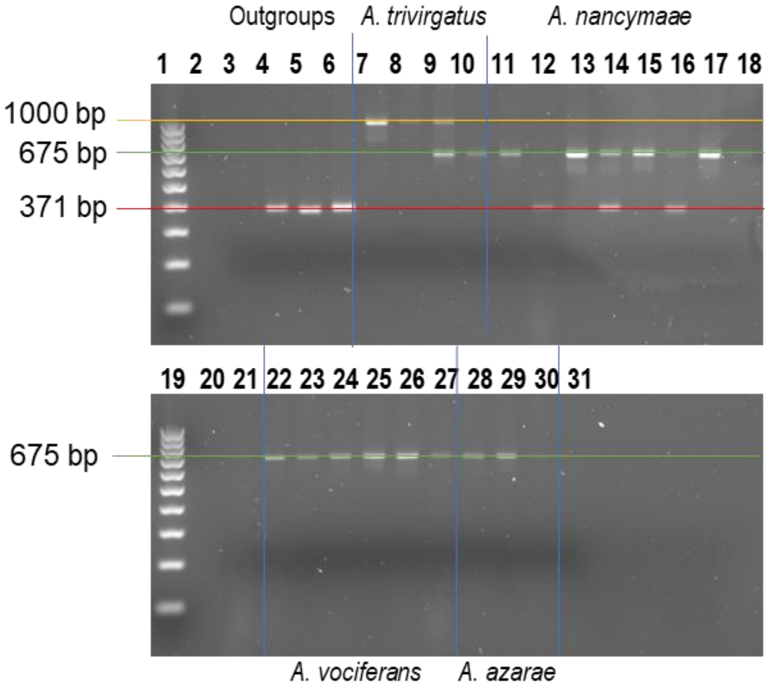

Figure S24a: *Alu* locus *Aotus*_68_018491627.1:7167631-7169129 (JW-68): Lanes: 1, 19- 100 bp DNA ladder, 2, 20-blank, 3- TLE (negative control), 4- Human (HeLa), 5- *Callithrix jacchus* (Common marmoset), 6- *Saimiri s. sciureus* (Common squirrel monkey), 7-10 *A. trivirgatus*, 11-18, 21 *A. nancymaae*, 22-27 *A. vociferans*, 28-30 *A. azarae*, 31-*A. lemurinus griseimembra.* The predicted PCR amplicon sizes are 675 bp (green line) for *Alu* present and 371 bp (red line) for *Alu* absent. *A. nancymaae* individuals appear polymorphic for presence / absence of the target insertion while homozygous present in other *Aotus* species. *A. trivirgatus* appears to harbor a possible 2nd *Alu* inserion (~1000 bp; yellow line) that is not present in other *Aotus* species. Perform DNA sequencing to identify this amplicon (See Figure S24b).

```
                                  210       220       230       240       250       260       270       280       290       300
                         ....|....|....|....|....|....|....|....|....|....|....|....|....|....|....|....|....|....|....|....|
Aotus_68_[Anan_2.0]      GGgggatttttatataatttattggaATGAACATGCCTAACCACTAGGAAATGGCAACCTGAAGTTGGACTGTAGGACACTGGGATTGGGTGGAGAATTT
JS-68-85818-SIntR-rc     ~~~~~~~~~~~~~~~~~~~~~~~~~~~~~~~~~~~~~~~~~~~~~~~~~~~~~~~~~~~~~~~~~~~~~~~~~~~~~~~~~~~~~~~~~~~~~~~~~~~
JW-68-85818-R            ~~~~~~~~~~~~~~~~~~~~~~~~~~~~~~~~~~~~~~~~~~~~~~~~~~~~~~~~~~~~~~~~~~~~~~~~~~~~~~~~~~~~~~~~~~~~~~~~~~~
JW-68-crl1556-R          ~~~~~~~~~~~~~~~~~~~~~~~~~~~~~~~~~~~~~~~~~~~~~~~~~~~~~~~~~~~~~~~~~~~~~~~~~~~~~~~~~~~~~~~~~~~~~~~~~~~
JW-68-crl1556-SIntR-rc   ~~~~~~~~~~~~~~~~~~~~~~~~~~~~~~~~~~~~~~~~~~~~~~~~~~~~~~~~~~~~~~~~~~~~~~~~~~~~~~~~~~~~~~~~~~~~~~~~~~~
JW-68-41436-R            ~~~~~~~~~~~~~~~~~~~~~~~~~~~~~~~~~~~~~~~~~~~~~~~~~~~~~~~~~~~~~~~~~~~~~~~~~~~~~~~~~~~~~~~~~~~~~~~~~~~
JW-68-41436-IntF         ~~~~~~~~~~~~~~~~~~~~~~~~~~~~~~~~~~~~~~~~~~~~~~~~~~~~~~~~~~~~~~~~~~~~~~~~~~~~~~~~~~~~~~~~~~~~~~~~~~~
JW-68-41436-SIntR-rc     ~~~~~~~~~~~~~~~~~~~~~~~~~~~~~~~~~~~~~~~~~~~~~~~~~~~~~~~~~~~~~~~~~~~~~~~~~~~~~~~~~~~~~~~~~~~~~~~~~~~

                                  310       320       330       340       350       360       370       380       390       400
                         ....|....|....|....|....|....|....|....|....|....|....|....|....|....|....|....|....|....|....|
Aotus_68_[Anan_2.0]      GGTTTCTCTTTGAATTTGCCTATATCCACTAGCCTTCTTTTGAAATTTGTCAGGGTCCTAGGAAATGCTCCTCTTCTCTGTGCTTCAAGGCTTGTTTAAA
JS-68-85818-SIntR-rc     ~~~~~~~~~~~~~~~~~~~~~~~~~~~~~~~~~~~~~~~~~~~~~~~~~~~~~~~~~~~~~~~~~~~~~~~~~~~~~~~~~~~~~~~~~~~~~~~~~~~
JW-68-85818-R            ~~~~~~~~~~~~~~~~~~~~~~~~~~~~~~~~~~CTTCTTTTGANTTTGTCAGGGTCCTAGGAAATGCTCCTCTTCT
JW-68-crl1556-R          ~~~~~~~~~~~~~~~~~~~~~~~~~~~~~CTAGCCTTCTTTTGAATTTGTCAGGGTCCTAGGAAATGCTCCTCTTCTCTGTGCTTCAAGGCTTGTTTAAA
JW-68-crl1556-SIntR-rc   ~~~~~~~~~~~~~~~~~~~~~~~~~~~~~~~~~~~~~~~~~~~~~~~~~~~~~~~~~~~~~~~~~~~~~~~~~~~~~~~~~~~~~~~~~~~~~~~~~~~
JW-68-41436-R            ~~~~~~~~~~~~~~~~~~~~~~~~~~~~~~~~~CCTTCTTTTGAATTTGTCAGGGTCCTAGGAAATGCTCCTCTTCTCTG
JW-68-41436-IntF         ~~~~~~~~~~~~~~~~~~~~~~~~~~~~~~~~~~~~~~~~~~~~~~~~~~~~~~~~~~~~~~~~~~~~~~~~~~~~~~~~~~~~~~~~~~~~~~~~~~~
JW-68-41436-SIntR-rc     ~~~~~~~~~~~~~~~~~~~~~~~~~~~~~~~~~~~~~~~~~~~~~~~~~~~~~~~~~~~~~~~~~~~~~~~~~~~~~~~~~~~~~~~~~~~~~~~~~~~

                                  410       420       430       440       450       460       470       480       490       500
                         ....|....|....|....|....|....|....|....|....|....|....|....|....|....|....|....|....|....|....|
Aotus_68_[Anan_2.0]      AGAGGTACAATTGAAGTTATTTACTTAACTCCTCATCATCTTGAAGGACTAGAGCAAAACCCcatgtgtttataaaaataaccatGA~~~~~~~~~~~~~
JS-68-85818-SIntR-rc     ~~~~~~~~~~~~~~~~~~~~~~~~~~AACTCCTCATCATCTTGAAGGACTAGAGCAAAACCCCATGTGTTTATAAAAATAACCATGA~~~~~~~~~~~~~
JW-68-85818-R
JW-68-crl1556-R          AGAGGTACAATTGAAGTTATTTACTTAACTCCTCATCATCTTGAAGGACTAGAGCAAAACCCCATGTGTTTATAAAAATAACCATGAGGGCCGGGCGCGG
JW-68-crl1556-SIntR-rc   ~~~~~~~~~~~~~~~~~~~~~~~~~~~~~~~~~~~~~~~~~~~~~~~~~~~~~~~~~~~~~~~~~~~~~~~~~~~~~~~~~~~~~~~~~~~~~~~~~~~
JW-68-41436-R
JW-68-41436-IntF         ~~~~~~~~~~~~~~~~~~~~~~~~~~~~~~~~~~~~~~~~~~~~~~~~~~~~~~~~~~~~~~~~~~~~~~~~~~~~~~~~~~~~~~~~~~~~~~~~~~~
JW-68-41436-SIntR-rc     ~~~~~~~~~~~~~~~~~~~~~~~~~~~~~~~~~~~~~~~~~~~~~~~~~~~~~~~~~~~~~~~~~~~~~~~~~~~~~~~~~~~~~~~~~~~~~~~~~~~
```

```
                                      510       520       530       540       550       560       570       580       590       600
                              ....|....|....|....|....|....|....|....|....|....|....|....|....|....|....|....|....|....|....|....|
Aotus_68_[Anan_2.0]           ~~~~~~~~~~~~~~~~~~~~~~~~~~~~~~~~~~~~~~~~~~~~~~~~~~~~~~~~~~~~~~~~~~~~~~~~~~~~~~~~~~~~~~~~~~~~~~~~~~~~
JS-68-85818-SIntR-rc          ~~~~~~~~~~~~~~~~~~~~~~~~~~~~~~~~~~~~~~~~~~~~~~~~~~~~~~~~~~~~~~~~~~~~~~~~~~~~~~~~~~~~~~~~~~~~~~~~~~~~
JW-68-85818-R
JW-68-crl1556-R               TGGCTCAAGCCTGTAATCCCAGCACTTTGGGAGGCCGAGGCGGGTGGATCACGAGGTCAAGAGATCAAGACCATCCTGGTCAACATGGTGAAACCCCGTC
JW-68-crl1556-SIntR-rc        ~~~~~~~~~~~~~~~~~~~~~~~~~~~~~~~~~~~~~~~~~~~~~~~~~~~~~~~~~~~~~~~~~~~~~~~~~~~~~~~~~~~~~~~~~~~~~~~~~~~~
JW-68-41436-R
JW-68-41436-IntF              ~~~~~~~~~~~~~~~~~~~~~~~~~~~~~~~~~~~~~~~~~~~~~~~~~~~~~~~~~~~~~~~~~~~~~~~~~~~~~~~~~~~~~~~~~~~~~~~~~~~~
JW-68-41436-SIntR-rc          ~~~~~~~~~~~~~~~~~~~~~~~~~~~~~~~~~~~~~~~~~~~~~~~~~~~~~~~~~~~~~~~~~~~~~~~~~~~~~~~~~~~~~~~~~~~~~~~~~~~~

                                      610       620       630       640       650       660       670       680       690       700
                              ....|....|....|....|....|....|....|....|....|....|....|....|....|....|....|....|....|....|....|....|
Aotus_68_[Anan_2.0]           ~~~~~~~~~~~~~~~~~~~~~~~~~~~~~~~~~~~~~~~~~~~~~~~~~~~~~~~~~~~~~~~~~~~~~~~~~~~~~~~~~~~~~~~~~~~~~~~~~~~~
JS-68-85818-SIntR-rc          ~~~~~~~~~~~~~~~~~~~~~~~~~~~~~~~~~~~~~~~~~~~~~~~~~~~~~~~~~~~~~~~~~~~~~~~~~~~~~~~~~~~~~~~~~~~~~~~~~~~~
JW-68-85818-R
JW-68-crl1556-R               TCTACTAACACAAAAAATTAGCTGGGCACGGTGGCGCGTGCCTGTAATCCCAGCTACTCAGGAGGCTGAGGCAGGAGAATTGCCTGAACCCAGGAGGCGG
JW-68-crl1556-SIntR-rc        ~~~~~~AACACAAAAAATTAGCTGGGCACGGTGGCGCGTGCCTGTAATCCCAGCTACTCAGGAGGCTGAGGCA
JW-68-41436-R
JW-68-41436-IntF              ~~~~~~~~~~~~~~~~~~~~~~~~~~~~~~~~~~~~~~~~~~~~~~~~~~~~~~~~~~~~~GAGGTCAAGAGATCGAGACCATCCTGGTCAACATGGTGA
JW-68-41436-SIntR-rc          ~~~~~~~~~~~~~~~~~~~~GCTGGGCACGGTGGCGCGTGCCTGTAATCCCAGCTACTCAGGAGG

                                      710       720       730       740       750       760       770       780       790       800
                              ....|....|....|....|....|....|....|....|....|....|....|....|....|....|....|....|....|....|....|....|
Aotus_68_[Anan_2.0}           ~~~~~~~~~~~~~~~~~~~~~~~~~~~~~~~~~~~~~~~~~~~~~~~~~~~~~~~~~~~~~~~~~~~~~~~~~~~~~TAGCATAAGCTATTTGAGGaatg
JS-68-85818-SIntR-rc          ~~~~~~~~~~~~~~~~~~~~~~~~~~~~~~~~~~~~~~~~~~~~~~~~~~~~~~~~~~~~~~~~~~~~~~~~~~~~~TAGCATAAGCTGTTTGAGGAATG
JW-68-85818-R
JW-68-crl1556-R               AGGTTGCGGTGAGCCGAGATCGCGCCATTGCACTCCAGCCTGGGTAACAAGAGCGAAACTCCGTCTCAAAAAAAAAA
JW-68-crl1556-SIntR-rc
JW-68-41436-R
JW-68-41436-IntF              AAC
JW-68-41436-SIntR-rc

                                      810       820       830       840       850       860       870       880       890       900
                              ....|....|....|....|....|....|....|....|....|....|....|....|....|....|....|....|....|....|....|....|
Aotus_68_[Anan_2.0]           agaaataaagtaaaacaatcagatcttagCAAAGGGTAGGCTGGGATGAAGCCATGACAAAGCAAGTTGATTAGGGAATAAGAAggttgtggccgggcgc
JS-68-85818-SIntR-rc          AGAAATAAAGTAAAACAATCAGATCTTAGCAAAGGGTAGGCTGGGATGAAGCCATCACAAAGCAAGTTGATTAGGGAATAAGAAGGTTGTGGCCGGGCGC
JW-68-85818-R
JW-68-crl1556-R
JW-68-crl1556-SIntR-rc
JW-68-41436-R
JW-68-41436-IntF
JW-68-41436-SIntR-rc
```

```
                                   910       920       930       940       950       960       970       980       990       1000
                          ....|....|....|....|....|....|....|....|....|....|....|....|....|....|....|....|....|....|....|....|
Aotus_68_[Anan_2.0]       ggtggctcacgcctgtaatcccagcactttgggaggcagaggcgggcggatcacgaggtcaagagatcgagaccatcctggtcaacatggtgaaaccccg
JS-68-85818-SIntR-rc      GGTGGCTCACGCCTGTAATCCCAGCACTTTGGGAGGCAGAGGCGGGTGGATCACGAGGTCAAGAGATCGAGACCATCCTNNTCAACATGGTGAAACCCCG
JW-68-85818-R
JW-68-crl1556-R
JW-68-crl1556-SIntR-rc
JW-68-41436-R
JW-68-41436-IntF
JW-68-41436-SIntR-rc

                                  1010      1020      1030      1040      1050      1060      1070      1080      1090      1100
                          ....|....|....|....|....|....|....|....|....|....|....|....|....|....|....|....|....|....|....|....|
Aotus_68_[Anan_2.0]       tctctactaaaaatacaaaaaaattagctgggcatggtggcgcgtgcctgtaatcccagctactcaggaggctgaggcaggagaattgcctgaacccagga
JS-68-85818-SIntR-rc      TCTCTACTAAAAATACAAAAAAATTNGCTGGGCATGGTGGCGCGTGCCTGTAATCCCAGCTACTCAGGAGGCTGAGG
JW-68-85818-R
JW-68-crl1556-R
JW-68-crl1556-SIntR-rc
JW-68-41436-R
JW-68-41436-IntF
JW-68-41436-SIntR-rc

                                  1110      1120      1130      1140      1150      1160      1170      1180      1190      1200
                          ....|....|....|....|....|....|....|....|....|....|....|....|....|....|....|....|....|....|....|....|
Aotus_68_[Anan_2.0]       ggcggaggttgcggtgagccgagatcgcgccattgcagcctgggtaacaagagcgaaactccgtctccaaaaaaaaaaaaaaaaaaaaaggttatcaCCG
JS-68-85818-SIntR-rc
JW-68-85818-R
JW-68-crl1556-R
JW-68-crl1556-SIntR-rc
JW-68-41436-R
JW-68-41436-IntF
JW-68-41436-SIntR-rc

                                  1210      1220      1230      1240      1250      1260      1270      1280      1290      1300
                          ....|....|....|....|....|....|....|....|....|....|....|....|....|....|....|....|....|....|....|....|
Aotus_68_[Anan_2.0]       AAAAATCGCATTTTGTAGTTTACAGAGGACACTGAAAGCTCTTTGTGTTTCAgcattttcaaaaagattttctACAGTATTTGGTCAAATTACCATGCTT
JS-68-85818-SIntR-rc
JW-68-85818-R
JW-68-crl1556-R
JW-68-crl1556-SIntR-rc
JW-68-41436-R
JW-68-41436-IntF
JW-68-41436-SIntR-rc
```

Figure S24b. Sequence alignment for *Alu* locus *Aotus*_68_018491627.1:7167631-7169129 (JW-68). PCR-based genotypes indicate the target *Alu* insertion is polymorphic among *A. nancymaae* individuals while homozygous present in other *Aotus* samples. *A. trivirgatus* appears to harbor a 2nd *Alu* insertion that is not present in other *Aotus* species (See Figure S24a). DNA sequencing was performed to confirm this prediction. The reference genome [Anan_2.0] is shown at the top followed by samples *A. azarae* 85818 and

A. trivirgatus crl-1556 and MSB-41436. Oligonucleotide primers for PCR are highlighted in bright green at positions 282 and 1225. *A. azarae* 85818 shares the target *Alu* which starts at position 891 (grey highlight) and is flanked by TSDs in yellow highlight. The PCR primer at position 1225-1246 is the reverse compliment of the forward primer shown in Supplemental File 1. It is located very close to the 3’ end of the target insertion and therefore did not generate sequence traces. *A. trivirgatus* crl-1556 has a 2nd *Alu* insertion that starts at position 488 (teal highlight) and extends to the A-tail at position 777 (*Alu* in aqua highlight) that is not present in [Anan_2.0] or *A. azarae* 85818. We have designed an alternative forward PCR primer, (*Aotus*_68_A.triv-F:ACTTGCTTTGTCATGGCTTCATC) shown in purple highlight at position 845 that could be used in conjunction with the existing reverse primer listed and shown in this alignment at position 282 (GGGATTGGGTGGAGAATTTGG) to potentially genotype this newly discovered *A. trivirgatus* derived *Alu* element separately from the [Anan_2.0] ascertained target insertion. We did not attempt this alternative PCR as the locus is not parsimony informative among our sample set.

```
                                 510       520       530       540       550       560       570       580       590       600
                        ....|....|....|....|....|....|....|....|....|....|....|....|....|....|....|....|....|....|....|....|
Owl_2LS_78_[Anan_2.0]   TCTGTAGATGGGTGGTAGAGGAACCTGTTTTACCCACTTTACAGGTTTGTTTCAATCGcagataaaagactatacatgcaagtgtgttataaaatataag
LR-78-crl1556-F         ~~~~~~~~~~~~~~~~~~~~~~~~~~~~~~~~~~~~~~~~~~~~~~~~~~~~~~~~~~~~~~~~~~~~ACTATACATGCAAGTGTGTTATAAAATAGAAG
LR-78-crl1556-SIntR-rc  ~~~~~~~~~~~~~~~~~~~~~~~~~~~TTTTACCCACTTTACAGGTTTGTTTCAATCACAGATAAAAGACTATACATGCAAGTGTGTTATAAAATAGAAG
LR-78-crl1556-IntF      ~~~~~~~~~~~~~~~~~~~~~~~~~~~~~~~~~~~~~~~~~~~~~~~~~~~~~~~~~~~~~~~~~~~~~~~~~~~~~~~~~~~~~~~~~~~~~~~~~~~~
LR-78-crl1556-R-rc      ~~~~~~~~~~~~~~~~~~~~~~~~~~~~~~~~~~~~~~~~~~~~~~~~~~~~~~~~~~~~~~~~~~~~~~~~~~~~~~~~~~~~~~~~~~~~~~~~~~~~
LR-78-85818-F           ~~~~~~~~~~~~~~~~~~~~~~~~~~~~~~~~~~~~~~~~~~~~~~~~~~~~~~~~~~~~~~~~~~~~~~TATACATGCAAGTGTGTTATAAAATATAAG
LS-78-85818-SIntR-rc    ~~~~~~~~~~~~~~~~~~~~~~~~~~~~~TTACCCACTTTACAGGTTTGTTTCAATCGCAGATAAAAGACTATACATGCAAGTGTGTTATAAAATATAAG
LR-78-85818-IntF        ~~~~~~~~~~~~~~~~~~~~~~~~~~~~~~~~~~~~~~~~~~~~~~~~~~~~~~~~~~~~~~~~~~~~~~~~~~~~~~~~~~~~~~~~~~~~~~~~~~~~
LS-78-85818-R-rc        ~~~~~~~~~~~~~~~~~~~~~~~~~~~~~~~~~~~~~~~~~~~~~~~~~~~~~~~~~~~~~~~~~~~~~~~~~~~~~~~~~~~~~~~~~~~~~~~~~~~~
LR_78-KB4883-R-rc       ~~~~~~~~~~~~~~~~~~~~~~~~~~~~~~~~~~~~~~~~~~~~~~~~~~~~~~~~~~~~~~~~~~~~~~~~~~~~~~~~~~~~~~~~~~~~~~~~~~~~
LR-78-86230-E-F         ~~~~~~~~~~~~~~~~~~~~~~~~~~~~~~~~~~~~~~~~~~~~~~~~~~~~~~ATCGCAGATAAAAGACTATACATGCAAGTGTGTTATAAAATATAAG
LR-78-86230-E-R-rc      ~~~~~~~~TGGGTGGTAGAGGAACCTGTTTTACCCACTTTACAGGTTTGTTTCGATCGCAGATAAAAGACTATACATGCAAGTGTGTTATAAAATATAAG

                                 610       620       630       640       650       660       670       680       690       700
                        ....|....|....|....|....|....|....|....|....|....|....|....|....|....|....|....|....|....|....|....|
Owl_2LS_78_[Anan_2.0}   atactcggccgggcgcggtggctcaagcctgtaatcccagcactttgggaggccgaggcgggtggatcacgaggtcaagagatcgagaccatcctggtca
LR-78-crl1556-F         ATACTCGGCCGGGCGCGGTGGCTCAAGCCTGTAATCCTAGCACTTTGGGAGGCCGAGGCGGGTGGATCATGAGGTCAGGAGATCGAGACCATTCTGGTCA
LR-78-crl1556-SIntR-rc  ATACTCGGCCGGGCGCGGTGGCTCAAGCCTGTAATCCTAGCACTTTGGGAGGCCGAGGCGGGTGGATCATGAGGTCAGGAGATCGAGACCATTCTGGTCA
LR-78-crl1556-IntF      ~~~~~~~~~~~~~~~~~~~~~~~~~~~~~~~~~~~~~~~~~~~~~~~~~~~~~~~~~~~~~~~~~~~~~~~~~~~~~~~~~~~~GAGACCATTCTGGTCA
LR-78-crl1556-R-rc      ~~~~~~~~~~~~~~~~~~~~~~~~~~~~~~~~~~~~~~~~~~~~~~~~~~~~~~~~~~~~~~~~~~~~~~~~~~~~~~~~~~~~~~~~~~~~~~~~~~~~
LR-78-85818-F           ATACTCNN~~GGGCGCGGTGGCTCAAGCCTGTAATCCCAGCACTTTGGGANGCCGAGGCGGGTGGATCACGAGGTCAAGAGATCGAGACCATCCTGGTCA
LS-78-85818-SIntR-rc    ATACTCGGCCGGGCGCGGTGGCTCAAGCCTGTAATCCCAGCACTTTGGGAGGCCGAGGCGGGTGGATCACGAGGTCAAGAGATCGAGACCATCCTGGTCA
LR-78-85818-IntF        ~~~~~~~~~~~~~~~~~~~~~~~~~~~~~~~~~~~~~~~~~~~~~~~~~~~~~~~~~~~~~~~~~~~~~~GAGGTCAAGAGATCGAGACCATCCTGGTCA
LS-78-85818-R-rc        ~~~~~~~~~~~~~~~~~~~~~~~~~~~~~~~~~~~~~~~~~~~~~~~~~~~~~~~~~~~~~~~~~~~~~~~~~~~~~~~~~~~~~~~~~~~~~~~~~~~~
LR_78-KB4883-R-rc       ~~~~~~~~~~~~~~~~~~~~~~~~~~~~~~~~~~~~~~~~~~~~~~~~~~~~~~~~~~~~~~~~~~~~~~~~~~~~~~~~~~~~~~~~~~~~~~~~~~~~
LR-78-86230-E-F         ATACT~~~~~~~~~~~~~~~~~~~~~~~~~~~~~~~~~~~~~~~~~~~~~~~~~~~~~~~~~~~~~~~~~~~~~~~~~~~~~~~~~~~~~~~~~~~~~~~
LR-78-86230-E-R-rc      ATACT~~~~~~~~~~~~~~~~~~~~~~~~~~~~~~~~~~~~~~~~~~~~~~~~~~~~~~~~~~~~~~~~~~~~~~~~~~~~~~~~~~~~~~~~~~~~~~~
```

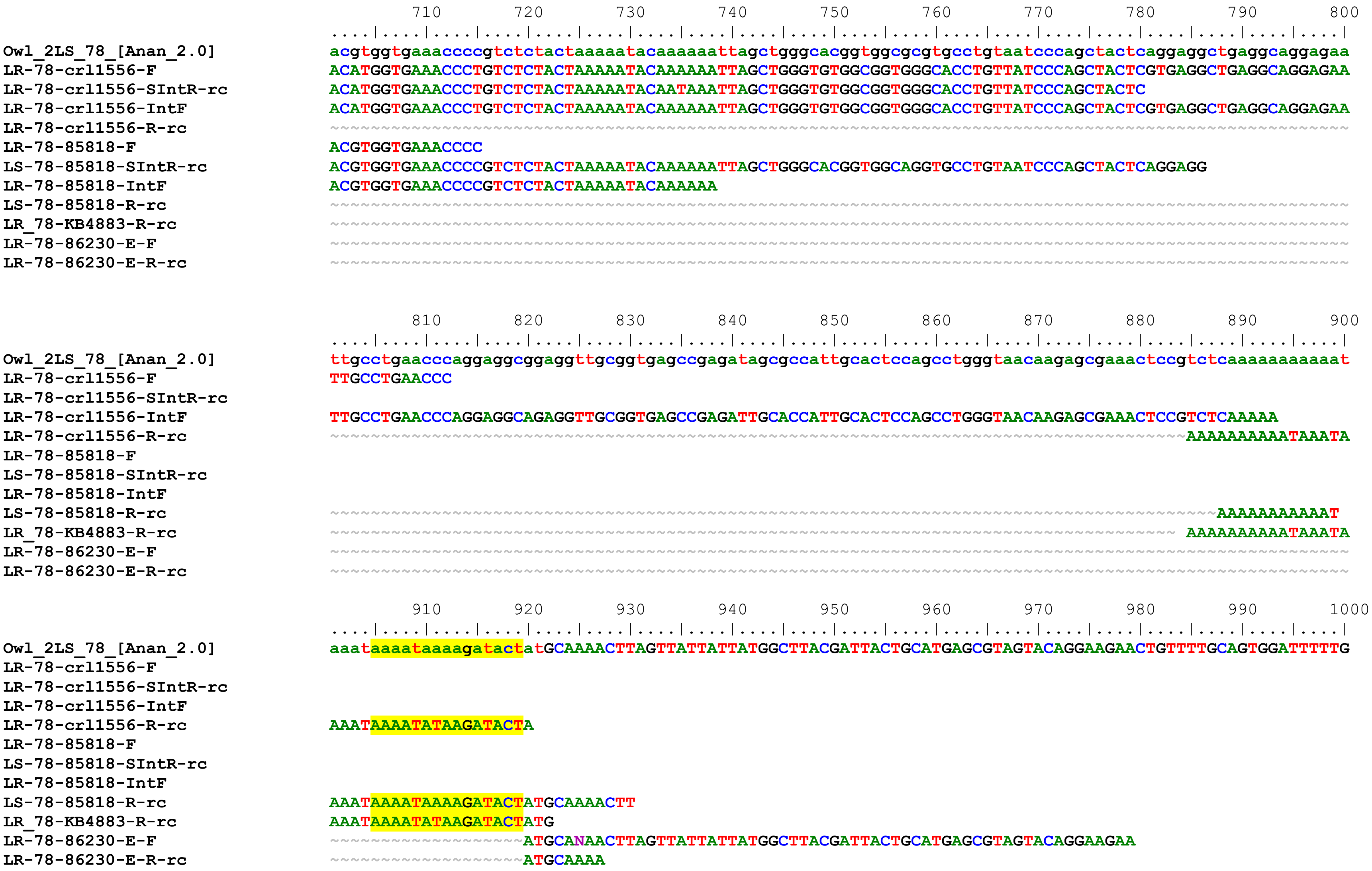

Figure S25. Sequence alignment for *Alu* locus Owl_2LS_078_018494005.1:2492157-2493663 (LR-78). PCR-based genotypes indicate that *A. vociferans* is homozygous absent (0/0) for the target *Alu* insertion, while other *Aotus* species are homozygous present (1/1), suggesting that *A. vociferans* is basal among the *Aotus* species on our panel. DNA sequencing was performed to confirm this

pattern. The reference genome [Anan_2.0] is shown at the top followed by samples *A. trivirgatus* crl-1556, *A. azarae* 85818 and *A. l. griseimembra* KB4883 (signal very weak, but has TSD, in yellow highlight). Sequencing confirms that *A. trivirgatus* crl-1556 and *A. azarae* 85818 share target *Alu* insertion starting at position 607 (grey highlight), while *A. vociferans* 86230 empty site amplicon displays a precise pre-integration site with both forward and reverse sequences spanning the target region.

```
                                  510       520       530       540       550       560       570       580       590       600
                          ....|....|....|....|....|....|....|....|....|....|....|....|....|....|....|....|....|....|....|....|
Aotus_1518_[Anan_2.0]     TGGCCATGGGTGCCAGTGACAAAGTAACAGCTCTGTCAAGCCCCAAACTAAGTACCTAGTTAGTCACTGTTGTGCAGCCGGTTAGAAAGAGGGGctcttg
JW-1518-crl1556-F         ~~~~~~~~~~~~~~~~~~~~~~~~~~~~~~~~~~~~~~~~~~~~~~~CTAAGTACCTAGTTAGTCACTGTTGTGCAGCCGGTTAGAAAGAGGGGCTCTTG
JW-1518-crl1556-SIntR-rc  ~~~~~~~~~~~~~~~~~~~~~~~~~~~~~~~~~~~~~~~~~~~~~~~~~~~~~~~~~~~~~~~~~~~~~~~~~~~~~~~~~~~~~~~~~~~~~~~~~~~~
JW-1518-crl1556-R-rc      ~~~~~~~~~~~~~~~~~~~~~~~~~~~~~~~~~~~~~~~~~~~~~~~~~~~~~~~~~~~~~~~~~~~~~~~~~~~~~~~~~~~~~~~~~~~~~~~~~~~~
JW-1518-85818-SIntR-rc    TGGCCATGGGTGCCAGTGACAAAGTAACAGCTTTGTCAAGCCCCAAACTAAGTACCTAGTTAGTCACTGTTGTGCAGCTGGTTAGAAAGAGGGGCTTTTG
JW-1518-85818-F           ~~~~~~~~~~~~~~~~~~~~~~~~~~~~~~~~~~~~~~~~~~~~~~~CTAAGTACCTAGTTAGTCACTGTTGTGCAGCTGGTTAGAAAGAGGGGCTCTTG
JW-1518-85818-IntF        ~~~~~~~~~~~~~~~~~~~~~~~~~~~~~~~~~~~~~~~~~~~~~~~~~~~~~~~~~~~~~~~~~~~~~~~~~~~~~~~~~~~~~~~~~~~~~~~~~~~~
JW-1518-85818-R-rc        ~~~~~~~~~~~~~~~~~~~~~~~~~~~~~~~~~~~~~~~~~~~~~~~~~~~~~~~~~~~~~~~~~~~~~~~~~~~~~~~~~~~~~~~~~~~~~~~~~~~~
JW-1518-86230-E-F         ~~~~~~~~~~~~~~~~~~~~~~~~~~~~~~~~CTGTC~AGCCCCAAACTAAGTACCTAGTTAGTCACTGTTGTGCAGCCGGTTAGAAAGAGGGGCTCTT~
JW-1518-86230-E-R-rc      TGGCCATGGGTGCCAGTGACAAAGTAACAGCTCTGTCAAGCCCCAAACTAAGTACCTAGTTAGTCACTGTTGTGCAGCCGGTTAGAAAGAGGGGCTCTT~

                                  610       620       630       640       650       660       670       680       690       700
                          ....|....|....|....|....|....|....|....|....|....|....|....|....|....|....|....|....|....|....|....|
Aotus_1518 [Anan_2.0]     gccgggcacggtggctcaagcctgtaatcccagcactttgggaggccgaggcgggtggatcacgaggtcgagagatcgagaccatcctggttaacattgt
JW-1518-crl1556-F         GCCAGGCGCGGTGGCTCAAGCCTGTAATCCCAGCACTTTGGGAGGCCGAGGCGGGTGGATCACGAGGTCGAGAGATCGAGACCATCCTGGTCAACATTGT
JW-1518-crl1556-SIntR-rc  ~~~~~~~~~~~~~~~~~~~~~~~~~~~~~~~~~~~~~~~~~~~~~~~~~~~~~~~~~~~~~~~~~~~~~~~~~~~~~~~~~~~~~~~~~~~~~~~~~~~~
JW-1518-crl1556-R-rc      ~~~~~~~~~~~~~~~~~~~~~~~~~~~~~~~~~~~~~~~~~~~~~~~~~~~~~~~~~~~~~~~~~~~~~~~~~~~~~~~~~~~~~~~~~~~~~~~~~~~~
JW-1518-85818-SIntR-rc    GCCGGGGGCGGTGACTCAAGCCTGTAATCCCAGCACTTTGGGAGGCCGAGGCGGGTGGATCACGAGGTCGAGAGATCGAGACCATCCTGGTCAACATTGT
JW-1518-85818-F           GCCGGGCGCGGTGACTCAAGCCTGTAATCCCAGCACTTTGGGAGTCCGAGGCGGGTGGATCACGAGGTCGAGAGATCGAGACCATCCTGGTCAACATTGT
JW-1518-85818-IntF        ~~~~~~~~~~~~~~~~~~~~~~~~~~~~~~~~~~~~~~~~~~~~~~~~~~~~~~~~~~~~~~~GAGGTCGAGAGATCGAGACCATCCTGGTCAACATTGT
JW-1518-85818-R-rc        ~~~~~~~~~~~~~~~~~~~~~~~~~~~~~~~~~~~~~~~~~~~~~~~~~~~~~~~~~~~~~~~~~~~~~~~~~~~~~~~~~~~~~~~~~~~~~~~~~~~~
JW-1518-86230-E-F         ~~~~~~~~~~~~~~~~~~~~~~~~~~~~~~~~~~~~~~~~~~~~~~~~~~~~~~~~~~~~~~~~~~~~~~~~~~~~~~~~~~~~~~~~~~~~~~~~~~~~
JW-1518-86230-E-R-rc      ~~~~~~~~~~~~~~~~~~~~~~~~~~~~~~~~~~~~~~~~~~~~~~~~~~~~~~~~~~~~~~~~~~~~~~~~~~~~~~~~~~~~~~~~~~~~~~~~~~~~

                                  710       720       730       740       750       760       770       780       790       800
                          ....|....|....|....|....|....|....|....|....|....|....|....|....|....|....|....|....|....|....|....|
Aotus_1518 [Anan_2.0]     gaaaccccgtctctactaatacaaaaaaaaaaa~ttagctgggcgcggtggcgcatgcctgtaatcccagctactcaggaggctgaggcaggagaattgc
JW-1518-crl1556-F         GAAACCCCGTCTCTACTAATACAAAAAAAAAAAATTACT~GGGCACGGGGGCCCGTGCCTGTAATCCCACCTACTCAGGAGGCTGAGGCAGGAAAATTGC
JW-1518-crl1556-SIntR-rc  ~~~~~~~~~~~~~~~~~~~TACAAAAAAAAAAAATTAGCTGGGCACGGTGGCGCGTGCCTG
JW-1518-crl1556-R-rc      ~~~~~~~~~~~~~~~~~~~~~~~~~~~~~~~~~~~~~~~~~~~~~~~~~~~~~~~~~~~~~~~~~~~~~~~~~~~~~~~~~~~~~~~~~~~~~~~~~~~~
JW-1518-85818-SIntR-rc    GAAACCCGGTTTTTAATAATACAAAAAAAAAAA~TTAGCTGGGCACGGTGGCGCATGCCTGTAATCCCAGCTACTCAGGAGG
JW-1518-85818-F           GAAACCCGGTCTCTACTAATACAAAAAAAAAAA~TTANCTGGGCACGGTGGCGCATGCCTGTAATCCCAGCTACTCAGGAGGCTGAGGCAGGAAAATTGC
JW-1518-85818-IntF        GAAACCCGGTCTCTACTAATACAAAAAAAAAAA
JW-1518-85818-R-rc        ~~~~~~~~~~~~~~~~~~~~~~~~~~~~~~~~~~~~~~~~~~~~~~~~~~~~~~~~~~~~~~~~~~~~~~~~~~~~~~~~~~~~~~~~~~~~~~~~~~~~
JW-1518-86230-E-F         ~~~~~~~~~~~~~~~~~~~~~~~~~~~~~~~~~~~~~~~~~~~~~~~~~~~~~~~~~~~~~~~~~~~~~~~~~~~~~~~~~~~~~~~~~~~~~~~~~~~~
JW-1518-86230-E-R-rc      ~~~~~~~~~~~~~~~~~~~~~~~~~~~~~~~~~~~~~~~~~~~~~~~~~~~~~~~~~~~~~~~~~~~~~~~~~~~~~~~~~~~~~~~~~~~~~~~~~~~~
```

```
                                        810       820       830       840       850       860       870       880       890       900
                                ....|....|....|....|....|....|....|....|....|....|....|....|....|....|....|....|....|....|....|....|
Aotus_1518 [Anan_2.0]           ctgaacccaggaggcggaggttgcggtgagccgagatcgcgccattgcactccagcctgggtaacaagagcgagactccgtctcaaaaaaaaaaaaaaaa
JW-1518-crl1556-F               CTGAACCCAGGAGGCGGAGGTTGCGGTGAGCCNAAATCGCCCCTTTGCACTCTACCCTGGGTAACAAGANTGANACTCCGTCTCAAAAAAAAAAAAAAAA
JW-1518-crl1556-SIntR-rc
JW-1518-crl1556-R-rc            ~~~~~~~~~~~~~~~~~~~~~~~~~~~~~~~~~~~~~~~~~~~~~~~~~~~~~~~~~~~~~~~~~~~~~~~~~~~~~~~~~AAAAAAAAAAAAAAAAAAA
JW-1518-85818-SIntR-rc
JW-1518-85818-F                 CTGAACCCAGGAGGCGGAGGTTGCGGTGAGCCGAGATCGCCCCATTGCTCTCCAGCCTGGGTAACAAGAGCGAGACTCCGTCTCAAAAAAAAAAAAAAAA
JW-1518-85818-IntF
JW-1518-85818-R-rc              ~~~~~~~~~~~~~~~~~~~~~~~~~~~~~~~~~~~~~~~~~~~~~~~~~~~~~~~~~~~~~~~~~~~~~~~~~~~~~~~~~AAAAAAAAAAAAAAAAAAA
JW-1518-86230-E-F               ~~~~~~~~~~~~~~~~~~~~~~~~~~~~~~~~~~~~~~~~~~~~~~~~~~~~~~~~~~~~~~~~~~~~~~~~~~~~~~~~~~~~~~~~~~~~~~~~~~~~
JW-1518-86230-E-R-rc            ~~~~~~~~~~~~~~~~~~~~~~~~~~~~~~~~~~~~~~~~~~~~~~~~~~~~~~~~~~~~~~~~~~~~~~~~~~~~~~~~~~~~~~~~~~~~~~~~~~~~

                                        910       920       930       940       950       960       970       980       990      1000
                                ....|....|....|....|....|....|....|....|....|....|....|....|....|....|....|....|....|....|....|....|
0_Aotus_1518::NW_018491627.1:1  aaagaaagaaagaggggctCTTGAGGAGTGGAGACCAGAAGTCCTACGGAAGGAACGTCTTTGTGAACATCATGGTGGATGCTCCCCTTGGGTCCTTGTC
JW-1518-crl1556-F               AAAAAAAAAAAA
JW-1518-crl1556-SIntR-rc
JW-1518-crl1556-R-rc            AAAAAAAGAAAGAGGGGCTCTTGAGGAGTGGAGACCAGAAGTCCTACGGAAGGA
JW-1518-85818-SIntR-rc
JW-1518-85818-F                 AAAAAAAAAAAAAAAA
JW-1518-85818-IntF
JW-1518-85818-R-rc              AAAAAAAGAAAGAGGGGCTCTTGAGGAGTGGAGACCAGAAGTCCTACGGAAGG
JW-1518-86230-E-F               ~~~~~~~~~~~~~~~~~~~~~~GAGGAGTGGAGACCAGAAGTCCTACGGAAGGAACGTCTTTGTGAACATCATGGTGGATGCTCCCCTTGGGTACTTGTC
JW-1518-86230-E-R-rc            ~~~~~~~~~~~~~~~~~~~~~~GAGGAGTGGAGACCAGAAGTCCTACGGAAGGAAC
```

Figure S26. Sequence alignment for *Alu* locus *Aotus*_1518_018491627.1:11015550-11017060 (JW-1518). The gel image is shown in the main text as Figure 1d. PCR based genotypes indicate that all *A. trivirgatus*, *A. nancymaae* and *A. azarae* individuals are homozygous present (1/1) for the target *Alu* while all *A. vociferans* individuals are homozygous absent (0/0) for the insertion. DNA sequencing was performed to confirm this pattern. The reference genome [Anan_2.0] is shown at the top followed by samples *A. trivirgatus* crl-1556 and *A. azarae* 85818, both of whom share the target *Alu* insertion that starts at position 600 (gray highlight), TSDs are in yellow highlight. Sequence for the empty site amplicon fragment for *A. vociferans* 86230 flanks the target region as a precise pre-integration site, confirming the genotypes. This *Alu* presence/absence pattern suggests *A. vociferans* is basal among the *Aotus* species on our panel.

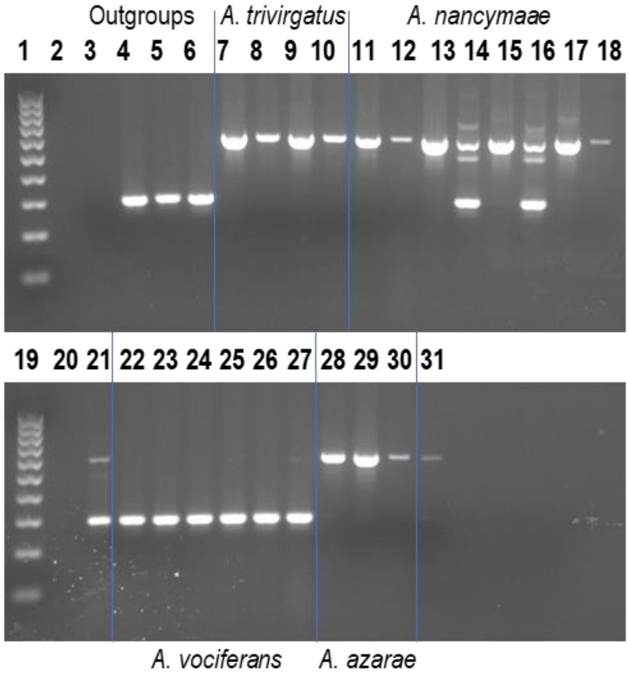

Figure S27a: *Alu* locus *Aotus*_1546_018502359.1:170192-171687 (JW-1546): Lanes: 1, 19- 100 bp DNA ladder, 2, 20-blank, 3- TLE (negative control), 4- Human (HeLa), 5- *Callithrix jacchus* (Common marmoset), 6- *Saimiri s. sciureus* (Common squirrel monkey), 7-10 *A. trivirgatus*, 11-18, 21 *A. nancymaae*, 22-27 *A. vociferans*, 28-30 *A. azarae*, 31-*A. lemurinus griseimembra*. *Alu* is homozygous present in *A. trivirgatus*, *A. azarae* and *A. lemurinus griseimembra* (~595 bp fragment) while homozygous absent (~298 bp fragment) in *A. vociferans* and showing heterozygosity in three of nine *A. nancymaae* individuals. DNA sequencing is required to confirm this insertion pattern (see alignment Figure S27b) Sequencing confirms that both *A. trivirgatus* and *A. azarae* share the target *Alu* insertion while *A. nancymaae* 86334 and *A. vociferans* 86230 empty site amplicons (~298 bp) represent precise pre-integration sites. This *Alu* pattern suggests *A. vociferans* is basal while *A. nancymaae* is more closely related to grey-neck species than *A. azarae* is.

```
                                   510       520       530       540       550       560       570       580       590       600
                          ....|....|....|....|....|....|....|....|....|....|....|....|....|....|....|....|....|....|....|....|
Aotus-1546_[Anan_2.0]     GACCTGGCCCTTGCAAATATTTGACTTTGCAACACTTGCCCTGAAGTTATAGGTTCTGAATGGTAGAGAAGCAGGGCTAAAGGCAGAATAAGAAAtggct
JW-1546-crl1556-SIntR-rc  GACCTGGCCCTTGCAAATATTTGACTTTGCAACACTTGCCCTGAAGTTATAGGTTCTGAATGGTAGAGAAGCAGGGCTAAATGCAGAATAAGAAATGGCT
JW-1546-crl1556-F         GACCTGGCCCTTGCAAATATTTGACTTTGCAACACTTGCCCTGAAGTTATAGGTTCTGAATGGTAGAGAAGCAGGGCTAAATGCAAAATAAGAAATGGCT
JW-1546-crl1556-IntF      ~~~~~~~~~~~~~~~~~~~~~~~~~~~~~~~~~~~~~~~~~~~~~~~~~~~~~~~~~~~~~~~~~~~~~~~~~~~~~~~~~~~~~~~~~~~~~~~~~~~~
JW-1546-crl1556-R-rc      ~~~~~~~~~~~~~~~~~~~~~~~~~~~~~~~~~~~~~~~~~~~~~~~~~~~~~~~~~~~~~~~~~~~~~~~~~~~~~~~~~~~~~~~~~~~~~~~~~~~~
JW-1546-85818-SIntR-rc    GACCTGGCCCTTGCAAATATTTGACTTTGCAACACTTGCCCTGAAGTTATAGGTTCTGAATGGTAGAGAAGCAGGGCTAAATGCAGAATAAGAAATGGCT
JW-1546-85818-F           GACCTGGCCCTTGCAAATATTTGACTTTGCAACACTTGCCCTGAAGTTATAGGTTCTGAATGGTAGAGAAGCAGGGCTAAATGCAAAATAAGAAATGGCT
JW-1546-85818-IntF        ~~~~~~~~~~~~~~~~~~~~~~~~~~~~~~~~~~~~~~~~~~~~~~~~~~~~~~~~~~~~~~~~~~~~~~~~~~~~~~~~~~~~~~~~~~~~~~~~~~~~
JW-1546-85818-R-rc        ~~~~~~~~~~~~~~~~~~~~~~~~~~~~~~~~~~~~~~~~~~~~~~~~~~~~~~~~~~~~~~~~~~~~~~~~~~~~~~~~~~~~~~~~~~~~~~~~~~~~
JW-1546-86334-E-F         GACCTGGCCCTTGCAAATATTTGACTTTGCAACACTTGCCCTAAAGTTATAGGTTCTGAATGGTAAAGAAGCAGGGCTAAATGCAGAATAAGAAATGGCT
JW-1546-86334-E-R-rc      GACCTGGCCCTTGCAAATATTTGACTTTGCAACACTTGCCCTAAAGTTATAGGTTCTGAATGGTAAAGAAGCAGGGCTAAATGCAGAATAAGAAATGGCT
JW-1546-86230-E-F         GACCTGGCCCTTGCAAATATTTGACTTTGCAACACTTGCCCTGAAGTTATAGGTTCTGAATGGTAGAGAAGCAGGGCTAAATGCAAAATAAGAAATGGCT
JW-1546-86230-E-R-rc      GACCTGGCCCTTGCAAATATTTGACTTTGCAACACTTGCCCTGAAGTTATAGGTTCTGAATGGTAGAGAAGCAGGGCTAAATGCAGAATAAGAAATGGCT

                                   610       620       630       640       650       660       670       680       690       700
                          ....|....|....|....|....|....|....|....|....|....|....|....|....|....|....|....|....|....|....|....|
Aotus-1546_[Anan_2.0]     ggccgggcacggtggctcaagcctgtaatcccagcactttgggaggccgaggcgggtgg~~~~atcacgaggtcaagagatcgagaccatcctggtcaac
JW-1546-crl1556-SIntR-rc  GGCCGGGCACGGTGGCTCAAGCCTGTAATCCCAGCACTTTGGGAGGCCGAGGCGGGTGG~~~~ATCACGAGGTCAAGAGATCGAGACCATCCTGGTCAAC
JW-1546-crl1556-F         GGCCGGGCACGGTGGCTCAAGCCTGTAATCCCAGCACTTTGGGAGGCCGAGGCGGGTGG~~~~ATCACGAGGTCAAGAGATCGAGACCATCCTGGTCAAC
JW-1546-crl1556-IntF      ~~~~~~~~~~~~~~~~~~~~~~~~~~~~~~~~~~~~~~~~~~~~~~~~~~~~~~~~~~~~~~~~~~~~GAGGTCAAGAGATCGAGACCATCCTGGTCAAC
JW-1546-crl1556-R-rc      ~~~~~~~~~~~~~~~~~~~~~~~~~~~~~~~~~~~~~~~~~~~~~~~~~~~~~~~~~~~~~~~~~~~~~~~~~~~~~~~~~~~~~~~~~~~~~~~~~~~~
JW-1546-85818-SIntR-rc    GGCCGGGCGCGGTGGCTCAAGCCTGTAATCCCAGCACTTTGGGAGGCCAAGGCGGGTGGGTGGATCACGAGGTCAAGAGATCGAGACCATCCTGGTCAAC
JW-1546-85818-F           GGCCGGGCGCGGTGGCTCAAGCCTGTAATCCCAGCACTTTGGGAGGCCAAGGCGGGTGGGTGGATCACGAGGTCAAGAGATCGAGACCATCCTGGTCAAC
JW-1546-85818-IntF        ~~~~~~~~~~~~~~~~~~~~~~~~~~~~~~~~~~~~~~~~~~~~~~~~~~~~~~~~~~~~~~~~~~~~GAGGTCAAGAGATCGAGACCATCCTGGTCAAC
JW-1546-85818-R-rc        ~~~~~~~~~~~~~~~~~~~~~~~~~~~~~~~~~~~~~~~~~~~~~~~~~~~~~~~~~~~~~~~~~~~~~~~~~~~~~~~~~~~~~~~~~~~~~~~~~~~~
JW-1546-86334-E-F         ~~~~~~~~~~~~~~~~~~~~~~~~~~~~~~~~~~~~~~~~~~~~~~~~~~~~~~~~~~~~~~~~~~~~~~~~~~~~~~~~~~~~~~~~~~~~~~~~~~~~
JW-1546-86334-E-R-rc      ~~~~~~~~~~~~~~~~~~~~~~~~~~~~~~~~~~~~~~~~~~~~~~~~~~~~~~~~~~~~~~~~~~~~~~~~~~~~~~~~~~~~~~~~~~~~~~~~~~~~
JW-1546-86230-E-F         ~~~~~~~~~~~~~~~~~~~~~~~~~~~~~~~~~~~~~~~~~~~~~~~~~~~~~~~~~~~~~~~~~~~~~~~~~~~~~~~~~~~~~~~~~~~~~~~~~~~~
JW-1546-86230-E-R-rc      ~~~~~~~~~~~~~~~~~~~~~~~~~~~~~~~~~~~~~~~~~~~~~~~~~~~~~~~~~~~~~~~~~~~~~~~~~~~~~~~~~~~~~~~~~~~~~~~~~~~~

                                   710       720       730       740       750       760       770       780       790       800
                          ....|....|....|....|....|....|....|....|....|....|....|....|....|....|....|....|....|....|....|....|
Aotus-1546_[Anan_2.0]     atggtgaaaccccgtctctactaatacaaaaaattagctgggcatggtggcgcgtgcctgtaatcccagctactcaggaggctgaggcaggagaattgcc
JW-1546-crl1556-SIntR-rc  ATGGTGAAACCCCGTCTCTACTAATACAAAAAATTAGCTGGGCATGGTGGCGCATGCCTGTAATCCCAGCTACTCAGGAGG
JW-1546-crl1556-F         ATGGTGAAACCCCGTCTCTACTAATACAAAAAATTAGCTGGGCATGGTGGCGCATGCCTGTAATCCCAGCTACTCAGGAGGCTGAGGCAGGAGAATTGCC
JW-1546-crl1556-IntF      ATGGTGAAACCCCGTCTCTACTAATACAAAAAATTAGCTGGGCATGGTGGCGCATGCCTGTAATCCCAGCTACTCAGGAGGCTGAGGCAGGAGAATTGCC
JW-1546-crl1556-R-rc      ~~~~~~~~~~~~~~~~~~~~~~~~~~~~~~~~~~~~~~~~~~~~~~~~~~~~~~~~~~~~~~~~~~~~~~~~~~~~~~~~~~~~~~~~~~~~~~~~~~~~
JW-1546-85818-SIntR-rc    ATGGTGAAACCCCGTCTCTACTAATACAAAAAATTAGCTGGGCATGGTGGCGCGTGCCTGTAATCCCAGCTACTCAGGAGG
JW-1546-85818-F           ATGGTGAA
JW-1546-85818-IntF        ATGGTGAAACCCCGTCTCTACTAATACAAAAAATTAGCTGGGCATGGTGGCGCGTGCCTGTAATCCCAGCTACTCAGGAGGCTGAGGCAGGAGAATTGCC
JW-1546-85818-R-rc        ~~~~~~~~~~~~~~~~~~~~~~~~~~~~~~~~~~~~~~~~~~~~~~~~~~~~~~~~~~~~~~~~~~~~~~~~~~~~~~~~~~~~~~~~~~~~~~~~~~~~
JW-1546-86334-E-F         ~~~~~~~~~~~~~~~~~~~~~~~~~~~~~~~~~~~~~~~~~~~~~~~~~~~~~~~~~~~~~~~~~~~~~~~~~~~~~~~~~~~~~~~~~~~~~~~~~~~~
JW-1546-86334-E-R-rc      ~~~~~~~~~~~~~~~~~~~~~~~~~~~~~~~~~~~~~~~~~~~~~~~~~~~~~~~~~~~~~~~~~~~~~~~~~~~~~~~~~~~~~~~~~~~~~~~~~~~~
JW-1546-86230-E-F         ~~~~~~~~~~~~~~~~~~~~~~~~~~~~~~~~~~~~~~~~~~~~~~~~~~~~~~~~~~~~~~~~~~~~~~~~~~~~~~~~~~~~~~~~~~~~~~~~~~~~
JW-1546-86230-E-R-rc      ~~~~~~~~~~~~~~~~~~~~~~~~~~~~~~~~~~~~~~~~~~~~~~~~~~~~~~~~~~~~~~~~~~~~~~~~~~~~~~~~~~~~~~~~~~~~~~~~~~~~
```

```
                                         810       820       830       840       850       860       870       880       890       900
                                ....|....|....|....|....|....|....|....|....|....|....|....|....|....|....|....|....|....|....|....|
Aotus-1546_[Anan_2.0]           tgaacccaggaggcggaggttgcggtgagccgagatcgcgccattgcactccagcctgggtaacgagcgaaactccatctcaaaaaaaaaaaaaaaagaaa
JW-1546-crl1556-SIntR-rc
JW-1546-crl1556-F               TGAACCCAGGAGGCGGAGGTTGCGGTGAGCCGAGATCGCGCCATTGCACTCCAGCCTGGGTAACGAGCGAAACTCCGTCTCAAAAAAAAAAAAAAAAAAA
JW-1546-crl1556-IntF            TGAACCCAGGAGGCGGAGGTTGCGGTGAGCCGAGATCGCGCCATTGCACTCCAGCCTGGGTAACGAGCGAAACTCCGTCTCAAAAAAAAAAAAAAAAAAA
JW-1546-crl1556-R-rc            ~~~~~~~~~~~~~~~~~~~~~~~~~~~~~~~~~~~~~~~~~~~~~~~~~~~~~~~~~~~~~~~~~~~~~~~~~~~~~~~~~AAAAAAAAAAAAAAAGAAA
JW-1546-85818-SIntR-rc
JW-1546-85818-F
JW-1546-85818-IntF              TGAACCCAGGAGGCGGAGGTTGCAGTGAGCCGAGATCGCGCCATTGCACTCCAGCCTGGGTAAGGAGCGA
JW-1546-85818-R-rc              ~~~~~~~~~~~~~~~~~~~~~~~~~~~~~~~~~~~~~~~~~~~~~~~~~~~~~~~~~~~~~~~~~~~~~~~~~~~~~~~~~AAAAAAAAGAAAAAAGAAA
JW-1546-86334-E-F               ~~~~~~~~~~~~~~~~~~~~~~~~~~~~~~~~~~~~~~~~~~~~~~~~~~~~~~~~~~~~~~~~~~~~~~~~~~~~~~~~~~~~~~~~~~~~~~~~~~~~
JW-1546-86334-E-R-rc            ~~~~~~~~~~~~~~~~~~~~~~~~~~~~~~~~~~~~~~~~~~~~~~~~~~~~~~~~~~~~~~~~~~~~~~~~~~~~~~~~~~~~~~~~~~~~~~~~~~~~
JW-1546-86230-E-F               ~~~~~~~~~~~~~~~~~~~~~~~~~~~~~~~~~~~~~~~~~~~~~~~~~~~~~~~~~~~~~~~~~~~~~~~~~~~~~~~~~~~~~~~~~~~~~~~~~~~~
JW-1546-86230-E-R-rc            ~~~~~~~~~~~~~~~~~~~~~~~~~~~~~~~~~~~~~~~~~~~~~~~~~~~~~~~~~~~~~~~~~~~~~~~~~~~~~~~~~~~~~~~~~~~~~~~~~~~~

                                         910       920       930       940       950       960       970       980       990      1000
                                ....|....|....|....|....|....|....|....|....|....|....|....|....|....|....|....|....|....|....|....|
Aotus-1546_[Anan_2.0]           tggctacATCGGTCGCTAGTCCACCAGGTGTCCATGACCCAGAGGTACCCTGCATTCAGACAGCCACCAAGTCCTGCTGATTCT---TGTACAGTGTCTA
JW-1546-crl1556-SIntR-rc
JW-1546-crl1556-F               AAAAA
JW-1546-crl1556-IntF            AAAAAA
JW-1546-crl1556-R-rc            TGGCTACATCGGTCGC
JW-1546-85818-SIntR-rc
JW-1546-85818-F
JW-1546-85818-IntF
JW-1546-85818-R-rc              TGN~TACATCGGTCGCTA
JW-1546-86334-E-F               ~~~~~ACATCGGTCACTAGTCCACCAGGTGTCCATGACCCAGAGGTACCCTGCATTCAGACAGCCACCAAGTCA
JW-1546-86334-E-R-rc            ~~~~~ACATCGGTCACTA
JW-1546-86230-E-F               ~~~~~ACATTGGTCACTAGTCCACCAGGTGTCCATGACCCAGAGGTACCCTGCATTCAGACAGCCACCAAG
JW-1546-86230-E-R-rc            ~~~~~A
```

Figure S27b. Sequence alignment for *Alu* locus *Aotus*_1546_018502359.1:170192-171687. The reference genome [Anan_2.0] is shown at the top followed by samples *A. trivirgatus* crl-1556, *A. azarae* 85818, both of whom share the target *Alu* insertion that starts at position 601 (gray highlight), TSDs are in yellow highlight. Empty site amplicon fragments for *A. nancymaae* 86334 and *A. vociferans* 86230 flank the target region and are precise pre-integration sites. This *Alu* pattern suggests *A. vociferans* is basal while *A. nancymaae* (86334 heterozygous for the target insertion) is more closely related to grey-neck species than *A. azarae* is.

```
                                                 410       420       430       440       450       460       470       480       490       500
                                        ....|....|....|....|....|....|....|....|....|....|....|....|....|....|....|....|....|....|....|....|
Aotus_1288 [Anan_2.0]                   atatgaGGGGTAGTCAACTTCCACAAGTGAGGTGCAGAGAGTTTGCAAACAGATTGTAATCAAACAGATCATAATCAaacaaatgctattttttaacagc
JW-1288V2-crl1556-F                     ~~~~~~~~~~~~~~~~~~~~~~~~~~~~~~~~~~~~~~~~~~~~~~~~~~~~~~~~~~~~~~~~~~~ATCATAATCAAACAAATGCTATTTTTTAACAGC
JW-1288V2-crl1556-IntF                  ~~~~~~~~~~~~~~~~~~~~~~~~~~~~~~~~~~~~~~~~~~~~~~~~~~~~~~~~~~~~~~~~~~~~~~~~~~~~~~~~~~~~~~~~~~~~~~~~~~~~
JW-1288V2-crl1556-SIntR-rc              ~~~~~~~~~~~~~~~~~~~~~~~~~~~~~~~~~~~~~~~~~~~~~~~~~~~~~~~~~~~~~~~~~~~~~~~~~~~~~~~~~~~~~~~~~~~~~~~~~~~~
JW-1288V2-crl1556-R-rc                  ~~~~~~~~~~~~~~~~~~~~~~~~~~~~~~~~~~~~~~~~~~~~~~~~~~~~~~~~~~~~~~~~~~~~~~~~~~~~~~~~~~~~~~~~~~~~~~~~~~~~
JW-1288V2-86218-F                       ~~~~~~~~~~~~~~~~~~~~~~~~~~~~~~~~~~~~~~~~~~~~~~~~~~~~~~~~~~~~~~~~~~~~~~~~~~TCAAACAAATGCTATTTTTTAACAGC
JW-1288V2-86218-IntF                    ~~~~~~~~~~~~~~~~~~~~~~~~~~~~~~~~~~~~~~~~~~~~~~~~~~~~~~~~~~~~~~~~~~~~~~~~~~~~~~~~~~~~~~~~~~~~~~~~~~~~
JW-1288V2-86218-SIntR-rc                ~~~~~~~~~~~~~~~~~~~~~~~~~~~~~~~~~~~~~~~~~~~~~~~~~~~~~~~~~~~~~~~~~~~~~~~~~~~~~~~~~~~~~~~~~~~~~~~~~~~~
JW-1288V2-86218-R-rc                    ~~~~~~~~~~~~~~~~~~~~~~~~~~~~~~~~~~~~~~~~~~~~~~~~~~~~~~~~~~~~~~~~~~~~~~~~~~~~~~~~~~~~~~~~~~~~~~~~~~~~
JW-1288V2-85818-F                       ~~~~~~~~~~~~~~~~~~~~~~~~~~~~~~~~~~~~~~~~~~~~~~~~~~~~~~~~~~~~~~~~~~~~TCATAATCAAACAAATGCTATTTTTTAACAGC
JW-1288V2-85818-IntF                    ~~~~~~~~~~~~~~~~~~~~~~~~~~~~~~~~~~~~~~~~~~~~~~~~~~~~~~~~~~~~~~~~~~~~~~~~~~~~~~~~~~~~~~~~~~~~~~~~~~~~
JW-1288V2-85818-SIntR-rc                ~~~~~~~~~~~~~~~~~~~~~~~~~~~~~~~~~~~~~~~~~~~~~~~~~~~~~~~~~~~~~~~~~~~~~~~~~~~~~~~~~~~~~~~~~~~~~~~~~~~~
JW-1288V2-85818-R-rc                    ~~~~~~~~~~~~~~~~~~~~~~~~~~~~~~~~~~~~~~~~~~~~~~~~~~~~~~~~~~~~~~~~~~~~~~~~~~~~~~~~~~~~~~~~~~~~~~~~~~~~
JW-1288V2-86100-E-F                     ~~~~~~~~~~~~~~~~~~~~~~~~~~~~~~~~~~~~~~~~~~~~~~~~~~~~~~~~~~~~~~~~~~~~~~~~~~~~~~~~~~~TGCTATTTTTTAACAGC
JW-1288V2-86100-E-R-rc                  ~~~~~~~~~~~~~~~~~~~~~~~~~~GTGAGGTGCAGAGAGTTTGCAAACAGATTGCAATCAAACAGATCATAATCGAACAAATGCTATTTTTTAACAGC
JW-1288V2-86230-E-F                     ~~~~~~~~~~~~~~~~~~~~~~~~~~~~~~~~~~~~~~~~~~~~~~~~~~~~~~~~~~~~~~~~~~~~~~~~~~~~~~~~~~~TGCTATTTTTTAACAGC
JW-1288V2-86230-E-R-rc                  ~~~~~~~~~~~~~~~~~~~~~~~~~~GTGAGGTGCAGAGAGTTTGCAAACAGATTGCAATCAAACAGATCATAATCGAACAAATGCTATTTTTTAACAGC

                                                 510       520       530       540       550       560       570       580       590       600
                                        ....|....|....|....|....|....|....|....|....|....|....|....|....|....|....|....|....|....|....|....|
Aotus_1288 [Anan_2.0]                   tttactaAAGTTTAGTCTGTATATAATGAAAACCTTgcatttaatacatatattttgataaGTTTGACATATGCATATACTCATAATAATATCAccacag
JW-1288V2-crl1556-F                     TTTACTAAAGTTTAGTCTGTATATAATGAAAACTTTGCATTTAATACATATATTTTGATAAGTTTGACATATGCATATACTCATAATAATATCACCACAG
JW-1288V2-crl1556-IntF                  ~~~~~~~~~~~~~~~~~~~~~~~~~~~~~~~~~~~~~~~~~~~~~~~~~~~~~~~~~~~~~~~~~~~~~~~~~~~~~~~~~~~~~~~~~~~~~~~~~~~~
JW-1288V2-crl1556-SIntR-rc              ~~~~~~~~~~~~~~~~~~~~~~~~~~~~~~~~~~~~~~~~~~~~~~~~~~~~~~~~~~~~~~~~~~~~~~~~~~~~~~~~~~~~~~~~~~~~~~~~~~~~
JW-1288V2-crl1556-R-rc                  ~~~~~~~~~~~~~~~~~~~~~~~~~~~~~~~~~~~~~~~~~~~~~~~~~~~~~~~~~~~~~~~~~~~~~~~~~~~~~~~~~~~~~~~~~~~~~~~~~~~~
JW-1288V2-86218-F                       TTTACTAAAGTTTAGTCTGTATATAATGAAAACTTTGCATTTAATACATACATTTTGATAAGTTTGACATATGCATATACTCATAATAATATCACCACAG
JW-1288V2-86218-IntF                    ~~~~~~~~~~~~~~~~~~~~~~~~~~~~~~~~~~~~~~~~~~~~~~~~~~~~~~~~~~~~~~~~~~~~~~~~~~~~~~~~~~~~~~~~~~~~~~~~~~~~
JW-1288V2-86218-SIntR-rc                ~~~~~~~~~~~~~~~~~~~~~~~~~~~~~~~~~~~~~~~~~~~~~~~~~~~~~~~~~~~~~~~~~~~~~~~~~~~~~~~~~~~~~~~~~~~~~~~~~~~~
JW-1288V2-86218-R-rc                    ~~~~~~~~~~~~~~~~~~~~~~~~~~~~~~~~~~~~~~~~~~~~~~~~~~~~~~~~~~~~~~~~~~~~~~~~~~~~~~~~~~~~~~~~~~~~~~~~~~~~
JW-1288V2-85818-F                       TTTACTAAAGTTTAGTCTGTATATAATGAAAACCTTGCATTTAATACATATATTTTGATAAGTTTGACATATGCATATACTCATAATAATATCACCACAG
JW-1288V2-85818-IntF                    ~~~~~~~~~~~~~~~~~~~~~~~~~~~~~~~~~~~~~~~~~~~~~~~~~~~~~~~~~~~~~~~~~~~~~~~~~~~~~~~~~~~~~~~~~~~~~~~~~~~~
JW-1288V2-85818-SIntR-rc                ~~~~~~~~~~~~~~~~~~~~~~~~~~~~~~~~~~~~~~~~~~~~~~~~~~~~~~~~~~~~~~~~~~~~~~~~~~~~~~~~~~~~~~~~~~~~~~~~~~~~
JW-1288V2-85818-R-rc                    ~~~~~~~~~~~~~~~~~~~~~~~~~~~~~~~~~~~~~~~~~~~~~~~~~~~~~~~~~~~~~~~~~~~~~~~~~~~~~~~~~~~~~~~~~~~~~~~~~~~~
JW-1288V2-86100-E-F                     TTTACTAAAGTTTAGTCTGTATATAATGAAAACTTTGCATTTAATACATACATTTTGATAAGTTTGACATATGCATATACTCATAATAATATCACCACA~
JW-1288V2-86100-E-R-rc                  TTTACTAAAGTTTAGTCTGTATATAATGAAAACTTTGCATTTAATACATACATTTTGATAAGTTTGACATATGCATATACTCATAATAA
JW-1288V2-86230-E-F                     TTTACTAAAGTTTAGTCTGTATATAATGAAAACTTTGCATTTAATACATACATTTTGATAAGTTTGACATATGCATATACTCATAATAATATCACCACA~
JW-1288V2-86230-E-R-rc                  TTTACTAAAGTTTAGTCTGTATATAATGAAAACTTTGCATTTAATACATACATTTTGATAAGTTTGACATATGCATATACTCATAATAATATCACCACA~

                                                 610       620       630       640       650       660       670       680       690       700
                                        ....|....|....|....|....|....|....|....|....|....|....|....|....|....|....|....|....|....|....|....|
Aotus_1288 [Anan_2.0]                   ccgggcgcggtggctcacgcctgtaatcccagcactttgggaggccgaggcaggtggatcacgaggtcaagagattgagaccatcctggtcaacatggtg
JW-1288V2-crl1556-F                     CCGGGCGCGGTGGCTCACGCCTGTAATCCCAGCACTTTGGGAGGCCGAGGCAGGTGGATCACGAGGTCAAGAGATTGAGACCATCCTGGTCAACATGGTG
JW-1288V2-crl1556-IntF                  ~~~~~~~~~~~~~~~~~~~~~~~~~~~~~~~~~~~~~~~~~~~~~~~~~~~~~~~~~~~~~~GAGGTCAAGAGATTGAGACCATCCTGGTCAACATGGTG
JW-1288V2-crl1556-SIntR-rc              ~~~~~~~~~~~~~~~~~~~~~~~~~~~~~~~~~~~~~~~~~~~~~~~~~~~~~~~~~~~~~~~~~~~~~~~~~~~~~~~~~~~~~~~~~~~~~~~~~~~~
JW-1288V2-crl1556-R-rc                  ~~~~~~~~~~~~~~~~~~~~~~~~~~~~~~~~~~~~~~~~~~~~~~~~~~~~~~~~~~~~~~~~~~~~~~~~~~~~~~~~~~~~~~~~~~~~~~~~~~~~
JW-1288V2-86218-F                       CCGGGCGCGGTGGCTCACGCCTGTAATCCTAGCACTTTGGGAGGCCGAGGCAGGTGGATCACGAGGTCAAGAGATTGAGACCATCCTGGTCAACATGGTG
JW-1288V2-86218-IntF                    ~~~~~~~~~~~~~~~~~~~~~~~~~~~~~~~~~~~~~~~~~~~~~~~~~~~~~~~~~~~~~~GAGGTCAAGAGATTGAGACCATCCTGGTCAACATGGTG
JW-1288V2-86218-SIntR-rc                ~~~~~~~~~~~~~~~~~~~~~~~~~~~~~~~~~~~~~~~~~~~~~~~~~~~~~~~~~~~~~~~~~~~~~~~~~~~~~~~~~~~~~~~~~~~~~~~~~~~~
JW-1288V2-86218-R-rc                    ~~~~~~~~~~~~~~~~~~~~~~~~~~~~~~~~~~~~~~~~~~~~~~~~~~~~~~~~~~~~~~~~~~~~~~~~~~~~~~~~~~~~~~~~~~~~~~~~~~~~
```

```
JW-1288V2-85818-F              CCGGGCGCGGTGACTCACGCCTGTAATCCCAGCACTTTGGGAGGCCGAGGCAGGTGGATCACGAGGTCAAGAGATTGAGACCATCCTGGTCAACATGGTG
JW-1288V2-85818-IntF           ~~~~~~~~~~~~~~~~~~~~~~~~~~~~~~~~~~~~~~~~~~~~~~~~~~~~~~~~~~~~~~GAGGTCAAGAGATTGAGACCATCCTGGTCAACATGGTG
JW-1288V2-85818-SIntR-rc       ~~~~~~~~~~~~~~~~~~~~~~~~~~~~~~~~~~~~~~~~~~~~~~~~~~~~~~~~~~~~~~~~~~~~~~~~~~~~~~~~~~~~~~~~~~~~~~~~~~~~
JW-1288V2-85818-R-rc           ~~~~~~~~~~~~~~~~~~~~~~~~~~~~~~~~~~~~~~~~~~~~~~~~~~~~~~~~~~~~~~~~~~~~~~~~~~~~~~~~~~~~~~~~~~~~~~~~~~~~
JW-1288V2-86100-E-F            ~~~~~~~~~~~~~~~~~~~~~~~~~~~~~~~~~~~~~~~~~~~~~~~~~~~~~~~~~~~~~~~~~~~~~~~~~~~~~~~~~~~~~~~~~~~~~~~~~~~~
JW-1288V2-86100-E-R-rc
JW-1288V2-86230-E-F            ~~~~~~~~~~~~~~~~~~~~~~~~~~~~~~~~~~~~~~~~~~~~~~~~~~~~~~~~~~~~~~~~~~~~~~~~~~~~~~~~~~~~~~~~~~~~~~~~~~~~
JW-1288V2-86230-E-R-rc         ~~~~~~~~~~~~~~~~~~~~~~~~~~~~~~~~~~~~~~~~~~~~~~~~~~~~~~~~~~~~~~~~~~~~~~~~~~~~~~~~~~~~~~~~~~~~~~~~~~~~

                                        710       720       730       740       750       760       770       780       790       800
                               ....|....|....|....|....|....|....|....|....|....|....|....|....|....|....|....|....|....|....|....|
Aotus_1288 [Anan_2.0]          aaaccccgtctctactaaaaatacaaaaaaaaaaaaacaaaaaaaatagctgggcatggtggcgcgtgcctgtaatcccagctactcaggaggctgagac
JW-1288V2-crl1556-F            AAACCCCGTCTCTACTAAAAATACAAAAAAAAAAAAAAAAAAAAAA
JW-1288V2-crl1556-IntF         AAACCCCGTCTCTACTAAAAATACAAAAAAAAAAAAAAAAAAAAAA
JW-1288V2-crl1556-SIntR-rc     ~~~~~~~~~~~~~~~~~~~~~~~AAAAAAAAAAAAAAAAAAAAAAATAGCTGGGCATGGTGGCGCATGCCTGTAATCCCAGCTACTCAGGA
JW-1288V2-crl1556-R-rc         ~~~~~~~~~~~~~~~~~~~~~~~~~~~~~~~~~~~~~~~~~~~~~~~~~~~~~~~~~~~~~~~~~~~~~~~~~~~~~~~~~~~~~~~~~~~~~~~~~~~~
JW-1288V2-86218-F              AAACCCCGTCTCTACTAAAAATACAAAAAAAAAAAAAAAAA
JW-1288V2-86218-IntF           AAACCCCGTCTCTACTAAAAATACAAAAAAAAAAAAAA
JW-1288V2-86218-SIntR-rc       ~~~~~~~~~~~~~~~~~~~~~~~AAAAAAAAAAAAAAAAAAAAAAATAGCTGGGCATGGTGGCGCGTGCCTGTAATCCCAGCTACTCGGGAGGCTGAGGC
JW-1288V2-86218-R-rc           ~~~~~~~~~~~~~~~~~~~~~~~~~~~~~~~~~~~~~~~~~~~~~~~~~~~~~~~~~~~~~~~~~~~~~~~~~~~~~~~~~~~~~~~~~~~~~~~~~~~~
JW-1288V2-85818-F              AAACCCCGTCTCTACTAAAAATACAAAAAAAAAAAAAAAAAAAAAAAAAA
JW-1288V2-85818-IntF           AAACCCCGTCTCTACTAAAAA
JW-1288V2-85818-SIntR-rc       ~~~~~~~~~~~~~~~~AAAAAAAAAAAAAAAAAAAAAAAAAAAAAATAGCTGGGCATGGTGGCGCATGCCTGTAATCCCAGCTACTCAGGAGG
JW-1288V2-85818-R-rc           ~~~~~~~~~~~~~~~~~~~~~~~~~~~~~~~~~~~~~~~~~~~~~~~~~~~~~~~~~~~~~~~~~~~~~~~~~~~~~~~~~~~~~~~~~~~~~~~~~~~~
JW-1288V2-86100-E-F            ~~~~~~~~~~~~~~~~~~~~~~~~~~~~~~~~~~~~~~~~~~~~~~~~~~~~~~~~~~~~~~~~~~~~~~~~~~~~~~~~~~~~~~~~~~~~~~~~~~~~
JW-1288V2-86100-E-R-rc
JW-1288V2-86230-E-F            ~~~~~~~~~~~~~~~~~~~~~~~~~~~~~~~~~~~~~~~~~~~~~~~~~~~~~~~~~~~~~~~~~~~~~~~~~~~~~~~~~~~~~~~~~~~~~~~~~~~~
JW-1288V2-86230-E-R-rc         ~~~~~~~~~~~~~~~~~~~~~~~~~~~~~~~~~~~~~~~~~~~~~~~~~~~~~~~~~~~~~~~~~~~~~~~~~~~~~~~~~~~~~~~~~~~~~~~~~~~~

                                        810       820       830       840       850       860       870       880       890       900
                               ....|....|....|....|....|....|....|....|....|....|....|....|....|....|....|....|....|....|....|....|
Aotus_1288 [Anan_2.0]          aggagaattgcctgaacccaggaggcagaggttgcggtgagccgagatcgcgccattgcactccagcctgggtaacaagagcgaaactccgtctcaaaaa
JW-1288V2-crl1556-F
JW-1288V2-crl1556-IntF
JW-1288V2-crl1556-SIntR-rc
JW-1288V2-crl1556-R-rc         ~~~~~~~~~~~~~~~~~~~~~~~~~~~~~~~~~~~~~~~~~~~~~~~~~~~~~~~~~~~~~~~~~~~~~~~~~~~~~~~~~~~~~~~~~~~~~~~AAAAA
JW-1288V2-86218-F
JW-1288V2-86218-IntF
JW-1288V2-86218-SIntR-rc       AG
JW-1288V2-86218-R-rc           ~~~~~~~~~~~~~~~~~~~~~~~~~~~~~~~~~~~~~~~~~~~~~~~~~~~~~~~~~~~~~~~~~~~~~~~~~~~~~~~~~~~~~~~~~~~~~AAAAAAA
JW-1288V2-85818-F
JW-1288V2-85818-IntF
JW-1288V2-85818-SIntR-rc
JW-1288V2-85818-R-rc           ~~~~~~~~~~~~~~~~~~~~~~~~~~~~~~~~~~~~~~~~~~~~~~~~~~~~~~~~~~~~~~~~~~~~~~~~~~~~~~~~~~~~~~~~~~~~~~AAAAAA
JW-1288V2-86100-E-F            ~~~~~~~~~~~~~~~~~~~~~~~~~~~~~~~~~~~~~~~~~~~~~~~~~~~~~~~~~~~~~~~~~~~~~~~~~~~~~~~~~~~~~~~~~~~~~~~~~~~~
JW-1288V2-86100-E-R-rc
JW-1288V2-86230-E-F            ~~~~~~~~~~~~~~~~~~~~~~~~~~~~~~~~~~~~~~~~~~~~~~~~~~~~~~~~~~~~~~~~~~~~~~~~~~~~~~~~~~~~~~~~~~~~~~~~~~~~
JW-1288V2-86230-E-R-rc         ~~~~~~~~~~~~~~~~~~~~~~~~~~~~~~~~~~~~~~~~~~~~~~~~~~~~~~~~~~~~~~~~~~~~~~~~~~~~~~~~~~~~~~~~~~~~~~~~~~~~
```

```
                                          910       920       930       940       950       960       970       980       990      1000
                                 ....|....|....|....|....|....|....|....|....|....|....|....|....|....|....|....|....|....|....|....|
Aotus_1288 [Anan_2.0]            aaaaaaaaaaaaaaataataataataatgatatcaccacaatcaaggtaataaatATATCCATTGCCTTTGAAAGTTTCCTTGTGTCCCTTTGTTACTGA
JW-1288V2-crl1556-F
JW-1288V2-crl1556-IntF
JW-1288V2-crl1556-SIntR-rc
JW-1288V2-crl1556-R-rc           AAAAAAAAATAATAATAATAATAATAATGATATCACCACAATCAAGGTAAT
JW-1288V2-86218-F
JW-1288V2-86218-IntF
JW-1288V2-86218-SIntR-rc
JW-1288V2-86218-R-rc             AAAATAAATTAATAATAATAATAATAATGATATCACCANAATCAAGGT~~~~~~~~~~~~~~~~~~~~
JW-1288V2-85818-F
JW-1288V2-85818-IntF
JW-1288V2-85818-SIntR-rc
JW-1288V2-85818-R-rc             TAAAATAAAATAAAATAATAATAATAATGATATCACCACAATCAAGGTAATAAATA
JW-1288V2-86100-E-F              ~~~~~~~~~~~~~~~~~~~~~~~~~~~~~~~~~~~~~~~~ATCAAGGTAATAAATATATCCATTGCCTTTGAAAGTTTCCTTGTGTCCCTTTGTTAC
JW-1288V2-86100-E-R-rc
JW-1288V2-86230-E-F              ~~~~~~~~~~~~~~~~~~~~~~~~~~~~~~~~~~~~~~~~ATCAAGGTAATAAATATATCCATTGCCTTTGAAAGTTTCCTTGTGTCCCTTTGTTAC
JW-1288V2-86230-E-R-rc           ~~~~~~~~~~~~~~~~~~~~~~~~~~~~~~~~~~~~~~~~ATCAAGGTAA
```

Figure S28. Sequence alignment for *Alu* locus *Aotus*_1288_V2 018488858.1:16800884-16802399. PCR-based genotypes indicate the target *Alu* insertion is homozygous present (1/1) in all *Aotus* samples with the exception of two *A. vociferans*, 86100 and 86230 who displayed only the empty site (0/0) or homozygous absent for the target insertion (See main text Figure 2a). DNA sequencing was performed to confirm this pattern. The reference genome [Anan_2.0] is shown at the top followed by *A. trivirgatus* crl-1556, *A. vociferans* 86218 and *A. azarae* 85818 all of whom share the target *Alu* insertion starting at position 600 (grey highlight) and flanked by TSDs in yellow highlight. This *Alu* element has a particularly long middle A-rich region that was difficult to sequence through, but the internal-*Alu* primer sequences complete the entire element. Empty site amplicons for *A. vociferans* 86100 and 86230 display precise pre-integration sites with sequence traces spanning the target region, confirming the genotypes. This pattern suggests that *A. vociferans* is basal among the *Aotus* species on our DNA panel. However, individuals 86100 and 86230 are known to be full siblings (sister and brother, respectively) having the same Dam and Sire. Therefore, this locus should be applied to wild *Aotus* populations for evaluation.

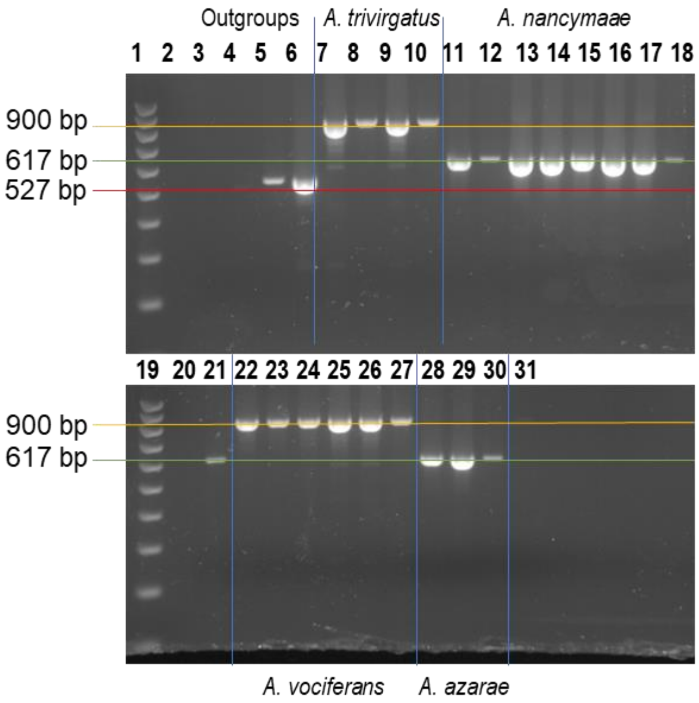

Figure S29a: *Alu* locus Owl_2LS_042_018498342.1:691757-693258 (LR-42): Lanes: 1, 19- 100 bp DNA ladder, 2, 20-blank, 3- TLE (negative control), 4- Human (HeLa), 5- *Callithrix jacchus* (Common marmoset), 6- *Saimiri s. sciureus* (Common squirrel monkey), 7-10 *A. trivirgatus*, 11-18, 21 *A. nancymaae*, 22-27 *A. vociferans*, 28-30 *A. azarae*, 31-*A. lemurinus griseimembra.* At first glance, this gel image seems to indicate that *A. trivirgatus* and *A. vociferans* contain the *Alu* insertion while *A. nancymaae* and *A. azarae* do not. However, the predicted amplicon sizes are 617 bp (green line) for the *A. nancymaae* reference genome containing a full length *Alu* insertion and 527 bp (red line) for the squirrel monkey outgroup. The amplicon sizes are not 300 bp smaller in the outgroups due to extra sequence that is not present in *Aotus*. *A. trivirgatus* and *A. vociferans* have a larger amplicon (900-1000 bp, yellow line) that was suspected to be a 2nd *Alu* element, in addition to the target, based on the size difference. However, DNA sequencing revealed that these larger amplicons do not contain a 2nd *Alu*, but rather include ~225 bp of extra non-repeat sequence about 80 bp 5’ of the reverse primer and about 100 bp downstream of the target (see alignment Figure S29b.) The target *Alu* is likely fixed present in *Aotus*. Evidence of shared extra sequence in both *A. trivirgatus* and *A. vociferans* implies a close relationship but it does not represent an additional homoplasy free element. Vertical blue lines superimposed on the gel image visually separate the species groups. Gel electrophoresis for this image consisted of 175V for 90 minutes.

```
                                       410       420       430       440       450       460       470       480       490       500
                              ....|....|....|....|....|....|....|....|....|....|....|....|....|....|....|....|....|....|....|....|
Owl_2LS_042_[Anan_2.0]        TAAGAGGAACTGAGGAGAGTGCCTCAGGATAGATAGACATTTGGGCCAATTTCATCATGTTTTTATAATGCTACACAGTCTTGATTTAGAGGGTAACAGG
LR-42-crl1556-R-rc            ~~~~~~~~~~~~~~~~~~~~~~~~~~~~~~~~~~~~~~~~~~~~~~~~~~~~~~~~~~~~~~~~~~~~~~~~~~~~~~~~~~~~~~~~~~~~~~~~~~~~
LR-42-86218-R-rc              ~~~~~~~~~~~~~~~~~~~~~~~~~~~~~~~~~~~~~~~~~~~~~~~~~~~~~~~~~~~~~~~~~~~~~~~~~~~~~~~~~~~~~~~~~~~~~~~~~~~~
LR-42-crl1556-F               ~~~~~~~~~~~~~~~~~~~~~~~~~~~~~~~~~~~~~~~~~~~~~~~~~~~~~~~~~~~~~~~~~~~~~~~~~~~~~~~~~~~~~~~~~~~~~~~~~~~~
LR-42-crl1556-IntF            ~~~~~~~~~~~~~~~~~~~~~~~~~~~~~~~~~~~~~~~~~~~~~~~~~~~~~~~~~~~~~~~~~~~~~~~~~~~~~~~~~~~~~~~~~~~~~~~~~~~~
LR-42-crl1556-SIntR-rc        ~~~~~~~~~~~~~~~~~~~~~~~~~~~~~~~~~~~~~~~~~~~~~~~~~~~~~~~~~~~~~~~~~~~~~~~~~~~~~~~~~~~~~~~~~~GGGTAACAGG
LR-42-86218-F                 ~~~~~~~~~~~~~~~~~~~~~~~~~~~~~~~~~~~~~~~~~~~~~~~~~~~~~~~~~~~~~~~~~~~~~~~~~~~~~~~~~~~~~~~~~~~~~~~~~~~~
LR-42-86218-IntF              ~~~~~~~~~~~~~~~~~~~~~~~~~~~~~~~~~~~~~~~~~~~~~~~~~~~~~~~~~~~~~~~~~~~~~~~~~~~~~~~~~~~~~~~~~~~~~~~~~~~~
LR-42-86218-SIntR-rc          ~~~~~~~~~~~~~~~~~~~~~~~~~~~~~~~~~~~~~~~~~~~~~~~~~~~~~~~~~~~~~~~~~~~~~~~~~~~~~~~~~~~~~~~~~~GGGTAACAGG

                                       510       520       530       540       550       560       570       580       590       600
                              ....|....|....|....|....|....|....|....|....|....|....|....|....|....|....|....|....|....|....|....|
Owl_2LS_042_[Anan_2.0]        CTTTGACAGTTTCTAATTCTACATTCTAAGCATCACTAATTCAAGCTATAATAATGGCTCTGGGATCTGTATCTAAACTTAGTATAAGATTTCAAgatcg
LR-42-crl1556-R-rc            ~~~~~~~~~~~~~~~~~~~~~~~~~~~~~~~~~~~~~~~~~~~~~~~~~~~~~~~~~~~~~~~~~~~~~~~~~~~~~~~~~~~~~~~~~~~~~~~~~~~~
LR-42-86218-R-rc              ~~~~~~~~~~~~~~~~~~~~~~~~~~~~~~~~~~~~~~~~~~~~~~~~~~~~~~~~~~~~~~~~~~~~~~~~~~~~~~~~~~~~~~~~~~~~~~~~~~~~
LR-42-crl1556-F               ~~~~~~~~~~~~~~~~~~~~~~~~~~~~~~~~~~~~~~~~~~~~~~~~~~~~~~~GGCTCTGGGATCTGTATCTAAACTTAGTATAAGATTTCAAGATCG
LR-42-crl1556-IntF            ~~~~~~~~~~~~~~~~~~~~~~~~~~~~~~~~~~~~~~~~~~~~~~~~~~~~~~~~~~~~~~~~~~~~~~~~~~~~~~~~~~~~~~~~~~~~~~~~~~~~
LR-42-crl1556-SIntR-rc        CTTTGACAGTTTCTAATTCTACATTCTAAGCATCACTAATTCAAGCTATAATAATGGCTCTGGGATCTGTATCTAAACTTAGTATAAGATTTCAAGATCG
LR-42-86218-F                 ~~~~~~~~~~~~~~~~~~~~~~~~~~~~~~~~~~~~~~~~~~~~~~TATAATAATGGCTCTGGGATCTGTATCTAAACTTAGTATAAGATTTCAAGATCG
LR-42-86218-IntF              ~~~~~~~~~~~~~~~~~~~~~~~~~~~~~~~~~~~~~~~~~~~~~~~~~~~~~~~~~~~~~~~~~~~~~~~~~~~~~~~~~~~~~~~~~~~~~~~~~~~~
LR-42-86218-SIntR-rc          CTTTGACAGTTTCTAATTCTACATTCTAAGCATCACTAATTCAAGCTATAATAATGGCTCTGGGATCTGTATCTAAACTTAGTATAAGATTTCAAGATCG

                                       610       620       630       640       650       660       670       680       690       700
                              ....|....|....|....|....|....|....|....|....|....|....|....|....|....|....|....|....|....|....|....|
Owl_2LS_042_[Anan_2.0]        gctgggcgcggtggctcaagcctgtaatcccagcactttgggaggccgaggcgggtggatcatgaggtcaagagatcgagaccatcctggtcaacatggt
LR-42-crl1556-R-rc            ~~~~~~~~~~~~~~~~~~~~~~~~~~~~~~~~~~~~~~~~~~~~~~~~~~~~~~~~~~~~~~~~~~~~~~~~~~~~~~~~~~~~~~~~~~~~~~~~~~~~
LR-42-86218-R-rc              ~~~~~~~~~~~~~~~~~~~~~~~~~~~~~~~~~~~~~~~~~~~~~~~~~~~~~~~~~~~~~~~~~~~~~~~~~~~~~~~~~~~~~~~~~~~~~~~~~~~~
LR-42-crl1556-F               GCTGGGCGCGGTGGCTCAAGCCTGTAATCCCAGCACTTTGGGAGGCCGAGGCGGGTGGATCACGAGGTCAAGAGATCGAGACCATCCTGGTCAACATGGT
LR-42-crl1556-IntF            ~~~~~~~~~~~~~~~~~~~~~~~~~~~~~~~~~~~~~~~~~~~~~~~~~~~~~~~~~~~~~~~GAGGTCNAGAGATCGAGACCATCCTGGTCAACATGGT
LR-42-crl1556-SIntR-rc        GCTGGGCGCGGTGGCTCAAGCCTGTAATCCCAGCACTTTGGGAGGCCGAGGCGGGTGGATCACGAGGTCAAGAGATCGAGACCATCCTGGTCAACATGGT
LR-42-86218-F                 GCTGGGCGCGGTGGCTCAAGCCTGTAATCCCAGCACTTTGGGAGGCCGAGGCGGGTGAATCACGAGGTCAAGAGATCGAGACCATCCTGGTCAACATGGT
LR-42-86218-IntF              ~~~~~~~~~~~~~~~~~~~~~~~~~~~~~~~~~~~~~~~~~~~~~~~~~~~~~~~~~~~~~~~~~~~~~~~~~~~~CGAGACCATCCTGGTCAACATGGT
LR-42-86218-SIntR-rc          GCTGGGCGCGGTGGCTCAAGCCTGTAATCCCAGCACTTTGGGAGGCCGAGGCGGGTGAATCACGAGGTCAAGAGATCGAGACCATCCTGGTCAACATGGT

                                       710       720       730       740       750       760       770       780       790       800
                              ....|....|....|....|....|....|....|....|....|....|....|....|....|....|....|....|....|....|....|....|
Owl_2LS_042_[Anan_2.0]        gaaaccccgtccctactaaaaaatacaaaaaattagctgggcatggtggcgcgtgcctgtaatcccagctactcaggaggctgaggcaggagaattgcct
LR-42-crl1556-R-rc            ~~~~~~~~~~~~~~~~~~~~~~~~~~~~~~~~~~~~~~~~~~~~~~~~~~~~~~~~~~~~~~~~~~~~~~~~~~~~~~~~~~~~~~~~~~~~~~~~~~~~
LR-42-86218-R-rc              ~~~~~~~~~~~~~~~~~~~~~~~~~~~~~~~~~~~~~~~~~~~~~~~~~~~~~~~~~~~~~~~~~~~~~~~~~~~~~~~~~~~~~~~~~~~~~~~~~~~~
LR-42-crl1556-F               GAAACCCCGTCCCTACTAAAAAATACAAAAAATTAGCTGGGCACGGTGGCGCGTGCCTGTAATCCCAGCTACTCAGGAGGCTGAGGCAGGAGAATTGCCT
LR-42-crl1556-IntF            GAAACCCCGTCCCTACTAAAAAATACAAAAAATTAGCTGGGCACGGTGGCGCGTGCCTGTAATCCCAGCTACTCAGGAGGCTGAGGCAGGAGAATTGCCT
LR-42-crl1556-SIntR-rc        GAAACCCCGTCCCTACTAAAAAATACAAAAAATTAGCTGGGCACGGTGGCGCGTGCC
LR-42-86218-F                 GAAACCCCGTCCCTACTAAAAAATACAAAAAATTAGCTGGGCATGGTGGCGCGTGCCTGTAATCCCAGCTACTCAGGAGGCTGAGGCAGGAGAATTGCCT
LR-42-86218-IntF              GAAACCCCGTCCCTACTAAAAAATACAAAAAATTAGCTGGGCACGGTGGCGCGTGCCTGTAATCCCAGCTACTCAGGAGGCTGAGGCAGGAGAATTGCCT
LR-42-86218-SIntR-rc          GAAACCCCGTCCCTACTAAAAAATACAAAAAATTAGCTGGGCACGGTGGCGCGTGCCTGTAATCCCAGCTACTCAGGAGG
```

```
                                    810       820       830       840       850       860       870       880       890       900
                           ....|....|....|....|....|....|....|....|....|....|....|....|....|....|....|....|....|....|....|....|
Owl_2LS_042_[Anan_2.0]     gaaccccaggaggcggaggttgcggtgagtcgagatcgcgccattgcactccagcctgggtaacaagagcgaaactccgtctcaaaaaaaaaaaaaaaaa
LR-42-crl1556-R-rc         ~~~~~~~~~~~~~~~~~~~~~~~~~~~~~~~~~~~~~~~~~~~~~~~~~~~~~~~~~~~~~~~~~~~~~~~~~~~~~~~~~~~~~AAAAAAAAAAAAAAA
LR-42-86218-R-rc           ~~~~~~~~~~~~~~~~~~~~~~~~~~~~~~~~~~~~~~~~~~~~~~~~~~~~~~~~~~~~~~~~~~~~~~~~~~~~~~~~~~~~~~~~~~~~~~~~~~~~
LR-42-crl1556-F            GAACCCCAGGAGGCGGAGGTTGCGGTGAGCCGAGATCGCGCCATTGCACTCCAGCCTGGGTAACAAGAGCGAAACTCCGTCTCAAAAAAAAAAAAAAA
LR-42-crl1556-IntF         GAACCCCAGGAGGCGGAGGTTGCGGTGAGCCGAGATCGCGCCATTGCACTCCAGCCTGGGTAACAAGAGCGAAACTCCGTCTCAAAAAAAAAAAAAAA
LR-42-crl1556-SIntR-rc
LR-42-86218-F              GAACCCCAGGAGGCGGAGGTTGCGGTGAGCCGAGATCGCGCCATTGCACTCCAGCCTGGGTAACAAGAGCGAAACTCCGTCTCAAAAAAAAAAAAAAA
LR-42-86218-IntF           GAACCCCAGGAGGCGGAGGTTGCGGTGAGCCGAGATCGCGCCATTGCACTCCAGCCTGGGTAACAAGAGCGAAACTCCATCTCAAAAAAAAAAAAAAA
LR-42-86218-SIntR-rc

                                    910       920       930       940       950       960       970       980       990       1000
                           ....|....|....|....|....|....|....|....|....|....|....|....|....|....|....|....|....|....|....|....|
Owl_2LS_042_[Anan_2.0]     aggtttcaagaTTATGGTATATTGAGATTCAGGCTGAACAGATGAAAGGTTTTGGCAAAAAAGGACCCAAGGACGGGAAagatatttctgctttttatta
LR-42-crl1556-R-rc         AGGTTTCAAGATTATGGTATATTGAGATTCAGGCTGAACAGATGAAAGGTTTTGGCAAAAAAGGACCCAAGGACGGGAAAGATATTTCTGCTTTTTATTA
LR-42-86218-R-rc           ~~~~~~~~~~~~~~~~~~~~~~~~~~~~~~~~~~~~~~~~~~~~~~~~~~~~~~~~~~~~~~~~~~~~~~~~~~~~~~~~~~~~~~~~~~~~~~~~~~~~
LR-42-crl1556-F
LR-42-crl1556-IntF
LR-42-crl1556-SIntR-rc
LR-42-86218-F
LR-42-86218-IntF
LR-42-86218-SIntR-rc

                                    1010      1020      1030      1040      1050      1060      1070      1080      1090      1100
                           ....|....|....|....|....|....|....|....|....|....|....|....|....|....|....|....|....|....|....|....|
Owl_2LS_042_[Anan_2.0]     a~~~~~~~~~~~~~~~~~~~~~~~~~~~~~~~~~~~~~~~~~~~~~~~~~~~~~~~~~~~~~~~~~~~~~~~~~~~~~~~~~~~~~~~~~~~~~~~~~~~
LR-42-crl1556-R-rc         ATGTTTTACTTGAAGTATCTTTTTGGTTGCTTTTACTTTTTCAAAGTAAATTTTCAAATTATCAAGCTATCTTTGGCTTGATAATTTGAAGATTTAATTA
LR-42-86218-R-rc           ~~~~~~~~~~~~~~~~~~~~~~~~~~~~~~~~~~~~~~~~~~AAAGTAAATTTTCAAATTATCAAGCTATCTTTGTCTTGATAATTTGAAGATTTAATTA
LR-42-crl1556-F
LR-42-crl1556-IntF
LR-42-crl1556-SIntR-rc
LR-42-86218-F
LR-42-86218-IntF
LR-42-86218-SIntR-rc

                                    1110      1120      1130      1140      1150      1160      1170      1180      1190      1200
                           ....|....|....|....|....|....|....|....|....|....|....|....|....|....|....|....|....|....|....|....|
Owl_2LS_042_[Anan_2.0]     ~~~~~~~~~~~~~~~~~~~~~~~~~~~~~~~~~~~~~~~~~~~~~~~~~~~~~~~~~~~~~~~~~~~~~~~~~~~~~~~~~~~~~~~~~~~~~~~~~~~~
LR-42-crl1556-R-rc         ATTATTATACACTGAACATATGTGTGCATTGTGAGCACACATAAAGGAATGTTAAAGTGCCTCATAGGAATTTCTCCTCTATCAACTCAAGAATCGTATG
LR-42-86218-R-rc           ATTATTATACACTGAACATATGTGTGCATTGTGAGCACACATAAAGGAATGTTAAAGTGCCTCATAGGAATTTCTCCTCTATCAACTCAAGAATCGTATG
LR-42-crl1556-F
LR-42-crl1556-IntF
LR-42-crl1556-SIntR-rc
LR-42-86218-F
LR-42-86218-IntF
LR-42-86218-SIntR-rc
```

```
                      1210      1220      1230      1240      1250      1260      1270      1280      1290      1300
                 ....|....|....|....|....|....|....|....|....|....|....|....|....|....|....|....|....|....|....|....|
Owl_2LS_042_[Anan_2.0]       ~~~~~~~~~~~~~~~~~~~~~~~~~~gGAAACTGACTCAATCATGGCCCCCAGGGTCCCTTGAGTCTAGAAGCAATCACTGCTCCTGGAACTTTGCCCCC
LR-42-crl1556-R-rc           TTGTCTAGGTACTGATATGTATAGAAGGAAACTGACTCAACCATGGCCCCCAGGGTCCCTTGAGTCTAGAAGCAA
LR-42-86218-R-rc             TTGTCTAGGTACTGATATGTATAGAAGGAAACTGACTCAACCATGGCCCCCAGGGTCCCTTGAGTCTAGAAGCAA
LR-42-crl1556-F
LR-42-crl1556-IntF
LR-42-crl1556-SIntR-rc
LR-42-86218-F
LR-42-86218-IntF
LR-42-86218-SIntR-rc
```

Figure S29b. Sequence alignment for *Alu* locus Owl_2LS_042_018498342.1:691757-693258 (LR-42). The reference genome [Anan_2.0] is shown at the top followed by samples *A. trivirgatus* crl-1556 and *A. vociferans* 86218. The target *Alu* insertion starts at position 600 (grey highlight) and ends in an A-tail at ~900 and is flanked by matching sequence indicating that these samples share the target insertion. The TSDs are in yellow highlight (AAGG/ATTTCAAGATC/T). However, beginning at position ~1002, *A. trivirgatus* crl-1556 and *A. vociferans* 86218 have a ~225 bp insertion of extra non-repeat (based on RepeatMasker) sequence (aqua highlight) that is about 80 bp 5’ of the reverse primer (not shown). The target *Alu* is likely homozygous present within *Aotus*. Evidence of shared extra sequence in both *A. trivirgatus* and *A. vociferans* hints at a close relationship but it does not represent an additional homoplasy free element. DNA sequencing using internal *Alu* primers easily detects if multiple *Alus* are present versus having a single signal. There is only one *Alu* at this locus, the target insertion.

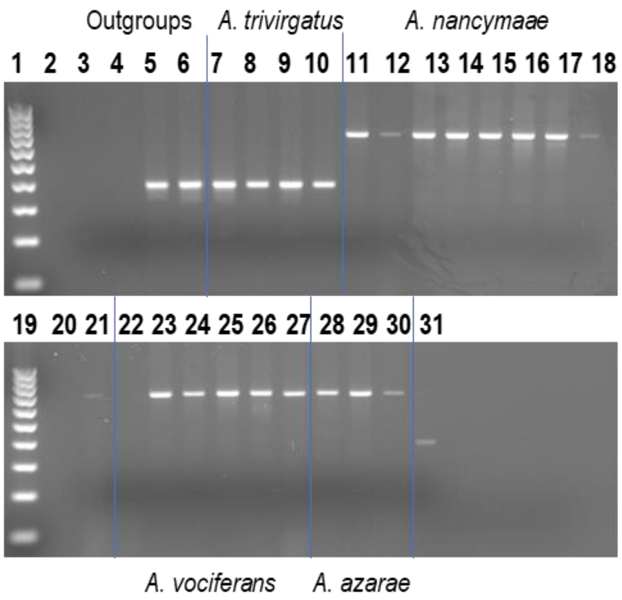

Figure S30a: *Alu* locus *Aotus*_127_018503376.1:406405-407905 (JW-127): Lanes: 1, 19- 100 bp DNA ladder, 2, 20-blank, 3- TLE (negative control), 4- Human (HeLa), 5- *Callithrix jacchus* (Common marmoset), 6- *Saimiri s. sciureus* (Common squirrel monkey), 7-10 *A. trivirgatus*, 11-18, 21 *A. nancymaae*, 22-27 *A. vociferans*, 28-30 *A. azarae*, 31-*A. lemurinus griseimembra*. Vertical blue lines superimposed on the gel image visually separate the species groups. The predicted amplicon sizes are 690/380 bp. These PCR genotypes indicate that the target *Alu* insertion is homozygous present in *A. nancymaae*, *A. vociferans* and *A. azarae* while homozygous absent in *A. trivirgatus* and *A. lemurinus griseimembra*. Similar to locus *Aotus*_1602, this suggests that *A. trivirgatus* is basal to *A. vociferans*. Perform DNA sequencing to confirm this pattern.

```
                                    510       520       530       540       550       560       570       580       590       600
                           ....|....|....|....|....|....|....|....|....|....|....|....|....|....|....|....|....|....|....|....|
Aotus_127_[Anan_2.0]       ATATCTATTAGTTGGTGTGAAAgtgcaaaaacc~gcaattacttttgcaccaacctcatagcaaataaaattgaaatcatttctaaaataaagatatttc
JW-127-crl1556-E-R         ATATCTATTAGTTGGTGTGAAAGTGCAAAAACC~GCAATTACTTCTGCACCAACCTCATATCAAATAAAATTGAAATCATTTCTAAAATAAAGATATTTC
JW-127-crl1556-E-F-rc      ATATCTATTAGTTGGTGTGAAAGTGCAAAAACC~GCAATTACTTCTGCACCAACCTCATATCAAATAAAATTGAAATCATTTCTAAAATAAAGATATTTC
JW-127-155159-SIntR-rc     ATATCTATTAGTTGGTGTGAAAGTGCAAAAACC~GCAATTACTTTTGCACCAACCTCATAGCAAATAAAATTGAAATCATTTCTAAAATAAAGATATTTC
JW-127-155159-R            ATATCTATTAGTTGGTGTGAAAGTGCAAAAACC
JW-127-155159-IntF         ~~~~~~~~~~~~~~~~~~~~~~~~~~~~~~~~~~~~~~~~~~~~~~~~~~~~~~~~~~~~~~~~~~~~~~~~~~~~~~~~~~~~~~~~~~~~~~~~~~~~
JW-127-155159-F-rc         ~~~~~~~~~~~~~~~~~~~~~~~~~~~~~~~~~~~~~~~~~~~~~~~~~~~~~~~~~~~~~~~~~~~~~~~~~~~~~~~~~~~~~~~~~~~~~~~~~~~~
JW-127-85818-SIntR-rc      ATATCTATTAGTTGGTGTGAAAGTGCAAAAACCCGCAATTACTTTTGCACCAACCTCATAGCAAATAAAATTGAAATCATTTCTAAAATAAAGATATTTC
JW-127-85818-R             ATATCTATTAGTTGGTGTGAAAGTGCAAAAACCCGCAATTACTTTTGCACCAACCTCATAGCAAATAAAATTG
JW-127-85818-IntF          ~~~~~~~~~~~~~~~~~~~~~~~~~~~~~~~~~~~~~~~~~~~~~~~~~~~~~~~~~~~~~~~~~~~~~~~~~~~~~~~~~~~~~~~~~~~~~~~~~~~~
JW-127-85818-F-rc          ~~~~~~~~~~~~~~~~~~~~~~~~~~~~~~~~~~~~~~~~~~~~~~~~~~~~~~~~~~~~~~~~~~~~~~~~~~~~~~~~~~~~~~~~~~~~~~~~~~~~
```

```
                                    610       620       630       640       650       660       670       680       690       700
                           ....|....|....|....|....|....|....|....|....|....|....|....|....|....|....|....|....|....|....|....|
Aotus_127_[Anan_2.0]       cggccgggcgcggtggctcacgcctgtaatcccagcactttgggaggccgaggcgggtggatcacgaggtcgagagatcgagaccatcctggtcaacatg
JW-127-crl1556-E-R         C~~~~~~~~~~~~~~~~~~~~~~~~~~~~~~~~~~~~~~~~~~~~~~~~~~~~~~~~~~~~~~~~~~~~~~~~~~~~~~~~~~~~~~~~~~~~~~~~~~~
JW-127-crl1556-E-F-rc      C~~~~~~~~~~~~~~~~~~~~~~~~~~~~~~~~~~~~~~~~~~~~~~~~~~~~~~~~~~~~~~~~~~~~~~~~~~~~~~~~~~~~~~~~~~~~~~~~~~~
JW-127-155159-SIntR-rc     CGGCCGGGCGTGGTGGCTCACGCCTGTAATCCCAGCACTTTGGGAGGCCGAGGCGGGTGGATCACGAGGTCAAGAGATCGAGACCATCCTGGTCAACATG
JW-127-155159-R
JW-127-155159-IntF         ~~~~~~~~~~~~~~~~~~~~~~~~~~~~~~~~~~~~~~~~~~~~~~~~~~~~~~~~~~~~~~~~~GAGGTCAAGAGATCGAGACCATCCTGGTCAACATG
JW-127-155159-F-rc         ~~~~~~~~~~~~~~~~~~~~~~~~~~~~~~~~~~~~~~~~~~~~~~~~~~~~~~~~~~~~~~~~~~~~~~~~~~~~~~~~~~~~~~~~~~~~~~~~~~~~
JW-127-85818-SIntR-rc      CGGCCAGGCGTGGTGGCTCACGCCTGTAATCCCAGCACTTTGGGAGGCCGAGGCGGGTGGATCACGAGGTCAGGAGATCAAGACCATCCTGGTCAACATG
JW-127-85818-R
JW-127-85818-IntF          ~~~~~~~~~~~~~~~~~~~~~~~~~~~~~~~~~~~~~~~~~~~~~~~~~~~~~~~~~~~~~~~~~GAGGTCAGGAGATCAAGACCATCCTGGTCAACATG
JW-127-85818-F-rc          ~~~~~~~~~~~~~~~~~~~~~~~~~~~~~~~~~~~~~~~~~~~~~~~~~~~~~~~~~~~~~~~~~~~~~~~~~~~~~~~~~~~~~~~~~~~~~~~~~~~~
```

```
                                    710       720       730       740       750       760       770       780       790       800
                           ....|....|....|....|....|....|....|....|....|....|....|....|....|....|....|....|....|....|....|....|
Aotus_127_[Anan_2.0]       gtgaaaccccgtctctactaaaaatacaaaaaattagctgggcgtggtggcgcgtgcctgtaattccagctactcaggaggctgaggcaggagaattgcc
JW-127-crl1556-E-R         ~~~~~~~~~~~~~~~~~~~~~~~~~~~~~~~~~~~~~~~~~~~~~~~~~~~~~~~~~~~~~~~~~~~~~~~~~~~~~~~~~~~~~~~~~~~~~~~~~~~~
JW-127-crl1556-E-F-rc      ~~~~~~~~~~~~~~~~~~~~~~~~~~~~~~~~~~~~~~~~~~~~~~~~~~~~~~~~~~~~~~~~~~~~~~~~~~~~~~~~~~~~~~~~~~~~~~~~~~~~
JW-127-155159-SIntR-rc     GTGAAACCCCGTCTCTACTAAAAATACAAAAAATTAGCTGGGCATGGTGGCGCGTGCCTGTAATCCCAGCTACTCAAGAGGCTGAGGCAG
JW-127-155159-R
JW-127-155159-IntF         GTGAAACCCCGTCTCTACTAAAAATACAAAAAATTACCTGGGCATGGTGGCGCGTGCCTGTAATCCCAGCTACTCAAGAGGCTGAGGCAGGAGAATTGCC
JW-127-155159-F-rc         ~~~~~~~~~~~~~~~~~~~~~~~~~~~~~~~~~~~~~~~~~~~~~~~~~~~~~~~~~~~~~~~~~~~~~~~~~~~~~~~~~~~~~~~~~~~~~~~~~~~~
JW-127-85818-SIntR-rc      GTGAAACCTCGTCTCTACTAAAAATACAAAAAATTAGCTGGGCATGGTGGCGCGTGCCTGTAATCCCAGCTACTCAGGAGGCTGAGGCAGG
JW-127-85818-R
JW-127-85818-IntF          GTGAAACCTCGTCTCTACTAAAAATACAAAAAATAACCTGGGCATGGTGGCGCGTGCCTGTAATCCCAGCTACTCAGGAGGCTGAGGCAGGAGAATTGCC
JW-127-85818-F-rc          ~~~~~~~~~~~~~~~~~~~~~~~~~~~~~~~~~~~~~~~~~~~~~~~~~~~~~~~~~~~~~~~~~~~~~~~~~~~~~~~~~~~~~~~~~~~~~~~~~~~~
```

```
                                810       820       830       840       850       860       870       880       890       900
                        ....|....|....|....|....|....|....|....|....|....|....|....|....|....|....|....|....|....|....|....|
Aotus_127_[Anan_2.0]    tgaacccaggaggcggaggttgcggtgagccgagatcgcgccattgcactccagcctgggcaacaagagcgaaactccgtctcaaaaaaaaaaaaaaaaa
JW-127-crl1556-E-R      ~~~~~~~~~~~~~~~~~~~~~~~~~~~~~~~~~~~~~~~~~~~~~~~~~~~~~~~~~~~~~~~~~~~~~~~~~~~~~~~~~~~~~~~~~~~~~~~~~~~~
JW-127-crl1556-E-F-rc   ~~~~~~~~~~~~~~~~~~~~~~~~~~~~~~~~~~~~~~~~~~~~~~~~~~~~~~~~~~~~~~~~~~~~~~~~~~~~~~~~~~~~~~~~~~~~~~~~~~~~
JW-127-155159-SIntR-rc
JW-127-155159-R
JW-127-155159-IntF      TGAACCCAGGAGGCGGAGGTTGCGGTGAGCCGAGATCGTGCCATTGCACTCCAGTCTGGGTAACAAGAGCG
JW-127-155159-F-rc      ~~~~~~~~~~~~~~~~~~~~~~~~~~~~~~~~~~~~~~~~~~~~~~~~~~~~~~~~~~~~~~~~~~~~~~~~~~~~~~~~~~~~~~~~~~AAAAAAAAAA
JW-127-85818-SIntR-rc
JW-127-85818-R
JW-127-85818-IntF       TGAACCCAGGAGGCGGAGGTTGTGGTGAGCCGAGATCGCGCCATTGCACTCCAGCCTGGGTAACAAGAGC
JW-127-85818-F-rc       ~~~~~~~~~~~~~~~~~~~~~~~~~~~~~~~~~~~~~~~~~~~~~~~~~~~~~~~~~~~~~~~~~~~~~~~~~~~~~AAAAAAAAAAAAAAAAAAAAAAA

                                910       920       930       940       950       960       970       980       990       1000
                        ....|....|....|....|....|....|....|....|....|....|....|....|....|....|....|....|....|....|....|
Aotus_127_[Anan_2.0}    aagatatttccatCACAAGGACTAGACAAGTTAGTTTACAGGTTTACAAGCTAAAGCAAATTTAGAATGCTAAAGATGACTACTTTTCATTATTCTCTGC
JW-127-crl1556-E-R      ~~~~~~~~~~~ATCACAAGGACTAGACAAGTTAGTTTACAGGTTTACAAGCTAAAGCAAATTTAGAATGCTAAAGATGACTACTTTTCATTATTCTCTGC
JW-127-crl1556-E-F-rc   ~~~~~~~~~~~ATCACAAGGACTAGACAAGTTAGTTTACAGGTTTACAAGCTAAAGCAAATTTAGAATGGTAAAGATGACTACTTTTCATTATTCTCTGC
JW-127-155159-SIntR-rc
JW-127-155159-R
JW-127-155159-IntF
JW-127-155159-F-rc      AGGATATTTCCATCACAAGGNCNNNNNNNNTTAGTTTACAGGTTTACAAGCTAAAGCAAATNTNNNNNNNNAAAGATGACTACTTTTCATTATTCTCTGC
JW-127-85818-SIntR-rc
JW-127-85818-R
JW-127-85818-IntF
JW-127-85818-F-rc       AAAATATTTCCATCACAAGGACTANANAAGTTAGTTTACAGATTTACAAGCTAAAGCAAATTTANAANNNNNAAGATGACTACTTTTCATTATTCTCTGC
```

Figure S30b. Sequence alignment for *Alu* locus *Aotus*_127_018503376.1:406405-407905 (JW-127). PCR based genotypes indicate the target *Alu* is homozygous present (1/1) in all *A. nancymaae*, *A. vociferans* and *A. azarae* individuals while homozygous absent (0/0) in all *A. trivirgatus* individuals and the *A. l. griseimembra* sample. DNA sequencing was performed to confirm this pattern. The reference genome [Anan_2.0] is shown at the top followed by the empty site sequence for *A. trivirgatus* crl-1556 and filled site amplicons for *A. vociferans* MVZ-155159 and *A. azarae* 85818 both of whom share the target *Alu* starting at position at 602 (grey highlight) and flanked by TSDs in yellow highlight. Sequencing of the empty site amplicon in *A. trivirgatus* crl-1556 has a precise pre-integration site spanning the target region, confirming the genotypes. This *Alu* insertion/absence pattern supports *A. trivirgatus* as basal to *A. vociferans* rather than the other way around, evidence of incomplete lineage sorting among grey-necked species.

```
                                      510       520       530       540       550       560       570       580       590       600
                                 ....|....|....|....|....|....|....|....|....|....|....|....|....|....|....|....|....|....|....|....|
Aotus_1602 [Anan_2.0]            TCTATTACTATTTTCCAATTCAGACAGTCAGAGAGTTGAAGTAACTTgattaaggtcacacagccagtaagtggcaagGCTCAGATAAAAGTCACatgcg
JW-1602-86230-SIntR-rc           TCTATTACTATTTTCCAATTCAGACAGTCAGAGAGTTGAAGTAACTTGATTAAGGTCACACAGCCAGTAAGTGGCAAGGCTCAGATAAAAGTCACATGCG
JW-1602-86230-F                  TCTATTACTATTTTCCAATTCAGACAGTCAGAGAGTTGAAGTAACTTGATTAAGGTCACACAGCCAGTAAGTGGCAAGGCTCAGATAAAAGTCACATGCG
JW-1602-86230-IntF               ~~~~~~~~~~~~~~~~~~~~~~~~~~~~~~~~~~~~~~~~~~~~~~~~~~~~~~~~~~~~~~~~~~~~~~~~~~~~~~~~~~~~~~~~~~~~~~~~~~~~
JW-1602-85818-SIntR-rc           TCTATTACTATTTTCCAATTCAGACAGTCAGAGAGTTGAAGTAACTTGATTAAGGTCACACAGCCAGTAAGTGGCAAGGCTCAGATAAAAGTCACATGCG
JW-1602-85818-F                  TCTATTACTATTTTCCAATTCAGACAGTCAGAGAGTTGAAGTAACTTGATTAAGANCACACAGCCAGTAAGTGGCAAGGCTCAGATAAAAGTCACATGCG
JW-1602-85818-IntF               ~~~~~~~~~~~~~~~~~~~~~~~~~~~~~~~~~~~~~~~~~~~~~~~~~~~~~~~~~~~~~~~~~~~~~~~~~~~~~~~~~~~~~~~~~~~~~~~~~~~~
JW-1602-crl1556-E-F              TCTAGTACTATTTTCCAATTCAGACAGTCAGAGAGTTGAAGTAACTTGATTAAGGTCACACAGCCAGTAAGTGGCAAGGCTCAGATAAAAGTCACATGC~
JW-1602-crl1556-E-R-rc           TCTAGTACTATTTTCCAATTCAGACAGTCAGAGAGTTGAAGTAACTTGATTAAGGTCACACAGCCAG

                                      610       620       630       640       650       660       670       680       690       700
                                 ....|....|....|....|....|....|....|....|....|....|....|....|....|....|....|....|....|....|....|....|
Aotus_1602 [Anan_2.0]            ggccgggcgcggtggcttaagcctgtaatcccagcactttgggaggccgagacgggtggatcacgaggtcaagagatcgagaccatcctggtcaacatgg
JW-1602-86230-SIntR-rc           GGCCGGGCGCGGTGGCTTAAGCCTGTAATCCCAGCACTTTGGGAGGCCGAGACGGGTGGATCACGAGGTCAAGAGATCGAGACCATCCTGGTCAACATGG
JW-1602-86230-F                  GGCCGGGCGCGGTGGCTTAAGCCTGTAATCCCAGCACTTTGGGAGGCCGAGACGGGTGAATCACGAGGTCAAGAGATCGAGACCATCCTGGTCAACATGG
JW-1602-86230-IntF               ~~~~~~~~~~~~~~~~~~~~~~~~~~~~~~~~~~~~~~~~~~~~~~~~~~~~~~~~~~~~~~~~~~~~~~~~~~~~~~~~~~~~~~~~~~~~~~~~~~~~
JW-1602-85818-SIntR-rc           GGCCGGGCGCGGTGGCTTAAGCCTGTAATCCCAGCACTTTGGGAGGCCGAGACGGGTGGATCACGAGGTCAAGAGATCGAGACCATCCTGGTCAACATGG
JW-1602-85818-F                  GGCCGGGCGCGGTGGCTTAAGCCTGTAATCCCAGCACTTTGGGAGGCCGAGACGGGTGGATCACGAGGTCAAGAGATCGAGACCATCCTGGTCAACATGG
JW-1602-85818-IntF               ~~~~~~~~~~~~~~~~~~~~~~~~~~~~~~~~~~~~~~~~~~~~~~~~~~~~~~~~~~~~~~~~GAGGTCAAGAGATCGAGACCATCCTGGTCAACATGG
JW-1602-crl1556-E-F              ~~~~~~~~~~~~~~~~~~~~~~~~~~~~~~~~~~~~~~~~~~~~~~~~~~~~~~~~~~~~~~~~~~~~~~~~~~~~~~~~~~~~~~~~~~~~~~~~~~~~
JW-1602-crl1556-E-R-rc

                                      710       720       730       740       750       760       770       780       790       800
                                 ....|....|....|....|....|....|....|....|....|....|....|....|....|....|....|....|....|....|....|....|
Aotus_1602 [Anan_2.0]            tgaaaccccgtctttactaatacaaaaaattagctgggcatggtggcgcatgcctgtaatcccagctactcaggaggctgaggcaggagaattgcctgaa
JW-1602-86230-SIntR-rc           TGAAACCCCGTCTTTACTAATACAAAAAATTAGCTGGGCATGGTGGCGCGTGCCTGTAATCCCAGCTACTCAGGAGG
JW-1602-86230-F                  TGAAACCCCGTCTTTACTAATACAAAAAATTAGCTGGGCATGGTGGCGCGTGCCTGTAATCCCAGCTACTCAGGAGGCTGAGGCAGGAGAATTGCCTGAA
JW-1602-86230-IntF               ~~AAACCCCGTCTTTACTAATACAAAAAATTAGCTGGGCATGGTGGCGCGTGCCTGTAATCCCAGCTACTCAGGAGGCTGAGGCAGGAGAATTGCCTGAA
JW-1602-85818-SIntR-rc           TGAAACCCCGTCTTTACTAATACAAAAAATTAGCTGGGCATGGTGGCGCGTGCTTGTAATCCCAGCTACTCAGGAGG
JW-1602-85818-F                  TGAAACCCCGTCTTTACTAATACAAAAAATTAGCTGGGCGTGGTGGCGCGTGCTTGTAATCCCAGCTACTCAGGAGGCTGAGGCAGGAGAATTGCCTGAA
JW-1602-85818-IntF               TGAAACCCCGTCTTTACTAATACAAAAAATTAGCTGGGCGTGGTGGCGCGTGCTTGTAATCCCAGCTACTCAGGAGGCTGAGGCAGGAGAATTGCCTGAA
JW-1602-crl1556-E-F              ~~~~~~~~~~~~~~~~~~~~~~~~~~~~~~~~~~~~~~~~~~~~~~~~~~~~~~~~~~~~~~~~~~~~~~~~~~~~~~~~~~~~~~~~~~~~~~~~~~~~
JW-1602-crl1556-E-R-rc

                                      810       820       830       840       850       860       870       880       890       900
                                 ....|....|....|....|....|....|....|....|....|....|....|....|....|....|....|....|....|....|....|....|
Aotus_1602 [Anan_2.0]            cccaggaggcggaggttgcggtgagccgagatcgcgccattgcactccagcctgggtaacgagcgaaactccgtctcaaaaaaaaaaaaaaagtcacatg
JW-1602-86230-SIntR-rc
JW-1602-86230-F                  CCCAGGAGGCGGAGGTTGCGGTGAGCCGAGATCGCGCCATTGCACTCCAGCCTGGGTAACGAGCGAAACTCCGTCTCAAAAAAAAAAAAAAA
JW-1602-86230-IntF               CCCAGGAGGCGGAGGTTGCAGTGAGCCGAGATCGCGCCATTGCACTCCAGCCTGGGTAACGAGCGAAACTCCGTCTCAAAAAAAAAAAAAA
JW-1602-85818-SIntR-rc
JW-1602-85818-F                  CCCAGGAGGCGGAGGTTGCGGTGAGCCGAGATCGCGCCATTGCACTCCAGCCTGGGTAACGAACGAAACTCCGTCTCAAAAAAAAAAAAA
JW-1602-85818-IntF               CCCAGGAGGCGGAGGTTGCGGTGAGCCGAGATCGCGCCATTGCACTCCAGCCTGGGTAACGAACGAAACTCCGTCTCAAAAAAAAAAAAA
JW-1602-crl1556-E-F              ~~~~~~~~~~~~~~~~~~~~~~~~~~~~~~~~~~~~~~~~~~~~~~~~~~~~~~~~~~~~~~~~~~~~~~~~~~~~~~~~~~~~~~~~~~~~~~~~~~~~
JW-1602-crl1556-E-R-rc
```

```
                              910       920       930       940       950       960       970       980       990      1000
                     ....|....|....|....|....|....|....|....|....|....|....|....|....|....|....|....|....|....|....|....|
Aotus_1602 [Anan_2.0]       caCCTGACACCACATGCTTCTTATATGTCCCCTCAGGAACATGGACTAATCAGGGAAGACTCAATCTTATCATTGGTCCATTCACAGTTAATACTTTGAT
JW-1602-86230-SIntR-rc
JW-1602-86230-F
JW-1602-86230-IntF
JW-1602-85818-SIntR-rc
JW-1602-85818-F
JW-1602-85818-IntF
JW-1602-crl1556-E-F         ~ACCTGACACCACATGCTTCTTA
JW-1602-crl1556-E-R-rc
```

Figure S31. Sequence alignment for *Alu* locus *Aotus*_1602_018513626.1:10501295-10502786 (JW-1602). PCR based genotypes indicate the target *Alu* is homozygous present (1/1) in all *A. nancymaae*, *A. vociferans* and *A. azarae* individuals while homozygous absent (0/0) in all *A. trivirgatus* individuals and the *A. l. griseimembra* sample. The gel image is shown in the main text as Figure 2b. DNA sequencing was performed to confirm this pattern. The reference genome [Anan_2.0] is shown at the top followed by samples *A. vociferans* 86230 and *A. azarae* 85818 both of whom share the target *Alu* based on 5' flanking sequence and the *Alu* start position at 601 (grey highlight). Sequencing of the empty site amplicon in *A. trivirgatus* crl-1556 has a precise pre-integration site spanning the target region, confirming the genotypes. This *Alu* insertion/absence pattern supports *A. trivirgatus* as basal to *A. vociferans* rather than the other way around.

```
                               510       520       530       540       550       560       570       580       590       600
                          ....|....|....|....|....|....|....|....|....|....|....|....|....|....|....|....|....|....|....|....|
Storer_2020_Aotus-622     TCCTCCGCCCCCAGTGCTCCCTTCAGTTAAAGATTAACGTGATCCTGTTAGCGATGCTGTTTTGTGAATCCAGGGCAGTCAGCACTGCtgaaaagaaaag
JS-622-155159-SIntR-rc    TCCTCCGCCCCCAGTGCTCCCTCCAGTTAAAGATTAACGTGATCCTGTTAGCGATGCTGTTTTGTGAATCCAGGGCAGTCAGCACTGCTGAAAAGAAAAG
JS-622-155159-F           ~~~~~~~~~~~~~~~~~~CCCTCCAGTTAAAGATTAACGTGATCCTGTTAGCGATGCTGTTTTGTGAATCCAGGGCAGTCAGCACTGCTGAAAAGAAAAG
JS-622-155159-R-rc        ~~~~~~~~~~~~~~~~~~~~~~~~~~~~~~~~~~~~~~~~~~~~~~~~~~~~~~~~~~~~~~~~~~~~~~~~~~~~~~~~~~~~~~~~~~~~~~~~~~~~
JS-622-85818-SIntR-rc     TCCTCCGCCCCCAGTGCTCCCTTCAGTTAAAGATTAACGTGATCCTGTTAGCGATGCTGTTTTGTGAATCCAGGGCAGTCAGCACTGCTGAAAAGAAAAG
JS-622-85818-F            ~~~~~~~~~~~~~~~~~~~~~~~~~~TTAAAGATTAACGTGATCCTGTTAGCGATGCTGTTTTGTGAATCCAGGGCAGTCAGCACTGCTGAAAAGAAAAG
JS-622-85818-IntF         ~~~~~~~~~~~~~~~~~~~~~~~~~~~~~~~~~~~~~~~~~~~~~~~~~~~~~~~~~~~~~~~~~~~~~~~~~~~~~~~~~~~~~~~~~~~~~~~~~~~~
JS-622-85818-R-rc         ~~~~~~~~~~~~~~~~~~~~~~~~~~~~~~~~~~~~~~~~~~~~~~~~~~~~~~~~~~~~~~~~~~~~~~~~~~~~~~~~~~~~~~~~~~~~~~~~~~~~
JS-622-86230-E-F          ~~~~~~~~~~~~~~~~~~~~~~~~~~~~~~~~~~~AACGTGATCCTGTTAGCGATGCTGTTTTGTGAATCCAGGGCAGTCAGCACTGCTGAAAAGAAAAG
JS-622-86230-E-R-rc       TCCTCCGCCCCCAGTGCTCCCTCCAGTTAAAGATTAACGTGATCCTGTTAGCGATGCTGTTTTGTGAATCCAGGGCAGTCAGCACTGCTGAAAAGAAAAG
JS-622-crl1556-E-F        ~~~~~~~~~~~~~~~~~~~~~~~~~~~~~~~~~~~AACGTGATCCTGTTAGCGATGCTGTTTTGTGAATCCAGGGCAGTCAGCACTGCTGAAAAGAAAAG
JS-622-crl1556-E-R-rc     TCCTCCGCCCCCAGTGCTCCCTCCAGTTAAAGATTAACGTGATCCTGTTAGCGATGCTGTTTTGTGAATCCAGGGCAGTCAGCACTGCTGAAAAGAAAAG

                               610       620       630       640       650       660       670       680       690       700
                          ....|....|....|....|....|....|....|....|....|....|....|....|....|....|....|....|....|....|....|
Storer_2020_Aotus-622     agcatttcgccgggcgcggtggctcacgcctgtaatcccaggactttgggaggccgaggcgggtggatcatgaggtcaagagatcaagaccatcctggtc
JS-622-155159-SIntR-rc    AGCATTTCGCCGGGCGCGGTGGCTCACGCCTGTAATCCCAGCACTTTGGGAGGCCGAGGCGGGTGGATCATGAGATCAAGAGATCGAGACCATCCTGGTC
JS-622-155159-F           NGCATTTCGCCGGGCGCGGTGGCTCACGCCTGTAATCCCAACACTTTGGGAGGCCGAGGCGG
JS-622-155159-R-rc        ~~~~~~~~~~~~~~~~~~~~~~~~~~~~~~~~~~~~~~~~~~~~~~~~~~~~~~~~~~~~~~~~~~~~~~~~~~~~~~~~~~~~~~~~~~~~~~~~~~~~
JS-622-85818-SIntR-rc     AGCATTTCGCCGGGCGCGGTGGCTCACGCCTGTAATCCCAGGACTTTGGGAGGCCGAAGCGGGTGGATCATGAGGTCAAGAGATCAAGACCATCCTGGTC
JS-622-85818-F            AGCATTTCGCCGGGCGCGGTGGCTCACGCCTGTAATCCCAGGACTTTGGGAGGCCGAAGCGGGTGGATCATGAGGTCAAGAGATCAAGACCATCCTGGTC
JS-622-85818-IntF         ~~~~~~~~~~~~~~~~~~~~~~~~~~~~~~~~~~~~~~~~~~~~~~~~~~~~~~~~~~~~~~~~~~~~~~~GAGGTCAAGAGATCAAGACCATCCTGGTC
JS-622-85818-R-rc         ~~~~~~~~~~~~~~~~~~~~~~~~~~~~~~~~~~~~~~~~~~~~~~~~~~~~~~~~~~~~~~~~~~~~~~~~~~~~~~~~~~~~~~~~~~~~~~~~~~~~
JS-622-86230-E-F          AGCATTT~~~~~~~~~~~~~~~~~~~~~~~~~~~~~~~~~~~~~~~~~~~~~~~~~~~~~~~~~~~~~~~~~~~~~~~~~~~~~~~~~~~~~~~~~~~~~
JS-622-86230-E-R-rc       AGCATTT~~~~~~~~~~~~~~~~~~~~~~~~~~~~~~~~~~~~~~~~~~~~~~~~~~~~~~~~~~~~~~~~~~~~~~~~~~~~~~~~~~~~~~~~~~~~~
JS-622-crl1556-E-F        AGCATTT~~~~~~~~~~~~~~~~~~~~~~~~~~~~~~~~~~~~~~~~~~~~~~~~~~~~~~~~~~~~~~~~~~~~~~~~~~~~~~~~~~~~~~~~~~~~~
JS-622-crl1556-E-R-rc     AGCATTT~~~~~~~~~~~~~~~~~~~~~~~~~~~~~~~~~~~~~~~~~~~~~~~~~~~~~~~~~~~~~~~~~~~~~~~~~~~~~~~~~~~~~~~~~~~~~

                               710       720       730       740       750       760       770       780       790       800
                          ....|....|....|....|....|....|....|....|....|....|....|....|....|....|....|....|....|....|....|
Storer_2020_Aotus-622     aacatggtgaaaccccgtctctactataaatacaaaaaattagctgggcatggtggcacgtgcctgtaatcccagctactcaggaggctgaggcaggaga
JS-622-155159-SIntR-rc    AACATGGTGAAACCCCGTCTCTACTATAAATACAAAAAANNTAGCTGGGCATGGTGGCACGTGCCTGTAATCCCAGCTACTCAGGAGG
JS-622-155159-F
JS-622-155159-R-rc        ~~~~~~~~~~~~~~~~~~~~~~~~~~~~~~~~~~~~~~~~~~~~~~~~~~~~~~~~~~~~~~~~~~~~~~~~~~~~~~~~~~~~~~~~~~~~~~~~~~~~
JS-622-85818-SIntR-rc     AACATGGTGAAACCCCGTCCCTACTATAAATACAAAAAATTAGCTGGGCATGGTGGCACGTGCCTGTAATCCCAGCTACTCAGGAGG
JS-622-85818-F            AACATGGTGAAACCCCGTCCCTACTATAAATACAAAAAATTAGCTGGGCATGGTGGCACGTGCCTGTAATCCCAGCTACTCAGGAGGCTGAGGCAGGAGA
JS-622-85818-IntF         AACATGGTGAAACCCCGTCCCTACTATAAATACAAAAAATTAGCTGGGCATGGTGGCACGTGCCTGTAATCCCAGCTACTCAGGAGGCTGAGGCAGGAGA
JS-622-85818-R-rc         ~~~~~~~~~~~~~~~~~~~~~~~~~~~~~~~~~~~~~~~~~~~~~~~~~~~~~~~~~~~~~~~~~~~~~~~~~~~~~~~~~~~~~~~~~~~~~~~~~~~~
JS-622-86230-E-F          ~~~~~~~~~~~~~~~~~~~~~~~~~~~~~~~~~~~~~~~~~~~~~~~~~~~~~~~~~~~~~~~~~~~~~~~~~~~~~~~~~~~~~~~~~~~~~~~~~~~~
JS-622-86230-E-R-rc       ~~~~~~~~~~~~~~~~~~~~~~~~~~~~~~~~~~~~~~~~~~~~~~~~~~~~~~~~~~~~~~~~~~~~~~~~~~~~~~~~~~~~~~~~~~~~~~~~~~~~
JS-622-crl1556-E-F        ~~~~~~~~~~~~~~~~~~~~~~~~~~~~~~~~~~~~~~~~~~~~~~~~~~~~~~~~~~~~~~~~~~~~~~~~~~~~~~~~~~~~~~~~~~~~~~~~~~~~
JS-622-crl1556-E-R-rc     ~~~~~~~~~~~~~~~~~~~~~~~~~~~~~~~~~~~~~~~~~~~~~~~~~~~~~~~~~~~~~~~~~~~~~~~~~~~~~~~~~~~~~~~~~~~~~~~~~~~~
```

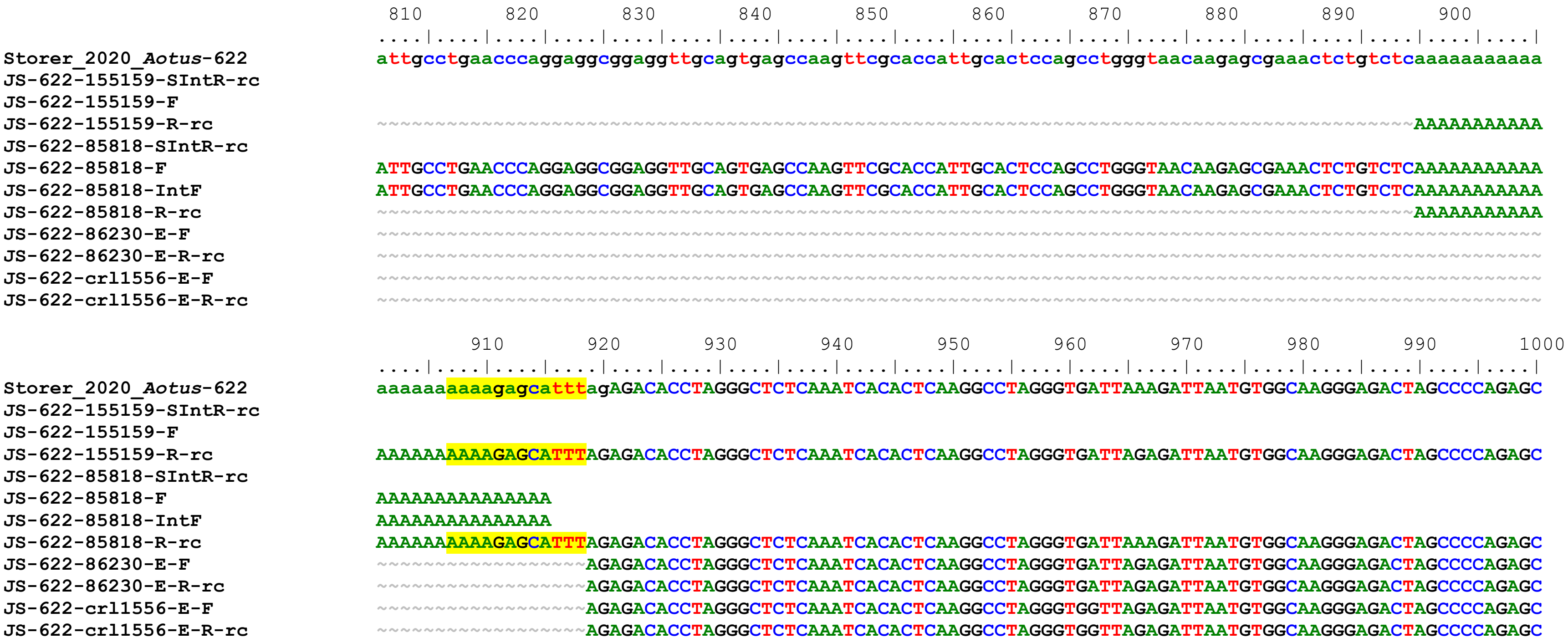

Figure S32. Sequence alignment for *Alu* locus: Storer, et al. 2020: *Aotus*_622_018491217_447777 (JS-622). PCR-based genotypes indicate that all *A. nancymaae* and *A. azarae* individuals are homozygous present (1/1) for the target *Alu* insertion, while *A. vociferans* samples have evidence of polymorphism and all four *A. trivirgatus* samples are homozygous absent (0/0) for the insertion. DNA sequencing was performed to confirm this pattern. The reference genome [Anan_2.0] is shown at the top followed by samples *A. vociferans* MVZ-155159 and *A. azarae* 85818, both of whom share the target *Alu* insertion starting at position 608 (grey highlight). The TSDs are shown in yellow highlight. Empty site amplicons for *A. vociferans* 86230 and *A. trivirgatus* crl-1556 both display a precise pre-integration site with both forward and reverse sequences spanning the target region, confirming the genotypes. This insertion pattern suggests that *A. trivirgatus* is most basal among the species on our panel, followed by other grey-neck species, while *A. nancymaae* and *A. azarae* are closely related.

```
                                510       520       530       540       550       560       570       580       590       600
                           ....|....|....|....|....|....|....|....|....|....|....|....|....|....|....|....|....|....|....|....|
Owl_2LS_018_[Anan_2.0]     TGGAGGGAGTGGTGGCAAAGCTTAAGCAGACAGAGTTCTAGAGAGAAGGGGGAAACCTGAAAGCAGCAAGTAGGAATAACTTGTAGAAGAGTCTgcatga
LR-18-crl1556-F            ~~~~~~~~~~~~~~~~~~~~~~~~~~~~~~~AGAGTTCTAGAGAGAAGGGGGAAACCTGAAAGCAGCAAGTAGGAATAACTTGTAGAAGAGTCTGCATGA
LR-18-crl1556-SIntR-rc     TGGAGGGAGTGGTGGCAAAGCTTAAGCAGACAGAGTTCTAGAGAGAAGGGGGAAACCTGAAAGCAGCAAGTAGGAATAACTTGTAGAAGAGTCTGCATGA
LR-18-crl1556-IntF         ~~~~~~~~~~~~~~~~~~~~~~~~~~~~~~~~~~~~~~~~~~~~~~~~~~~~~~~~~~~~~~~~~~~~~~~~~~~~~~~~~~~~~~~~~~~~~~~~~~~~
LR-18-crl1556-R-rc         ~~~~~~~~~~~~~~~~~~~~~~~~~~~~~~~~~~~~~~~~~~~~~~~~~~~~~~~~~~~~~~~~~~~~~~~~~~~~~~~~~~~~~~~~~~~~~~~~~~~~
LR-18-86230-F              ~~~~~~~~~~~~~~~~~~~~~~~~~~~~~~~~~~~~~~~~~~~~~~~GGGGGAAACCTGAAAGCAGCAAGTAGGAATAACTTGTAGAAGAGTCTGCATGA
LR-18-86230-IntF           ~~~~~~~~~~~~~~~~~~~~~~~~~~~~~~~~~~~~~~~~~~~~~~~~~~~~~~~~~~~~~~~~~~~~~~~~~~~~~~~~~~~~~~~~~~~~~~~~~~~~
LR-18-86230-SIntR-rc       TGGAGGGAGTGGTGGCAAAGCTTAAGCAGACAGAGTTCTAGAGAGAAGGGGGAAACCTGAAAGCAGCAAGTAGGAATAACTTGTAGAAGAGTCTGCATGA
LR-18-86230-R-rc           ~~~~~~~~~~~~~~~~~~~~~~~~~~~~~~~~~~~~~~~~~~~~~~~~~~~~~~~~~~~~~~~~~~~~~~~~~~~~~~~~~~~~~~~~~~~~~~~~~~~~
LR-18-KB4883-F             ~~~~~~~~~~~~~~~~~~~~~~~~~~~~~~~~GAGTTCTAGAGAGAAGGGGGAAACCTGAAAGCAGCAAGTAGGAATAACTTGNAGAAGAGTCTGCATGA
LR-18-KB4883-IntF          ~~~~~~~~~~~~~~~~~~~~~~~~~~~~~~~~~~~~~~~~~~~~~~~~~~~~~~~~~~~~~~~~~~~~~~~~~~~~~~~~~~~~~~~~~~~~~~~~~~~~
LR-18-KB4883-SIntR-rc      TGGAGGGAGTGGTGGCAAAGCTTAAGCAGACAGAGTTCTAGAGAGAAGGGGGAAACCTGAAAGCAGCAAGTAGGAATAACTTGTAGAAGAGTCTGCATGA
LR-18-KB4883-R-rc          ~~~~~~~~~~~~~~~~~~~~~~~~~~~~~~~~~~~~~~~~~~~~~~~~~~~~~~~~~~~~~~~~~~~~~~~~~~~~~~~~~~~~~~~~~~~~~~~~~~~~
LR-18-85464-E-F            ~~~~~~~~~~~~~~~~~~~~~~~~~~~~~~~~GAGTTCTAGAGAGAAGGGGGAAACCTGAAAGCAGCAAGTAGGAATAACTTGTAGAAGAGTCTGCATGA
LR-18-85464-E-R-rc         TGGAGGGAGTGGTGGCAAAGCTTAAGCAGACAGAGTTCTAGAGAGAAGGGGGAAACCTGAAAGCAGCAAGTAGGAATAACTTGTAGAAGAGTCTGCATGA
LR-18-85818-E-F            ~~~~~~~~~~~~~~~~~~~~~~~~~~~~~~~~~~~TTCTAGAGAGAAGGGGGAAACCTGAAAGCAGCAAGTAGGAATAACTTGTAGAAGGGTCTGCATGA
LR-18-85818-E-R-rc         TGGAGGGAGTGGTGGCAAAGCTTAAGCAGACAGAGTTCTAGAGAGAAGGGGGAAACCTGAAAGCAGCAAGTAGGAATAACTTGTAGAAGGGTCTGCATGA
LR-18-86100-E-F            ~~~~~~~~~~~~~~~~~~~~~~~~~~~~~~~~~~~TTCTAGAGAGAAGGGGGAAACCTGAAAGCAGCAAGTAGGAATAACTTGTAGAAGANTCTGCATGA
LR-18-86100-E-R-rc         TGGAGGGAGTGGTGGCAAAGCTTAAGCAGACAGAGTTCTAGAGAGAAGGGGGAAACCTGAAAGCAGCAAGTAGGAATAACTTGTAGAAGAGTCTGCATGA

                                610       620       630       640       650       660       670       680       690       700
                           ....|....|....|....|....|....|....|....|....|....|....|....|....|....|....|....|....|....|....|
Owl_2LS_018_[Anan_2.0]     ggccgggcgcggtggctcaagcctgtaatcccagcactttgggaggccgaggcgggtggatcacgaggtgaacagatcgagaccatcctggtcaacatgg
LR-18-crl1556-F            GGCCGGGCGCGGTGGCTCAAGCCTGTAATCCCAGCACTTTGGGAGGCTGAGGCGGGTGGATCACGAAGTGAACAGATCGAGACCATCCTGGTCAACATGG
LR-18-crl1556-SIntR-rc     GGCCGGGCGCGGTGGCTCAAGCCTGTAATCCCAGCACTTTGGGAGGCTGAGGCGGGTGGATCACGAAGTGAACAGATCGAGACCATCCTGGTCAACATGG
LR-18-crl1556-IntF         ~~~~~~~~~~~~~~~~~~~~~~~~~~~~~~~~~~~~~~~~~~~~~~~~~~~~~~~~~~~~~~~~~~~~~~~~~~~~~~GAGACCATCCTGGTCAACATGG
LR-18-crl1556-R-rc         ~~~~~~~~~~~~~~~~~~~~~~~~~~~~~~~~~~~~~~~~~~~~~~~~~~~~~~~~~~~~~~~~~~~~~~~~~~~~~~~~~~~~~~~~~~~~~~~~~~~~
LR-18-86230-F              GGCCGGGCGCGGTGGCTCAAGCCTGTAATCCCAGCACTTTGGGAGGCCGAGGCGGGTGGATCACGAGGTGAACAGATCGAGACCATCCTGGTCAACATGG
LR-18-86230-IntF           ~~~~~~~~~~~~~~~~~~~~~~~~~~~~~~~~~~~~~~~~~~~~~~~~~~~~~~~~~~~~~~~~GAGGTGAACAGATCGAGACCATCCTGGTCAACATGG
LR-18-86230-SIntR-rc       GGCCGGGCGCGGTGGCTCAAGCCTGTAATCCCAGCACTTTGGGAGGCCGAGGCGGGTGGATCACGAGGTGAACAGATCGAGACCATCCTGGTCAACATGG
LR-18-86230-R-rc           ~~~~~~~~~~~~~~~~~~~~~~~~~~~~~~~~~~~~~~~~~~~~~~~~~~~~~~~~~~~~~~~~~~~~~~~~~~~~~~~~~~~~~~~~~~~~~~~~~~~~
LR-18-KB4883-F             GGCCGGGCGCGGTGGCTCAAGCCTGTAATCCCAGCACTTTGGGAGGCTGAGGCGGGTGGATCACGAGGTGAACAGATCGAGACCATCCTGATCAACATGG
LR-18-KB4883-IntF          ~~~~~~~~~~~~~~~~~~~~~~~~~~~~~~~~~~~~~~~~~~~~~~~~~~~~~~~~~~~~~~~~~~~~~~~~CAGATCGAGACCATCCTGATCAACATGG
LR-18-KB4883-SIntR-rc      GGCCGGGCGCGGTGGCTCAAGCCTGTAATCCCAGCACTTTGGGAGGCTGAGGCGGGTGGATCACGAGGTGAACAGATCGAGACCATCCTGATCAACATGG
LR-18-KB4883-R-rc          ~~~~~~~~~~~~~~~~~~~~~~~~~~~~~~~~~~~~~~~~~~~~~~~~~~~~~~~~~~~~~~~~~~~~~~~~~~~~~~~~~~~~~~~~~~~~~~~~~~~~
LR-18-85464-E-F            ~~~~~~~~~~~~~~~~~~~~~~~~~~~~~~~~~~~~~~~~~~~~~~~~~~~~~~~~~~~~~~~~~~~~~~~~~~~~~~~~~~~~~~~~~~~~~~~~~~~~
LR-18-85464-E-R-rc         ~~~~~~~~~~~~~~~~~~~~~~~~~~~~~~~~~~~~~~~~~~~~~~~~~~~~~~~~~~~~~~~~~~~~~~~~~~~~~~~~~~~~~~~~~~~~~~~~~~~~
LR-18-85818-E-F            ~~~~~~~~~~~~~~~~~~~~~~~~~~~~~~~~~~~~~~~~~~~~~~~~~~~~~~~~~~~~~~~~~~~~~~~~~~~~~~~~~~~~~~~~~~~~~~~~~~~~
LR-18-85818-E-R-rc         ~~~~~~~~~~~~~~~~~~~~~~~~~~~~~~~~~~~~~~~~~~~~~~~~~~~~~~~~~~~~~~~~~~~~~~~~~~~~~~~~~~~~~~~~~~~~~~~~~~~~
LR-18-86100-E-F            ~~~~~~~~~~~~~~~~~~~~~~~~~~~~~~~~~~~~~~~~~~~~~~~~~~~~~~~~~~~~~~~~~~~~~~~~~~~~~~~~~~~~~~~~~~~~~~~~~~~~
LR-18-86100-E-R-rc         ~~~~~~~~~~~~~~~~~~~~~~~~~~~~~~~~~~~~~~~~~~~~~~~~~~~~~~~~~~~~~~~~~~~~~~~~~~~~~~~~~~~~~~~~~~~~~~~~~~~~

                                710       720       730       740       750       760       770       780       790       800
                           ....|....|....|....|....|....|....|....|....|....|....|....|....|....|....|....|....|....|....|
Owl_2LS_018_[Anan_2.0]     tgaaaccccgtctctactaatacaaaaacttagctgggcatggtggcgcgtgcctgtaatcccagctactcaggaggctgaggcagggaattgcctgaac
LR-18-crl1556-F            TGAAACCCCGTCTCTACTAATACAAAAAATTAGCTGGGCATGGTGGCGCGTGACTGTAATCCCAGCTACTCAGGAGGCTGAGGCAGGGAATTGCCTGAAC
LR-18-crl1556-SIntR-rc     TGAAACCCCGTCTCTACTAATACAAAAAATTAGCTGGGCATGGTGGCGCGTGACTGTAATCCCAGCTACTCAGG
LR-18-crl1556-IntF         TGAAACCCCGTCTCTACTAATACAAAAAATTAGCTGGGCATGGTGGCGCGTGACTGTAATCCCAGCTACTCAGGAGGCTGAGGCAGGGAATTGCCTGAAC
LR-18-crl1556-R-rc         ~~~~~~~~~~~~~~~~~~~~~~~~~~~~~~~~~~~~~~~~~~~~~~~~~~~~~~~~~~~~~~~~~~~~~~~~~~~~~~~~~~~~~~~~~~~~~~~~~~~~
```

```
LR-18-86230-F            TGAAACCCCGTCTCTACTAATACAAAAAATTAGCTGGGCATGGTGGCGCGTGCCTGTAATCCCAGCTACTCAGGAGGCTGAGGCAGGGAATTGCCTGAAC
LR-18-86230-IntF         TGAAACCCCGTCTCTACTAATACAAAAAATTAGCTGGGCATGGTGGCGCGTGCCTGTAATCCCAGCTACTCAGGAGGCTGAGGCAGGGAATTGCCTGAAC
LR-18-86230-SIntR-rc     TGAAACCCCGTCTCTACTAATACAAAAAATTAGCTGGGCATGGTGGCGCGTGCCTGTAATCCCAGCTACTCAGGAGG
LR-18-86230-R-rc         ~~~~~~~~~~~~~~~~~~~~~~~~~~~~~~~~~~~~~~~~~~~~~~~~~~~~~~~~~~~~~~~~~~~~~~~~~~~~~~~~~~~~~~~~~~~~~~~~~~~~
LR-18-KB4883-F           TGAAACCCCGTCTCTACTAATACAAAAAATTAGCTGGGCATGGTGGCGCGTGACTGTAATCCCAGCTACTCAGGAGGCTGAGGCAGGGAATTGCCTGAAC
LR-18-KB4883-IntF        TGAAACCCCGTCTCTACTAATACAAAAAATTAGCTGGGCATGGTGGCGCGTGACTGTAATCCCAGCTACTCAGGAGGCTGAGGCAGGGAATTGCCTGAAC
LR-18-KB4883-SIntR-rc    TGAAACCCCGTCTCTACTAATACAAAAAATTAGCTGGGCATGGTGGCGCGTGACTGTAATCCCAGCTACTCAGGAGG
LR-18-KB4883-R-rc        ~~~~~~~~~~~~~~~~~~~~~~~~~~~~~~~~~~~~~~~~~~~~~~~~~~~~~~~~~~~~~~~~~~~~~~~~~~~~~~~~~~~~~~~~~~~~~~~~~~~~
LR-18-85464-E-F          ~~~~~~~~~~~~~~~~~~~~~~~~~~~~~~~~~~~~~~~~~~~~~~~~~~~~~~~~~~~~~~~~~~~~~~~~~~~~~~~~~~~~~~~~~~~~~~~~~~~~
LR-18-85464-E-R-rc       ~~~~~~~~~~~~~~~~~~~~~~~~~~~~~~~~~~~~~~~~~~~~~~~~~~~~~~~~~~~~~~~~~~~~~~~~~~~~~~~~~~~~~~~~~~~~~~~~~~~~
LR-18-85818-E-F          ~~~~~~~~~~~~~~~~~~~~~~~~~~~~~~~~~~~~~~~~~~~~~~~~~~~~~~~~~~~~~~~~~~~~~~~~~~~~~~~~~~~~~~~~~~~~~~~~~~~~
LR-18-85818-E-R-rc       ~~~~~~~~~~~~~~~~~~~~~~~~~~~~~~~~~~~~~~~~~~~~~~~~~~~~~~~~~~~~~~~~~~~~~~~~~~~~~~~~~~~~~~~~~~~~~~~~~~~~
LR-18-86100-E-F          ~~~~~~~~~~~~~~~~~~~~~~~~~~~~~~~~~~~~~~~~~~~~~~~~~~~~~~~~~~~~~~~~~~~~~~~~~~~~~~~~~~~~~~~~~~~~~~~~~~~~
LR-18-86100-E-R-rc       ~~~~~~~~~~~~~~~~~~~~~~~~~~~~~~~~~~~~~~~~~~~~~~~~~~~~~~~~~~~~~~~~~~~~~~~~~~~~~~~~~~~~~~~~~~~~~~~~~~~~

                                  810       820       830       840       850       860       870       880       890       900
                         ....|....|....|....|....|....|....|....|....|....|....|....|....|....|....|....|....|....|....|....|
Owl_2LS_018_[Anan_2.0]   ccaggaggcggaagttgcggtgagccgagatcgcgccattgcactccagcctgggtaacgagcgaaactccgtctcaaaaaaaaaaaaaaaaaaaagagt
LR-18-crl1556-F          CCAGGAGGCGGAAGTTGCGGTGAGCCAAGATCGCGCCATTGCACTCCAGCCTGGGTAACGAGCGAAACTCCGTCCCAAAAAAAAAAAAAAA
LR-18-crl1556-SIntR-rc
LR-18-crl1556-IntF       CCAGGAGGCGGAAGTTGCGGTGAGCCGAGATCGCGCCATTGCACTCCAGCCTGGGTAACGAGCGAAACTCCGTCCCAAAAAAAAAAAAAAAAA
LR-18-crl1556-R-rc       ~~~~~~~~~~~~~~~~~~~~~~~~~~~~~~~~~~~~~~~~~~~~~~~~~~~~~~~~~~~~~~~~~~~~~~~~~~~~~~~~~~AAAAAAAAAAAAAAGAGT
LR-18-86230-F            CCAGGAGGCGGAAGTTGCGGTGAGCCGAGATCGCGCCATTGCACTCCAGCCTGGGTAACAAGGGAAACTCCGTCTCAAAAAAAAAAAAAAAAAAAA
LR-18-86230-IntF         CCAGGAGGCGGAAGTTGCGGTGAGCCGAGATCGCGCCATTGCACTCCAGCCTGGGTAACAAGGGAAACTCCGTCTCAAAAAAAAAAAAAAAAAA
LR-18-86230-SIntR-rc
LR-18-86230-R-rc         ~~~~~~~~~~~~~~~~~~~~~~~~~~~~~~~~~~~~~~~~~~~~~~~~~~~~~~~~~~~~~~~~~~~~~~~~~~~~~~~~AAAAAAAAAAAAAAAAGAGT
LR-18-KB4883-F           CCAGGAGGCGGAAGTTGCGGTGAGCCGAGATCACGCCATTGCACTCCAGCCTGGGTAACGAGCGAAACTCCGTCCCAAAAAAAAAAAAA
LR-18-KB4883-IntF        CCAGGAGGCGGAAGTTGCGGTGAGCCGAGATCACGCCATTGCACTCCAGCCTGGGTAACGAGCGAAACTCCGTCCCAAAAAAAAAAAAAA
LR-18-KB4883-SIntR-rc
LR-18-KB4883-R-rc        ~~~~~~~~~~~~~~~~~~~~~~~~~~~~~~~~~~~~~~~~~~~~~~~~~~~~~~~~~~~~~~~~~~~~~~~~~~~~~~~~~~~~AAAAAAAAAAAAGAGT
LR-18-85464-E-F          ~~~~~~~~~~~~~~~~~~~~~~~~~~~~~~~~~~~~~~~~~~~~~~~~~~~~~~~~~~~~~~~~~~~~~~~~~~~~~~~~~~~~~~~~~~~~~~~~~~~~
LR-18-85464-E-R-rc       ~~~~~~~~~~~~~~~~~~~~~~~~~~~~~~~~~~~~~~~~~~~~~~~~~~~~~~~~~~~~~~~~~~~~~~~~~~~~~~~~~~~~~~~~~~~~~~~~~~~~
LR-18-85818-E-F          ~~~~~~~~~~~~~~~~~~~~~~~~~~~~~~~~~~~~~~~~~~~~~~~~~~~~~~~~~~~~~~~~~~~~~~~~~~~~~~~~~~~~~~~~~~~~~~~~~~~~
LR-18-85818-E-R-rc       ~~~~~~~~~~~~~~~~~~~~~~~~~~~~~~~~~~~~~~~~~~~~~~~~~~~~~~~~~~~~~~~~~~~~~~~~~~~~~~~~~~~~~~~~~~~~~~~~~~~~
LR-18-86100-E-F          ~~~~~~~~~~~~~~~~~~~~~~~~~~~~~~~~~~~~~~~~~~~~~~~~~~~~~~~~~~~~~~~~~~~~~~~~~~~~~~~~~~~~~~~~~~~~~~~~~~~~
LR-18-86100-E-R-rc       ~~~~~~~~~~~~~~~~~~~~~~~~~~~~~~~~~~~~~~~~~~~~~~~~~~~~~~~~~~~~~~~~~~~~~~~~~~~~~~~~~~~~~~~~~~~~~~~~~~~~

                                  910       920       930       940       950       960       970       980       990      1000
                         ....|....|....|....|....|....|....|....|....|....|....|....|....|....|....|....|....|....|....|....|
Owl_2LS_018_[Anan_2.0]   ctgcatgaaaaggaggtaaaaaaaaaggagaggattGGGGTCAcaagagttgtttttgtttttagatggcaGATAGTAGCTTGTTATATGCTGATGGGA
LR-18-crl1556-F
LR-18-crl1556-SIntR-rc
LR-18-crl1556-IntF
LR-18-crl1556-R-rc       CTGCATGAAAAGGAGGTAAA~GAAAAGGAGGGGATTAGGGTC
LR-18-86230-F
LR-18-86230-IntF
LR-18-86230-SIntR-rc
LR-18-86230-R-rc         CTGCATGAAAAGGAGATAAA~GAAAAGGAGGGGATTGGGGTCACAAGA
LR-18-KB4883-F
LR-18-KB4883-IntF
LR-18-KB4883-SIntR-rc
```

```
LR-18-KB4883-R-rc      NTGCANGAAAAGGAGGTAAAGAAAAGGAGGGGATTGGGGTCACAAGAG
LR-18-85464-E-F        ~~~~~~~~AAAGGAGGTCAAGAAAAGGAGGGGATTGGGGTCACAAGAGTTGTTTTTGTTTTCAGATGGCAGATAATAGCTTGTTATATGCTGATGGGAAG
LR-18-85464-E-R-rc     ~~~~~~~~AAAGGAGGTCAAGAAAAGGAGGGGATTGGGGTCACAAGAGTTGT
LR-18-85818-E-F        ~~~~~~~~AAAGGAGGTCAAGAAAAGGAGGGGATTGGGGTCACAAGAGTTGTTTTTGTTTTCAGATGGCAGATAATAGCTTGTTATATGCTGATGGGAAG
LR-18-85818-E-R-rc     ~~~~~~~~AAAGGAGGTCAAGAAAAGGAGGGGATTGGGGTCACAAGA
LR-18-86100-E-F        ~~~~~~~~AAAGGAGGTAAAGAAAAGGAGGGGATTGGGGTCACAAGAGTTGTTTTTGTTTTCAGATGGCAGATAATAGCTTGTGATATGCTGATGGGAAG
LR-18-86100-E-R-rc     ~~~~~~~~AAAGGAGGTAAAGAAAAGGAGGGGA
```

Figure S33. Sequence alignment for *Alu* locus Owl_2LS_018_018514491.1:3469855-3471351 (LR-18). PCR analyses produced a strange pattern of insertion presence/absence in which the target *Alu* is polymorphic among *A. nancymaae* and *A. vociferans*; homozygous present in all four *A. trivirgatus* individuals and the *A. l. griseimembra* sample KB4883 (1/1), while homozygous absent (0/0) in all three *A. azarae* samples. DNA sequencing was performed to confirm these genotypes. The reference genome [Anan_2.0] is shown at the top followed by samples *A. trivirgatus* crl-1556, *A. vociferans* 86230 and *A. l. griseimembra* sample KB4883, all of whom share the target *Alu* insertion starting at position 601 (grey highlight). The TSDs are highlighted in yellow. Sequence of empty site amplicons in *A. nancymaae* 85464, *A. azarae* 85818 and *A. vociferans* 86100 all reveal precise pre-integration sites with both forward and reverse sequences spanning the target region, confirming the genotypes. This pattern suggests extensive incomplete lineage sorting (ILS) of the target *Alu* insertion. **NOTE: A. voc. 86230 (with *Alu*) and 86100 (*Alu* absent) are siblings, same Dam and Sire.**

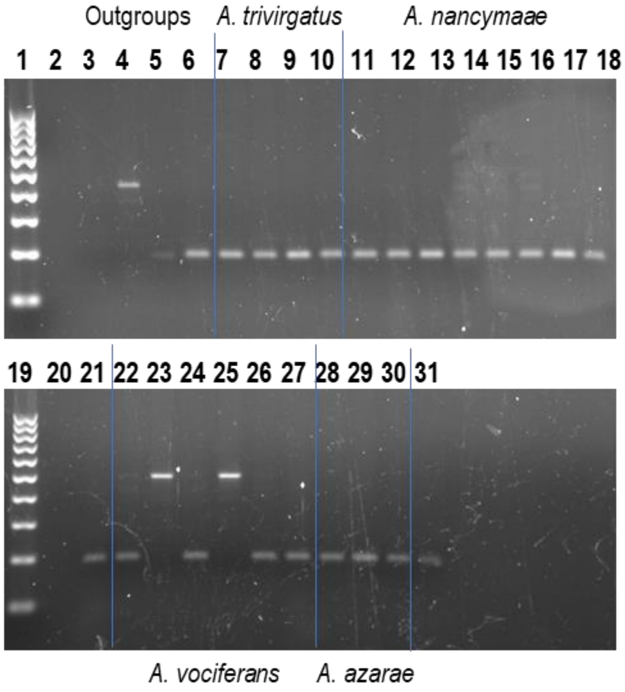

Figure S34a: *Alu* locus *Aotus*_1582_018497893.1:1531600-1533091 (JW-1582): Lanes: 1, 19- 100 bp DNA ladder, 2, 20-blank, 3-TLE (negative control), 4- Human (HeLa-has a different *Alu* in this region), 5- *Callithrix jacchus* (Common marmoset), 6- *Saimiri s. sciureus* (Common squirrel monkey), 7-10 *A. trivirgatus*, 11-18, 21 *A. nancymaae*, 22-27 *A. vociferans*, 28-30 *A. azarae*, 31-*A. lemurinus griseimembra.* Vertical blue lines superimposed on the gel image visually separate the species groups. The predicted amplicon sizes are 483/192 bp. PCR-based genotypes indicate that all *Aotus* individuals are homozygous absent (~192 bp fragment) except for these two *A. vociferans* individuals who have a homozygous present (~483 bp fragment) shown in lanes 23 and 25. DNA sequencing was performed to determine if *A. vociferans* MVZ-155159 and 86218 share the target *Alu* or perhaps have a near parallel insertion unique to *A. vociferans*. See alignment Figure S34b.

```
                                      510       520       530       540       550       560       570       580       590       600
                             ....|....|....|....|....|....|....|....|....|....|....|....|....|....|....|....|....|....|....|....|
Aotus-1582-rc_[Anan_2.0]     GAGTCAGCCATTTCTTTAAGAAGCCTTGGTTTGTTTGGTTGGAGAATGGTCTTTAGAAGCCAAAGAAGGGATGGGATTAAGCACTTCcccaaagtgaaaa
JW-1582-155159-SIntR-rc      GAGTCAGCCATTTCTTTAAGAAGCCTTGGTTTGTTTGGTTGGAGAATGGTCTTTAGAAGCCAAAGAAGGGATGGGATTAAGCACTTCCCCAAACTGAAAA
JW-1582-155159-R             ~~~~~~~~~~~~~~~~~~~~~~~~~~~~~~~~GTTTGGTTGGAGAATGGTCTTTAGAAGCCAAAGAAGGGATGGGATTAAGCACTTCCCCAAACTGAAAA
JW-1582-155159-IntF1         ~~~~~~~~~~~~~~~~~~~~~~~~~~~~~~~~~~~~~~~~~~~~~~~~~~~~~~~~~~~~~~~~~~~~~~~~~~~~~~~~~~~~~~~~~~~~~~~~~~~~
JW-1582-155159-F-rc          ~~~~~~~~~~~~~~~~~~~~~~~~~~~~~~~~~~~~~~~~~~~~~~~~~~~~~~~~~~~~~~~~~~~~~~~~~~~~~~~~~~~~~~~~~~~~~~~~~~~~
JW-1582-86218-SIntR-rc       GAGTCAGCCATTTCTTTAAGAAGCCTTGGTTTGTTTGGTTGGAGAATGGTCTTTAGAAGCCAAAGAAGGGATGGGATTAAGCACTTCCCCAAACTGAAAA
JW-1582-86218-R              ~~~~~~~~~~~~~~~~~~~~~~~~~~~~~TTTGTTTGGTTGGAGAATGGTCTTTAGAAGCCAAAGAAGGGATGGGATTAAGCACTTCCCCAAACTGAAAA
JW-1582-86218-IntF           ~~~~~~~~~~~~~~~~~~~~~~~~~~~~~~~~~~~~~~~~~~~~~~~~~~~~~~~~~~~~~~~~~~~~~~~~~~~~~~~~~~~~~~~~~~~~~~~~~~~~
JW-1582-86218-F-rc           ~~~~~~~~~~~~~~~~~~~~~~~~~~~~~~~~~~~~~~~~~~~~~~~~~~~~~~~~~~~~~~~~~~~~~~~~~~~~~~~~~~~~~~~~~~~~~~~~~~~~

                                      610       620       630       640       650       660       670       680       690       700
                             ....|....|....|....|....|....|....|....|....|....|....|....|....|....|....|....|....|....|....|
Aotus-1582-rc_[Anan_2.0]     gaaacattaattagtagcaggccgggcgcggtggctcaagcctgtaatcccagcactttgggaggccgaggcgggtggatcacaaggtcaagagatcgag
JW-1582-155159-SIntR-rc      GAAACATTAATTAGTAGCAGGCCGGGCGCGGTGGCTCAAGCCTGTAATCCCAGCACTTTGGGAGGCCGAGGCGGGTGGATCACAAGGTCAAGAGATCGAG
JW-1582-155159-R             GAAACATTAATTAGTAGCAGGCCGGGCGCGGTGGCTCAAGCCTGTAATCCCAGCACTTTGGGAGGCCGAGGCGGGTGGATCACAAGGTCAAGAGATCGAG
JW-1582-155159-IntF1         ~~~~~~~~~~~~~~~~~~~~~~~~~~~~~~~~~~~~~~~~~~~~~~~~~~~~~~~~~~~~~~~~~~~~~~~~~~~~~~~~~~~~~~~~~~~~~~~~CGAG
JW-1582-155159-F-rc          ~~~~~~~~~~~~~~~~~~~~~~~~~~~~~~~~~~~~~~~~~~~~~~~~~~~~~~~~~~~~~~~~~~~~~~~~~~~~~~~~~~~~~~~~~~~~~~~~~~~~
JW-1582-86218-SIntR-rc       GAAACATTAATTAGTAGCAGGCCGGGCACGGTGGCTCAAGCCTGTAATCCCAGCACTTTGGGAGGCCAAGGCGGGTGGATCACAAGGTCAAGAGATCGAG
JW-1582-86218-R              GAAACATTAATTAGTAGCAGGCCGGGCACGGTGGCTCAAGCCTGTAATCCCAGCACTTTGGGAGGCCAAGGCGGGTGGATCACAAGGTCAAGAGATCGAG
JW-1582-86218-IntF           ~~~~~~~~~~~~~~~~~~~~~~~~~~~~~~~~~~~~~~~~~~~~~~~~~~~~~~~~~~~~~~~~~~~~~~~~~~~~~~~~~~~~~~~~~~~~~~~~CGAG
JW-1582-86218-F-rc           ~~~~~~~~~~~~~~~~~~~~~~~~~~~~~~~~~~~~~~~~~~~~~~~~~~~~~~~~~~~~~~~~~~~~~~~~~~~~~~~~~~~~~~~~~~~~~~~~~~~~

                                      710       720       730       740       750       760       770       780       790       800
                             ....|....|....|....|....|....|....|....|....|....|....|....|....|....|....|....|....|....|....|
Aotus-1582-rc_[Anan_2.0]     accatcctggtcaacatggtgaaaccccgtctctactaacacaaaaaattagctgggcatggtggcacgtgcctctaatcccagctactcaggaggctga
JW-1582-155159-SIntR-rc      ACCATCCTGGTCAACATGGTGAAACCCCGTCTCTACTAACACAAAAAATTAGCTGGGCATGGTGGCGCGTGCCTGTAATCCCAGCTACTCAGGAGGCTGA
JW-1582-155159-R             ACCATCCTGGTCAACATGGTGAAACCCCGTCTCTACTAACACAAAAAATTAGCTGGGCATGGTGGCGCGTGCCTGTAATCCCAGCTACTCAGGAGGCTGA
JW-1582-155159-IntF1         ACCATCCTGGTCAACATGGTGAAACCCCGTCTCTACTAACACAAAAAATTAGCTGGGCATGGTGGCGCGTGCCTGTAATCCCAGCTACTCAGGAGGCTGA
JW-1582-155159-F-rc          ~~~~~~~~~~~~~~~~~~~~~~~~~~~~~~~~~~~~~~~~~~~~~~~~~~~~~~~~~~~~~~~~~~~~~~~~~~~~~~~~~~~~~~~~~~~~~~~~~~~~
JW-1582-86218-SIntR-rc       ACCATCCTGGTCAACATGGTGAAACCCCGTCTCTACTAACACAAAAAATTAGCTGGGCATGGTGGCGCGTGCCTGTAATCCCAGCTACTCAGGAGG
JW-1582-86218-R              ACCATCCTGGTCAACATGGTGAAACCCCGTCTCTACTAACACAAAAAATTAGCTGGGCATGGTGGCGCGTGCCTGTAATCCCAGCTACTCAGGAGGCTGA
JW-1582-86218-IntF           ACCATCCTGGTCAACATGGTGAAACCCCGTCTCTACTAACACAAAAAATTAGCTGGGCATGGTGGCGCGTGCCTGTAATCCCAGCTACTCAGGAGGCTGA
JW-1582-86218-F-rc           ~~~~~~~~~~~~~~~~~~~~~~~~~~~~~~~~~~~~~~~~~~~~~~~~~~~~~~~~~~~~~~~~~~~~~~~~~~~~~~~~~~~~~~~~~~~~~~~~~~~~

                                      810       820       830       840       850       860       870       880       890       900
                             ....|....|....|....|....|....|....|....|....|....|....|....|....|....|....|....|....|....|....|
Aotus-1582-rc_[Anan_2.0]     ggcaggagaattgcctgaacccaggaggcggaggttgcggtgagccgagatcgcgccattgcactccagcctgggtaacgagcgaaactccgttgccaaa
JW-1582-155159-SIntR-rc
JW-1582-155159-R             GGCAGGAGAATTGCCTGAACCCAGGAGGCGGAGGTTGCGGTGAGCCGAGATCGCGCCATTGCACTCCAGCCTGGGTAACGAGCGAAACTCCGTCACAAAA
JW-1582-155159-IntF1         GGCAGGAGAATTGCCTGAACCCAGGAGGCGGAGGTTGCGGTGAGCCGAGATCGCGCCATTGCACTCCAGCCTGGGTAACGAGCGAAACTCCGTCACAAAA
JW-1582-155159-F-rc          ~~~~~~~~~~~~~~~~~~~~~~~~~~~~~~~~~~~~~~~~~~~~~~~~~~~~~~~~~~~~~~~~~~~~~~~~~~~~~~~~~~~~~~~~~~~~~~~~AAAA
JW-1582-86218-SIntR-rc
JW-1582-86218-R              GGCAGGAGAATTGCCTGAACCCAGGAGGCGGAGGTTGCGGTGAGCCGAGATCGCGCCATTGCACTCCAGCCTGGGTAACGAGCGAAACTCCGTCGCAAAA
JW-1582-86218-IntF           GGCAGGAGAATTGCCTGAACCCAGGAGGCGGAGGTTGCGGTGAGCCGAGATCGCGCCATTGCACTCCAGCCTGGGTAACGAGCGAAACTCCGTCGCAAAA
JW-1582-86218-F-rc           ~~~~~~~~~~~~~~~~~~~~~~~~~~~~~~~~~~~~~~~~~~~~~~~~~~~~~~~~~~~~~~~~~~~~~~~~~~~~~~~~~~~~~~~~~~~~~~~~AAAA
```

```
                                     910       920       930       940       950       960       970       980       990      1000
                               ....|....|....|....|....|....|....|....|....|....|....|....|....|....|....|....|....|....|....|....|
Aotus-1582-rc_[Anan_2.0]       aaaaaaaaaaattagtagcaaCAGCTAAAAGCCGGGTGCCAACAGCTGAACAAAGTGACAggacaggaaggagaagaggaaatagTGCTCTGGGGCTAGA
JW-1582-155159-SIntR-rc
JW-1582-155159-R               AAAAAAAAAAAAA
JW-1582-155159-IntF1           AAAAAAAAAAAAAA
JW-1582-155159-F-rc            AAAAAAAAAAATTAGTAGCAACAGC
JW-1582-86218-SIntR-rc
JW-1582-86218-R                AAAAAAAAAAAAAAAAA
JW-1582-86218-IntF             AAAAAAAAAAAAAAAA
JW-1582-86218-F-rc             AAAAAAAAAAATTAGTAGCAACAGC
```

Figure S34b. Sequence alignment for *Alu* locus *Aotus*_1582 018497893.1:1531600-1533091. The reference genome [Anan_2.0] is shown at the top followed by samples *A. vociferans* MVZ155159 and 86218. PCR-based genotypes indicate that all *Aotus* individuals are homozygous absent (0/0) except for these two *A. vociferans* individuals who have a homozygous present (1/1) insertion pattern. DNA sequencing was performed to determine if they share the target *Alu* or perhaps have a near parallel insertion unique to *A. vociferans*. This alignment confirms that both *A. vociferans* samples share the target *Alu* with the reference *A. nancymaae* genome. The *Alu* starts at position 620 (grey highlight), TSDs are in yellow highlight. This locus illustrates an interesting case of incomplete lineage sorting (ILS), especially given that none of our *A. nancymaae* samples have the target reference genome insertion.

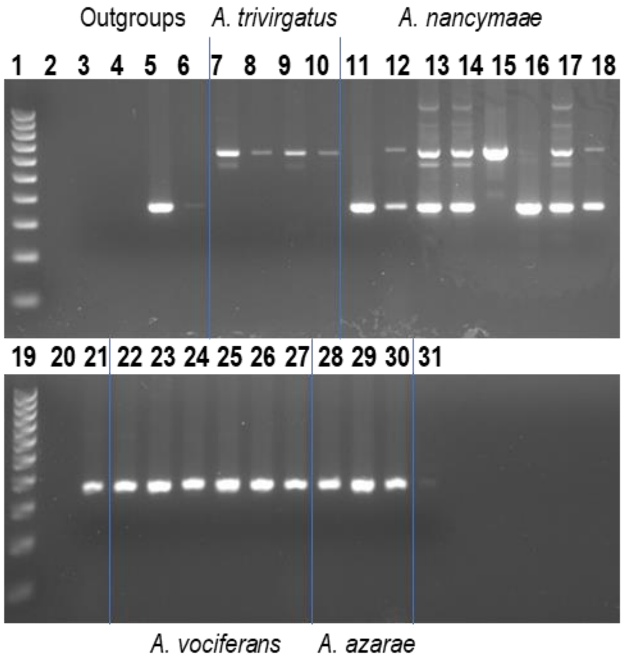

Figure S35a: *Alu* locus Owl_2LS_046_018514732.1:7169258-7170758 (LR-46): Lanes: 1, 19- 100 bp DNA ladder, 2, 20-blank, 3-TLE (negative control), 4- Human (HeLa), 5- *Callithrix jacchus* (Common marmoset), 6- *Saimiri s. sciureus* (Common squirrel monkey), 7-10 *A. trivirgatus,* 11-18, 21 *A. nancymaae,* 22-27 *A. vociferans,* 28-30 *A. azarae,* 31-*A. lemurinus griseimembra.* The predicted amplicon sizes are 668/363 bp. PCR based genotypes indicate that the target *Alu* is polymorphic among *A. nancymaae* individuals, homozygous absent (363 bp fragment) in *A. vociferans* and *A. azarae,* while homozygous present in all four *A. trivirgatus* individuals. This patten suggests that *A. trivirgatus* is most related to *A. nancymaae*. Perform DNA sequencing to confirm. See alignment Figure S35b.

```
                                  510       520       530       540       550       560       570       580       590       600
                          ....|....|....|....|....|....|....|....|....|....|....|....|....|....|....|....|....|....|....|....|
Owl_2LS_046 [Anan_2.0]    ctcaaaaacatTTCGACATTATAAAtgttagattttaattttaacaagtaAAAAATGCTACCACTTTGTAAAGTAACTTCAAGTCAAGGAATTTAAAGTC
LR-46-crl1556-F           CTCAAAAACATTTCGACATTATAAATGTTAGATTTTAATTTTAACAAGTAAAAAATGCTACCACTTTGTAAAGTAACTTCAAGTCAAGGAATTTAAAGTC
LR-46-crl1556-R-rc        TTCAAAAACATTTTGACATTATAAATGTTAGATTTTAATTTTAACAAGTAAAAAATGCTACCCCTTTGTAAAGTAACTTCAAGTCAAGGAATTTAAAGTC

                                  610       620       630       640       650       660       670       680       690       700
                          ....|....|....|....|....|....|....|....|....|....|....|....|....|....|....|....|....|....|....|....|
Owl_2LS_046_[Anan_2.0]    AAGAACgaggcggccgggcgcggtggctcaagcctgtaatcccagcactttgggaggccgaggcgggtggatcacgaggtcaagagatcgagaccatcct
LR-46-crl1556-F           ACTCATAGGAGGGTGTTGAACAAGGAGAACACATGGACACAGGGAGGGGAGCACTATACACGGGGGTCTGTTGCGGGAGAGTGGGGGGGGACAGCAGTGG
LR-46-crl1556-R-rc        ACTCATAGGAGGGTGTTGAACAAGGGGAACACATGGACACAGGGAGGGGAGCACTATACACGGGGGTTTGTTGCGGGAGAGTGGGGGGGGACAGCAGTGG

                                  710       720       730       740       750       760       770       780       790       800
                          ....|....|....|....|....|....|....|....|....|....|....|....|....|....|....|....|....|....|....|....|
Owl_2LS_046_[Anan_2.0]    ggtcaacatggtgaaaccccgtctctactaatacaaaaaattagctgggcacggtggcgcgtgcctgtaatcccagctactcaggaggctgaggcaggag
LR-46-crl1556-F           GGGAGTTGGGGAGGGATAGCAAGGGTAGAAATGCCAGATATGGGTGAGGGGGAGGGAGGCAGCAAATCACACTGCCATGAATGCACCTATGCAACAACTT
LR-46-crl1556-R-rc        GGGAGTTGGGGAGGGATAGCAAGGGTAGAAATGCCAGATATGGGTGAGGGGGAGGGAGGCAGCAAATCACACTGCCATGAATGCACCTATGCAACAACTT

                                  810       820       830       840       850       860       870       880       890       900
                          ....|....|....|....|....|....|....|....|....|....|....|....|....|....|....|....|....|....|....|....|
Owl_2LS_046_[Anan_2.0}    aattgcctgaacccaggaggcggaggttgcggtgagccgagatcgcgccattgcactccagcctgggtaacaaagcaagactctgtctcaaaaaaaaaaa
LR-46-crl1556-F           TGCATGTTTTTCACATGTACCCCAAAACCTAAAATGCAATAAAAAAAAATAATAAAAAAAATAAAAATAAAAAAAAAAAATAAAGTCAAAAACGAGGCATACT
LR-46-crl1556-R-rc        TGCATGTTTTTCACATGTACCCCAAAACCTAAAATGCAATAAAAAAAAATAATAAAAAAAATAAAAATAAAAAAAAAAAATAAAGTCAAGAACGAGGCATACT

                                  910       920       930       940       950       960       970       980       990       1000
                          ....|....|....|....|....|....|....|....|....|....|....|....|....|....|....|....|....|....|....|....|
Owl_2LS_046_[Anan_2.0]    aaaaaaaaagaacgaggCATACTGGTGCTCTCGCTAAAGGCCTTTTTGAGGGACGTTTAGCACCCTCTAGTGACAGCAATTCAAGCCGTTCAAAGGGAAC
LR-46-crl1556-F           GGTGAT~~~~~~~~~~~~~~~~~~~~~~~CTCGCTAAAGGCCTTTTTGAGGGACGTTTANCACCCTCTAGTGACAGCAATTCAAGCCGTTCAAAGGGAAC
LR-46-crl1556-R-rc        GGTGAT~~~~~~~~~~~~~~~~~~~~~~~CTCGCTAAAGGCCTTTTTGAGGGACGTTTAGCACCCTCTAGTGACAGCAATTCAAGCCGT~CAAAGG
```

Figure S35b. Sequence alignment for *Alu* locus Owl_2LS_046_018514732.1:7169258-7170758 (LR-46). PCR based genotypes indicate that the target *Alu* is polymorphic among *A. nancymaae* individuals, homozygous absent in *A. vociferans* and *A. azarae*, while homozygous present in all four *A. trivirgatus* individuals. This patten suggests that *A. trivirgatus* is most related to *A. nancymaae*. Perform DNA sequencing to confirm. The reference genome [Anan_2.0] is shown at the top, the target *Alu* starts at position 612 (grey highlight) and TSDs are shown in yellow highlight. While sequencing *A. trivirgatus* crl-1556 the internal-*Alu* primer sequences failed, meaning the *A. trivirgatus* filled site amplicon does not contain an *Alu* element. Instead, *A. trivirgatus* contains 306 bp of non-*Alu* sequence (aqua highlight), some of which is an L1, before matching flanking sequence resumes. *A. trivirgatus* is 0/0 for the target *Alu* based on this sequence alignment. However, we recommend not using this *Alu* locus for *Aotus* phylogeny due to the potential for confusing amplicons.

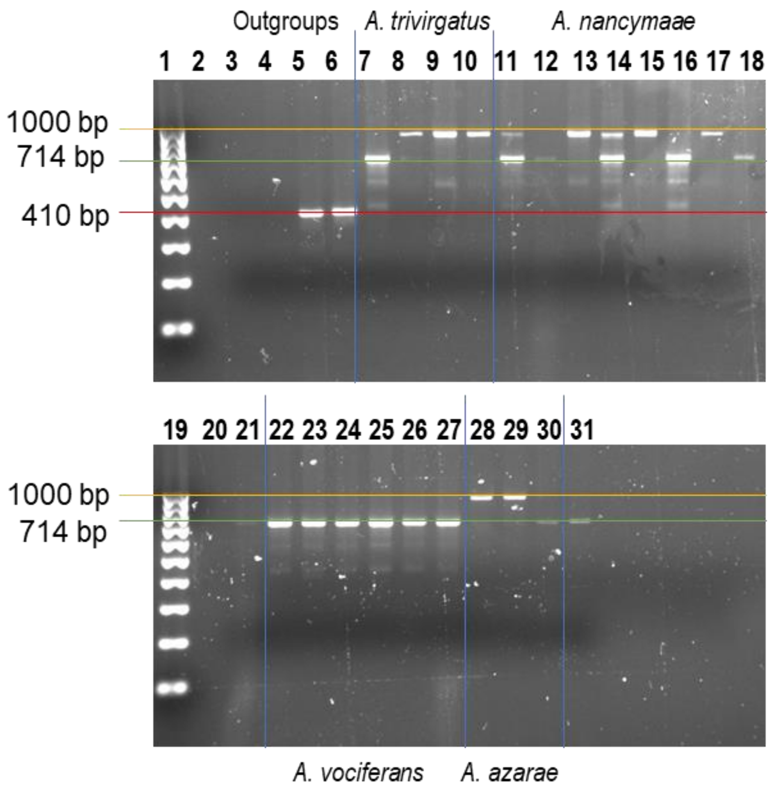

Figure S36a: *Alu* locus *Aotus*_1153 018486804.1:1542204-1543700 (JW-1153): Lanes: 1, 19- 100 bp DNA ladder, 2, 20-blank, 3-TLE (negative control), 4- Human (HeLa), 5- *Callithrix jacchus* (Common marmoset), 6- *Saimiri s. sciureus* (Common squirrel monkey), 7-10 *A. trivirgatus*, 11-18, 21 *A. nancymaae,* 22-27 *A. vociferans*, 28-30 *A. azarae,* 31-*A. lemurinus griseimembra*. Vertical blue lines superimposed on the gel image visually separate the species groups. The predicted amplicon sizes are 714 bp (green line) for the *A. nancymaae* reference genome containing a full length *Alu* insertion and 410 bp (red line) for the outgroups. There is a larger than predicted amplicon (~1000 bp, yellow line) in some *Aotus* individuals of *A. trivirgatus*, *A. nancymaae* and *A. azarae* but not in *A. vociferans*. DNA sequencing was performed to determine if this larger amplicon was a 2nd *Alu* insertion, in addition to the target insertion, that could perhaps be genotyped separately, as a surreptitious finding not ascertained from the reference genome (see alignment Figure S36b). This larger amplicon is not a 2nd *Alu* that can be genotyped separately. The target *Alu* is likely homozygous present in *Aotus*.

```
                                510       520       530       540       550       560       570       580       590       600
                           ....|....|....|....|....|....|....|....|....|....|....|....|....|....|....|....|....|....|....|....|
Aotus_1153_RC [Anan_2.0]   ggtacaaaaaaaaatagaaagaataagacctactatttgatagcacagtagggtgactatattcaataacaattgtacattttaaagtaacttaaaggtt
JW-1153-41436-SInR-rc      GGTACAAAAAAAAATAGAAAGAATAAGACCTACTATTTGATAGCACAGTAGGGTGACTATATTCAATAACAATTGTACATTTTAGAGTAACTTAAAGGTT
JW-1153-41436-F-rc         ~~~~~~~~~~~~~~~~~~~~~~~~~~~~~~~~~~~~~~~~~~~~~~~~~~~~~~~~~~~~~~~~~~~~~~~~~~~~~~~~~~~~~~~~~~~~~~~~~~~~
JW-1153-41436-IntF         ~~~~~~~~~~~~~~~~~~~~~~~~~~~~~~~~~~~~~~~~~~~~~~~~~~~~~~~~~~~~~~~~~~~~~~~~~~~~~~~~~~~~~~~~~~~~~~~~~~~~
JW-1153-85457-SIntR-rc     GGTACAAAAAAAAATAGAAAGAATAAGACCTACTATTTGATAGCACAGTCGGGTGACTATATTCAATAACAATTGTACATTTTAAAGTAACCTAAAGGTT
JW-1153-85457-F-rc         ~~~~~~~~~~~~~~~~~~~~~~~~~~~~~~~~~~~~~~~~~~~~~~~~~~~~~~~~~~~~~~~~~~~~~~~~~~~~~~~~~~~~~~~~~~~~~~~~~~~~
JW-1153-85457-R            ~~~~~~~~~~~~~~~~~~~~~~~~~~~ACCTACTATTTGATAGCACAGTAGGGTGACTATATTCAATAACAATTGTANATTTTAAAGTAACTTAAAGGTT
JW-1153-86116-SIntR-rc     ~~~~~~~~~~~~~~~~~~~~~~~~~~~~~~~~~~~~~~~~~~~~~~~~~~~~~~~~~TATATTCAATANCCNTTGTACATTTTAAAGTAACTTAAAGGTT
JW-1153-86116-F-rc         ~~~~~~~~~~~~~~~~~~~~~~~~~~~~~~~~~~~~~~~~~~~~~~~~~~~~~~~~~~~~~~~~~~~~~~~~~~~~~~~~~~~~~~~~~~~~~~~~~~~~
JW-1153-86116-IntF         ~~~~~~~~~~~~~~~~~~~~~~~~~~~~~~~~~~~~~~~~~~~~~~~~~~~~~~~~~~~~~~~~~~~~~~~~~~~~~~~~~~~~~~~~~~~~~~~~~~~~

                                610       620       630       640       650       660       670       680       690       700
                           ....|....|....|....|....|....|....|....|....|....|....|....|....|....|....|....|....|....|....|....|
Aotus_1153_RC [Anan_2.0]   ggccgggtgcggtggctcaagcctgtaatcccagcactttgggaggccgaggcgggtggatcacgaggtcaagagatcgagaccatcctggtcaacatgg
JW-1153-41436-SInR-rc      GGCCGGGCGCGGTGGCTCAAGCCTGTAATCCCAGCACTTTGGGAGGCCGAGGAAAAAAAAAAAAAAAAAAAAGAGATCGAGACCATCCCGGTCAACATGG
JW-1153-41436-F-rc         ~~~~~~~~~~~~~~~~~~~~~~~~~~~~~~~~~~~~~~~~~~~~~~~~~~~~~~~~~~~~~~~~~~~~~~~~~~~~~~~~~~~~~~~~~~~~~~~~~~~~
JW-1153-41436-IntF         ~~~~~~~~~~~~~~~~~~~~~~~~~~~~~~~~~~~~~~~~~~~~~~~~~~~~~~~~~~~~~~~~GAGGTCAAGAGATCGAGACCATCCCGGTCAACATTG
JW-1153-85457-SIntR-rc     GGCCGGGCGCGNTGGCTCAAGCCTGTAATCCCAGCAGTGTGGGAGGCCGAGAAGGAAGGATCAAAAAAAAAAGAGATCGAGACCATCCTGGTCAACATGG
JW-1153-85457-F-rc         ~~~~~~~~~~~~~~~~~~~~~~~~~~~~~~~~~~~~~~~~~~~~~~~~~~~~~~~~~~~~~~~~~~~~~~~~~~~~~~~~~~~~~~~~~~~~~~~~~~~~
JW-1153-85457-R            GGCCGGGCGCGGTGGCTCAAGCCTGTAATCCCAGCACTTTGGGAGGCCGAGGCGGGTGGATCACGAGGTCAAGAGATCGAGACCATCCTGGTCAACATGG
JW-1153-86116-SIntR-rc     GGCCGGGCGCGGTGGCTCAAGCCTGTAATCCCAGCACTTTGGGAGGCCGAAAAAAAAAAAAAAAAAAAAAAAGAGATCGAGACCATCCTGGTCAACATGG
JW-1153-86116-F-rc         ~~~~~~~~~~~~~~~~~~~~~~~~~~~~~~~~~~~~~~~~~~~~~~~~~~~~~~~~~~~~~~~~~~~~~~~~~~~~~~~~~~~~~~~~~~~~~~~~~~~~
JW-1153-86116-IntF         ~~~~~~~~~~~~~~~~~~~~~~~~~~~~~~~~~~~~~~~~~~~~~~~~~~~~~~~~~~~~~~~~GAGGTCAAGAGATCGAGACCATCCTGGTCAACATGG

                                710       720       730       740       750       760       770       780       790       800
                           ....|....|....|....|....|....|....|....|....|....|....|....|....|....|....|....|....|....|....|....|
Aotus_1153_RC [Anan_2.0]   tgaaaccctgtctctactaaaaatacaaaaaattagctgggcgtggtggcgcgtgcctgtaatcccagctactcaggaggctgaggcaggagaattgcct
JW-1153-41436-SInR-rc      TGAAACCCCGTCTCTACTAAAAATACAAAAAATTAGCTGGGCACGGTGGCGCGTGCCTGTAATCCCAGCTACTCAGGAGGCTGAGGCAG
JW-1153-41436-F-rc         ~~~~~~~~~~~~~~~~~~~~~~~~~~~~~~~~~~~~~~~~~~~~~~~~~~~~~~~~~~~~~~~~~~~~~~~~~~~~~~~~~~~~~~~~~~~~~~~~~~~~
JW-1153-41436-IntF         TGAAACCCCNTCTCTACTAAAAATACAAAAAATTACCTGGGCACGG
JW-1153-85457-SIntR-rc     TGAAACCCCGTCTCTACTAAAAATACAAAAAATTAGCTGGGCATGGTGGCGCGTGCCTGTAATCCCAGCTACTCAGGAGGCTGAGGCAG
JW-1153-85457-F-rc         ~~~~~~~~~~~~~~~~~~~~~~~~~~~~~~~~~~~~~~~~~~~~~~~~~~~~~~~~~~~~~~~~~~~~~~~~~~~~~~~~~~~~~~~~~~~~~~~~~~~~
JW-1153-85457-R            TGAAACCCCGTCTCTACTAAAAATACAAAAAATTAGCTGGGCACGGTGGCGCGTGCCTATAATCCCAGCTACTCAGGAGGCTGAGGCAGGAAAATTGCCT
JW-1153-86116-SIntR-rc     TGAAACCCCGTCTCTACTAAAAATACAAAAAATTAGCTGGGCACGGTGGCGCGTGCCTGTAATCCCAGCTACTCAGGAGGCTGAGGC
JW-1153-86116-F-rc         ~~~~~~~~~~~~~~~~~~~~~~~~~~~~~~~~~~~~~~~~~~~~~~~~~~~~~~~~~~~~~~~~~~~~~~~~~~~~~~~~~~~~~~~~~~~~~~~~~~~~
JW-1153-86116-IntF         TGAAACCCCNTCTCTACTAAAAATACAAAAAAGAACGAGG

                                810       820       830       840       850       860       870       880       890       900
                           ....|....|....|....|....|....|....|....|....|....|....|....|....|....|....|....|....|....|....|....|
Aotus_1153_RC [Anan_2.0}   gaacccaggaggcggaggttgcggtgagccgagatcgcgccattgcactccagcctgggtaacaagagcgaaactccatctcaaaaaaaaaaaaaaagta
JW-1153-41436-SInR-rc
JW-1153-41436-F-rc         ~~~~~~~~~~~~~~~~~~~~~~~~~~~~~~~~~~~~~~~~~~~~~~~~~~~~~~~~~~~~~~~~~~~~~~~~~~~~~~~~~~~~~~~~~~~~~~~~~~~~
JW-1153-41436-IntF
JW-1153-85457-SIntR-rc
JW-1153-85457-F-rc         ~~~~~~~~~~~~~~~~~~~~~~~~~~~~~~~~~~~~~~~~~~~~~~~~~~~~~~~~~~~~~~~~~~~~~~~~AAAAAAAATAAAAAAAAAAAAAAAAGTA
JW-1153-85457-R            GAACCCAGGAGGCGGAGGTTGCGTGGAGCCAAAATTGCGCCATTC
```

Figure S36b. Sequence alignment for *Alu* locus *Aotus*_1153 018486804.1:1542204-1543700 (JW-1153). PCR analyses revealed a larger than predicted amplicon in some *Aotus* samples but not in *A. vociferans* (see Figure S36a). DNA sequencing was performed to determine if this larger amplicon was a 2nd *Alu* insertion, in addition to the target insertion, that could perhaps be genotyped separately, as a surreptitious finding not ascertained from the reference genome. The reference genome [Anan_2.0] is shown at the top followed by samples *A. trivirgatus* 41436, *A. azarae* 85457, and *A. nancymaae* 86116. The target *Alu* starts at position 601 (grey highlight) and is shared by the sequenced individuals. The SIntR signal gets messy going out the 5' end of the target *Alu* suggesting that the 5' flanking has extra sequence; the reverse primer entering the 5' *Alu* fails or is very messy. The target *Alu* is likely homozygous present in *Aotus*. A 2nd *Alu* is unlikely or perhaps inserted within the target (A-stretch in aqua highlight) unable to be genotyped independently. RepeatMasker indicates that the target *Alu* is inside an L1. Therefore, we recommend not using this locus for *Aotus* phylogeny.

```
                                510       520       530       540       550       560       570       580       590       600
                        ....|....|....|....|....|....|....|....|....|....|....|....|....|....|....|....|....|....|....|....|
JW_1422-rc_[Anan_2.0]   CTGTGCTCAATAATACCATATTTGGCTTTTGGTTAACTAGTCTGACAGATaagaaacttttattatttaagtatAATGCTGCTTTAAGATGATGggatat
JW-1422-86230-R         CTGTGCTCAATAATACTATATTTGGCTTTTGGTTAACTAGTCTGACAGATAAGAAACTTTTATTATTTAAGTATAATGCTGCTCAATAATACCATATTTG
JW-1422-86230-F-rc      CTGTGCTCAATAATACTATATTTGGCTTTTGGTTAACTAGTCTGACAGATAAGAAACTTTTATTATTTAAGTATAATGCTGCTCAATAATACCATATTTG

                                610       620       630       640       650       660       670       680       690       700
                        ....|....|....|....|....|....|....|....|....|....|....|....|....|....|....|....|....|....|....|....|
JW_1422-rc_[Anan_2.0]   ggccgggcgcggtggctcaagcctgtaatcccagcactttgggaggccgaggcgggtggatcacgaggtcaagagatcgagaccatcctggtcaacattg
JW-1422-86230-R         GCTTTTGGTTAACTAGTCTGACAGATAAGAAACTTTTATTATTTAAGTATAATGCTGCTCAATAATACCATATTTGGCTTTTGGTTAACTAGTCTGACAG
JW-1422-86230-F-rc      GCTTTTGGTTAACTAGTCTGACAGATAAGAAACTTTTATTATTTAAGTATAATGCTGCTCAATAATACCATATTTGGCTTTTGGTTAACTAGTCTGACAG

                                710       720       730       740       750       760       770       780       790       800
                        ....|....|....|....|....|....|....|....|....|....|....|....|....|....|....|....|....|....|....|....|
JW_1422-rc_[Anan_2.0]   tgaaaccctgtctctactaatacaaaaaaattagctgggtatggtggcgcgtgcctgtaatcccagctactcaggaggctgaggcaggagaattgcctga
JW-1422-86230-R         ATAAGAAACTTTTATTATTTAAGTATAATGCTGCTTTAAG~~~~~~~~~~~~~~~~~~~~~~~~~~~~~~~~~~~~~~~~~~~~~~~~~~~~~~~~~~~~
JW-1422-86230-F-rc      ATAAGAAACTTTTATTATTTAAGTATAATGCTGCTTTAAG~~~~~~~~~~~~~~~~~~~~~~~~~~~~~~~~~~~~~~~~~~~~~~~~~~~~~~~~~~~~

                                810       820       830       840       850       860       870       880       890       900
                        ....|....|....|....|....|....|....|....|....|....|....|....|....|....|....|....|....|....|....|....|
JW_1422-rc_[Anan_2.0]   acccaggaggcggaggttgcggtgagccgagatcgcgccattgcactccagcctgggtaacgagagcgaaactccgtctcaaaaaaaaaaagatgatggg
JW-1422-86230-R         ~~~~~~~~~~~~~~~~~~~~~~~~~~~~~~~~~~~~~~~~~~~~~~~~~~~~~~~~~~~~~~~~~~~~~~~~~~~~~~~~~~~~~~~~~~~~ATGATGGG
JW-1422-86230-F-rc      ~~~~~~~~~~~~~~~~~~~~~~~~~~~~~~~~~~~~~~~~~~~~~~~~~~~~~~~~~~~~~~~~~~~~~~~~~~~~~~~~~~~~~~~~~~~~ATGATGGG

                                910       920       930       940       950       960       970       980       990      1000
                        ....|....|....|....|....|....|....|....|....|....|....|....|....|....|....|....|....|....|....|....|
JW_1422-rc_[Anan_2.0]   aTATGCAGTATCACCTCACAAAAGAACTCCAAATTACTTAGCAGTTGCAGTTTTGTCAATCAGAGCATTATAGTATGAATATGAAATGTTAGTGAGTTCA
JW-1422-86230-R         ATATGCAGTATCACCTCACAAAAGAACTCCAAATTACTTAGCAGTTGCAGTTTTGTCAATCAGAGCATTATAGTATGAATATGAAATGTTAGTGAGTTCA
JW-1422-86230-F-rc      ATATGCAGTATCACCTCACAAAAGAACTCCAAATTACTTAGCAGTTGCAGTTTTGTCAATC
```

Figure S37. Sequence alignment for *Alu* locus *Aotus*_1422 018505596.1:1353282-1354772 (JW-1422). The predicted PCR fragment

sizes are 565/256 bp. PCR based genotypes indicate the target *Alu* is restricted to *A. nancymaae*, however the *A. vociferans* individuals have amplicon that is a different size, about 400 bp. DNA sequencing was performed to determine if this fragment contained the target *Alu* with some missing sequence or is an artifact. The reference genome [Anan_2.0] is shown at the top followed by *A. vociferans* 86230. The target *Alu* starts at position 601 (grey highlight) and the TSDs are shown in yellow highlight. During sequencing of *A. vociferans* 86230 the internal-*Alu* primers failed, meaning no *Alu* element is present. Instead, *A. vociferans* has ~140 bp of extra non-repetitive sequence (aqua highlight) in this region resulting in the observed ~400 bp amplicon. Therefore, *A. vociferans* are 0/0 for the target insertion.

```
                                    510       520       530       540       550       560       570       580       590       600
                           ....|....|....|....|....|....|....|....|....|....|....|....|....|....|....|....|....|....|....|....|
Owl_2LS_110_[Anan_2.0]     ATCAAAGAAATGGGCAGGATATGTGTCCGTGGCCCACACTGAAGAGCAGGTGAAATCATCAAGGTGGATAAATAAGGATAttgttaaaaaacagaaaaaa
LR-110-41436-SIntR-rc      ATCAAAGAAATGGGCAGGATATGTGCCCGTGGCCCACACTGAAGAGCAGGTGAAATCATCAAGGTGGATAAATAAGGATATTGTTAAAAAGCAGAAAAAA
LR-110-41436-IntF          ~~~~~~~~~~~~~~~~~~~~~~~~~~~~~~~~~~~~~~~~~~~~~~~~~~~~~~~~~~~~~~~~~~~~~~~~~~~~~~~~~~~~~~~~~~~~~~~~~~~
LR-110-41436-R-rc          ~~~~~~~~~~~~~~~~~~~~~~~~~~~~~~~~~~~~~~~~~~~~~~~~~~~~~~~~~~~~~~~~~~~~~~~~~~~~~~~~~~~~~~~~~~~~~~~~~~~
LR-110-41436-E-F           ATCAAAGAAATAGGCAGGATATGTGTCCGTGGCCCACACTGAAGAGCAGGTGAAATCATCAAGGTGGATAAATAAGGATAC
LR-110-41436-E-R-rc        ATCAAAGAAATAGGCAGGATATGTGTCCGTGGCCCACACTGAAGAGCAGGTGAAATCATCAAGGTGGATAAATAAGGATACTGTTAAAAAACAGAAAA~~

                                    610       620       630       640       650       660       670       680       690       700
                           ....|....|....|....|....|....|....|....|....|....|....|....|....|....|....|....|....|....|....|....|
Owl_2LS_110_[Anan_2.0]     gggccgggcgcggtggctcatgcctgtaatcccagcactttgggaggccgaggcgggtggatcacgaggtcaagacatcgagaccatcctggtcaacatg
LR-110-41436-SIntR-rc      GGGCCGGGCGCGGTGGCTCATGCCTGTAATCCCAGCACTTTGGGAGGCCGAGGCGGGTGGATCACGAGGTCAAGAGATCGAGACCATCCTGGTCAACATG
LR-110-41436-IntF          ~~~~~~~~~~~~~~~~~~~~~~~~~~~~~~~~~~~~~~~~~~~~~~~~~~~~~~~~~~~~~~~~~GAGGTCAAGAGATCGAGACCATCCTGGTCAACATG
LR-110-41436-R-rc          ~~~~~~~~~~~~~~~~~~~~~~~~~~~~~~~~~~~~~~~~~~~~~~~~~~~~~~~~~~~~~~~~~~~~~~~~~~~~~~~~~~~~~~~~~~~~~~~~~~~
LR-110-41436-E-F
LR-110-41436-E-R-rc        ~~~~~~~~~~~~~~~~~~~~~~~~~~~~~~~~~~~~~~~~~~~~~~~~~~~~~~~~~~~~~~~~~~~~~~~~~~~~~~~~~~~~~~~~~~~~~~~~~~~

                                    710       720       730       740       750       760       770       780       790       800
                           ....|....|....|....|....|....|....|....|....|....|....|....|....|....|....|....|....|....|....|....|
Owl_2LS_110_[Anan_2.0]     gtgaaaccccgtctctactaatacaaaacattagctgggcatggtggcgcgtgcctgtaatcccagctattcaggaggctgaggcaggagaattgcctga
LR-110-41436-SIntR-rc      GTGAAACCCCGTCTCTACTAATACAAAACATTAGCTGGGCATGGTGGCGCGTGCCTGTAATCCCAGCTACTCAGGAGG
LR-110-41436-IntF          GTGAAACCCCNTCTCNACTAATACAAAACATTAGCTGGGCATGGTGGCGCGTGCCTGTAATCCCAGCTACTCAGGAGGCTGAGGCAGGAGAATTGCCTGA
LR-110-41436-R-rc          ~~~~~~~~~~~~~~~~~~~~~~~~~~~~~~~~~~~~~~~~~~~~~~~~~~~~~~~~~~~~~~~~~~~~~~~~~~~~~~~~~~~~~~~~~~~~~~~~~~~
LR-110-41436-E-F
LR-110-41436-E-R-rc        ~~~~~~~~~~~~~~~~~~~~~~~~~~~~~~~~~~~~~~~~~~~~~~~~~~~~~~~~~~~~~~~~~~~~~~~~~~~~~~~~~~~~~~~~~~~~~~~~~~~

                                    810       820       830       840       850       860       870       880       890       900
                           ....|....|....|....|....|....|....|....|....|....|....|....|....|....|....|....|....|....|....|....|
Owl_2LS_110_[Anan_2.0]     acgcaggaggcggaggttgcggtgagccgagattgcgccattgcactccaggctgggtaacaagagcgaaactccgtctcaaaaaaaaaaaacagaaaaa
LR-110-41436-SIntR-rc
LR-110-41436-IntF          ACCCAGGAGGTGGAGGTTGCGGTGAGCCGAGATCACGCCATTGCACTCCAGCCTGGGTAACAAGAA
LR-110-41436-R-rc          ~~~~~~~~~~~~~~~~~~~~~~~~~~~~~~~~~~~~~~~~~~~~~~~~~~~~~~~~~~~~~~~~~~~~~~~~~~~~~~~~~~AAAAAAAAAACAGAAAAA
LR-110-41436-E-F
LR-110-41436-E-R-rc        ~~~~~~~~~~~~~~~~~~~~~~~~~~~~~~~~~~~~~~~~~~~~~~~~~~~~~~~~~~~~~~~~~~~~~~~~~~~~~~~~~~~~~~~~~~~~~~~~~~A
```

```
                                      910       920       930       940       950       960       970       980       990       1000
                                  ....|....|....|....|....|....|....|....|....|....|....|....|....|....|....|....|....|....|....|....|
Owl_2LS_110_[Anan_2.0]            caactaaaGCACTGCATTTGCCCTTTAAAGTTTCACGTGTTTATCTTAACTGCCCCATAAAGACAATTGGGACACATGTCCATGGATCACGTACCTGCTT
LR-110-41436-SIntR-rc
LR-110-41436-IntF
LR-110-41436-R-rc                 CAACTAAAGCACTGCAT
LR-110-41436-E-F
LR-110-41436-E-R-rc               CAACTAAAGCACTGC
```

Figure S38. Sequence alignment for *Alu* locus Owl_2LS_110_018501792.1:6759460-6760958 (LR-110): PCR-based genotypes indicate that *A. trivirgatus* MSB-41436 is the only *Aotus* sample that is not homozygous present (1/1) for the target insertion. DNA sequencing was performed for both the filled and empty amplicon fragments to confirm the heterozygous genotype. The reference genome [Anan_2.0] is shown at the top followed by *A. trivirgatus* MSB-41436 who shares the target *Alu* insertion starting at position 602 (grey highlight) and flanked by TSDs in yellow highlight, while the empty site sequence displays a precise pre-integration site spanning the target region, confirming the genotype.

```
                                      510       520       530       540       550       560       570       580       590       600
                                  ....|....|....|....|....|....|....|....|....|....|....|....|....|....|....|....|....|....|....|....|
Aotus_1765 [Anan_2.0]             AAATAGTGTCCTCTTTGTGTGTGAGTCTCTGTGCCCAGACATatagtggggtgtgtgtg-ttgtgtgtgtgtgttttgaaatattttatattaaaaaatt
JW-1765-85464-E-F                 AAATAGTGTCCTCTTTGTGTGTGAGTCTCTGTGCCCAGACATATAGTGGGGTGTGTGTGTTTGTGTGTGTGTGTTTTGAAATATTTTATATTAAAAAATG
JW-1765-85464-E-R-rc              AAATAGTGTCCTCTTTGTGTGTGAGTCTCTGTGCCCAGACATATAGTGGGGTGTGTGTGTTTGTGTGTGTGTGTTTTGAAATATTTTATATTAAAAAATG
JW-1765-85464-R-rc                ~~~~~~~~~~~~~~~~~~~~~~~~~~~~~~~~~~~~~~~~~~~~~~~~~~~~~~~~~~~~~~~~~~~~~~~~~~~~~~~~~~~~~~~~~~~~~~~~~~~~
JW-1765-85464-SIntR-rc            AAATAGTATCCTCTTTGTGTGTGAGTCTCTGTGCCCAGACATATAGTGGGGTGTGTTTGTGTGTGTG~~~~TTTTGAAATATTTTATATTAAAAAATT
JW-1765-85464-IntF                ~~~~~~~~~~~~~~~~~~~~~~~~~~~~~~~~~~~~~~~~~~~~~~~~~~~~~~~~~~~~~~~~~~~~~~~~~~~~~~~~~~~~~~~~~~~~~~~~~~~~

                                      610       620       630       640       650       660       670       680       690       700
                                  ....|....|....|....|....|....|....|....|....|....|....|....|....|....|....|....|....|....|....|....|
Aotus_1765 [Anan_2.0]0            aggccgggcgcggtggctcaagcctgtaatcccagcactttgggaggccaaggcgggtggatcacgaggtcaagagatcaagaccatcctggtcaacatg
JW-1765-85464-E-F                 A~~~~~~~~~~~~~~~~~~~~~~~~~~~~~~~~~~~~~~~~~~~~~~~~~~~~~~~~~~~~~~~~~~~~~~~~~~~~~~~~~~~~~~~~~~~~~~~~~~~
JW-1765-85464-E-R-rc              A~~~~~~~~~~~~~~~~~~~~~~~~~~~~~~~~~~~~~~~~~~~~~~~~~~~~~~~~~~~~~~~~~~~~~~~~~~~~~~~~~~~~~~~~~~~~~~~~~~~
JW-1765-85464-R-rc                ~~~~~~~~~~~~~~~~~~~~~~~~~~~~~~~~~~~~~~~~~~~~~~~~~~~~~~~~~~~~~~~~~~~~~~~~~~~~~~~~~~~~~~~~~~~~~~~~~~~~
JW-1765-85464-SIntR-rc            AGGCCGGGCGCGGTGGCTCAAGCCTGTAATCCCAGCACTTTGGGAGGCCAAGGCGGGTGGATCACGAGGTCAAGAGATCAAGACCATCCTGGTCAACATG
JW-1765-85464-IntF                ~~~~~~~~~~~~~~~~~~~~~~~~~~~~~~~~~~~~~~~~~~~~~~~~~~~~~~~~~~~~~~~~~GAGGTCA~GAGATCAAGACCATCCTGGTCAACATG

                                      710       720       730       740       750       760       770       780       790       800
                                  ....|....|....|....|....|....|....|....|....|....|....|....|....|....|....|....|....|....|....|....|
Aotus_1765 [Anan_2.0]             gtgaaaccccgtctctactaatacaaaaaaaattagctgggcatggtggcgcgtgcctgtaatcgcagctactcaggaggctgaggcaggagaattgcct
JW-1765-85464-E-F                 ~~~~~~~~~~~~~~~~~~~~~~~~~~~~~~~~~~~~~~~~~~~~~~~~~~~~~~~~~~~~~~~~~~~~~~~~~~~~~~~~~~~~~~~~~~~~~~~~~~~~
JW-1765-85464-E-R-rc              ~~~~~~~~~~~~~~~~~~~~~~~~~~~~~~~~~~~~~~~~~~~~~~~~~~~~~~~~~~~~~~~~~~~~~~~~~~~~~~~~~~~~~~~~~~~~~~~~~~~~
JW-1765-85464-R-rc                ~~~~~~~~~~~~~~~~~~~~~~~~~~~~~~~~~~~~~~~~~~~~~~~~~~~~~~~~~~~~~~~~~~~~~~~~~~~~~~~~~~~~~~~~~~~~~~~~~~~~
JW-1765-85464-SIntR-rc            GTGAAACCCCGTCTCTACTAATACAAAAAAAATNAGCTGGGCATGGTGGCGCGTGCCTGTAATCGCAGCTACTCAGGAGG
JW-1765-85464-IntF                GTGAAACCCCGTCNCTANNAATACAAAAAAAATTAGCTGGGCATGGTGGCGCGNNCNTGNAATCGCAGCTACTCAGGAGGCTGAGGCAGGAGAATTGCCT
```

```
                                         810       820       830       840       850       860       870       880       890       900
                                ....|....|....|....|....|....|....|....|....|....|....|....|....|....|....|....|....|....|....|....|
Aotus_1765 [Anan_2.0]          gaacccaggaggcggaggttgcggtgagccgagatcgcgccattgcactccagcctgggtaacaagagcgaaactccgtctcaaaaaaaaaaaaaaaaaa
JW-1765-85464-E-F               ~~~~~~~~~~~~~~~~~~~~~~~~~~~~~~~~~~~~~~~~~~~~~~~~~~~~~~~~~~~~~~~~~~~~~~~~~~~~~~~~~~~~~~~~~~~~~~~~~~~~
JW-1765-85464-E-R-rc            ~~~~~~~~~~~~~~~~~~~~~~~~~~~~~~~~~~~~~~~~~~~~~~~~~~~~~~~~~~~~~~~~~~~~~~~~~~~~~~~~~~~~~~~~~~~~~~~~~~~
JW-1765-85464-R-rc              ~~~~~~~~~~~~~~~~~~~~~~~~~~~~~~~~~~~~~~~~~~~~~~~~~~~~~~~~~~~~~~~~~~~~~~~~~~~~~~~~~~~~~~~~~~~~~AAAAAAA
JW-1765-85464-SIntR-rc
JW-1765-85464-IntF              GAACCCAGGAGGCGGAGGTTGCGGTGAGCCTANATCGCGCCATTGCACTCCAGCCTGGGTAACAAGAGCGAAACTCCATCTCAAAAAAAAAAAAAAAAAA

                                         910       920       930       940       950       960       970       980       990       1000
                                ....|....|....|....|....|....|....|....|....|....|....|....|....|....|....|....|....|....|....|....|
Aotus_1765 [Anan_2.0]          aaaatgacaaaaaattataGAGAGCTCCCATGTAATTTTCATTCAACTTCCCCCAATGATAAAATCTTGTATTACCGTAGTGCAACTATCAAAGCCAGAT
JW-1765-85464-E-F               ~~~~~~~CAAAAAATTATAGAGAGCTCCCATGTAATTTTCATTCAACTTCCCCCAATGATAAAATCTTGTATTACCGTAGTGCAACTATCAAAGCCAGAT
JW-1765-85464-E-R-rc            ~~~~~~~CAAAAAATTATAGAGAGCTCCCATGTAATTTTCATTCAACTTCCCCCAATGATAAAATCTTGTATTACCGTAGTGCAACTATCAAAGCCAGAT
JW-1765-85464-R-rc              AAAATGACAAAAAATTATAGAGAGCTCCCATGTAATTTTCATTCAACTTCCCCCAATGATAAAATCTTGTATTACCGTAGTGCAACTATCAAAGCCAGAT
JW-1765-85464-SIntR-rc
JW-1765-85464-IntF              AAA
```

Figure S39. Sequence alignment for *Alu* locus *Aotus*_1765_ 018493376.1:4506820-4508322 (JW-1765). PCR-based genotypes indicate the target *Alu* insertion is homozygous present (1/1) in all *Aotus* samples except for *A. nancymaae* 85464 who appears heterozygous (1/0) for the insertion. The reference genome [Anan_2.0] is shown at the top followed by sequence traces for the empty site amplicon in *A. nancymaae* 85464 followed by filled site traces, The target *Alu* elements starts at position 602 (grey highlight) and flanked by TSDs in yellow highlight. The filled site amplicon shares the target *Alu* while the empty site displays a precise pre-integration site spanning the target region, confirming the genotype.
